# Reactions of Ta^+^ with Acetylene Studied with Infrared Photodissociation Spectroscopy and Theory

**DOI:** 10.1021/acs.jpca.6c05456

**Published:** 2026-10-21

**Authors:** Jens van der Hulst, Richard B. Odonkor, Nathan J. Dynak, Michael A. Duncan

**Affiliations:** † Department of Chemistry, 1355 University of Georgia, Athens, Georgia 30602, United States; ‡ Faculty of Science, Radboud University, Postbus 9010, 6500 GL Nijmegen, The Netherlands

## Abstract

Tantalum cation complexes with up to four acetylene molecules are investigated using mass spectrometry and infrared photodissociation spectroscopy. The ion complexes are produced by laser ablation in an acetylene/argon molecular beam and analyzed using a time-of-flight mass spectrometer. After mass selection, infrared photodissociation spectra recorded in the C–H and C–C stretching region are compared with spectra predicted by DFT for different isomers. Ions of the form Ta^+^(C_2_H_2_) and Ta^+^(C_2_H_2_)_*n*_–H_2_ (*n* = 2–4) are observed, revealing efficient dehydrogenation and ligand coupling from the second acetylene molecule onward. Infrared spectroscopy enables the identification of these ligand-coupling products, revealing unexpected dehydrogenated metallacycle, acetylide, and metal hydride structures for *n* = 2 and 3. The third acetylene ligand completes the metal coordination sphere, resulting in an externally bound fourth acetylene ligand. Overall, this study demonstrates the remarkable efficiency of tantalum in promoting acetylene dehydrogenation and ligand coupling.

## Graphical abstract

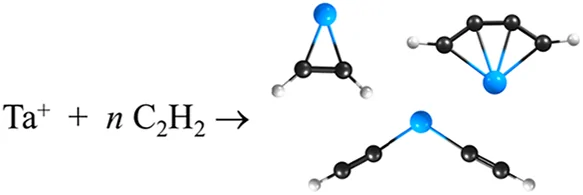

## Introduction

Metal–ligand interactions have been studied extensively due to their importance in organometallic chemistry, catalysis, and hydrocarbon activation processes.^1–9^ In particular, interactions between transition metals and hydrocarbons often promote C–H activation, dehydrogenation, cation-π coordination, and ligand coupling reactions.^1–9^ One of these coupling reactions, the cycloaddition of acetylene to form benzene, is well-known and has been studied extensively.^1–4^ Due to the strong chemical bonds in acetylene, this reaction has high activation barriers. Consequently, it typically requires specialized catalysts combined with high temperature and pressure conditions. Gas-phase studies of transition-metal ion complexes with small hydrocarbons such as acetylene provide powerful model systems for investigating intrinsic bonding, structure, coordination, and reaction mechanisms under isolated conditions, free from the complications of counterions, solvents, and matrices.^10–43^ These studies are complemented with computational studies to provide detailed insights into the electronic structures and geometries of these complexes. Additionally, electronic spectroscopy,^44–55^ infrared spectroscopy,^56–72^ and photofragment imaging measurements^55,73^ have further investigated the bonding interactions and structures of metal ion-acetylene complexes. For complexes containing multiple acetylene units, ligand-coupling reactions may be possible, from which the products can be identified using infrared spectroscopy.^60,64,66,72^ Experimental and theoretical work up to this point has suggested that reactions in these systems are more efficient for early transition metal systems. Therefore, in this work, we use mass spectrometry, infrared photodissociation spectroscopy, and density functional theory (DFT) calculations to study the structures and coordination of Ta^+^(C_2_H_2_)_*n*_ complexes and their dehydrogenated forms.

Tantalum cations are known to exhibit high reactivity toward small hydrocarbons.^10,18,31,41–43,60,66^ Previous studies have shown that Ta^+^ reacts readily with methane, ethane, and ethylene through stepwise dehydrogenation pathways, leading to the formation of carbides, carbenes, and metallacycle products. Early studies by Freiser and co-workers showed that the reaction between Ta^+^ and methane produces Ta^+^(CH_2_) and subsequently undergoes additional reactions with three methane molecules to form Ta^+^(C_4_H_8_) species.^10^ Similar dehydrogenation chemistry was observed by Irikura and Beauchamp,^11,18^ where they reported that Ta^+^ dehydrogenates methane more efficiently than first and second-row transition metals. Further ion beam experiments by Armentrout and co-workers provided additional insight into the Ta^+^CH_2_ structure, with theoretical investigations revealing a surface crossing between the singlet and triplet electronic states that leads to the exothermic formation of the dehydrogenated species.^42^ The reactions of Nb^+^ and Ta^+^ with various alkanes and alkenes were reported by Freiser and co-workers,^21^ who showed that Ta^+^ undergoes dehydrogenation more efficiently than Nb^+^. More recently, Infrared Multiphoton Dissociation (IR-MPD) studies carried out by Bakker and co-workers show that the reactions of Ta^+^ with ethylene led to the formation of Ta^+^(C_2_H_2_) and Ta^+^(C_2_H_2_)_2_ metallacyclopropene products through hydrocarbon activation processes.^66^ Even though much research has focused on dehydrogenated tantalum-cation hydrocarbon systems, there is no previous spectroscopy of tantalum ions reacting with acetylene.

Our lab has carried out extensive studies on metal-ion acetylene and ethylene complexes using infrared laser photodissociation spectroscopy.^56–59,61–65,67–72^ For complexes containing multiple acetylene ligands, infrared spectroscopic methods are particularly valuable for providing structural insight into the nature of the coordinated hydrocarbon species. Coordination of acetylene to metal cations is generally stabilized through cation–π interactions. This perturbs the ligand via charge-transfer interactions, leading to red shifts in the C–H stretching frequencies. As more acetylene ligands bind to the metal center, competing isomeric structures and possible ligand-coupling pathways emerge. For many metal ions (Cu^+^, Au^+^, Ag^+^, Co^+^, Fe^+^, and Pt^+^), unreacted cation–π complexes were observed with three or four acetylene ligands completing the solvation sphere around the metal ion.^61,62,68–71^ For certain metal ions, acetylene complexes exhibit ligand-coupling chemistry.^64,67,72^ The reactions for V^+^(acetylene)_*n*_ complexes proceeded via the formation of a five-membered metallacycle intermediate and subsequent formation of benzene.^64^ Zn^+^(acetylene)_*n*_ complexes reacted to form polyacetylene structures.^67^ Ti^+^(C_2_H_2_) complexes formed a five-membered metallacycle, but were not observed to form benzene.^72^ The present study reports the selected-ion infrared photodissociation spectra of Ta^+^(C_2_H_2_) and Ta^+^(C_2_H_2_)_*n*_–H_2_ complexes. Experiments and their comparison with DFT computations are used to investigate the possible acetylene coupling behavior and identify reaction products in these systems.

## Experimental Section

Tantalum cation-hydrocarbon complexes of the form Ta^+^(C_2_H_2_) and Ta^+^(C_2_H_2_)*_n_*–H_2_ were produced in a pulsed nozzle laser vaporization source^74^ using the third harmonic of a Nd:YAG laser (355 nm; Spectra-Physics INDI) in a gas mixture of acetylene in argon. The laser was focused onto a rotating and translating 1/4 in. diameter tantalum rod mounted with a pulsed nozzle (General Valve Series 9) with a sample holder in the so-called “offset” configuration.^74^ The acetylene complexes and dehydrogenated reaction products formed spontaneously by reactions within the supersonic expansion of metal vapor and acetylene. The supersonic expansion was skimmed into a differentially pumped chamber, where the resulting positive ions were extracted and detected using a reflectron time-of-flight mass spectrometer for mass analysis and for mass selecting specific cluster sizes. Photodissociation measurements used excitation of the ion beam at the turning point in the reflectron field.^75,76^

Mass-selected ions were studied via infrared photodissociation spectroscopy in the C–H stretching region (2900–3400 cm^–1^) and the C–C stretching region (1750–2050 cm^–1^). Tunable infrared radiation was provided by an optical parametric oscillator (OPO/OPA; LaserVision, Inc.) pumped by a Nd:YAG laser (Spectra-Physics Pro-250). The C–C stretching region was reached by addition of an external AgGaSe_2_ crystal. This OPO system has a line width of about 2 cm^–1^. Argon tagging was used to enhance the IR photodissociation since the acetylene ligand binding energy usually exceeds the IR photon energy. Excitation of the ligand vibrational modes resulted in the loss of argon from the tagged complexes. The infrared spectra were generated by monitoring the fragment-ion yield as the IR frequency was scanned. Frequencies were calibrated with photoacoustic spectroscopy measurements of the rotationally resolved antisymmetric C–H stretch vibration of methane.

Computational studies on the Ta^+^(C_2_H_2_) and Ta^+^(C_2_H_2_)_*n*_–H_2_ complexes were performed with the Gaussian 16 software package,^77^ using density functional theory and the B3LYP functional with the cc-pVTZ-pp basis set, which includes the ECP60MDF Stuttgart/Köln pseudopotential for tantalum.^78^ The all-electron cc-pVTZ basis set was used for carbon and argon, while for hydrogen a cc-pVDZ basis set was used.^79^ The relative energies reported include zero-point energy corrections. For comparison with the experimental spectra, the calculated frequencies were scaled by a factor of 0.964, based on the comparison of the computed asymmetric C–H stretch of acetylene with the known values from NIST,^80^ and given a Lorentzian line shape with a line width of roughly 5 cm^–1^. All optimized structures were confirmed to be true minima with no imaginary vibrational frequencies. Computations were carried out on argon-tagged complexes and the corresponding tag-free ions to investigate the effects of the tagging on vibrational frequencies. For the computations on reaction coordinates, we used the synchronous-transition-guided quasi-Newton (STQN) method in Gaussian 16 with the QST3 command. Transition states were confirmed with subsequent TS and IRC calculations.

## Results and Discussion

The mass spectrum shown in Figure 1 was obtained via the laser ablation of a tantalum rod in a pulsed expansion containing ∼0.8% acetylene seeded in argon. The spectrum shows a clear peak for Ta^+^(C_2_H_2_), which is then followed by a series of masses corresponding to dehydrogenated Ta^+^(C_2_H_2_)_*n*_–H_2_ ions. Additionally, there is a second series, with a lower intensity, corresponding to the argon-tagged species of these ions, which lie 40 amu higher. These observations demonstrate that tantalum promotes the efficient dehydrogenation of acetylene in the multiligand complexes, resulting in the loss of two hydrogen atoms, rather than undergoing simple acetylene addition.

**Figure 1:**
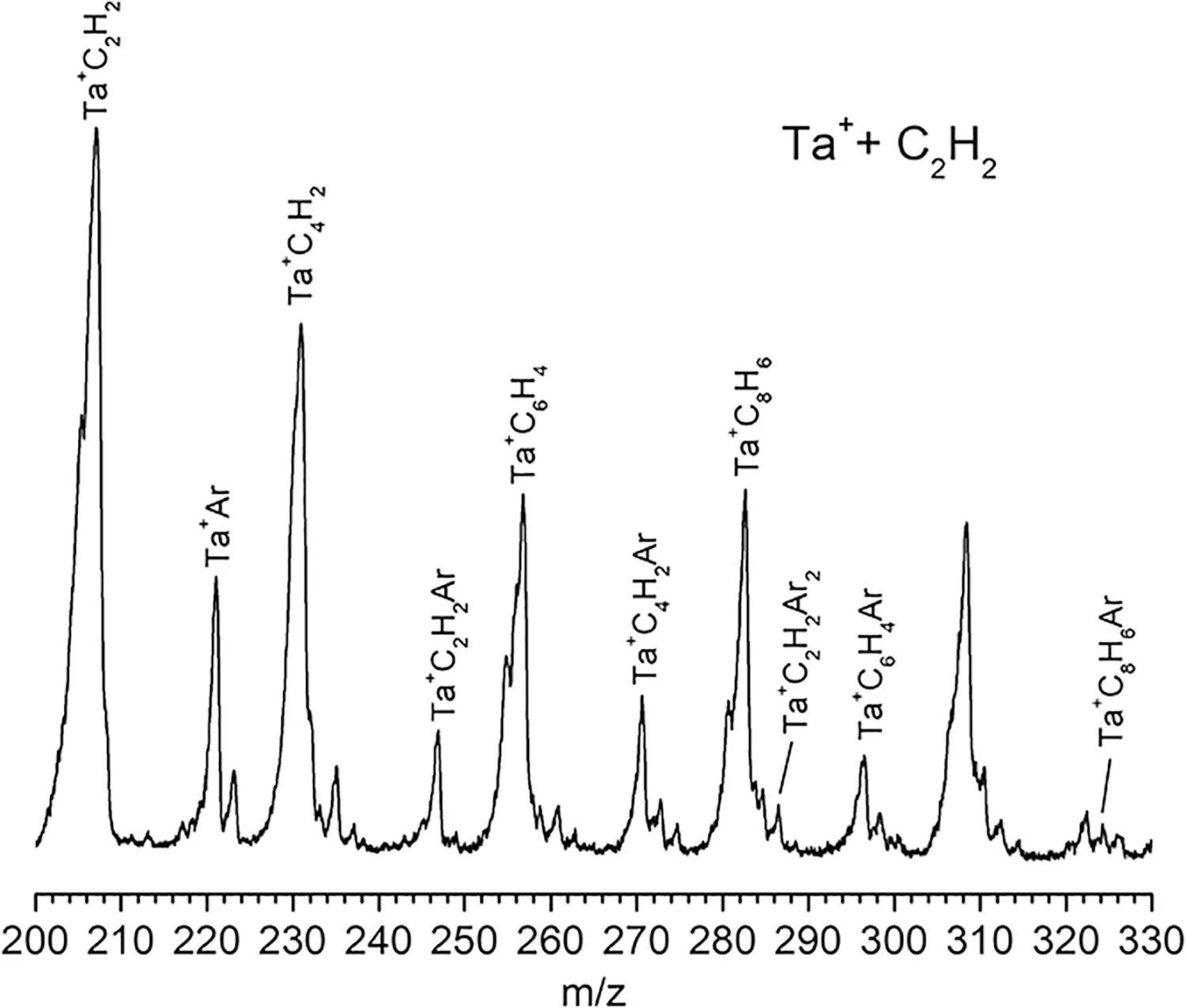
Mass spectrum obtained for tantalum hydrocarbon complexes generated in a supersonic expansion containing 0.8% acetylene diluted in argon. Ion series corresponding to Ta^+^(C_2_H_2_) and Ta^+^(C_2_H_2_)_*n*_–H_2_, as well as the Ar-tagged analogs, are observed.

The infrared photodissociation spectra in the C–H stretching region obtained for Ta^+^(C_2_H_2_)­Ar_2_ and those for the Ta^+^(C_2_H_2_)_*n*_Ar–H_2_ complexes for *n* = 2–4 are shown in Figure 2. The top spectrum was measured by tagging with two argons, whereas the lower three were measured via tagging with one argon. This was done because the binding energy of the argon is presumably too high for the photofragmentation of Ta^+^(C_2_H_2_)­Ar. Although the computed bond energy (6.5 kcal/mol; 2275 cm^–1^) is below the energy range of the experiment, no fragmentation could be measured for this ion. Addition of the second argon made photofragmentation possible. The spectra for the larger dehydrogenated species deviate significantly from that of Ta^+^(C_2_H_2_), and then stay relatively consistent, suggesting a common structural motif in the larger clusters. This is also consistent with the observation that dehydrogenation starts to occur from the coordination of the second ligand, as seen in Figure 1. The antisymmetric and symmetric stretching frequencies of the isolated acetylene ligand are shown by the dashed red lines at 3289 and 3374 cm^–1^, respectively.^80^ The vibrational bands observed for all the complexes are shifted to lower frequencies (i.e., “red-shifted”) relative to these vibrations, indicating significant perturbation of the acetylene bonding upon coordination to the tantalum ion. Such red shifts are consistent with predictions from the well-known Dewar–Chatt–Duncanson model.^81–83^ According to this model, the cation polarizes the acetylene ligands by withdrawing electron density toward the metal center, thereby decreasing the electron density within the acetylene framework. This charge-transfer interaction weakens the C–H bonds, resulting in a shift of their vibrational frequencies to lower frequencies. Bonding in acetylene is further weakened through π back-donation from occupied tantalum d orbitals into the acetylene π* antibonding orbitals.

**Figure 2:**
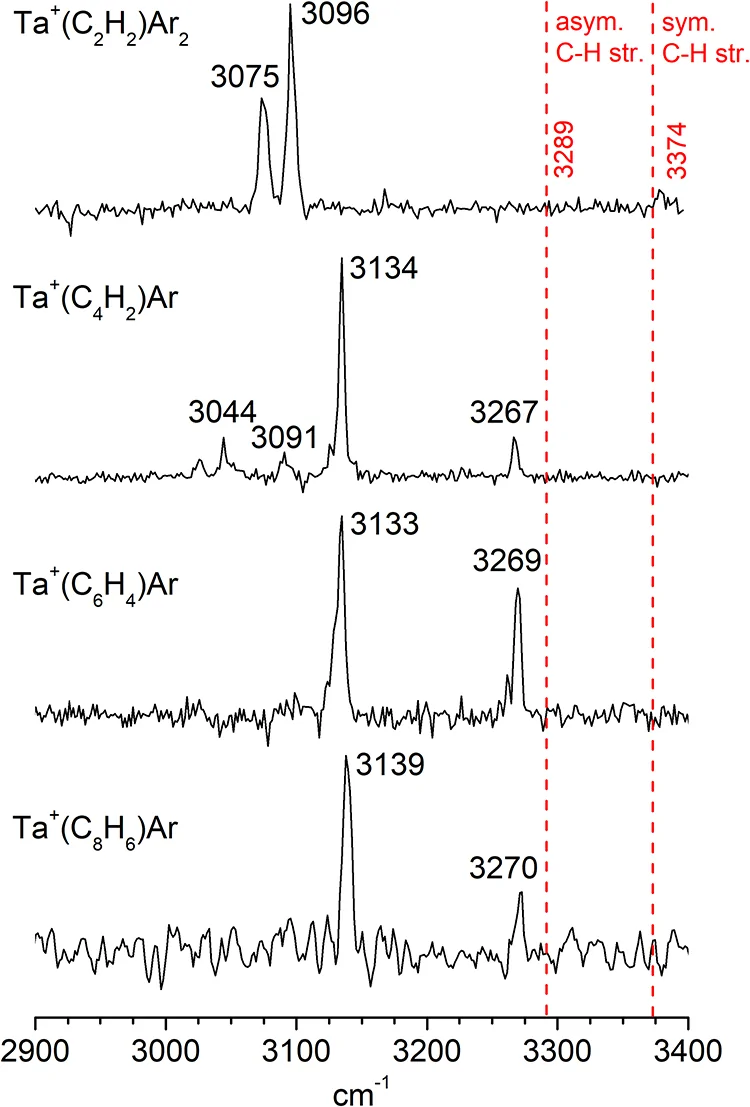
Infrared photodissociation spectra of Ta^+^(C_2_H_2_)­Ar_2_ and Ta^+^(C_2_H_2_)_*n*_Ar–H_2_ complexes for *n* = 2–4 measured in the C–H stretching region. The dashed vertical lines indicate the symmetric and antisymmetric C–H stretching frequencies of free acetylene at 3374 and 3289 cm^–1^, respectively.^80^

DFT calculations were performed on the different tantalum-ion acetylene complexes to identify their structures. All cluster sizes were investigated in the singlet, triplet, and quintet spin states for both tagged and tag-free complexes. The full details of the computational work are in the Supporting Information, where it is shown that the argon tag has little to no effect on the calculated spectra of the smallest two cluster sizes. Therefore, only the computed tag-free spectra are given for *n* = 3 and 4. The lowest energy and/or best matching structures and spin states are discussed here, while the information on the higher-energy isomers and spin states are provided in the Supporting Information.

Figure 3 shows the optimized structures for the low-energy isomers found for Ta^+^(C_2_H_2_) and Ta^+^(C_2_H_2_)_*n*_–H_2_ complexes for *n* = 2–4. These are labeled by the number of acetylene ligands and their corresponding isomer letter, in energetic order, with isomer “*n*a” being the most stable.

**Figure 3:**
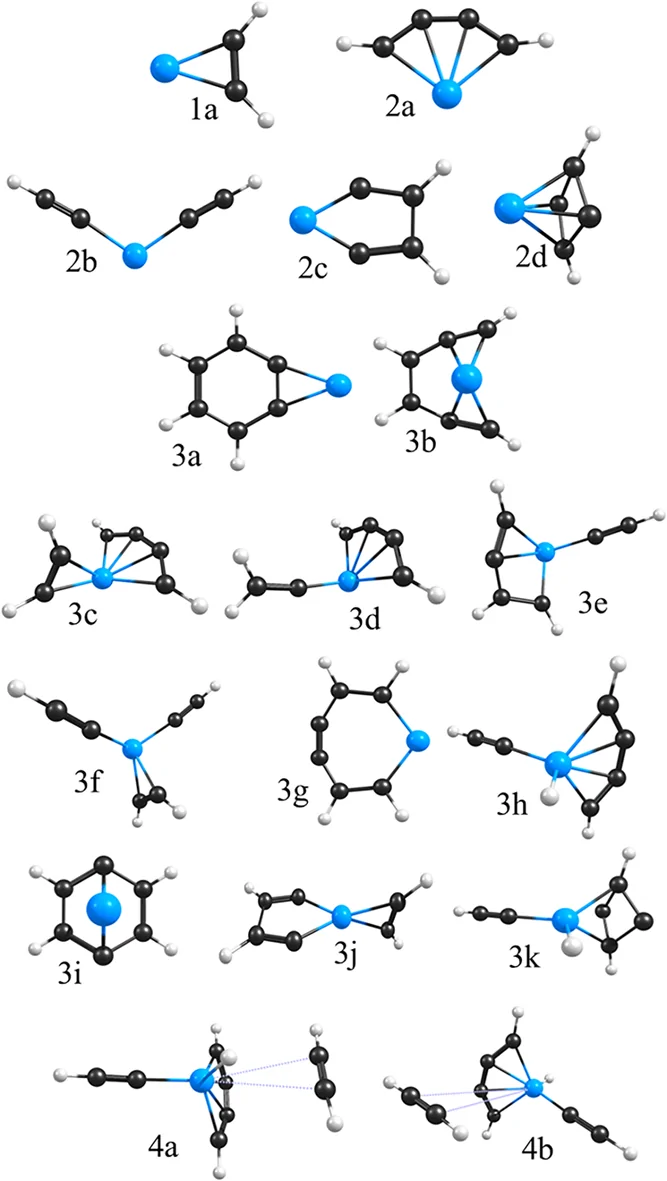
Optimized geometries for the low-energy Ta^+^(C_2_H_2_) and Ta^+^(C_2_H_2_)*_n_*–H_2_ isomers for *n* = 2–4.

The **1a** complex exhibits a well-defined η^2^ cation–π coordination with *C*_2*v*_ symmetry. The metal is symmetrically positioned over the midpoint of the C–C bond with equal Ta–C distances (1.97 Å). The C–C bond length in the complex (1.35 Å) is elongated relative to free acetylene (1.20 Å) and comparable to that of the double bond in ethylene (1.34 Å). The H–C–C angle is reduced to 136°, indicating that the hydrogen atoms bend away from the tantalum ion. This metallacycle structure is similar to those found previously for vanadium-acetylene^64^ and titanium-acetylene^72^ complexes with a single acetylene. However, it is quite different from those for the other transition metals, where cation-π complexes formed with minimal distortion of acetylene.

In the larger clusters, additional ligands become dehydrogenated and also introduce asymmetry and more complex bonding patterns. For *n* = 2, the predicted isomers include both noncoupled-ligand coordination complexes and ligand-coupled structures. Isomer **2a** corresponds to a partially dehydrogenated metallacycle, with metal–carbon bonds to all carbon atoms. The bonds adjacent to the metal in the ring are slightly shorter (2.14 Å) than those across the ring (2.18 Å). The remaining hydrogens bind on the carbon atoms adjacent to the metal. Isomer **2b** corresponds to two acetylide ligands coordinated symmetrically to the metal center. The bonding angle between the acetylide units is 112° in the most stable triplet spin state. **2c** is also a dehydrogenated metallacycle, but with the hydrogens attached remotely from the metal and metal–carbon bonds only to the carbons adjacent to the metal. **2d** is a dehydrogenated cyclobutadienyl structure, with missing hydrogens on opposite carbons. The number of isomers for *n* = 3 increases rapidly with the growing number of atoms and therefore this cluster size has greater structural diversity than the smaller ions. All of these isomers are dehydrogenated, and most of them exhibit some kind of ligand-coupling reaction. The **3a** structure represents a dehydrogenated benzene complex with the metal centered above one C–C bond in an overall planar configuration. **3b** has a structure with the metal bridging two acetylide units and side-on coordination to an additional acetylene. Isomers **3c**–**3e**, **3h**, and **3j** are different forms of dehydrogenated metallacycles like isomer **2a**, with different carbons dehydrogenated and either acetylene or acetylide moieties opposite this. **3f** has a diacetylide structure like **2b**, but with an additional acetylene coordinated in a cation–π configuration opposite this. **3k** has a dehydrogenated cyclobutadienyl structure like **2d**, but with an additional acetylene coordinated opposite this. **3i** is the only isomer resembling a benzene structure, but with the metal coordinated above the six-membered ring, forming metal carbon bonds to opposite carbons. The number of possible *n* = 4 isomers is intractable. However, we have identified some that have features in common with the smaller systems and have IR spectra that match the experiments. Therefore, isomers **4a**–**4c** resemble isomers **2a** and **3h**, with an outer acetylene ligand bound on different sides.

We investigated how argon tagging affects the spectra by comparing the predicted structures and IR spectra of the tagged and tag-free clusters of *n* = 1 and 2 complexes (see Supporting Information). Argon has little to no effect on the structures and only shifts the frequencies very slightly (<10–20 cm^–1^) in the most stable structures where it is attached to the metal ion. The shift becomes greater when argon is bound to a hydrogen, however, this is energetically unfavorable. Therefore, the predicted spectra and energetics shown below for all the cluster sizes are those for the bare complexes. The relative energies of these different isomers for each cluster size are shown in Table 1, with more details provided in the Supporting Information. The spin-state competition seen across the tantalum acetylene clusters can be described by the electronic structure of the isolated tantalum ion. The metal ion has a ^5^F (6s^1^5d^3^) ground state with nearby ^3^P (6s^2^5d^2^; 3242 cm^–1^), ^3^F (6s^2^5d^2^; 5048 cm^–1^) and ^1^D (6s^2^5d^2^; 15323 cm^–1^) excited states. For several of the tantalum acetylene clusters, singlet and triplet states are energetically competitive, while the quintet states consistently lie much higher in energy. Because density functional theory is known to have limitations in accurately describing spin-state energetics,^84,85^ the relative energies of different spin states here may not be reliable and should be interpreted with caution.

**Table 1:** Energetics of Ta^+^(C_2_H_2_)*_n_* for *n* = 1 and Ta^+^(C_2_H_2_)*_n_*–H_2_ for *n* = 2–4 Complexes from Computational Studies^a^

*n*	2s + 1	isomer	*E* (Hartree)	Δ*E* (kcal/mol)	BDE acet. (kcal/mol)
0	1		–57.003052	+24.1	
0	3		–57.027178	+8.9	
0	5		–57.041383	0.0	
1	1	**1a**	–134.500248	+4.1	103.3
1	3	**1a**	–134.506735	0.0	92.2
1	5	**1a**	–134.447546	+37.1	46.1
2	1	**2a**	–210.693054	+1.3	
2	3	**2a**	–210.69506	0.0	
2	1	**2b**	–210.655709	+24.7	
2	3	**2b**	–210.661137	+21.3	
2	1	**2c**	–210.641421	+33.7	
2	3	**2c**	–210.634917	+37.7	
2	1	**2d**	–210.592836	+64.1	
2	3	**2d**	–210.586653	+68.0	
3	1	**3a**	–288.151204	+4.6	
3	3	**3a**	–288.158473	0.0	
3	1	**3b**	–288.150619	+4.9	
3	3	**3b**	–288.111351	+29.6	
3	1	**3c**	–288.144688	+8.7	
3	3	**3c**	–288.108513	+31.4	
3	1	**3d**	–288.115498	+27.0	
3	3	**3d**	–288.098838	+37.4	
3	1	**3e**	–288.116966	+26.0	
3	3	**3e**	–288.094111	+40.4	
3	1	**3f**	–288.115828	+26.8	
3	3	**3f**	–288.0711	+54.8	
3	1	**3g**	–288.053435	+65.9	
3	3	**3g**	–288.061379	+60.9	
3	1	**3h**	–288.136615	+13.7	
3	3	**3h**	–288.048559	+69.0	
3	1	**3i**	–288.064483	+59.0	
3	3	**3i**	–288.041378	+73.5	
3	1	**3j**	–288.037082	+76.2	
3	3	**3j**	–288.038506	+75.3	
3	1	**3k**	–287.98262	+110.3	
3	3	**3k**	–287.986379	+108.0	
4	1	**4a**	–365.472897	0.0	2.3
4	3	**4a**	–365.469133	+2.4	55.2
4	1	**4b**	–365.472407	+0.3	2.0
4	3	**4b**	–365.385403	+54.9	2.6

a For other isomers, see Supporting Information.

Figure 4 shows the experimental spectrum measured for Ta^+^(C_2_H_2_)­Ar_2_ compared to the spectra predicted for this complex for the singlet and triplet spin states. The experiment has two prominent bands in the C–H stretching region at 3075 and 3096 cm^–1^. These are the acetylene asymmetric and symmetric C–H stretches, which have been shifted to lower frequencies relative to the free molecule by 214 and 278 cm^–1^, respectively, due to the interaction with the tantalum ion. These shifts are similar to those seen previously for the V^+^(C_2_H_2_) complex.^64^ In both systems, the symmetric C–H stretch has significant IR intensity, actually becoming more intense than the antisymmetric stretch, even though the symmetric stretch of isolated acetylene is IR forbidden. This arises from the significant distortion of the acetylene moiety, with the hydrogens bent away from the C–C axis. The triplet state, which is the lowest in energy, is predicted to have bands at 3059 and 3082 cm^–1^, and these are spaced by 23 cm^–1^. This pattern is in good agreement with the experiment. The singlet state yields a similar spectrum (bands at 3057 and 3082 cm^–1^), but is computed to lie +4.1 kcal/mol higher in energy. On the basis of the predicted spectra, the assignment of the spin state is not clear. However, based on the computed energetics, the triplet state is favored and therefore we assign this to the Ta^+^(C_2_H_2_)­Ar_2_ spectrum. Bakker, Armentrout, and co-workers measured an infrared spectrum for this same ion produced by the dehydrogenation of ethylene by tantalum ions.^66^ Based on infrared patterns and theory, they also concluded that the ground state is a triplet. They observed two bands at low frequency (682 and 1026 cm^–1^) in addition to the C–H stretch. However, because of the lower resolution of their experiment and the multiphoton laser conditions employed, they detected only a single broad band in the C–H stretching region centered at 3070 cm^–1^. This is lower than either of our bands, but band broadening and a red shift of vibrational frequencies is not uncommon when multiphoton photodissociation is employed.^86^

**Figure 4:**
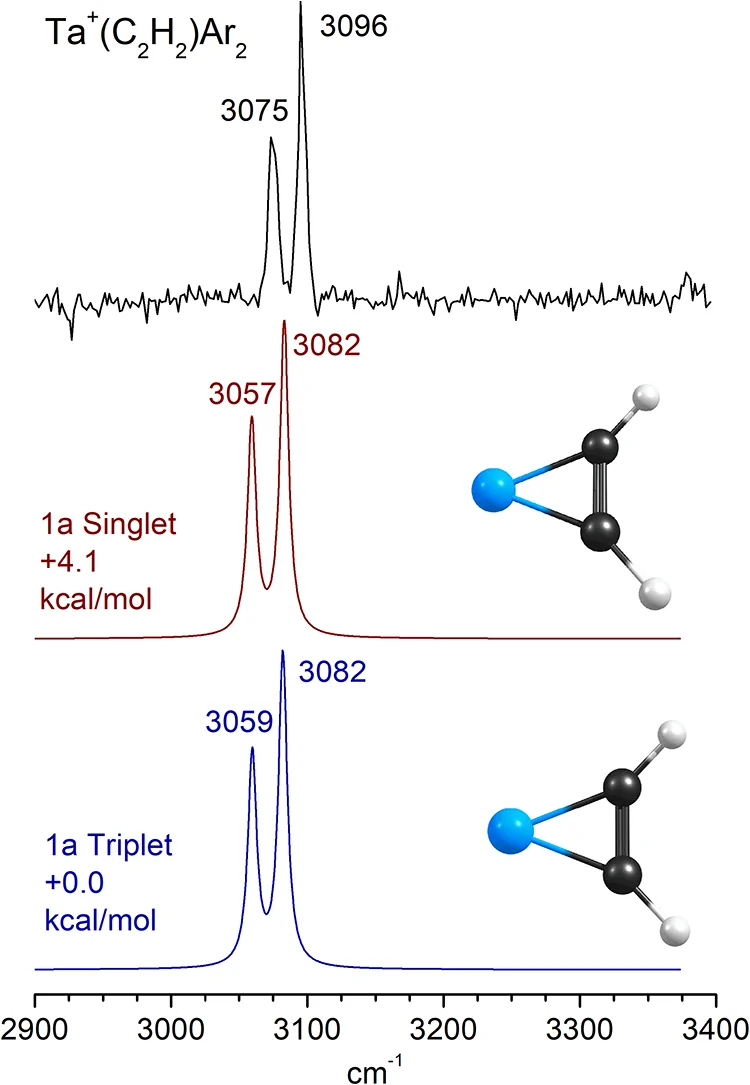
Experimental infrared photodissociation spectrum of Ta^+^(C_2_H_2_)­Ar_2_ in the C–H stretching region. The experimental spectrum is compared with calculated spectra for the low-lying singlet, and triplet isomers. Relative energies are relative to the lowest energy isomer and include zero-point energy corrections. The predicted spectra are shown for the tag-free ions because the argon atom has only a small effect on the vibrational frequencies (see Supporting Information).

Figure 5 shows the infrared photodissociation spectra measured for Ta^+^(C_4_H_2_)Ar compared with the spectra predicted by theory. As mentioned earlier, the effect of tagging with argon was investigated by theory and shown to have very little effect on the spectra. Therefore, the spectra predicted for tag-free complexes are shown. The experimental spectrum has two prominent bands at 3134 and 3267 cm^–1^ along with minor bands at 3044 and 3091 cm^–1^. Four different isomers were examined, while only the two best matches are shown here; the others are shown in the Supporting Information. The most intense band at 3134 cm^–1^ likely corresponds to the C–H stretch of the most energetically favored isomer **2a**. The slightly higher energy band likely corresponds to the C–H stretch of acetylide ligands, which is the second most energetically favored isomer **2b**. Here only the triplet spin states are given because they are energetically favored, while the singlet states have similar predicted spectra. For the two weaker lower energy bands at 3044 and 3091 cm^–1^ there were no good matches with theory. These could conceivably be overtones of lower frequency bending modes. From this experimental spectrum, we therefore assign the Ta^+^(C_4_H_2_) complex to a mixture of the triplet dehydrogenated metallacycle isomer **2a** and the triplet bis-acetylide isomer **2b**, which lies +21.3 kcal/mol higher in energy. Because the IR intensities of these bands are comparable, isomer **2a** makes the dominant contribution. Thus, ligand coupling is first observed for *n* = 2, consistent with the clear changes in the spectral features shown in Figure 2. In addition to the C–H stretching region, we also attempted to measure the C–C stretching region, where bands are predicted just below 2000 cm^–1^. However, no photofragmentation was observed in this region, likely because the argon binding energy exceeds the photon energy available for this complex at these frequencies. This makes sense because the computed binding energy for the argon in the **2a** structure (with argon attached to the Ta^+^) is 6.7 kcal/mol (2345 cm^–1^).

**Figure 5:**
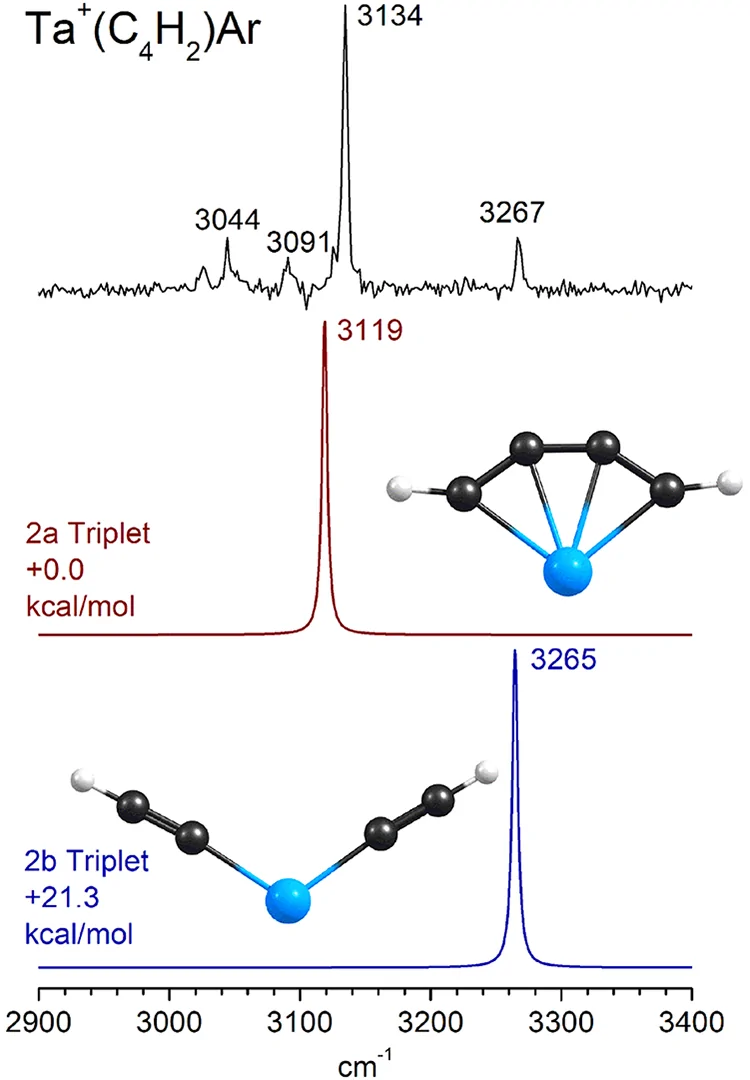
Experimental infrared photodissociation spectrum of Ta^+^(C_4_H_2_)Ar in the C–H stretching region. The experimental spectrum is compared with calculated spectra of the two best matching isomers and spin states; see Supporting Information for the spectra of other isomers.

The observation of two isomers suggests that the kinetics of the reactions are playing a role in product formation. We therefore considered the reaction coordinate for the *n* = 2 system. Figure 6 shows the relative energies and transition states for isomers **2a**, **2b**, and **2c**. Isomer **2a** in its triplet spin state is the zero on this scale. The reaction which forms this from Ta^+^ and two acetylenes is predicted by theory to be highly exothermic (see Supporting Information), e.g.

$${\mathrm{Ta}}^{+}+2C_{2}H_{2}\to {\mathrm{Ta}}^{+}\left(C_{4}H_{2}\right)\left(2a\right)+H_{2},\Delta H=-104.3\;\mathrm{kcal}/\mathrm{mol}$$

**Figure 6:**
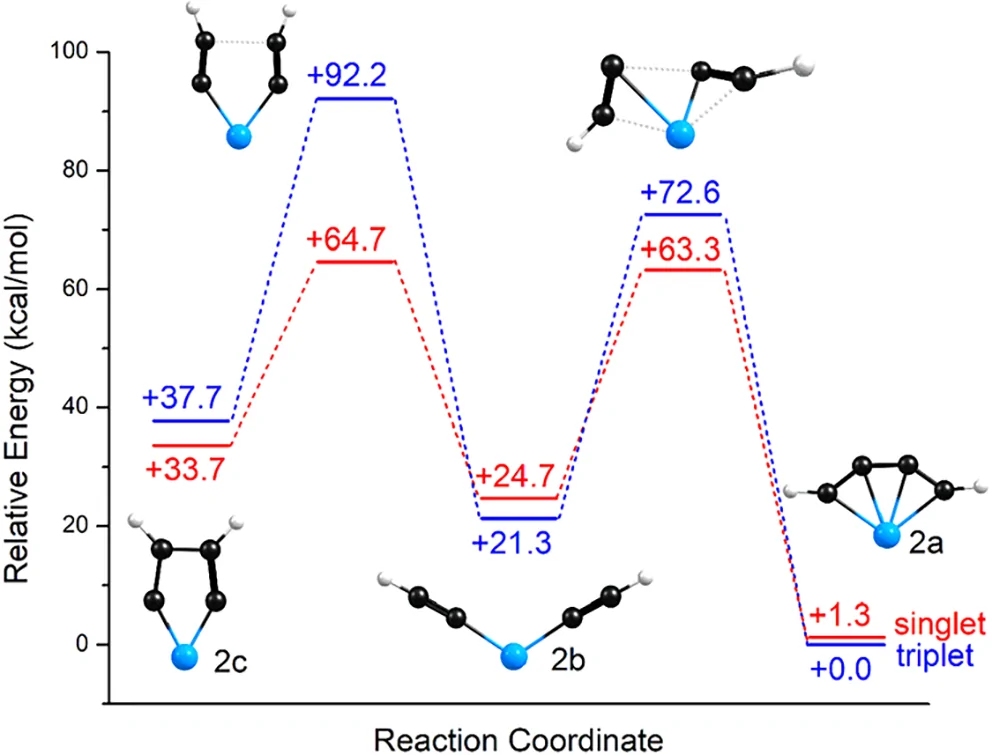
Computed reaction coordinate for isomers **2a**, **2b**, and **2c**.

Therefore, each of these isomers is energetically possible from the initial reaction. However, once formed, each of these isomers has a significant activation energy toward rearranging to the other structures. According to our spectra, both **2a** and **2b** form and **2b** is kinetically trapped against rearrangement to **2a** even though **2a** is more stable. **2c** is apparently not formed in the reaction, because it would also be trapped against rearrangement if it were.

Figure 7 shows the experimental spectrum for Ta^+^(C_6_H_4_)Ar in both the C–C and C–H stretching regions, compared with the spectra predicted for the two best matching isomers for Ta^+^(C_6_H_4_). The energies displayed are referenced to the lowest-energy structure (**3a**, triplet), which corresponds to a dehydrogenated benzene metallacycle complex, with the metal bridging one C–C bond. A total of 11 different isomers were investigated, most of which were inspired by the isomers **2a** and **2b**, due to the similarity of the experimental bands in the C–H stretching region. Isomer **3h** combines both the dehydrogenated metallacycle and acetylide groups from isomers **2a** and **2b**, respectively, along with an additional hydride group. Isomer **3k** contains the same acetylide and hydride groups but has the dehydrogenated square structure from isomer **2d** instead of the metallacycle and is therefore +94.3 kcal/mol higher in energy. The Ta^+^(C_6_H_4_)Ar experimental spectrum has four significant peaks at 1881, 1981, 3133, and 3269 cm^–1^. The latter two are similar in frequency to the spectrum given in Figure 5, indicating a similar structure. Both isomers singlet **3h** and triplet **3k** show good agreement with the experimental spectrum, especially with the 1981, 3133, and 3269 cm^–1^ bands. The 1881 cm^–1^ seems to agree a little bit less with theory, with a shift of 21 and 59 cm^–1^ for isomers **3h** and **3k**, respectively. Due to the larger frequency shift and the higher energy of isomer **3k**, the Ta^+^(C_6_H_4_) complex is assigned to isomer **3h** in the singlet spin state. In order of increasing frequency, the experimental bands correspond to the Ta–H stretch, the acetylide C–C stretch, the dehydrogenated metallacycle asymmetric C–H stretch and the acetylide C–H stretch.

**Figure 7:**
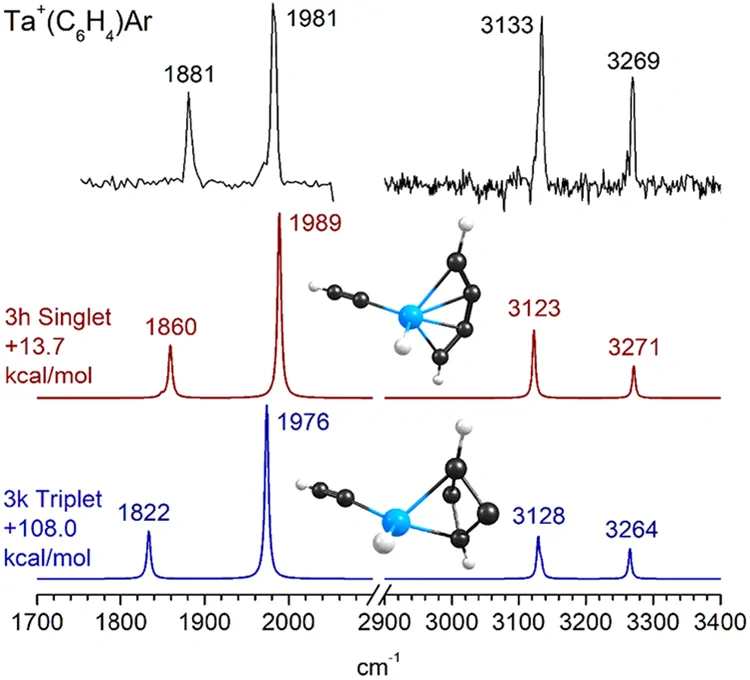
Experimental infrared photodissociation spectrum of Ta^+^(C_6_H_4_)Ar in both the C–H and C–C stretching regions. The experimental spectrum is compared with calculated spectra of the two best matching isomers and spin states; see Supporting Information for the spectra predicted for other isomers.

It is clear from this discussion that the most stable isomer for the *n* = 3 complex is not formed and that the isomer that does form is less stable, all assuming that the DFT energies determined are reliable. Kinetics must again be playing a role in which isomers are formed. The reaction forming these structures directly from acetylene is predicted by theory to be highly exothermic (see Supporting Information)­

$${\mathrm{Ta}}^{+}+3C_{2}H_{2}\to {\mathrm{Ta}}^{+}\left(C_{6}H_{4}\right)\left(3a\right)+H_{2},\Delta H=-186.4\;\mathrm{kcal}/\mathrm{mol}$$

Therefore, several *n* = 3 isomers are energetically possible, but apparently only isomer **3h** forms. We have not computed the reaction coordinate for the *n* = 3 system, but it is reasonable to conclude that once **3h** forms there are barriers that prevent its rearrangement to more stable structures. Likewise it makes sense that isomer **3h** would form from the reaction of the most abundant isomer **2a** with an additional acetylene molecule. The **3h** structure requires essentially no rearrangement from the **2a** structure. A similar argument could be made for isomer **3c**, but it is not detected. Apparently isomer **2a** is reactive enough to insert into acetylene rather than to just add an intact molecule.

Figure 8 shows the experimental C–H stretching spectrum of Ta^+^(C_8_H_6_)­Ar, compared with the spectrum predicted for the best-matching isomer, **4b** in the singlet state. This structure is only +0.3 kcal/mol higher in energy than the lowest-energy isomer. Based on the strong similarity to the *n* = 3 spectrum, the *n* = 4 complex was expected to contain an externally bound, intact acetylene ligand. Accordingly, the three isomers investigated were based on the structure of isomer **3h**, with the external acetylene ligand bound at three different sites, as shown in Figure 3. For one of these structures, **4c**, the calculations failed to converge despite numerous attempts, suggesting that this is not a favorable binding site for the acetylene ligand. Although the experimental spectrum is somewhat noisy, it exhibits two prominent peaks at 3139 and 3270 cm^–1^, which closely resemble those observed in the C–H stretching region of Figure 7. These features are in good agreement with the calculated spectrum of singlet isomer **4b**, where the asymmetric C–H stretch of the intact acetylene ligand overlaps with the C–H stretch of the acetylide, resulting in a single predicted band at 3267 cm^–1^. We therefore assign the Ta^+^(C_8_H_6_) complex to the singlet isomer **4b**. The observed bands at 3139 and 3270 cm^–1^ correspond to the dehydrogenated metallacycle C–H stretch and the combined acetylide C–H stretch/external acetylene asymmetric C–H stretch, respectively. Three acetylene ligands completing the inner coordination sphere is consistent with earlier studied metal-acetylene complexes in our lab, with metals like Cu^+^, Au^+^, Zn^+^, Pt^+^, and Co^+^. For the *n* = 4 spectrum, the C–C stretching region was also attempted. However, due to low parent ion signal and lower laser power in this region, no spectrum was obtained.

**Figure 8:**
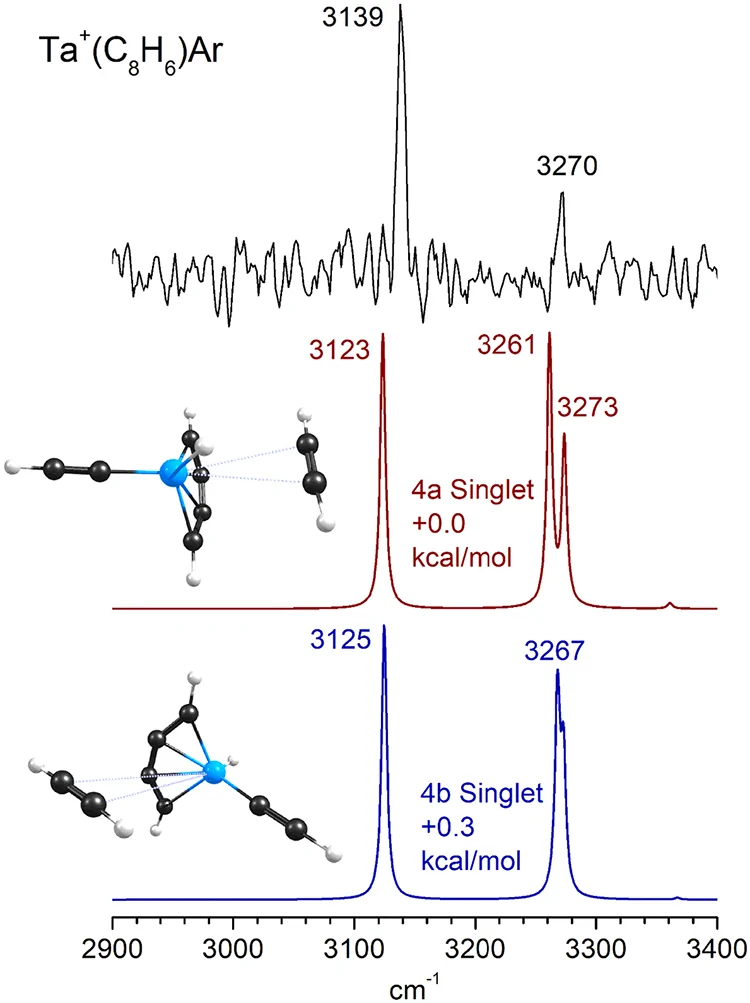
Experimental Infrared photodissociation spectrum of Ta^+^(C_6_H_4_)Ar in the C–H stretching region. The experimental spectrum is compared with calculated spectrum of the best matching isomer **4b**.

These measurements clearly show the strong tendency for tantalum ions to dehydrogenate acetylene. This is consistent with previous mass spectrometry data, but the IR spectra show what structures are actually formed in these reactions. The only previous work with the same kind of information is that by Bakker, Armentrout, and co-workers on the reactions of Ta^+^ with ethylene,^66^ where dehydrogenation produced the same kind of monoacetylene structure found here. However, structures like that for the Ta^+^(C_4_H_2_) metallacycle were suggested previously for uranium ions in the fragmentation of metal-benzene or metal-cyclooctatetraene complexes.^87,88^ The formation of the HC_4_H ligand structure suggests that chemistry like this could conceivably lead to the formation of polyynes. However, the structures found here have strong tantalum–carbon bonds and it is unlikely that this ligand would be released from the metal center. Additionally, the larger clusters do not appear to form structures that extend the carbon chain. In both the metallacycle structures and the metal-acetylide structures, a common theme is that tantalum is forming stable metal–carbon bonds. It is perhaps worth noting that under different laser ablation conditions the group 4 and 5 metals continue to dehydrogenate small hydrocarbons to eventually form the well-known metal-carbide cages and nanocrystal structures.^89–92^

As noted earlier, the enhanced reactivity of tantalum compared to other transition metals has been documented previously in gas phase ion chemistry.^10,11,18,31,41–43,60,66^ However, it has also been described for *neutral* tantalum in conventional inorganic chemistry by Schrock and co-workers, among others.^93–95^ In that chemistry, dehydrogenation and metallacycle formation is also important and tantalum is recognized to be reactive because it is stable in high oxidation states. In tantalum ions, the ground state is a high-spin quintet, but spin-paired triplet and singlet states lie at relatively low energies. Spin-pairing makes it possible to reduce ligand-electron repulsion, achieving orbital interactions not unlike those in the high oxidation states of the neutral, perhaps explaining why the ion is also reactive in a similar way.

## Conclusion

Tantalum cation-acetylene complexes containing up to four acetylene ligands were produced by laser ablation in a supersonic molecular beam and investigated using mass-selected infrared photodissociation spectroscopy. Coordination of the second through fourth acetylene ligands results in efficient dehydrogenation, yielding complexes of the form Ta^+^(C_2_H_2_) and Ta^+^(C_2_H_2_)_*n*_–H_2_ (*n* = 2–4). Photodissociation spectroscopy was performed with argon tagging, which was shown to have little or no effect on the band positions in the infrared spectra. The experimental spectra were assigned by comparison with structures predicted by DFT at the B3LYP level, based on their calculated infrared spectra and relative energies. All complexes were characterized in the C–H stretching region, while the *n* = 3 complex was additionally studied in the C–C stretching region. The Ta^+^(C_2_H_2_) complex is assigned to a simple metallacyclopropene structure. In contrast, ligand coupling is observed from the second acetylene ligand onward. The Ta^+^(C_4_H_2_) complex is assigned to a mixture of a dehydrogenated metallacycle and a bis-acetylide structure, which are both structures not known previously for these systems. The Ta^+^(C_6_H_4_) spectrum further supports this assignment, corresponding to a structure containing a dehydrogenated metallacycle, an acetylide ligand, and a metal hydride. Finally, the Ta^+^(C_8_H_6_) complex demonstrates that the tantalum coordination sphere is complete after coordination of three acetylene ligands, with the fourth acetylene remaining intact and binding externally. These results demonstrate that tantalum is exceptionally reactive toward acetylene, promoting efficient dehydrogenation and extensive ligand coupling, with the monoacetylene complex being the only exception. This reactivity is consistent with previous studies in which V^+^, Zn^+^, and Ti^+^ were found to induce ligand coupling, whereas Cu^+^, Au^+^, Ag^+^, Fe^+^, and Co^+^ predominantly formed simple acetylene adducts. While both acetylene coupling and hydrocarbon dehydrogenation are individually well established, tantalum is, to our knowledge, the first metal ion to exhibit both processes simultaneously within a single gas-phase system. The unique organometallic structures observed in this work provide new insight into how tantalum activates hydrocarbons and may help improve our understanding of the elementary steps involved in catalysis.

## Supplementary Material



## Notes

Details of the DFT computations done, including the structures and energetics for each of the ions considered (PDF)
