# Supplementary material for: Reactions of Ta+ with Acetylene Studied with Infrared Photodissociation Spectroscopy and Theory
Source: J Phys Chem A. 2026 Oct 21;130(44):8828–37. doi: 10.1021/acs.jpca.6c05456 (PMC13746808; doi:10.1021/acs.jpca.6c05456)
Supplement: Supplementary file 1 [file jp6c05456_si_001.pdf]

Supporting Information

Reactions of Ta^+ with Acetylene Studied with Infrared Photodissociation Spectroscopy and Theory

Jens van der Hulst,[1,2] Richard B. Odonkor,[1] Nathan J. Dynak,[1] Michael A. Duncan*[1]

[1]Department of Chemistry, University of Georgia, Athens, GA 30602, United States
[2]Faculty of Science, Radboud University, Nijmegen, The Netherlands

*Email: maduncan@uga.edu

Table of Contents

Computational details S3

C_2H_2
Table S1: energy S4
Figures S1: structural parameters & unscaled vibrational frequencies S5

Ta^+
Table S2: relative energies S6

$Ta^+C_2H_2$, $Ta^+C_2H_2Ar$, & $Ta^+C_2H_2Ar_2$
Tables S3 – S5: calculated energies S7 – S9
Figures S2 – S19: structural parameters & unscaled vibrational frequencies S11 – S27
Figures S20 – S23: simulated spectra S28 – S31

$Ta^+C_4H_2$ & $Ta^+C_4H_2Ar$
Tables S6 – S8: calculated energies S32 – S34
Figures S24 – S63: structural parameters & unscaled vibrational frequencies S35 – S74
Figures S64 – S78: simulated spectra S75 – S89

$Ta^+C_6H_4$
Tables S9 – S10: calculated energies S89 – S91
Figure S75: predicted minimum energy structures S92
Tables S11 – S43: cartesian coordinates & unscaled vibrational frequencies S93 – S125
Figures S80 – S91: simulated spectra S126 – S137

$Ta^+C_8H_6$
Table S44: calculated energies S138
Figure S92: predicted minimum energy structures S139
Tables S45 – S49: cartesian coordinates & unscaled vibrational frequencies S140 – S144
Figures S93 – S95: simulated spectra S145 – S147

Computational details;

All calculations were performed using density functional theory (DFT) with the B3LYP functional. The fully relativistic ECP60MDF Stuttgart/Cologne pseudopotential and corresponding correlation-consistent triple-ζ basis set (cc-pVTZ-pp) were used for tantalum. The all-electron cc-pVDZ and cc-pVTZ basis sets were used for hydrogen and carbon, respectively. Optimizations were performed with the *nosymm* option, and wavefunction stability was verified using the *stable=opt* keyword. The thresholds for energy and structure optimizations were set to "tight," and all calculations used the default "ultrafine" integration grid. All optimized structures were confirmed to be true minima, with no imaginary vibrational frequencies, except for the transition states. All electronic energies are zero-point vibrational energy (ZPVE) corrected. Cartesian coordinates presented in this document are listed with atomic number (Z) and x, y, and z Cartesian coordinates. Listed vibrational frequencies (cm^{-1}) are unscaled with intensities (km/mol). The scaling factor used (0.964) was determined by comparing the computed CH-stretch vibrational frequency of acetylene, obtained at the same level of theory, with its known experimental value.

Table S1. C_2H_2 electronic energy calculated at the B3LYP/cc-pVDZ [H] & cc-pVTZ [C] level of theory using Gaussian 16.

2s + 1	E(hartree)	Relative E (kcal/mol)
1	-77.332652	+0.0

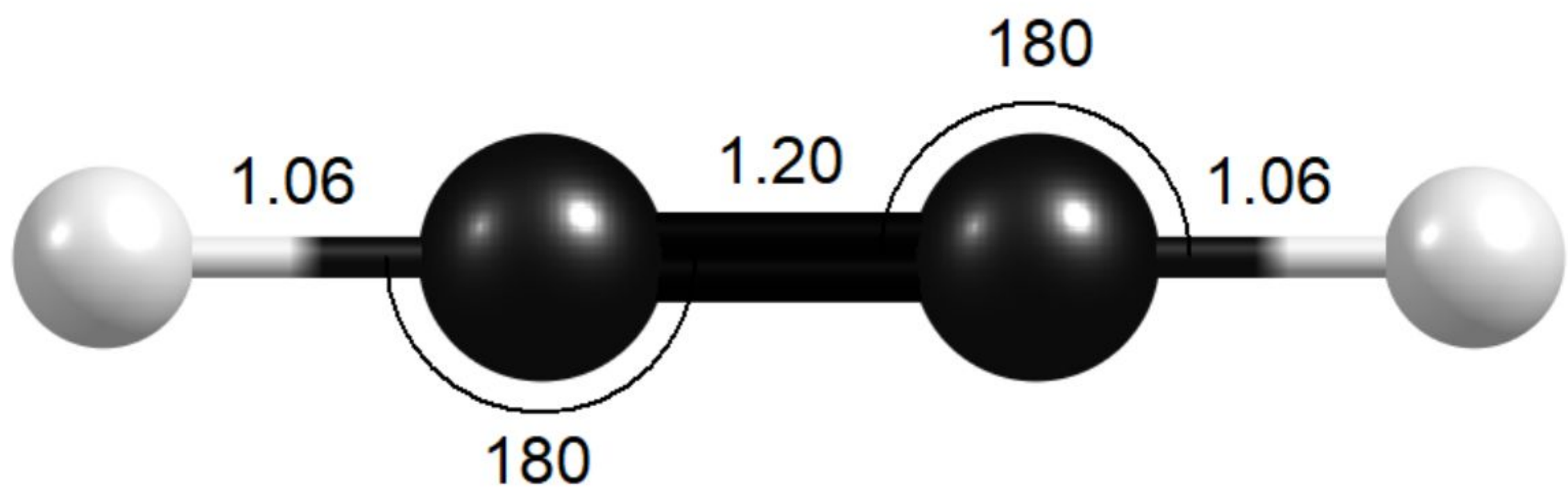

Figure S1. The optimized geometry of C_2H_2 followed by its predicted frequencies (cm^{-1}) and IR intensities (km/mol).

Frequency (cm^{-1})	Intensity (km/mol)
637.24	0.0000
637.24	0.0000
766.20	102.0768
766.20	102.0768
2072.61	0.0000
3404.08	93.2556
3505.54	0.0000

Table S2. Ta^+ electronic energy calculated at the B3LYP/cc-pVTZ(-pp) level with Stuttgardt/Koeln pseudopotential.

2s + 1	E(hartree)	Relative E (kcal/mol)
1	-57.003052	+24.1
3	-57.027178	+8.9
5	-57.041383	+0.0

Table S3. $Ta^+C_2H_2$ electronic energy calculated at the B3LYP/cc-pVTZ(-pp) level with Stuttgardt/Koeln pseudopotential.

2s + 1	E(hartree)	Relative E (kcal/mol)
1	-134.500248	+4.1
3	-134.506735	+0.0
5	-134.447546	+37.1

Table S4. $Ta^+C_2H_2Ar$ electronic energy calculated at the B3LYP/cc-pVTZ(-pp) level with Stuttgardt/Koeln pseudopotential.

Isomer	2s + 1	E (hartree)	Relative E (kcal/mol)	Ar BE (kcal/mol)
1aI	1	-662.07011	+4.0	6.6
1aII	1	-662.060088	+10.3	0.3
1aI	3	-662.07643	+0.0	6.5
1aII	3	-662.066571	+6.2	0.3
1aI	5	-662.019875	+35.5	8.1
1aII	5	-662.007732	+43.1	0.5

Table S5. $Ta^+C_2H_2Ar_2$ electronic energy calculated at the B3LYP/cc-pVTZ(-pp) level with Stuttgardt/Koeln pseudopotential.

Isomer	2s + 1	E (hartree)	Relative E (kcal/mol)	Ar BE (kcal/mol)
1aI	1	-1189.634054	+3.8	2.9
1aII	1	-1189.629865	+6.5	0.2
1aIII	1	-1189.619942	+12.7	0.3
1aI	3	-1189.640181	+0.0	2.7
1aII	3	-1189.636181	+2.5	0.2
1aIII	3	-1189.626409	+8.6	0.3
1aI	5	-1189.583243	+35.7	2.5
1aII	5	-1189.579915	+37.8	0.4
1aIII	5	-1189.567944	+45.3	0.5

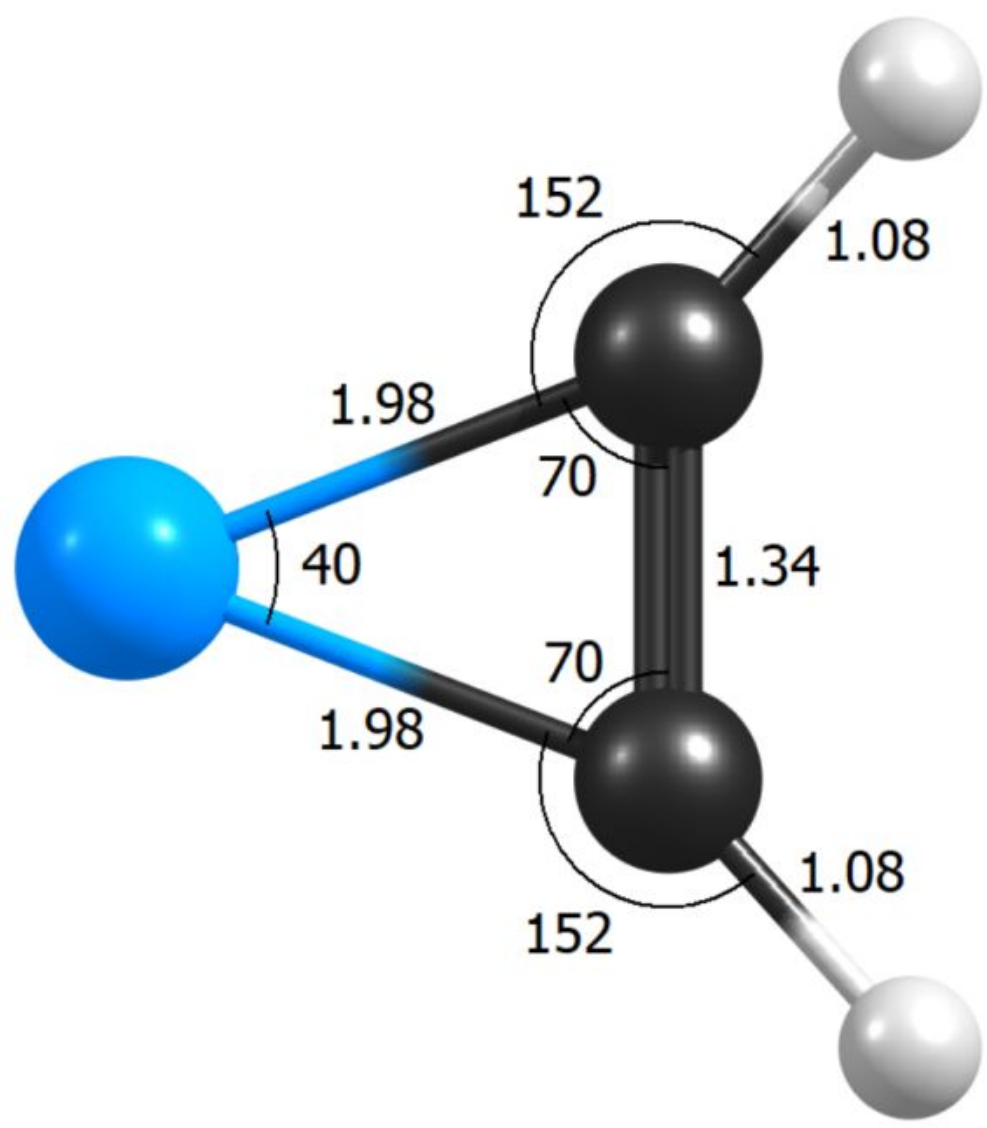

Figure S2. The optimized geometry of isomer singlet $Ta^{+}C_2H_2$ followed by its predicted frequencies (cm^{-1}) and IR intensities (km/mol).

Frequency (cm^{-1})	Intensity (km/mol)
603.35	0.2255
678.69	18.3501
747.12	83.7845
832.48	0.0440
946.10	0.0000
1080.00	75.3100
1427.81	0.9770
3173.60	29.9369
3198.28	43.8000

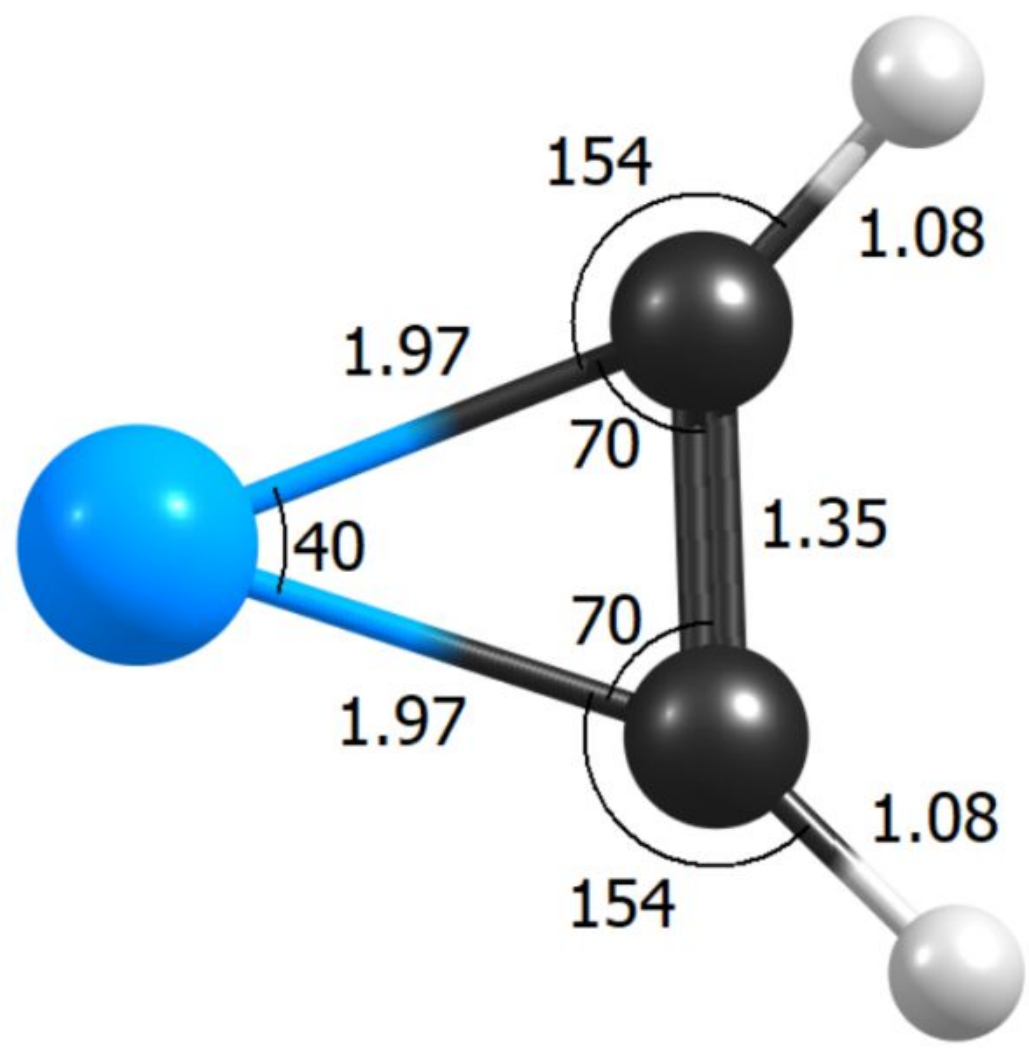

Figure S3. The optimized geometry of isomer triplet $Ta^+C_2H_2$ followed by its predicted frequencies (cm^{-1}) and IR intensities (km/mol).

Frequency (cm^{-1})	Intensity (km/mol)
605.75	3.3370
682.99	22.8052
740.11	83.6937
823.46	1.1510
946.57	0.0000
1085.10	76.0058
1410.33	0.2940
3173.99	29.8014
3197.18	43.8257

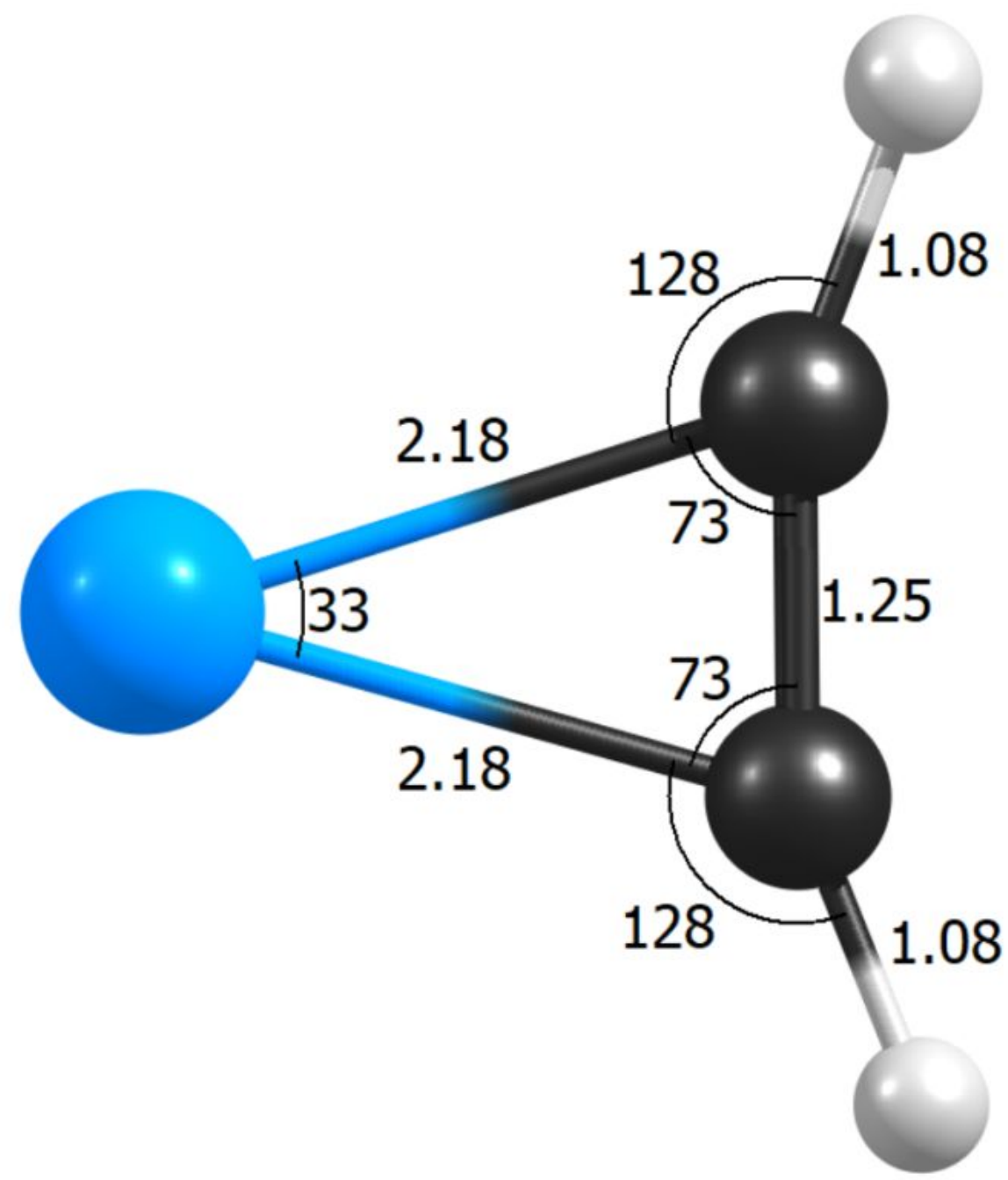

Figure S4. The optimized geometry of isomer quintet $Ta^{+}C_2H_2$ followed by its predicted frequencies (cm^{-1}) and IR intensities (km/mol).

Frequency (cm^{-1})	Intensity (km/mol)
435.61	4.5232
438.62	2.3316
674.38	0.0000
683.57	64.6803
697.87	82.1323
774.59	0.1974
1773.99	13.3668
3202.97	165.7308
3274.72	84.7251

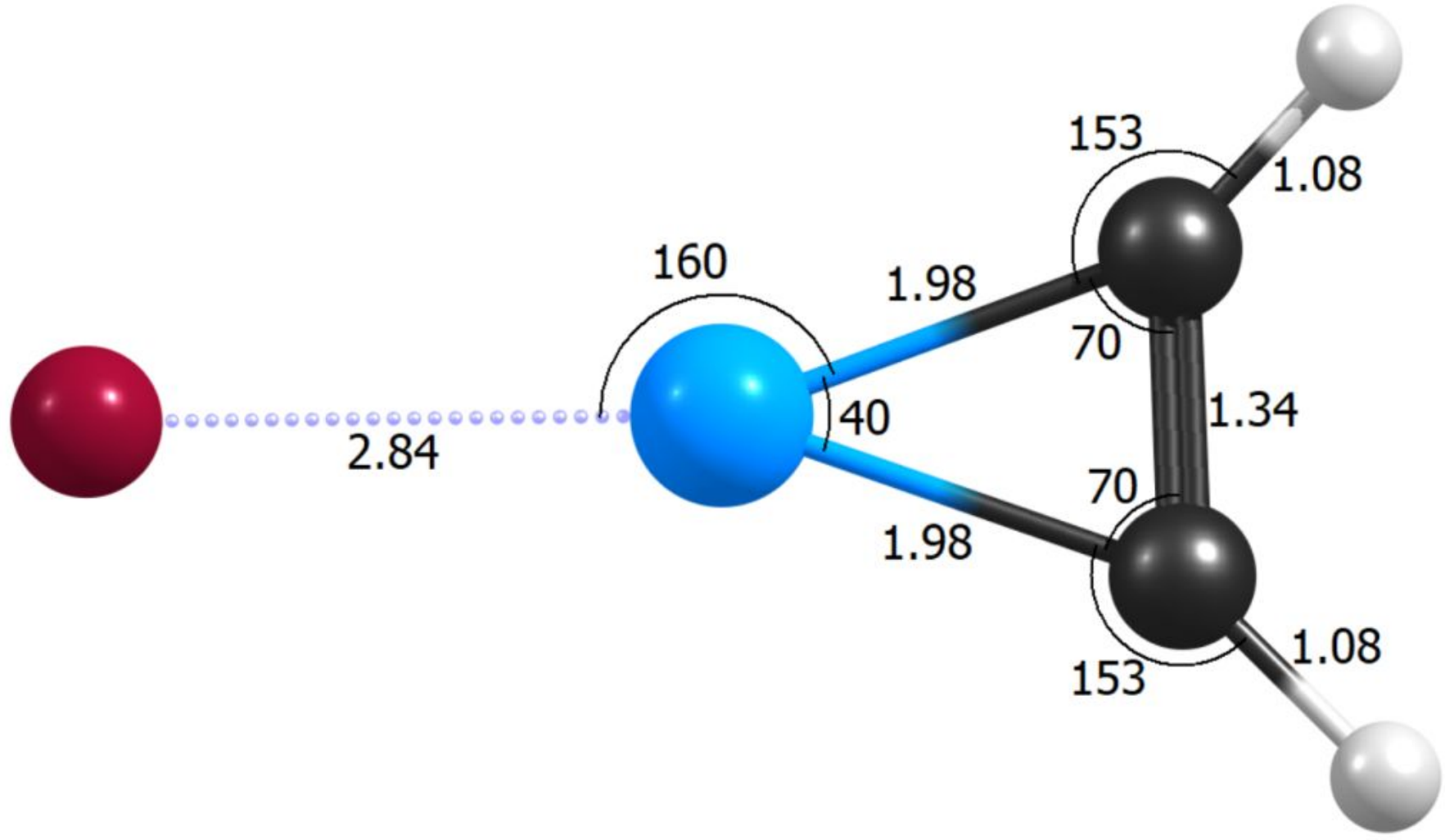

Figure S5. The optimized geometry of isomer 1aI-singlet $Ta^{+}C_2H_2Ar$ followed by its predicted frequencies (cm^{-1}) and IR intensities (km/mol).

Frequency (cm^{-1})	Intensity (km/mol)
22.53	0.0559
57.63	0.0000
116.19	7.6840
596.03	2.8564
687.54	13.9449
753.34	74.5219
824.98	0.3894
951.85	0.0000
1084.45	65.0800
1423.46	3.4036
3176.41	20.7097
3200.06	39.0679

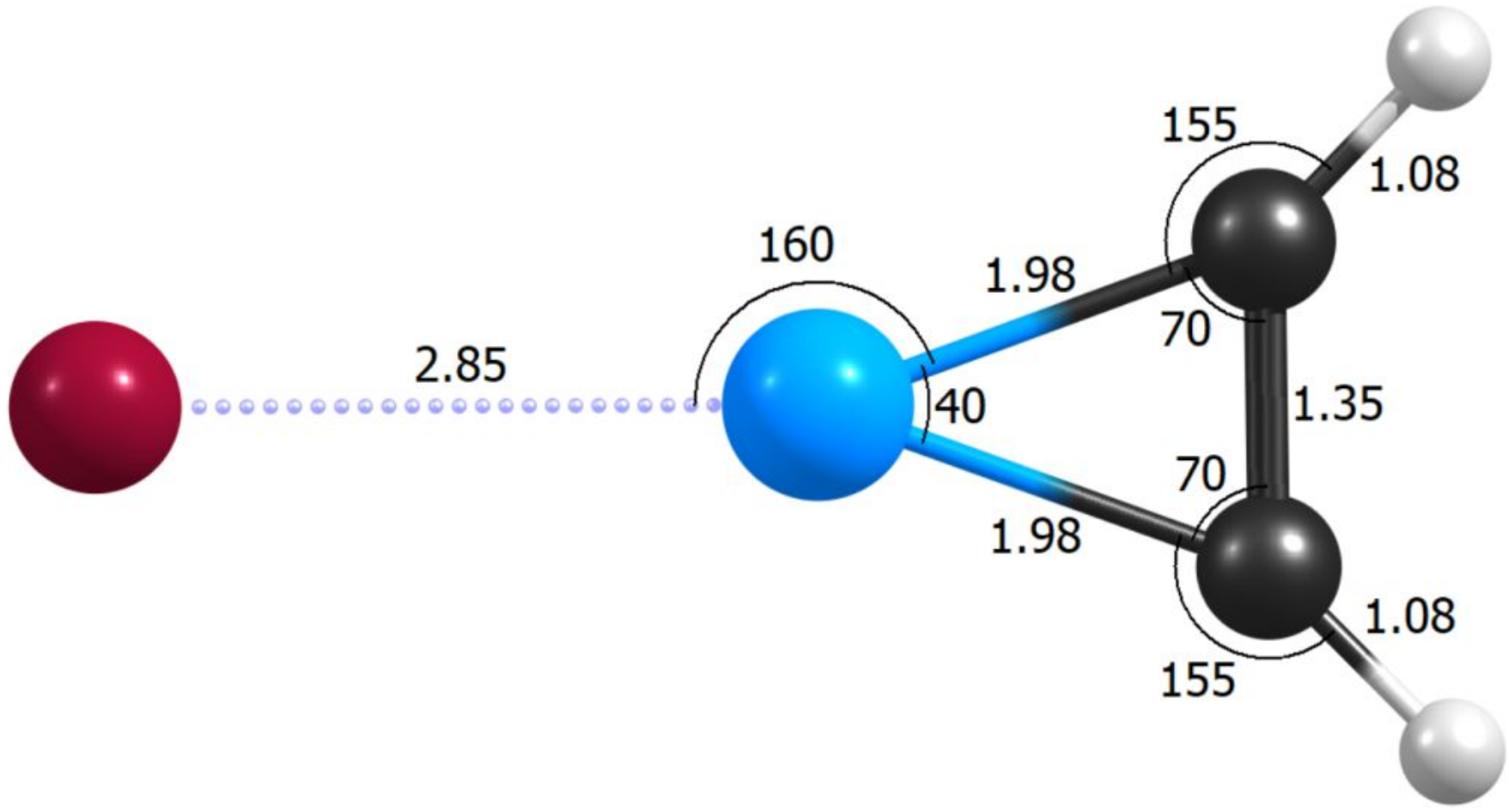

Figure S6. The optimized geometry of isomer 1aI-triplet $Ta^{+}C_2H_2Ar$ followed by its predicted frequencies (cm^{-1}) and IR intensities (km/mol).

Frequency (cm^{-1})	Intensity (km/mol)
22.02	0.0815
54.21	0.0087
114.72	8.0646
596.48	8.2328
692.05	17.9980
746.34	75.4719
815.40	0.0066
952.28	0.0000
1088.93	65.3568
1402.29	0.3897
3176.89	20.7941
3199.00	40.2494

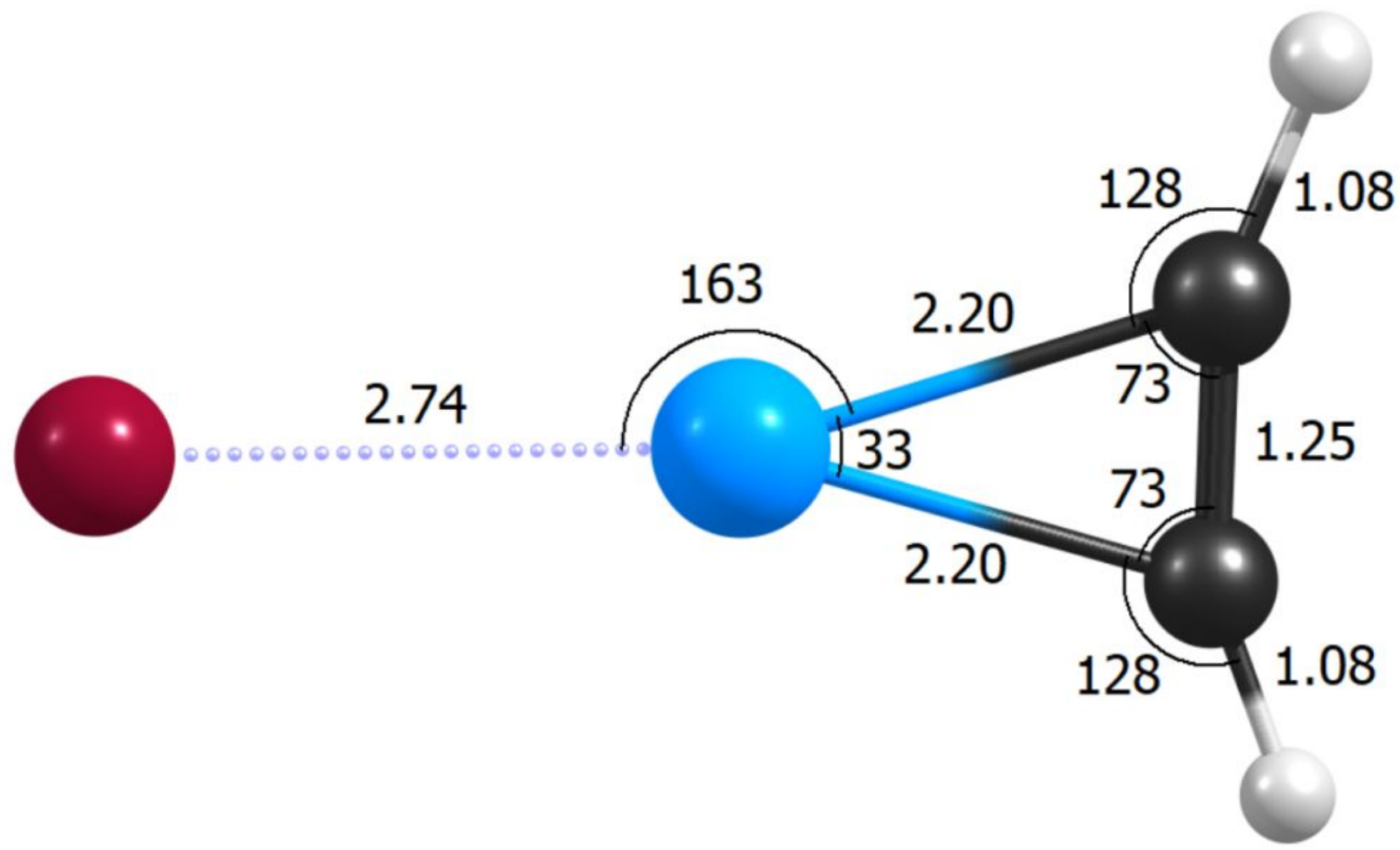

Figure S7. The optimized geometry of isomer 1aI-quintet $Ta^+C_2H_2Ar$ followed by its predicted frequencies (cm^{-1}) and IR intensities (km/mol).

Frequency (cm^{-1})	Intensity (km/mol)
27.69	0.4483
48.47	0.3254
134.57	7.6961
429.06	0.2955
444.03	6.6316
678.97	0.0000
680.80	60.1679
703.96	81.8834
773.34	0.7043
1785.90	21.8536
3212.05	142.8481
3283.40	83.8173

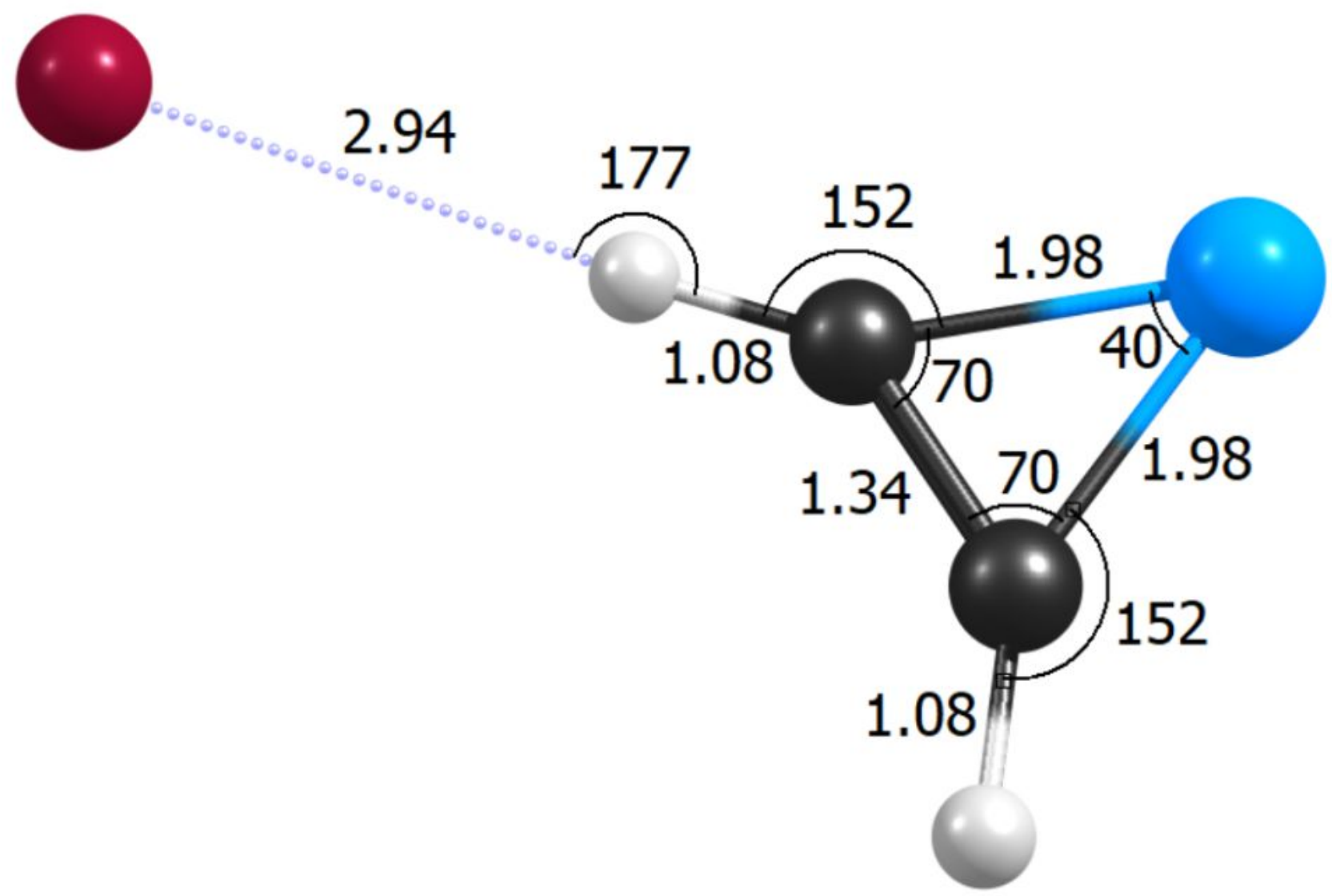

Figure S8. The optimized geometry of isomer 1aII-singlet $Ta^+C_2H_2Ar$ followed by its predicted frequencies (cm^{-1}) and IR intensities (km/mol).

Frequency (cm^{-1})	Intensity (km/mol)
11.55	0.5186
24.62	0.0798
29.75	0.9690
603.45	0.1482
680.04	15.6812
751.42	76.9354
835.03	0.0672
949.68	0.0241
1080.71	78.5404
1427.12	0.5685
3170.43	54.5474
3195.81	59.4004

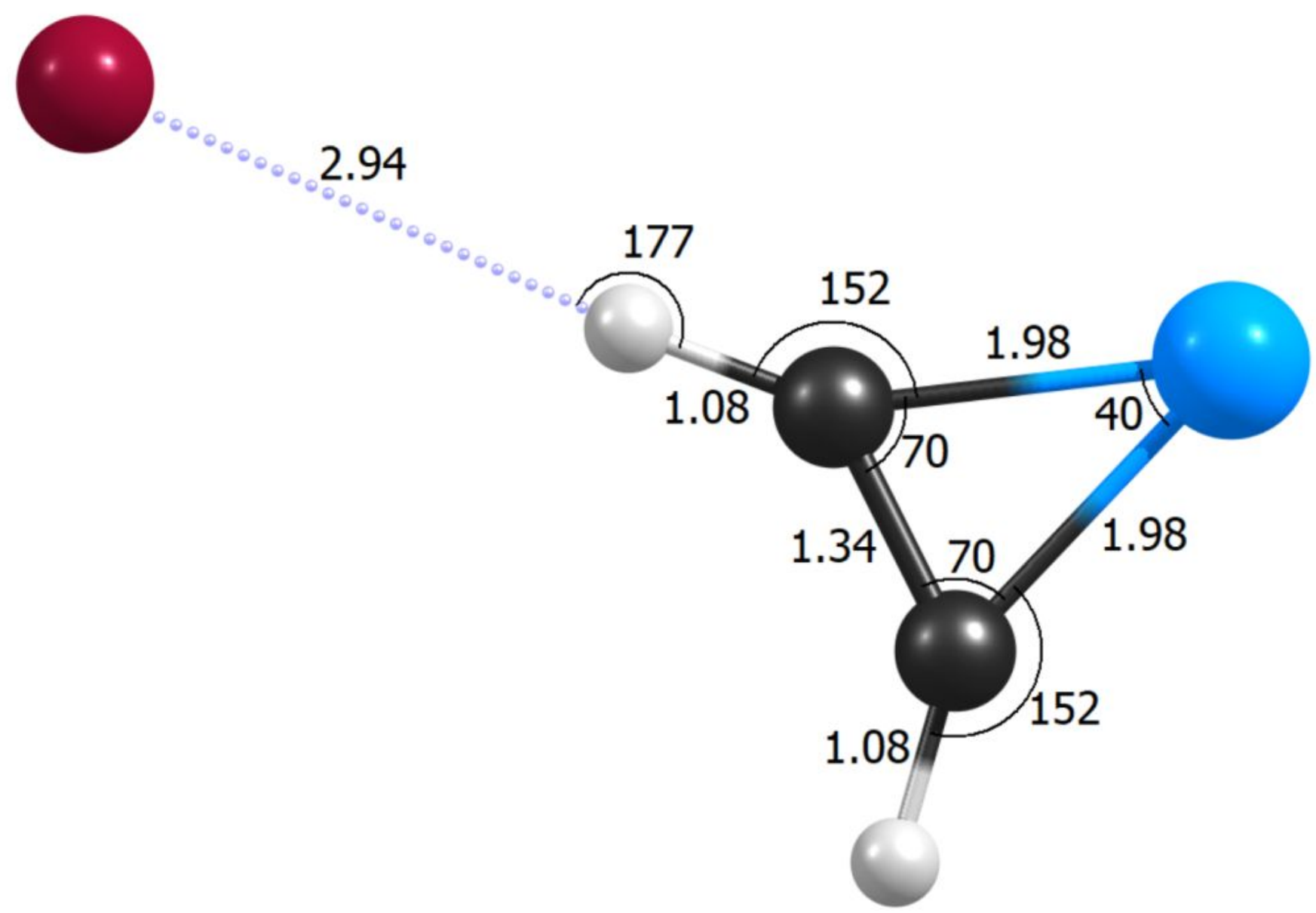

Figure S9. The optimized geometry of isomer 1aII-triplet $Ta^+C_2H_2Ar$ followed by its predicted frequencies (cm^{-1}) and IR intensities (km/mol).

Frequency (cm^{-1})	Intensity (km/mol)
11.97	0.5569
24.02	0.1016
29.24	0.9616
605.92	3.0345
684.46	19.8000
744.46	76.8263
826.03	0.4793
950.19	0.0268
1085.93	78.6290
1409.23	0.9282
3170.68	54.5877
3194.67	59.1724

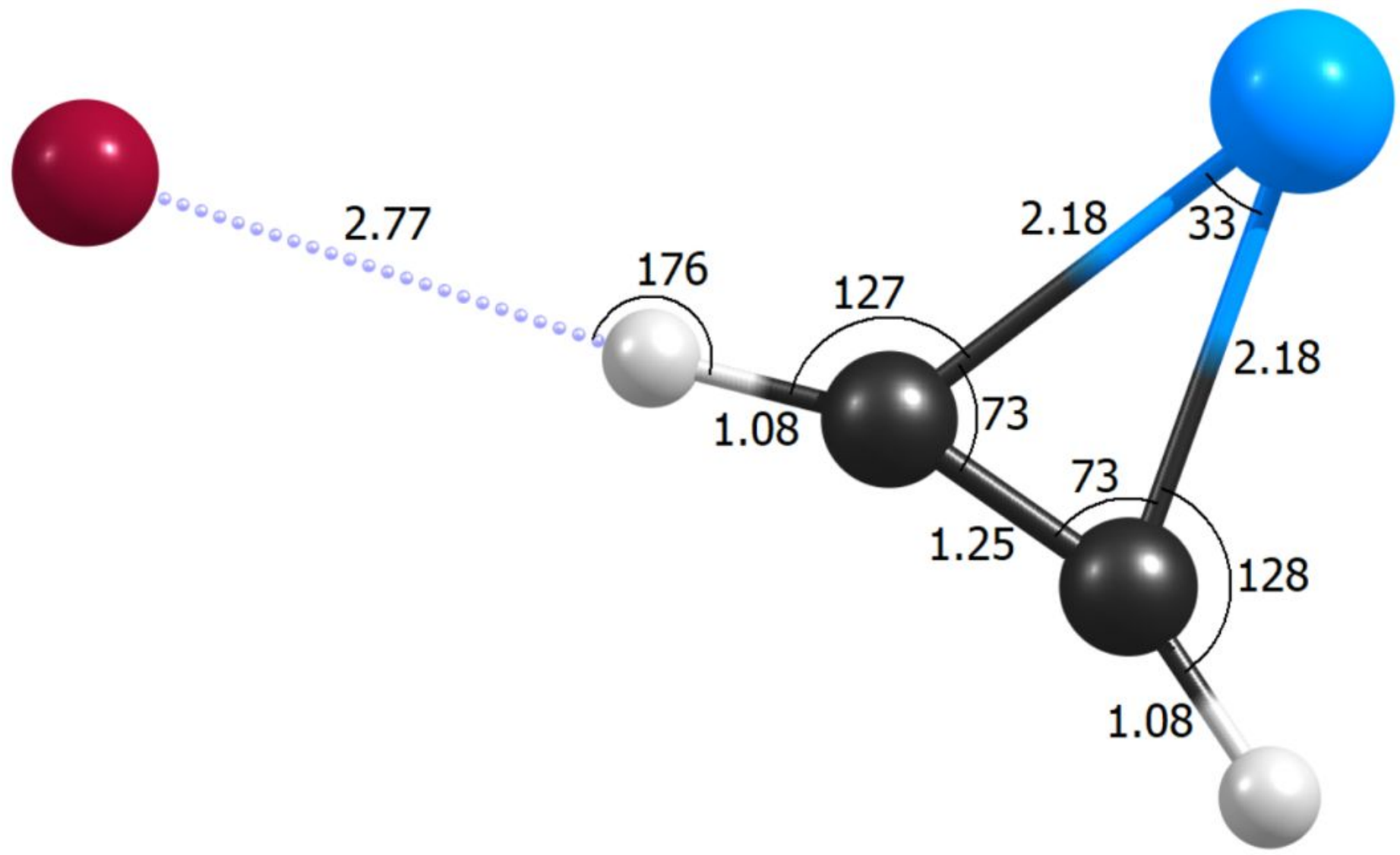

Figure S10. The optimized geometry of isomer 1aII-quintet $Ta^+C_2H_2Ar$ followed by its predicted frequencies (cm^{-1}) and IR intensities (km/mol).

Frequency (cm^{-1})	Intensity (km/mol)
12.06	0.1977
38.21	0.0142
44.45	2.3519
436.76	6.4795
438.56	2.1904
678.09	6.7181
684.07	69.3455
706.75	65.6925
778.15	1.6556
1773.39	11.3428
3192.51	264.2706
3267.80	103.9547

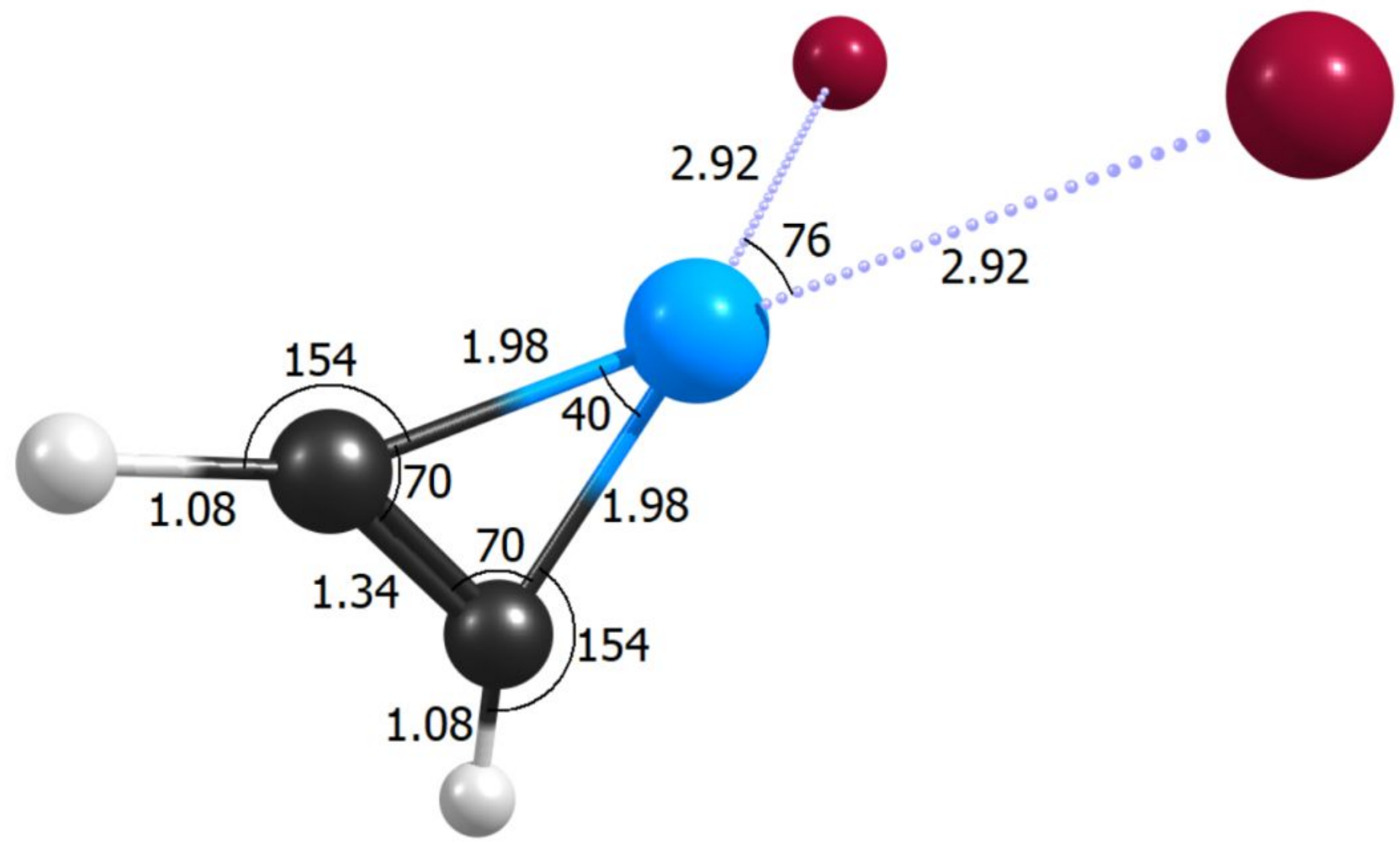

Figure S11. The optimized geometry of isomer 1aI-singlet $Ta^+C_2H_2Ar_2$ followed by its predicted frequencies (cm^{-1}) and IR intensities (km/mol).

Frequency (cm^{-1})	Intensity (km/mol)
56.14	0.5139
61.44	0.0005
69.92	0.0789
85.71	6.5142
90.66	0.0000
101.85	9.8670
593.81	4.8713
687.33	15.5080
752.62	72.3979
832.61	0.2606
953.69	0.0000
1089.15	66.9053
1418.05	4.1384
3173.99	13.7074
3197.27	28.7259

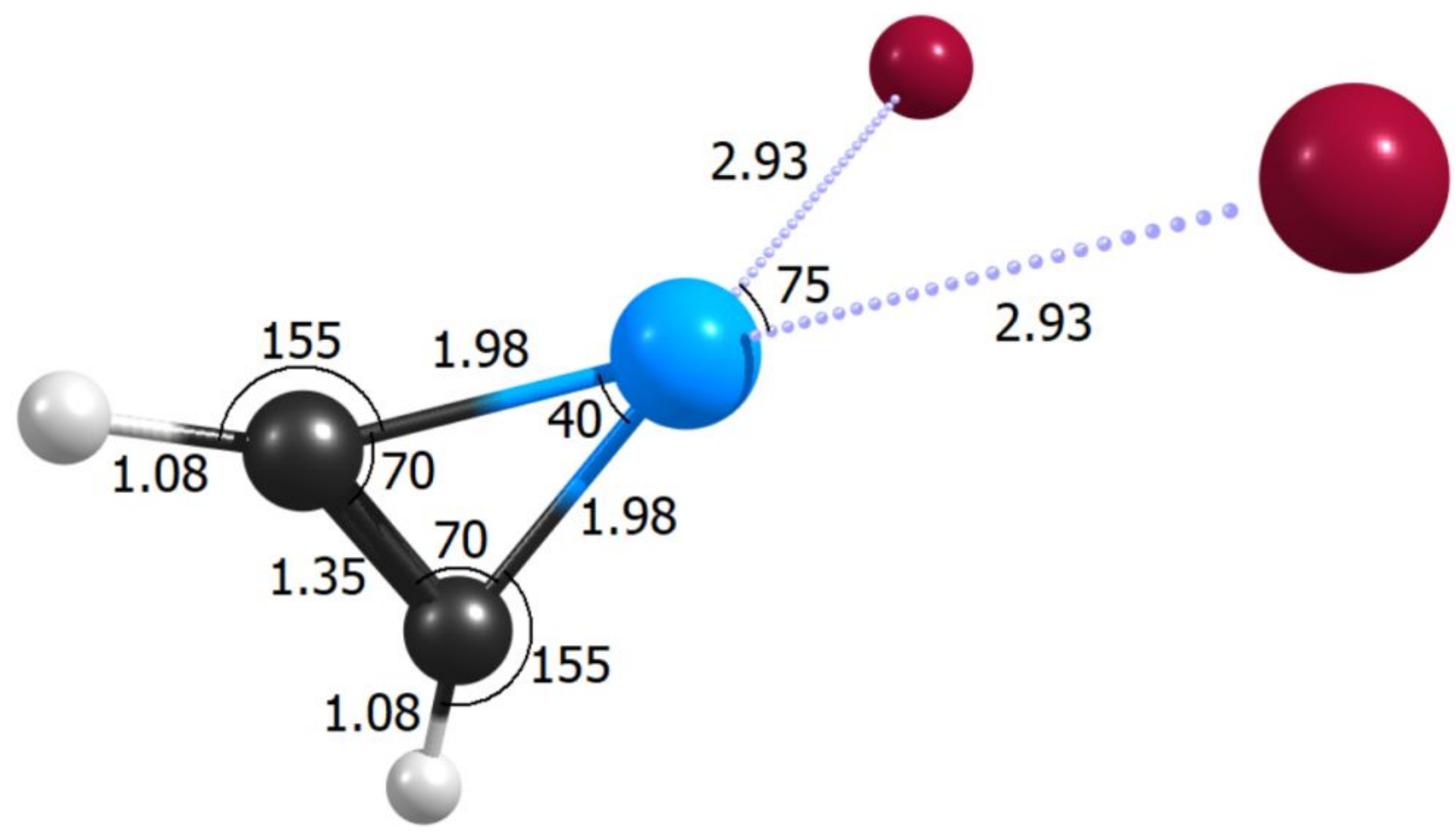

Figure S12. The optimized geometry of isomer 1aI-triplet $Ta^+C_2H_2Ar_2$ followed by its predicted frequencies (cm^{-1}) and IR intensities (km/mol).

Frequency (cm^{-1})	Intensity (km/mol)
58.68	0.4622
60.28	0.0062
66.72	0.0321
80.80	0.0000
82.71	6.6259
99.72	9.5318
598.40	10.2414
689.84	17.8406
746.63	71.3720
824.81	0.0000
954.12	0.0000
1091.50	68.4885
1410.48	0.8665
3174.18	14.3059
3196.57	29.6343

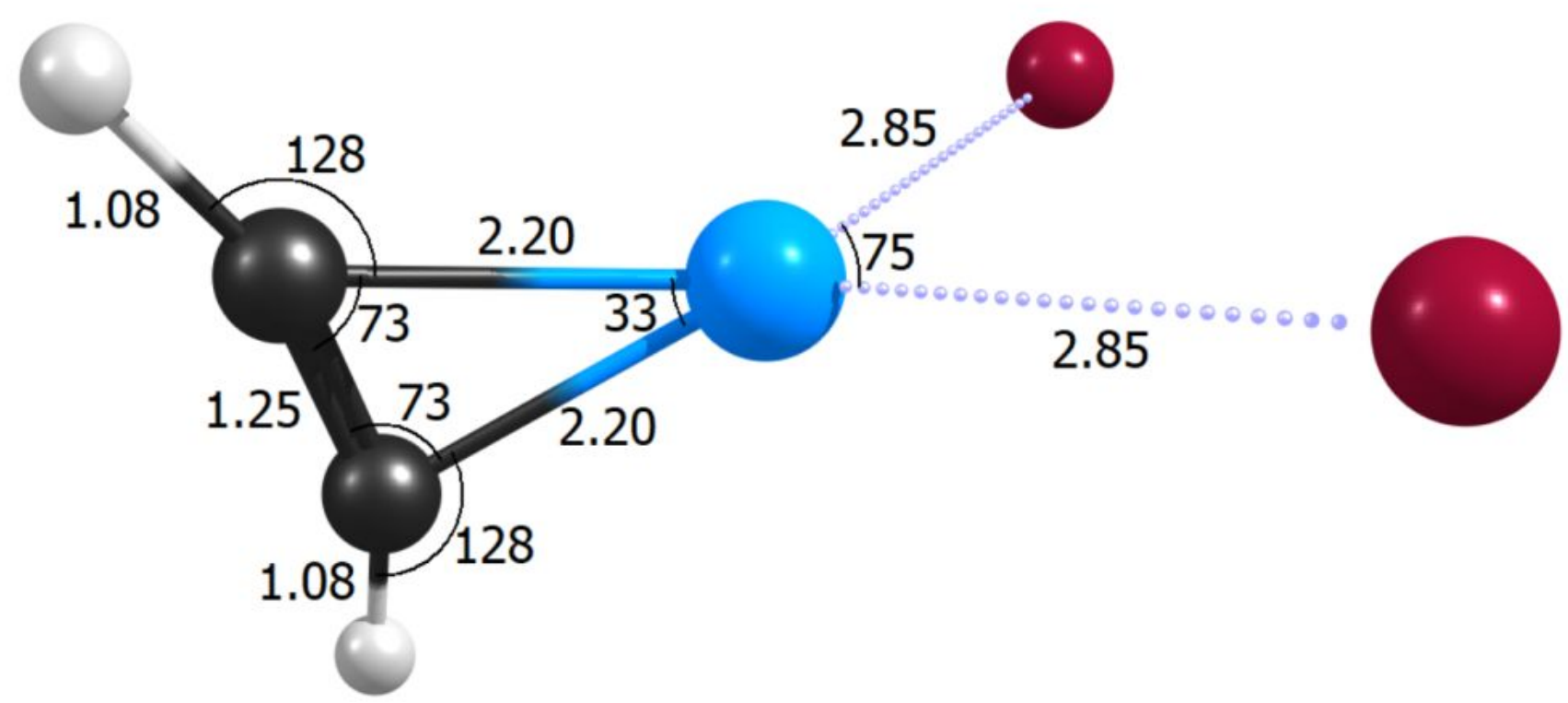

Figure S13. The optimized geometry of isomer 1aI-quintet $Ta^{+}C_2H_2Ar_2$ followed by its predicted frequencies (cm^{-1}) and IR intensities (km/mol).

Frequency (cm^{-1})	Intensity (km/mol)
53.30	0.0000
57.52	0.7302
58.03	0.6320
74.30	0.3392
87.92	5.7877
113.01	7.2070
424.52	0.5372
437.96	8.5524
675.89	0.0000
681.45	58.4273
713.39	89.1042
771.29	1.8807
1787.57	30.5374
3215.71	126.1602
3286.28	84.5947

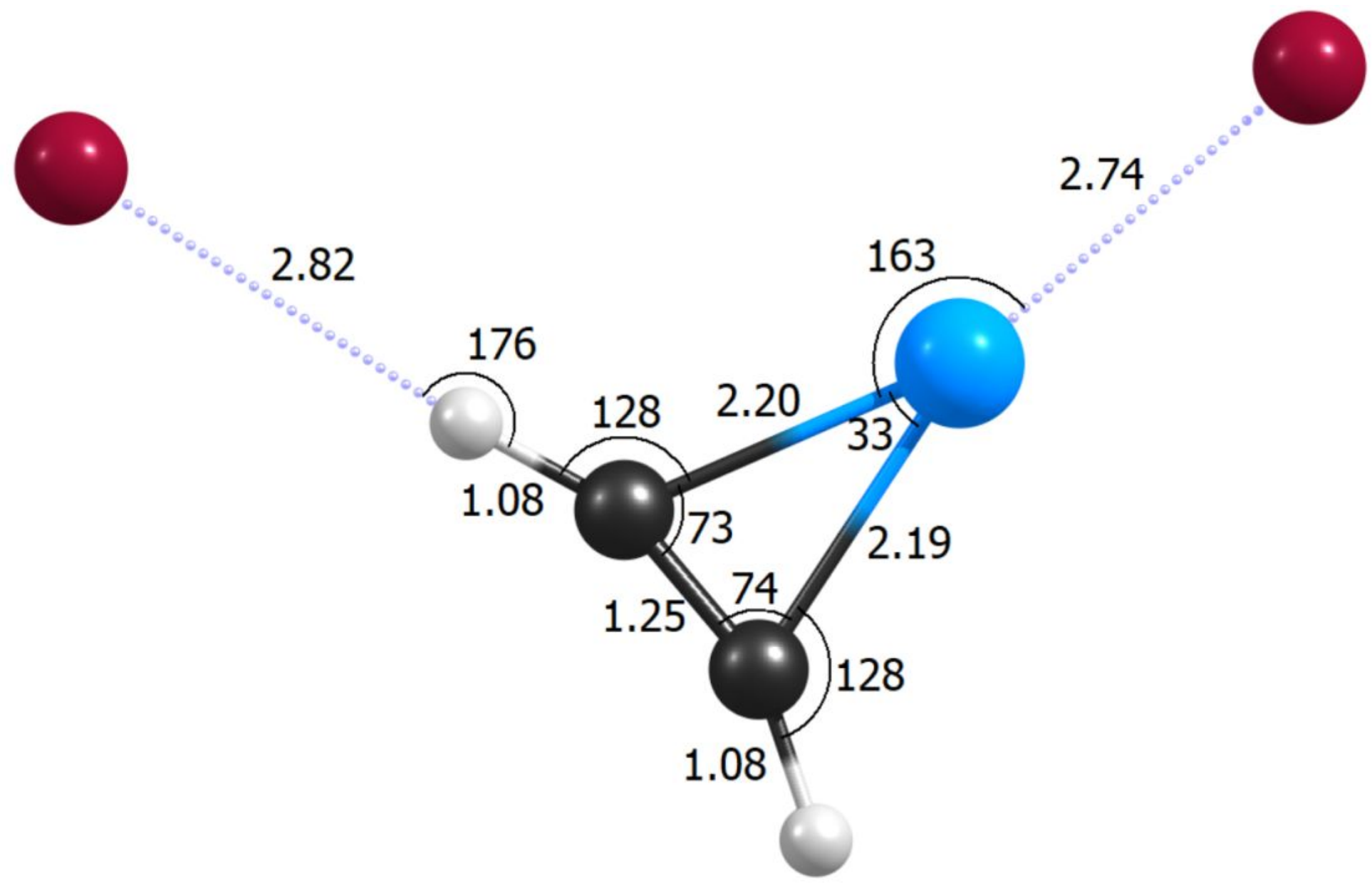

Figure S14. The optimized geometry of isomer 1aII-singlet $Ta^{+}C_2H_2Ar_2$ followed by its predicted frequencies (cm^{-1}) and IR intensities (km/mol).

Frequency (cm^{-1})	Intensity (km/mol)
6.51	0.1456
17.81	0.0095
24.76	0.9218
26.47	0.1007
59.24	0.0370
115.70	7.8730
596.15	2.5103
688.68	11.8368
757.12	68.7396
827.22	1.0747
954.96	0.0229
1085.14	67.4722
1422.85	2.5225
3174.36	37.3862
3198.30	53.3248

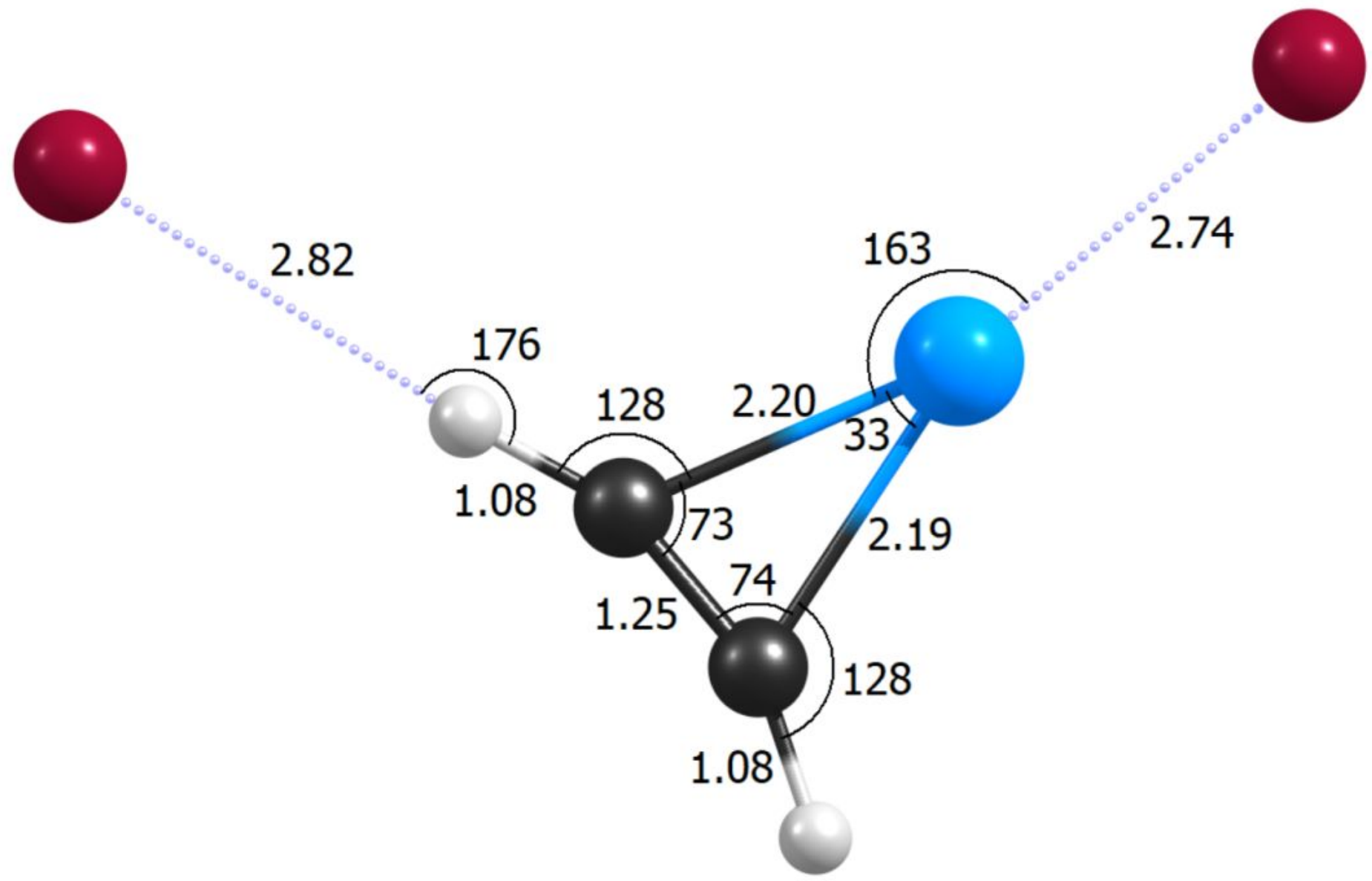

Figure S15. The optimized geometry of isomer 1aII-triplet $Ta^+C_2H_2Ar_2$ followed by its predicted frequencies (cm^{-1}) and IR intensities (km/mol).

Frequency (cm^{-1})	Intensity (km/mol)
6.72	0.1371
17.21	0.0178
24.52	0.9312
26.29	0.1295
55.83	0.0310
114.21	8.2758
596.67	7.7044
693.29	15.5801
750.14	69.6168
817.66	0.1032
955.42	0.0246
1089.71	67.2038
1401.38	0.1932
3174.73	37.7283
3197.18	54.6629

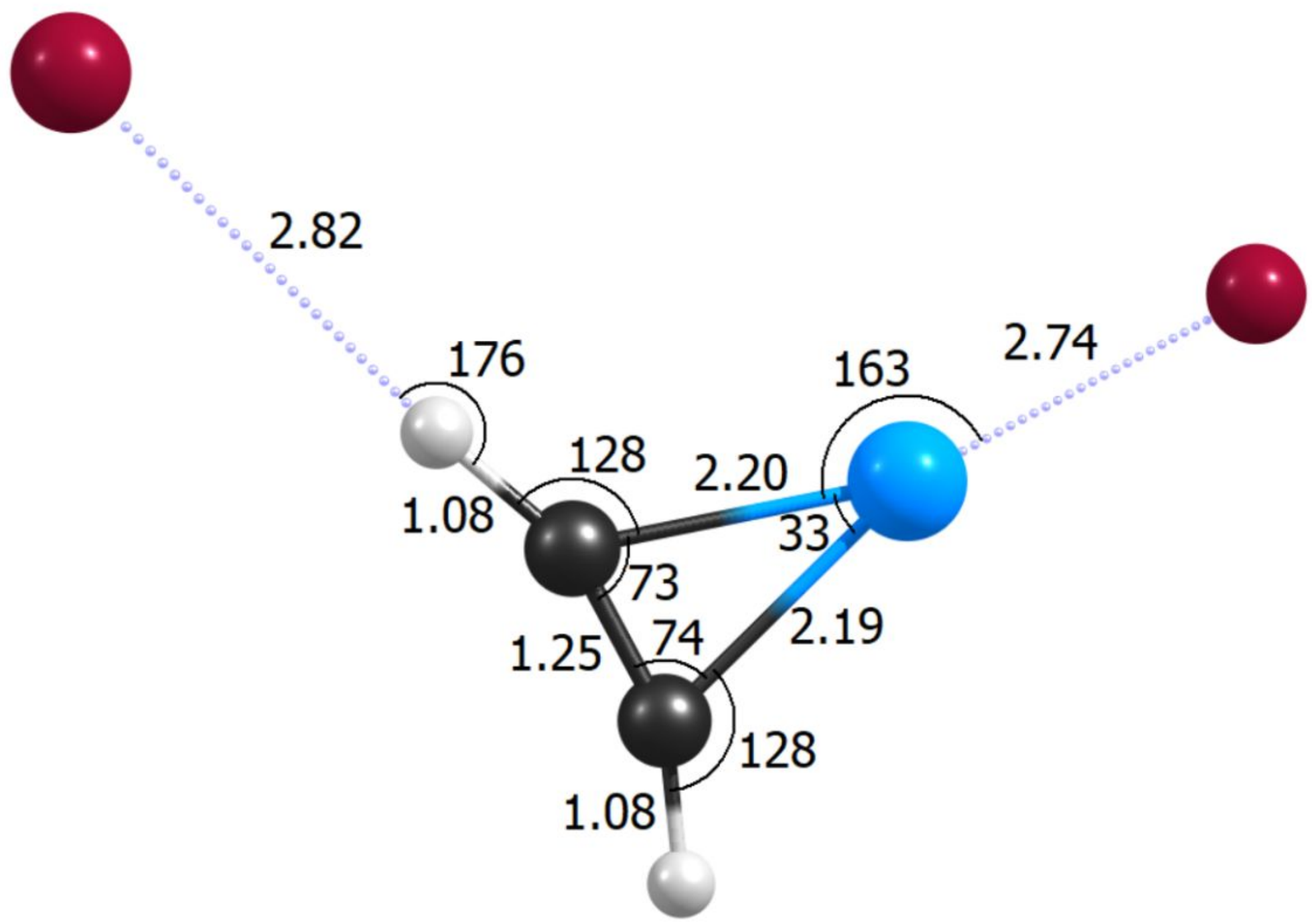

Figure S16. The optimized geometry of isomer 1aII-quintet $Ta^+C_2H_2Ar_2$ followed by its predicted frequencies (cm^{-1}) and IR intensities (km/mol).

Frequency (cm^{-1})	Intensity (km/mol)
7.60	0.1795
26.33	0.3294
31.64	0.7577
35.89	0.0163
55.44	1.3885
133.90	7.7115
429.11	0.2575
444.99	8.8058
681.25	64.4729
682.33	4.9824
711.26	68.0584
776.27	2.4832
1785.42	19.0855
3204.40	219.2058
3278.00	102.4774

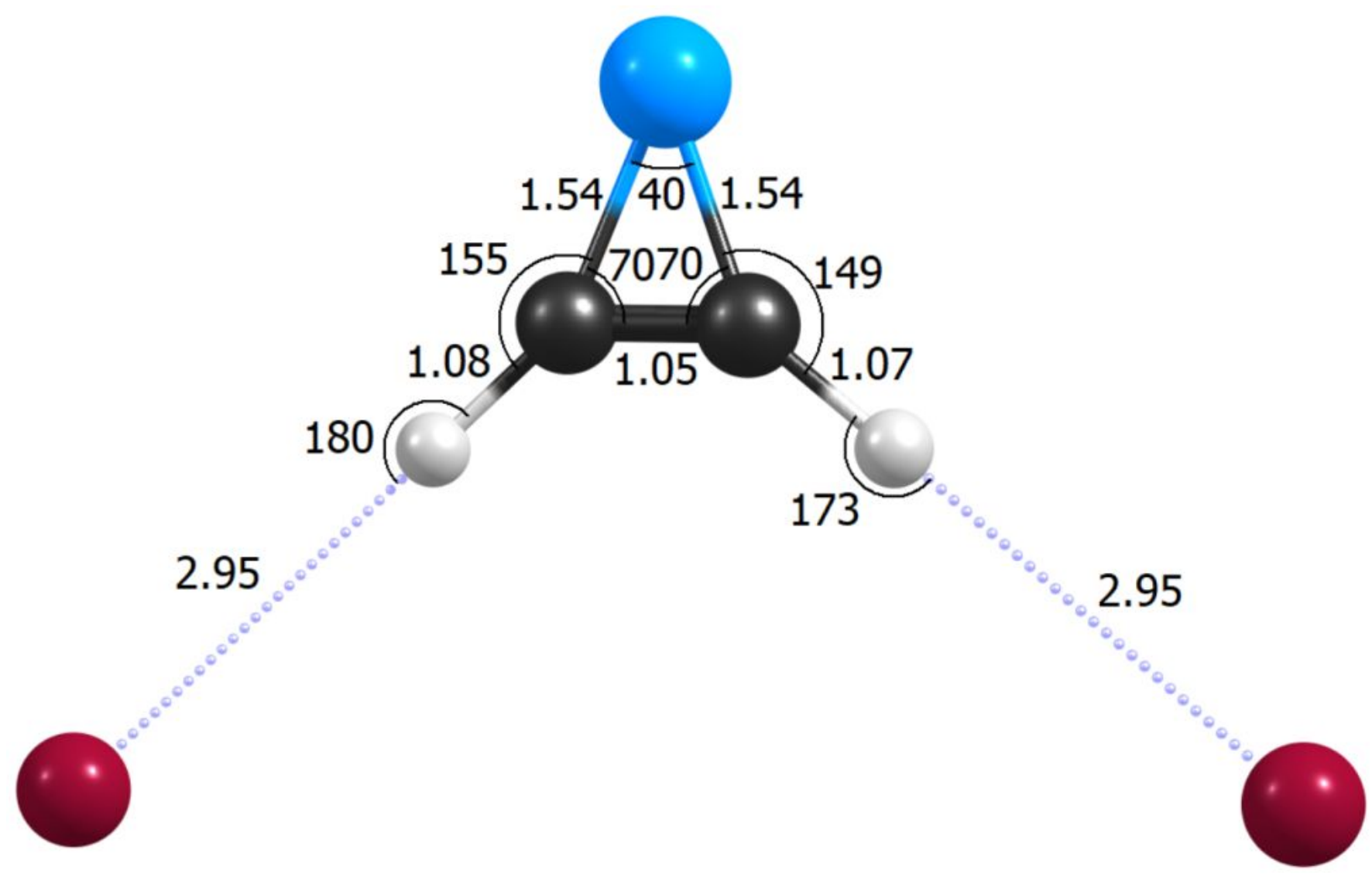

Figure S17. The optimized geometry of isomer 1aIII-singlet $Ta^+C_2H_2Ar_2$ followed by its predicted frequencies (cm^{-1}) and IR intensities (km/mol).

Frequency (cm^{-1})	Intensity (km/mol)
3.58	0.4891
13.66	0.8243
14.25	0.1698
25.92	1.2423
30.09	0.0000
33.37	0.5678
603.53	0.0709
681.39	13.1832
755.76	70.2792
837.41	0.4499
953.07	0.0000
1081.27	81.0284
1426.41	0.1255
3168.07	69.1285
3192.91	83.1016

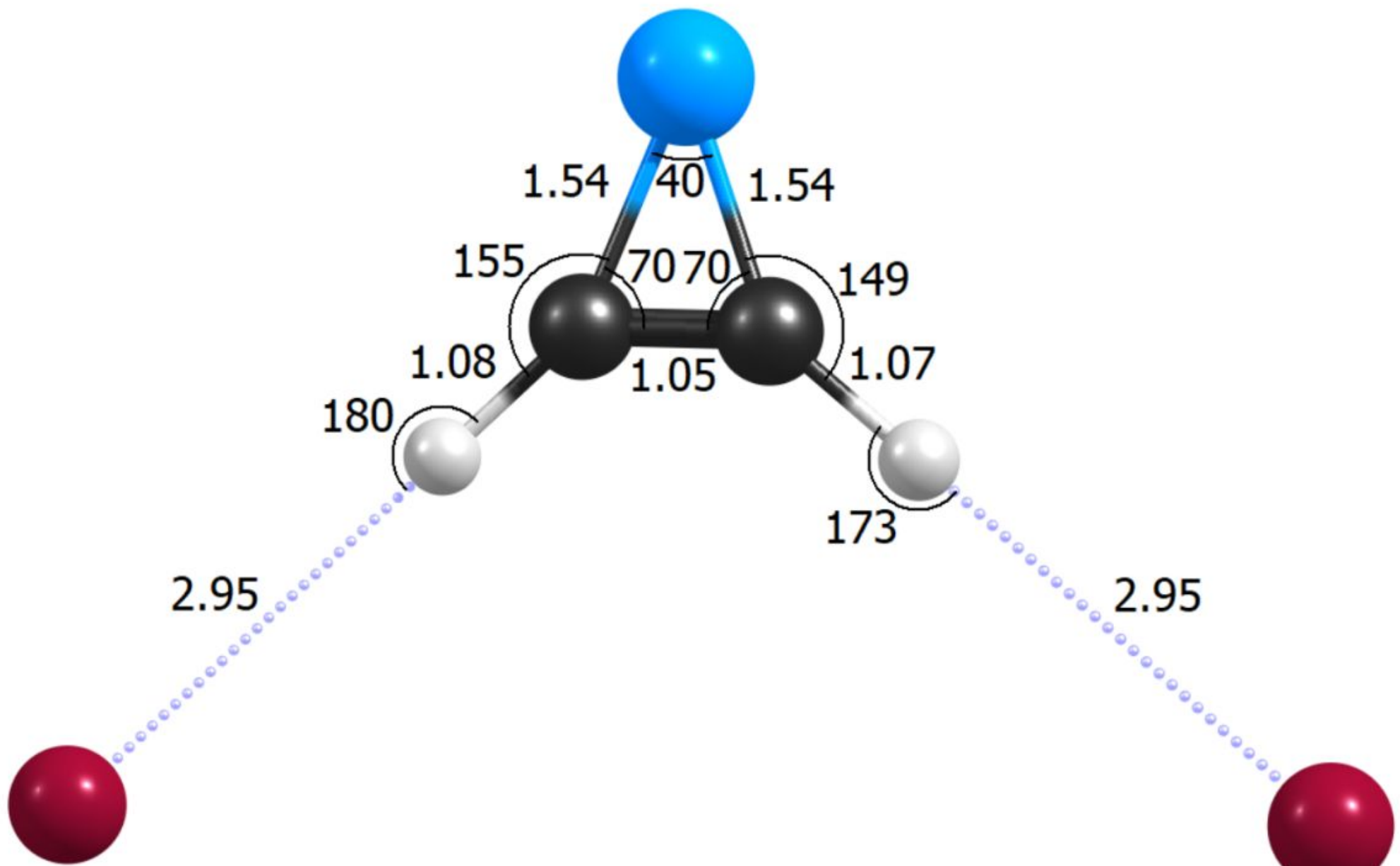

Figure S18. The optimized geometry of isomer 1aIII-triplet $Ta^{+}C_2H_2Ar_2$ followed by its predicted frequencies (cm^{-1}) and IR intensities (km/mol).

Frequency (cm^{-1})	Intensity (km/mol)
3.97	0.4529
14.62	0.9066
15.66	0.2386
26.04	1.3216
31.71	0.0000
32.64	0.4598
606.09	2.7390
685.98	16.9285
748.94	70.1698
828.46	0.0823
953.68	0.0000
1086.64	80.5119
1408.14	1.5036
3168.28	67.9679
3191.62	84.0177

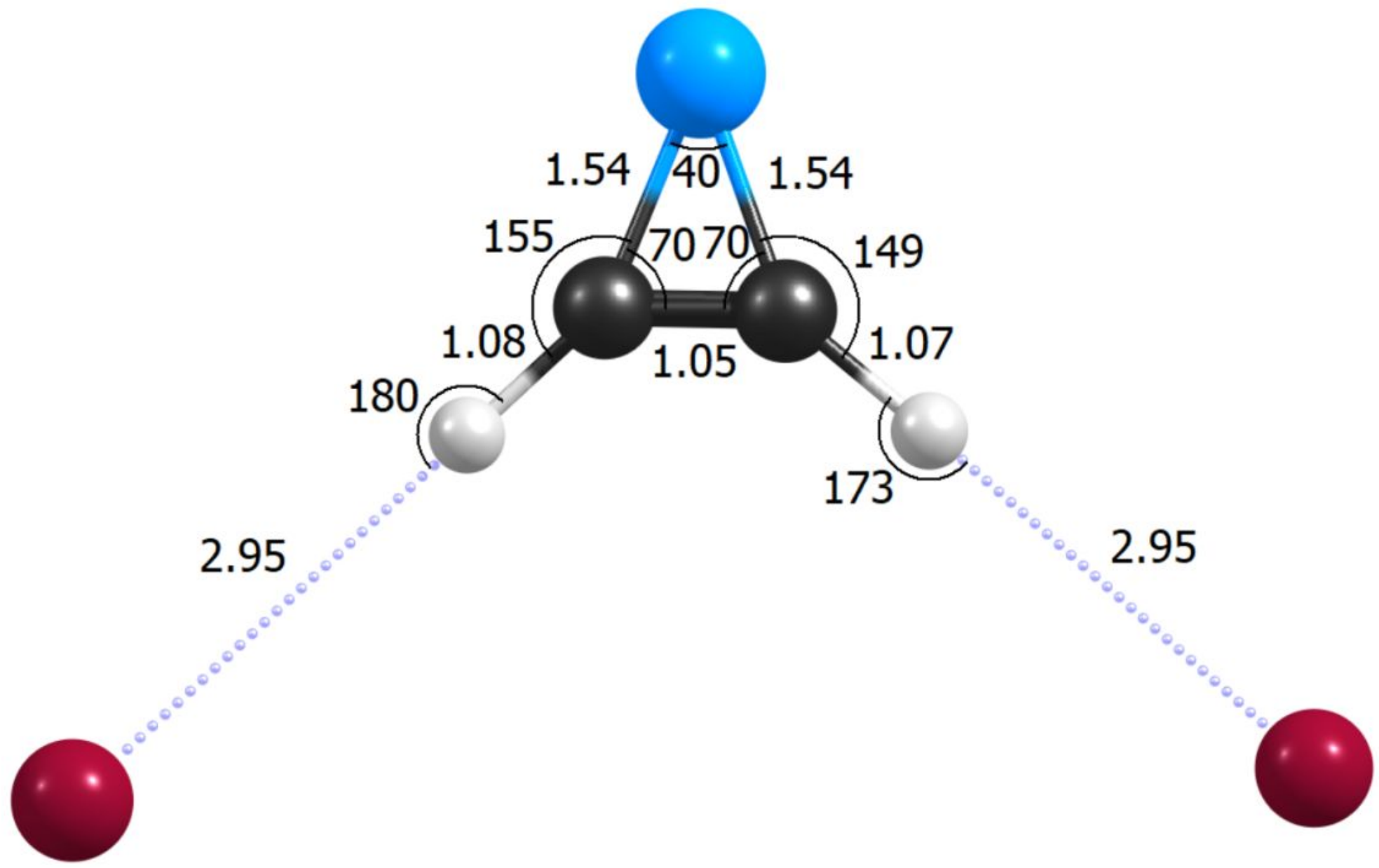

Figure S19. The optimized geometry of isomer 1aIII-quintet $Ta^+C_2H_2Ar_2$ followed by its predicted frequencies (cm^{-1}) and IR intensities (km/mol).

Frequency (cm^{-1})	Intensity (km/mol)
5.48	0.7400
13.51	0.2930
14.34	0.2366
31.50	0.4771
51.05	0.0000
53.89	3.7709
437.71	8.9279
438.35	2.0336
683.07	73.9764
684.72	0.0000
711.71	62.9506
780.99	3.1775
1772.91	8.1081
3184.89	350.2281
3259.28	134.0748

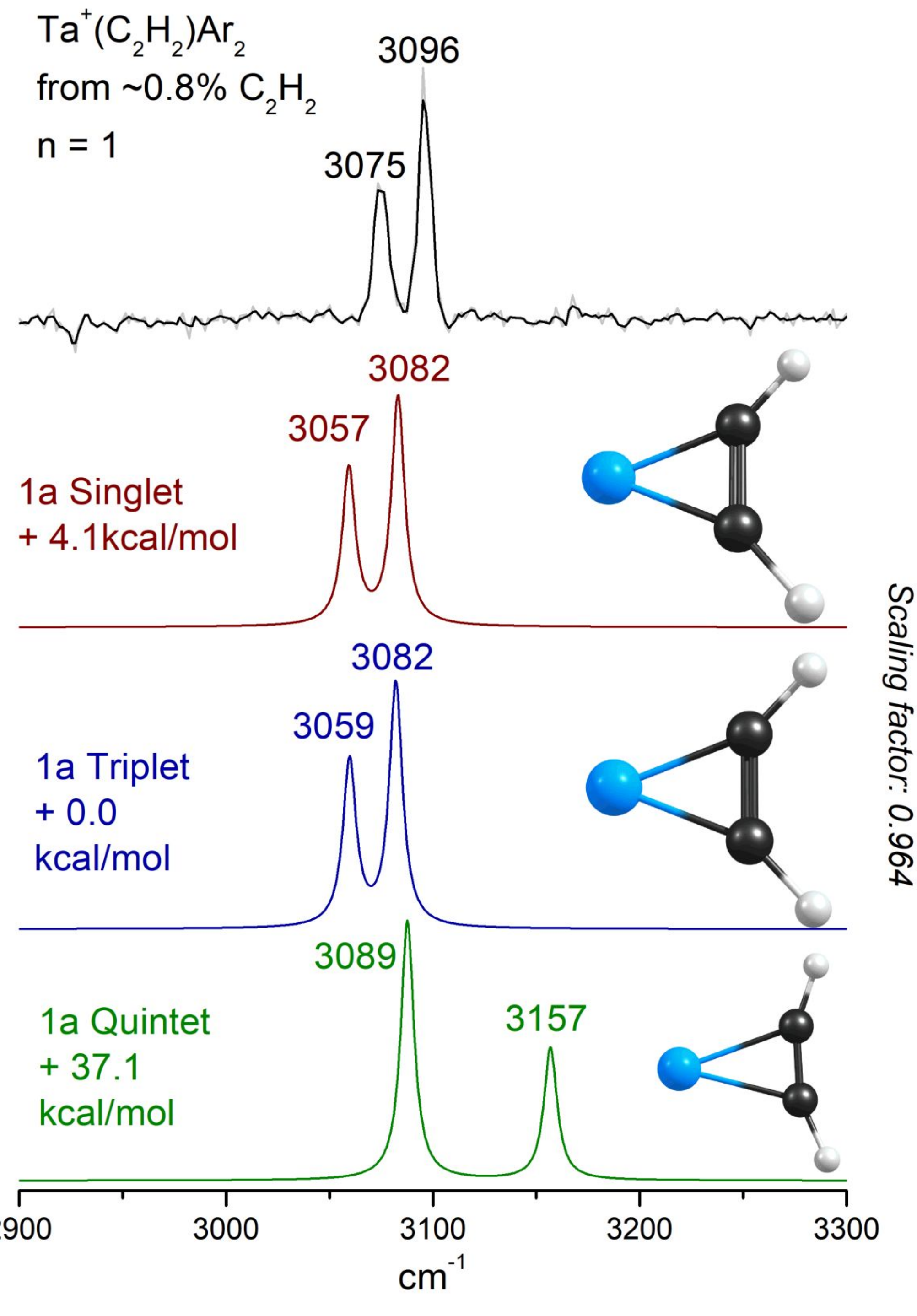

Figure S20. The experimental spectrum measured for $Ta^+C_2H_2Ar_2$ with simulated spectra for $Ta^+(C_2H_2)$ as a singlet, triplet, and quintet.

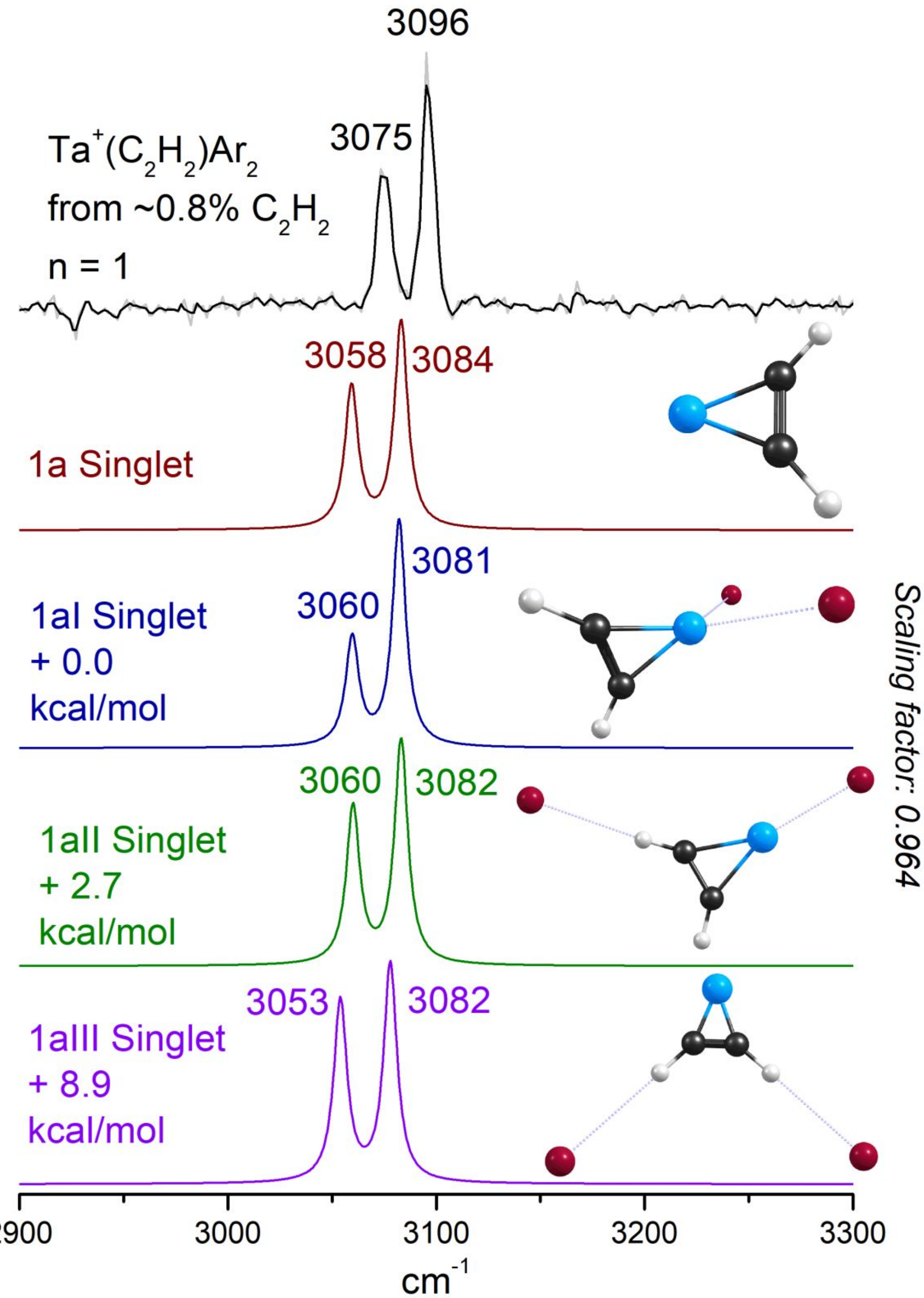

Figure S21. The experimental spectrum measured for $Ta^{+}C_2H_2Ar_2$ with simulated spectra for the 1a-singlet and doubly-tagged singlet isomers.

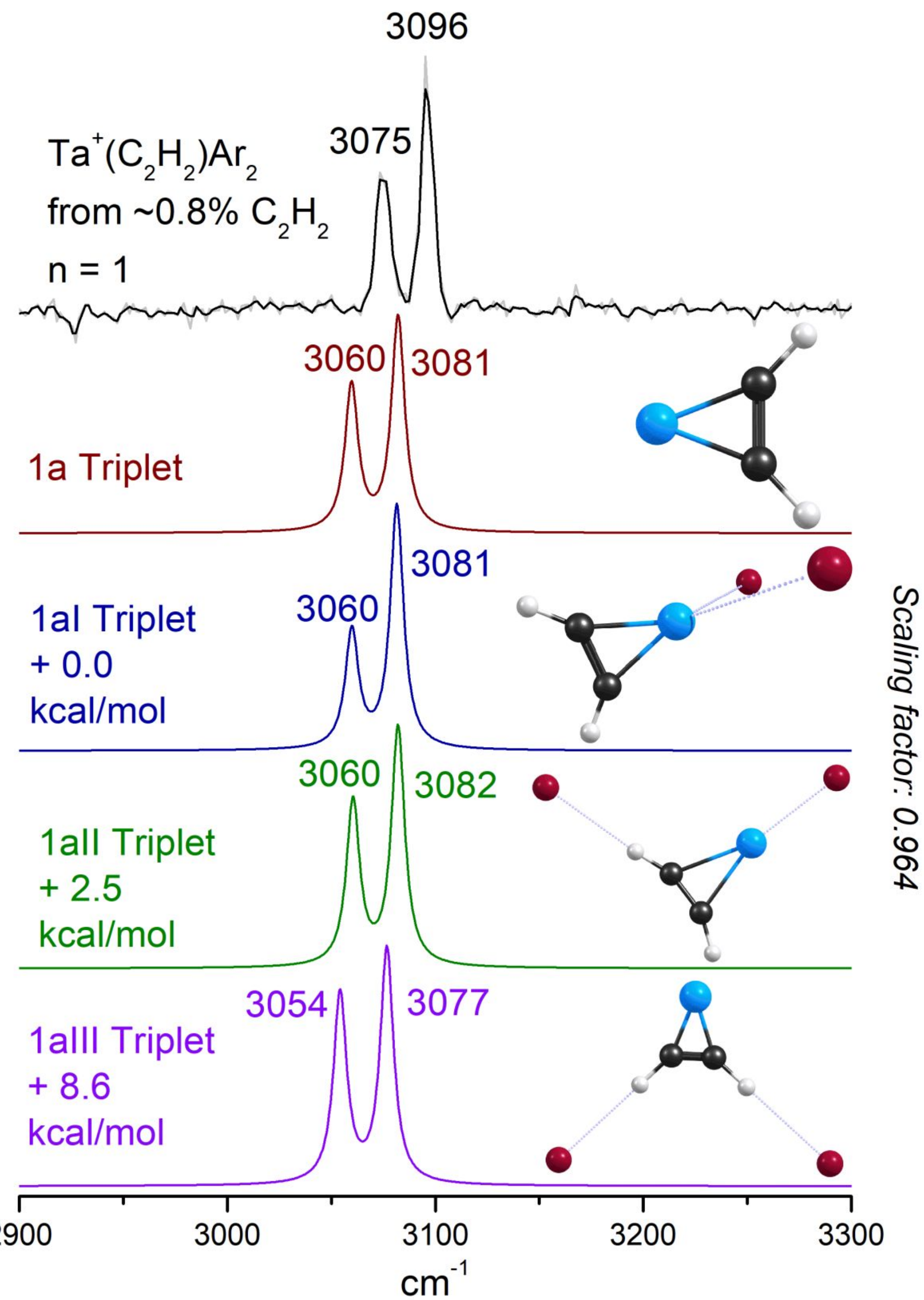

Figure S22. The experimental spectrum measured for $Ta^{+}C_2H_2Ar_2$ with simulated spectra for the 1a-triplet and doubly-tagged triplet isomers.

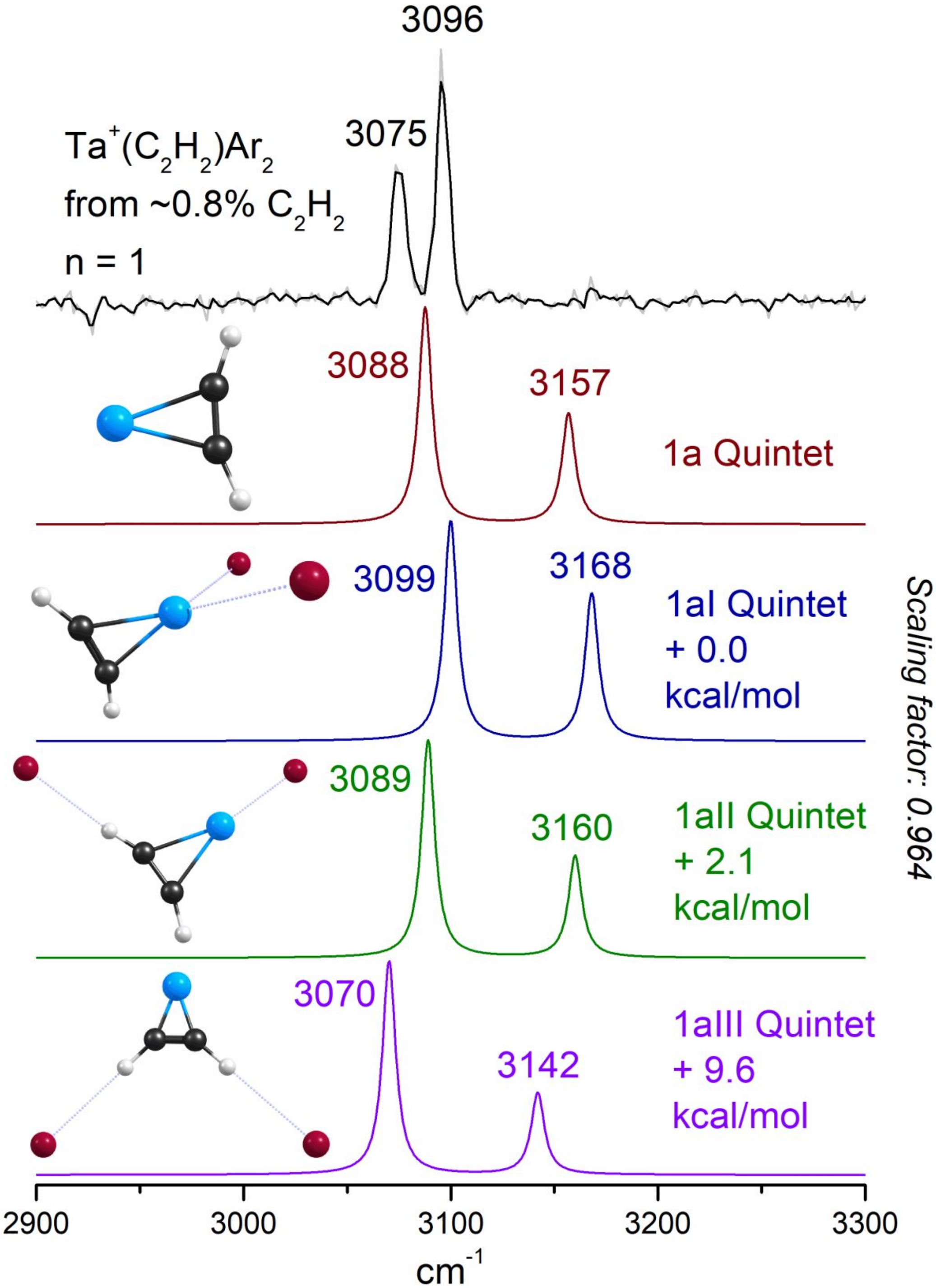

Figure S23. The experimental spectrum measured for $Ta^+C_2H_2Ar_2$ with simulated spectra for the 1a-quintet and doubly-tagged quintet isomers.

Table S6. $Ta^+C_4H_2$ electronic energy calculated at the B3LYP/cc-pVTZ(-pp) level with Stuttgardt/Koeln pseudopotential.

Isomer	2s + 1	E (hartree)	Relative E (kcal/mol)
2a	1	-210.693054	+1.3
2a	3	-210.69506	+0.0
2a	5	-210.613687	+51.1
2b	1	-210.655709	+24.7
2b	3	-210.661137	+21.3
2b	5	-210.584862	+69.2
2c	1	-210.641421	+33.7
2c	3	-210.634917	+37.7
2c	5	-210.591961	+64.7
2d	1	-210.592836	+64.1
2d	3	-210.586653	+68.0
2d	5	-210.562076	+83.4
TS1	1	-210.594182	+63.3
TS1	3	-210.579346	+72.6
TS2	1	-210.591934	+64.7
TS2	3	-210.548126	+92.2

Table S7. $Ta^+C_4H_2Ar$ electronic energy calculated at the B3LYP/cc-pVTZ(-pp) level with Stuttgardt/Koeln pseudopotential.

Isomer	2s + 1	E (hartree)	Relative E (kcal/mol)	Ar BE (kcal/mol)
2aI	1	-738.262053	+0.0	6.0
2aII	1	-738.252702	+5.9	0.2
2aI	3	-738.265142	+0.0	6.7
2aII	3	-738.254661	+6.6	0.1
2aI	5	-738.182322	+0.0	5.8
2aII	5	-738.17336	+5.6	0.2
2bI	1	-738.222024	+0.0	4.4
2bII	1	-738.215662	+4.0	0.4
2bI	3	-738.230797	+0.0	6.5
2bII	3	-738.22107	+6.1	0.4
2bI	5	-738.154675	+0.0	6.6
2bII	5	-738.144758	+6.2	0.3
2cI	1	-738.210821	+0.0	6.3
2cII	1	-738.201051	+6.1	0.2
2cI	3	-738.205695	+0.0	7.2
2cII	3	-738.195595	+6.3	0.8
2cI	5	-738.162673	+0.0	7.1
2cII	5	-738.151705	+6.9	0.2
2dI	1	-738.164286	+0.0	7.6
2dII	1	-738.152329	+7.5	0.1
2dI	3	-738.160326	+0.0	9.0
2dII	3	-738.146174	+8.9	0.1
2dI	5	-738.137743	+0.0	10.2
2dII	5	-738.12176	+10.0	0.2

Table S8. Energetics for the formation of $Ta^+C_4H_2$ isomers and transition states from Ta^+ + $2C_2H_2$ at the B3LYP/cc-pVTZ(-pp) level with Stuttgardt/Koeln pseudopotential.

isomer	2s+1	E (hartree)	$[E(Ta^+C_4H_2) + E(H_2)]$- $[E(Ta^+) + 2C_2H_2]$ (kcal/mol)
H_2	1	-1.16365	
C_2H_2	1	-77.332652	
Ta^+	1	-57.003052	
Ta^+	3	-57.027178	
$Ta^+ + 2C_2H_2$	1	-211.668356	+15.14
$Ta^+ + 2C_2H_2$	3	-211.692482	0
2a	1	-210.693054	-103.05
2a	3	-210.69506	-104.31
2b	1	-210.655709	-79.62
2b	3	-210.661137	-83.02
2c	1	-210.641421	-70.65
2c	3	-210.634917	-66.57
2d	1	-210.592836	-40.16
2d	3	-210.586653	-36.28
TS1_ba	1	-210.594182	-41.01
	3	-210.579346	-31.70
TS2_cb	1	-210.591934	-39.60
	3	-210.548126	-12.11

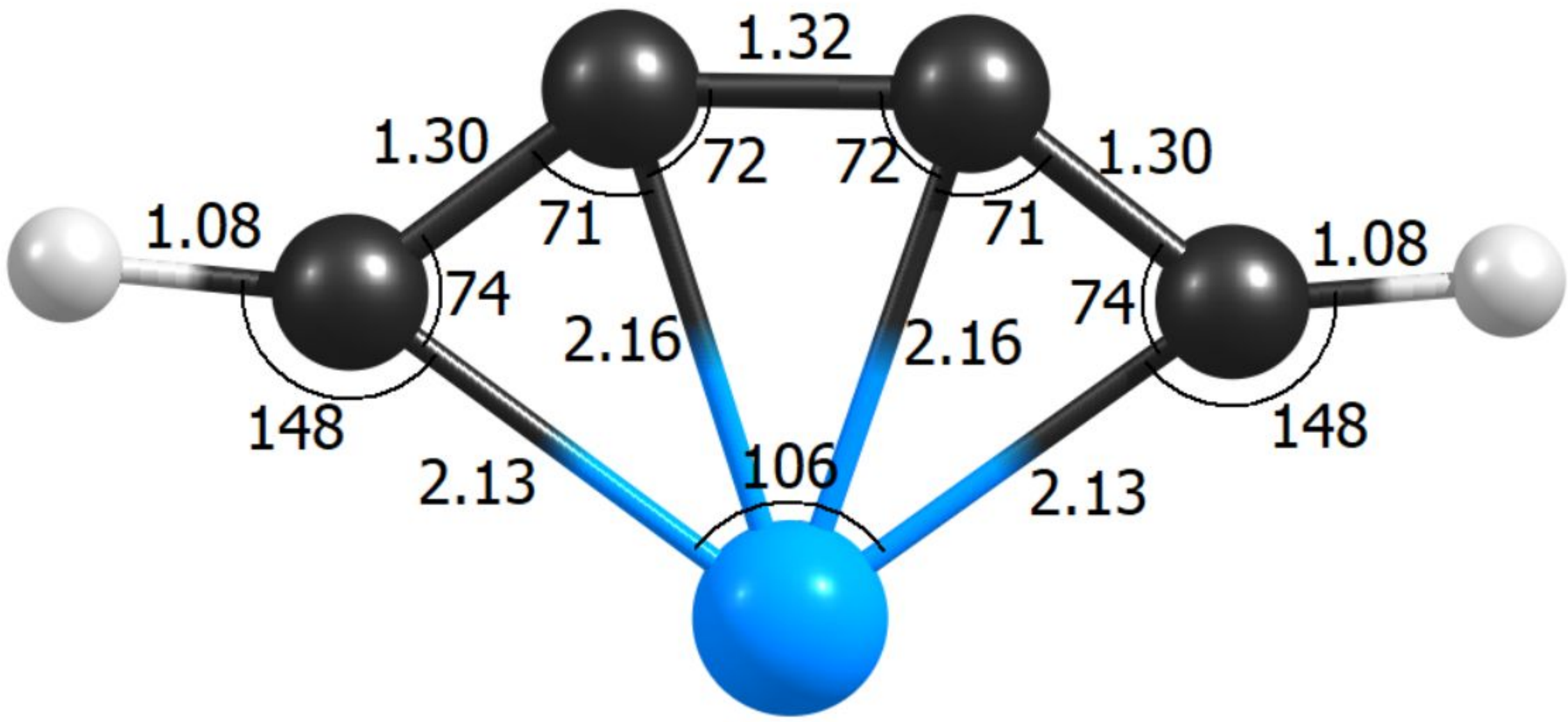

Figure S24. The optimized geometry of isomer 2a-singlet $Ta^+C_4H_2$ followed by its predicted frequencies (cm^{-1}) and IR intensities (km/mol).

Frequency (cm^{-1})	Intensity (km/mol)
204.27	0.7821
421.21	28.7574
443.81	0.5249
535.27	0.0000
599.13	0.1921
630.14	0.1376
715.04	62.3360
869.39	0.0000
921.02	124.8061
922.36	34.9163
1121.93	0.0779
1633.44	5.1693
1925.90	0.3134
3237.34	6.4078
3238.33	155.1061

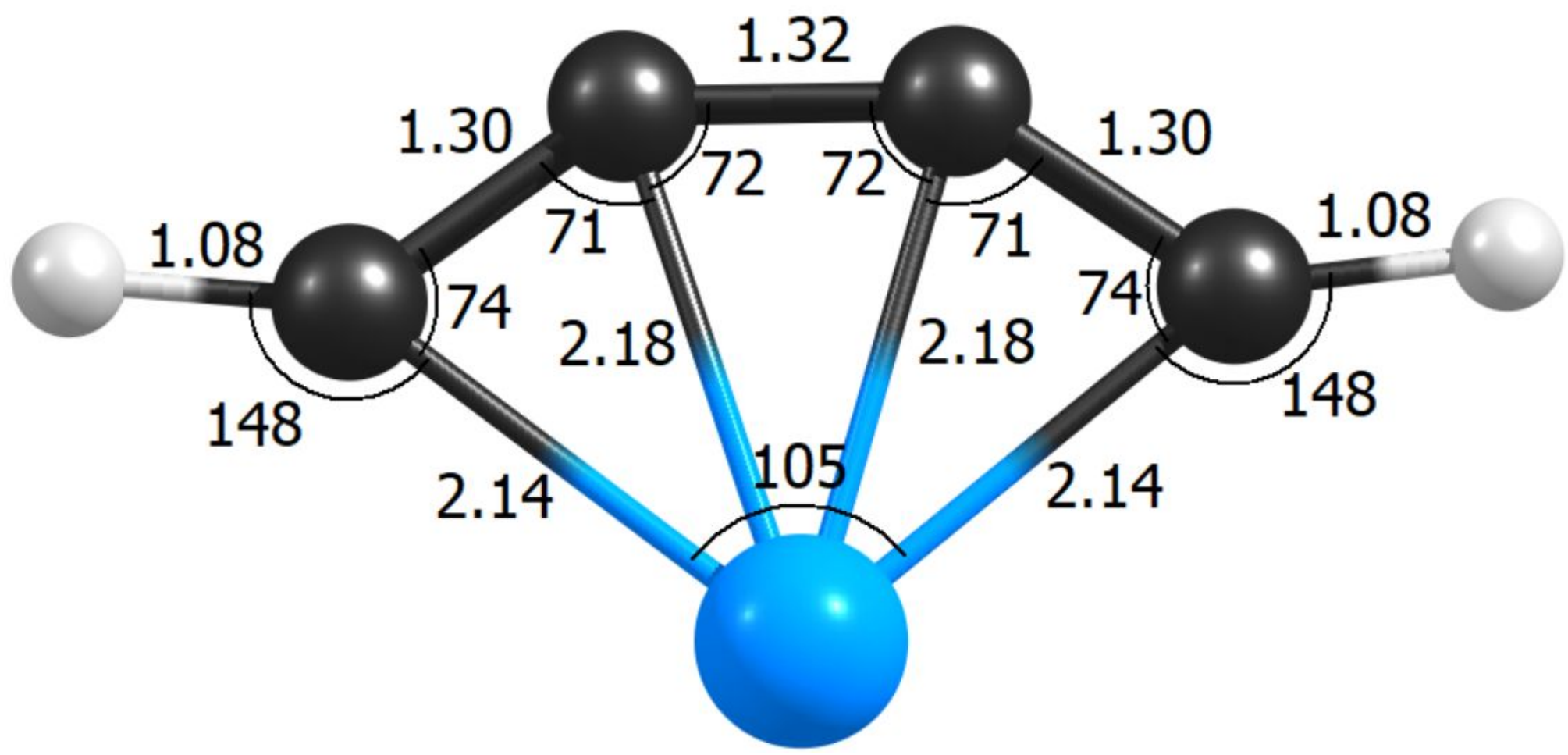

Figure S25. The optimized geometry of isomer 2a-triplet $Ta^+C_4H_2$ followed by its predicted frequencies (cm^{-1}) and IR intensities (km/mol).

Frequency (cm^{-1})	Intensity (km/mol)
257.11	2.6140
389.56	28.6639
438.77	1.8859
535.41	0.0000
552.53	1.5817
614.02	0.3926
718.38	59.0926
862.46	0.0000
924.37	33.1209
932.26	140.5665
1121.53	0.0098
1623.18	12.0180
1914.38	0.0145
3234.17	4.9110
3235.36	133.2061

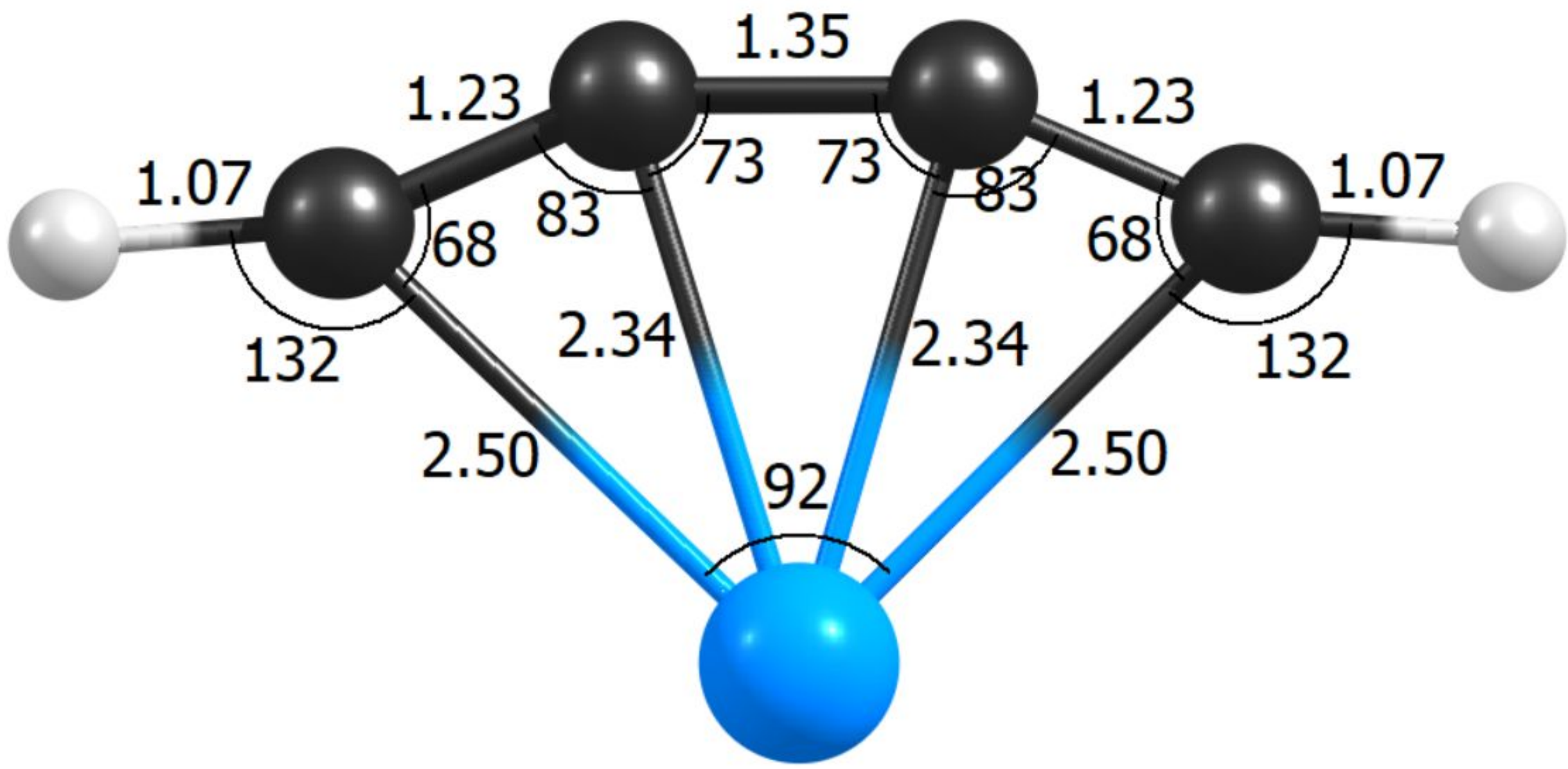

Figure S26. The optimized geometry of isomer 2a-quintet $Ta^{+}C_4H_2$ followed by its predicted frequencies (cm^{-1}) and IR intensities (km/mol).

Frequency (cm^{-1})	Intensity (km/mol)
73.11	18.3286
174.00	5.0025
235.81	1.4503
371.08	0.3817
485.15	0.0000
507.35	3.0459
662.00	63.4405
666.83	0.0008
719.89	48.3009
732.96	17.0762
1007.83	0.1721
1938.28	0.8565
2116.67	22.7570
3334.46	9.4733
3335.36	339.4846

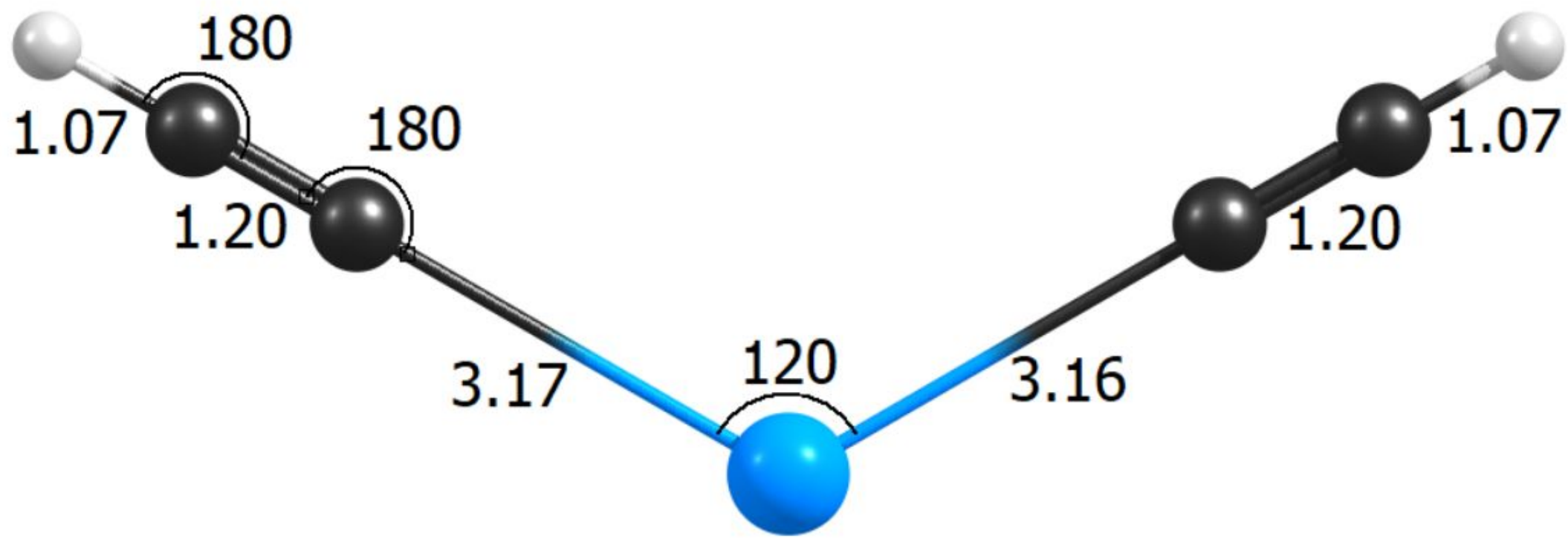

Figure S27. The optimized geometry of isomer 2b-singlet $Ta^{+}C_4H_2$ followed by its predicted frequencies (cm^{-1}) and IR intensities (km/mol).

Frequency (cm^{-1})	Intensity (km/mol)
65.04	5.9094
166.67	59.3197
170.54	9.5561
175.64	0.0000
259.76	29.7160
516.54	82.1576
524.25	26.2272
776.23	38.7764
776.82	22.2794
787.49	54.2428
787.96	0.0000
2025.83	609.7966
2039.67	120.2191
3385.11	155.6171
3385.57	38.7584

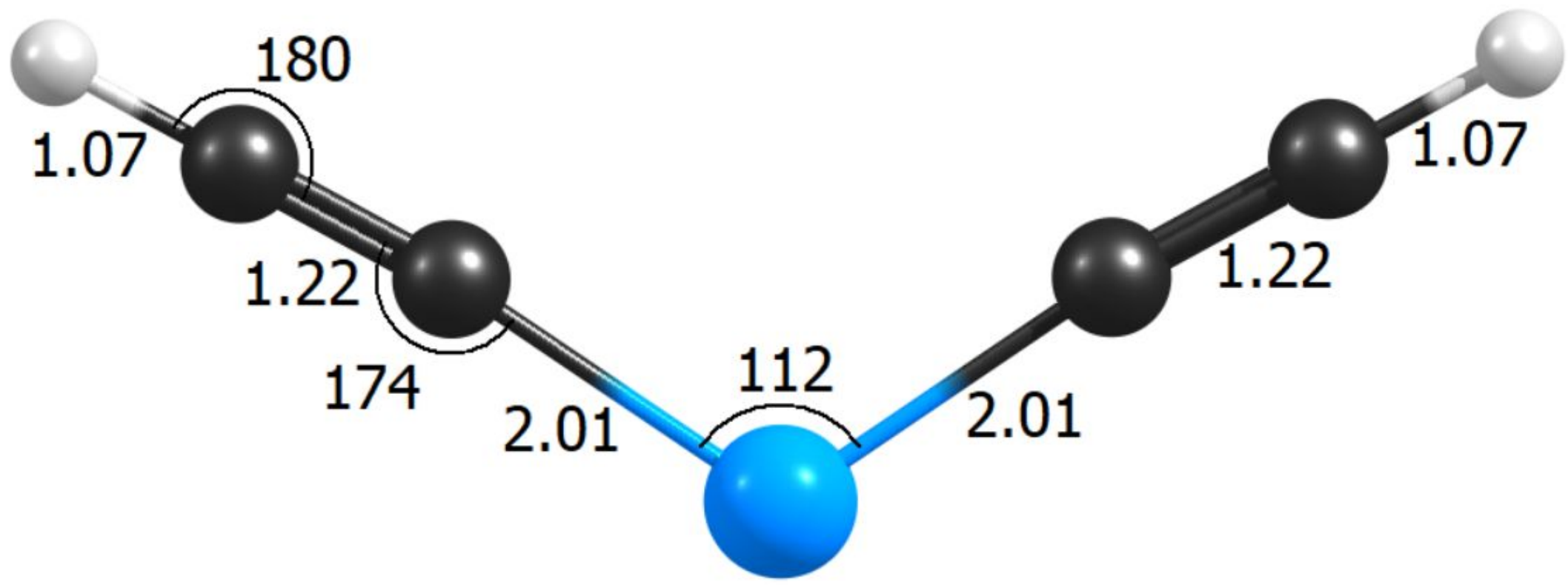

Figure S28. The optimized geometry of isomer 2b-triplet $Ta^+C_4H_2$ followed by its predicted frequencies (cm^{-1}) and IR intensities (km/mol).

Frequency (cm^{-1})	Intensity (km/mol)
64.79	5.2352
167.50	11.4149
172.69	64.7381
180.15	0.0000
248.40	30.2476
512.52	76.4777
522.98	25.5721
774.62	60.4262
774.84	0.0002
788.31	35.3911
788.97	22.6103
2028.13	542.1655
2042.13	121.2929
3386.28	165.7361
3387.06	37.6451

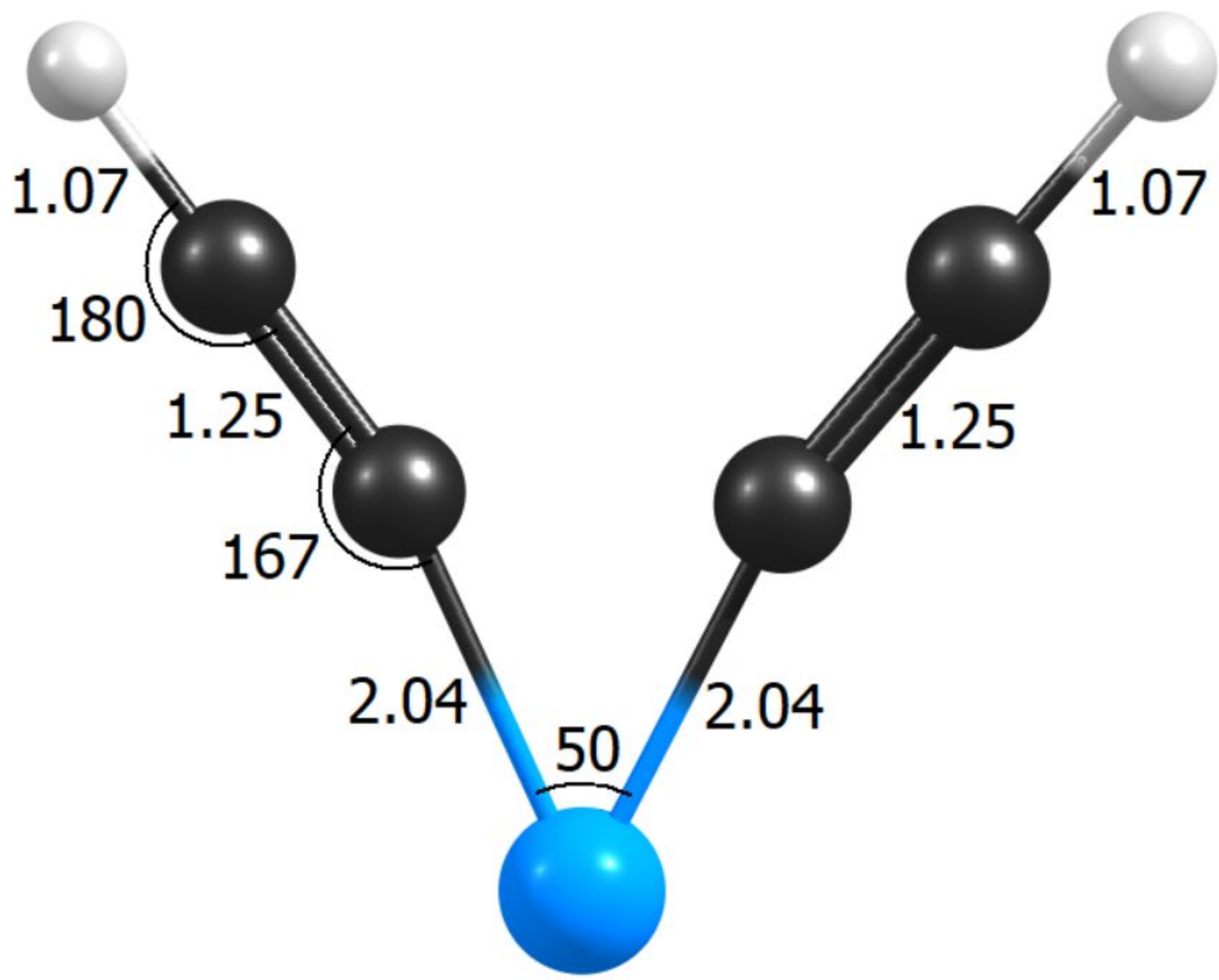

Figure S29. The optimized geometry of isomer 2b-quintet $Ta^+C_4H_2$ followed by its predicted frequencies (cm^{-1}) and IR intensities (km/mol).

Frequency (cm^{-1})	Intensity (km/mol)
160.67	0.2176
244.50	2.4640
260.53	42.7637
273.36	26.1413
283.48	0.0000
433.64	8.1947
483.76	0.0758
577.86	21.4352
591.72	28.0211
652.83	12.4776
652.86	74.2191
1715.38	0.1901
1796.19	0.2130
3374.19	173.2605
3378.45	218.7731

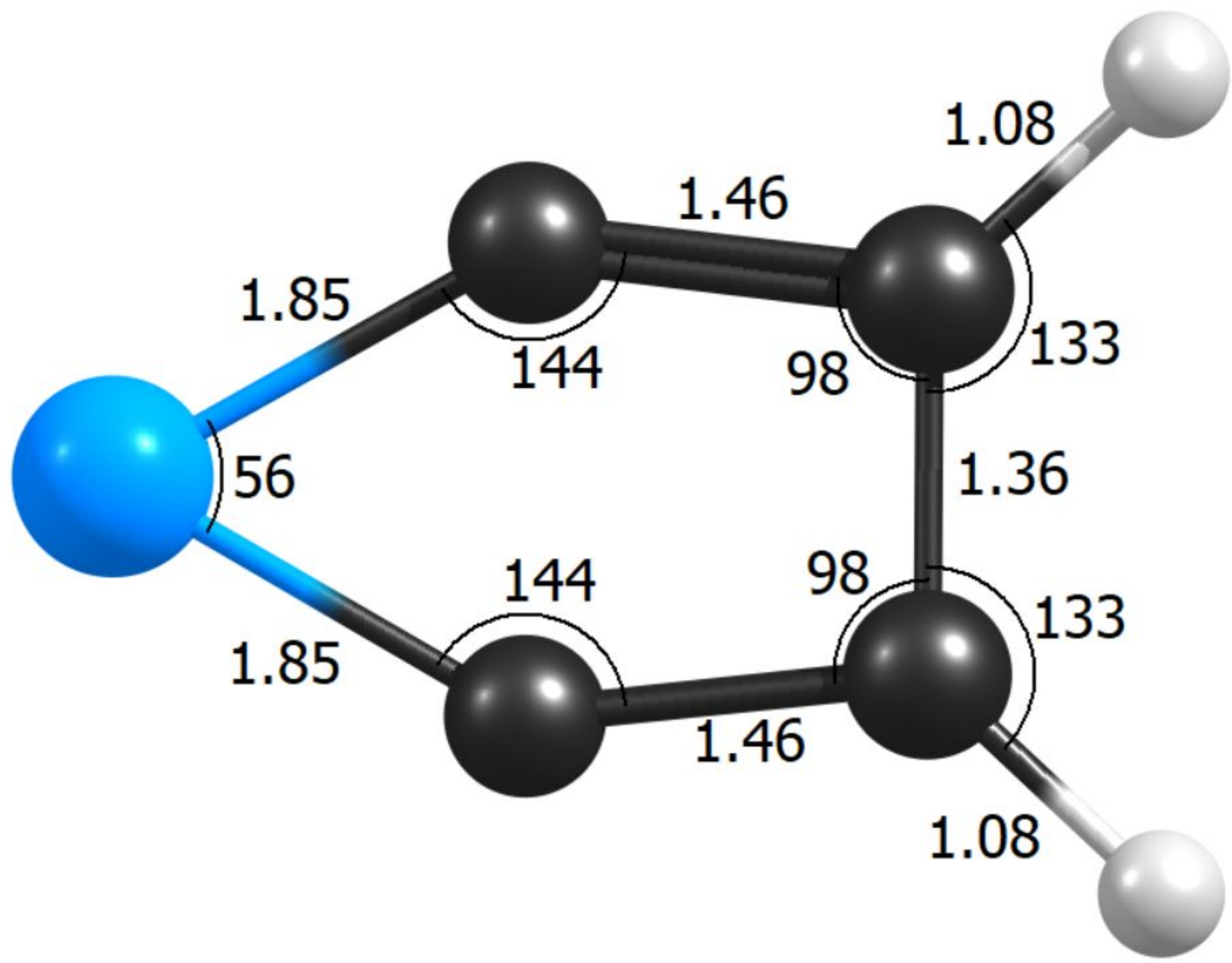

Figure S30. The optimized geometry of isomer 2c-singlet $Ta^+C_4H_2$ followed by its predicted frequencies (cm^{-1}) and IR intensities (km/mol).

Frequency (cm^{-1})	Intensity (km/mol)
293.69	64.2879
471.14	1.9862
538.18	0.0000
589.11	37.1245
732.60	20.1186
733.63	35.2156
797.05	0.0387
928.01	0.0000
1088.73	5.6620
1165.26	5.0321
1168.00	4.4846
1309.17	15.9782
1475.30	14.8167
3218.41	7.3569
3239.07	12.6656

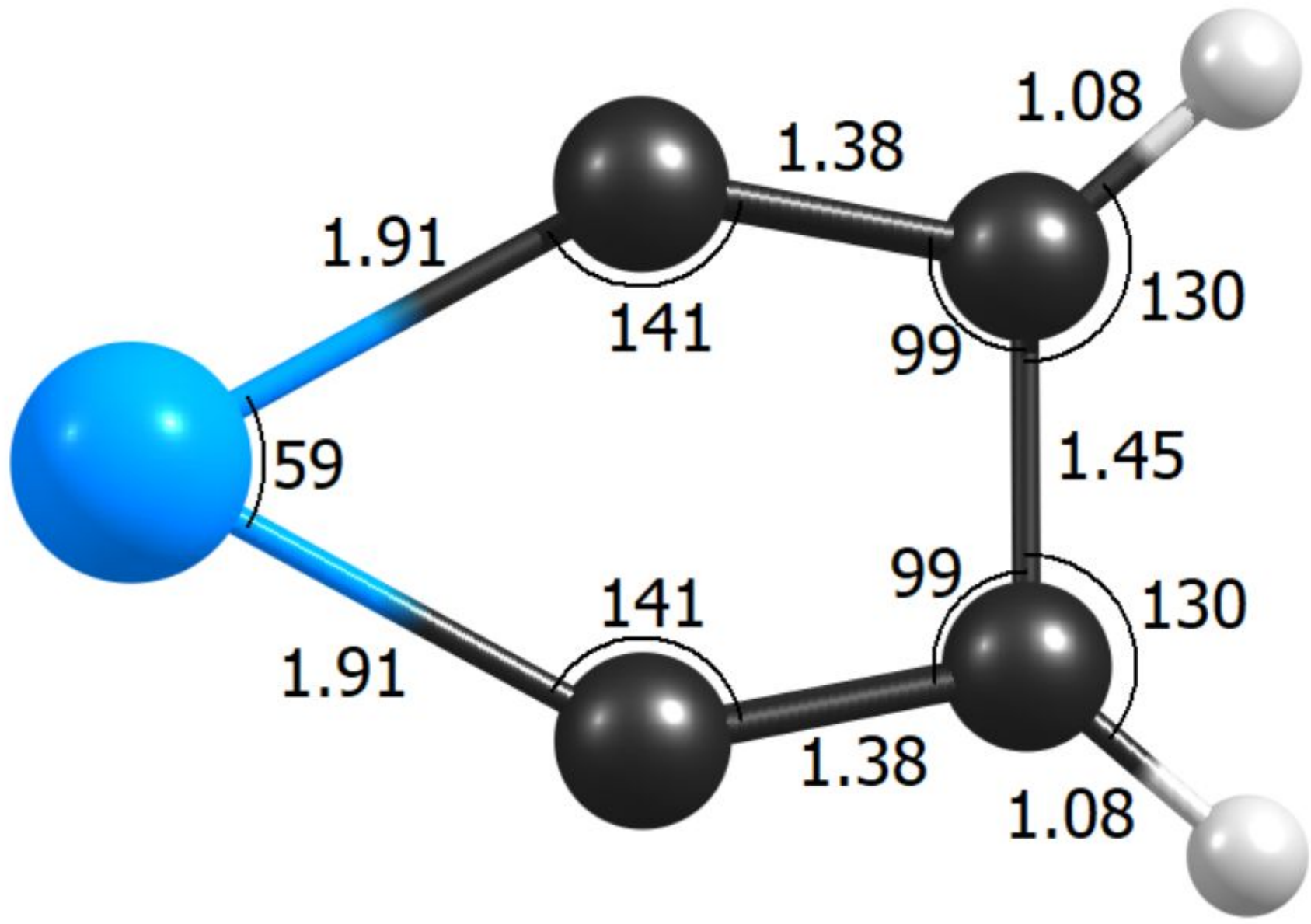

Figure S31. The optimized geometry of isomer 2c-triplet $Ta^+C_4H_2$ followed by its predicted frequencies (cm^{-1}) and IR intensities (km/mol).

Frequency (cm^{-1})	Intensity (km/mol)
215.03	45.7807
434.29	0.0000
484.90	0.1756
486.91	0.4748
666.34	72.9702
726.29	0.0044
760.63	20.0297
906.97	0.0000
1001.63	9.1427
1130.77	4.1953
1244.99	7.9867
1339.50	46.2659
1383.48	10.3712
3196.14	7.9048
3209.78	19.3315

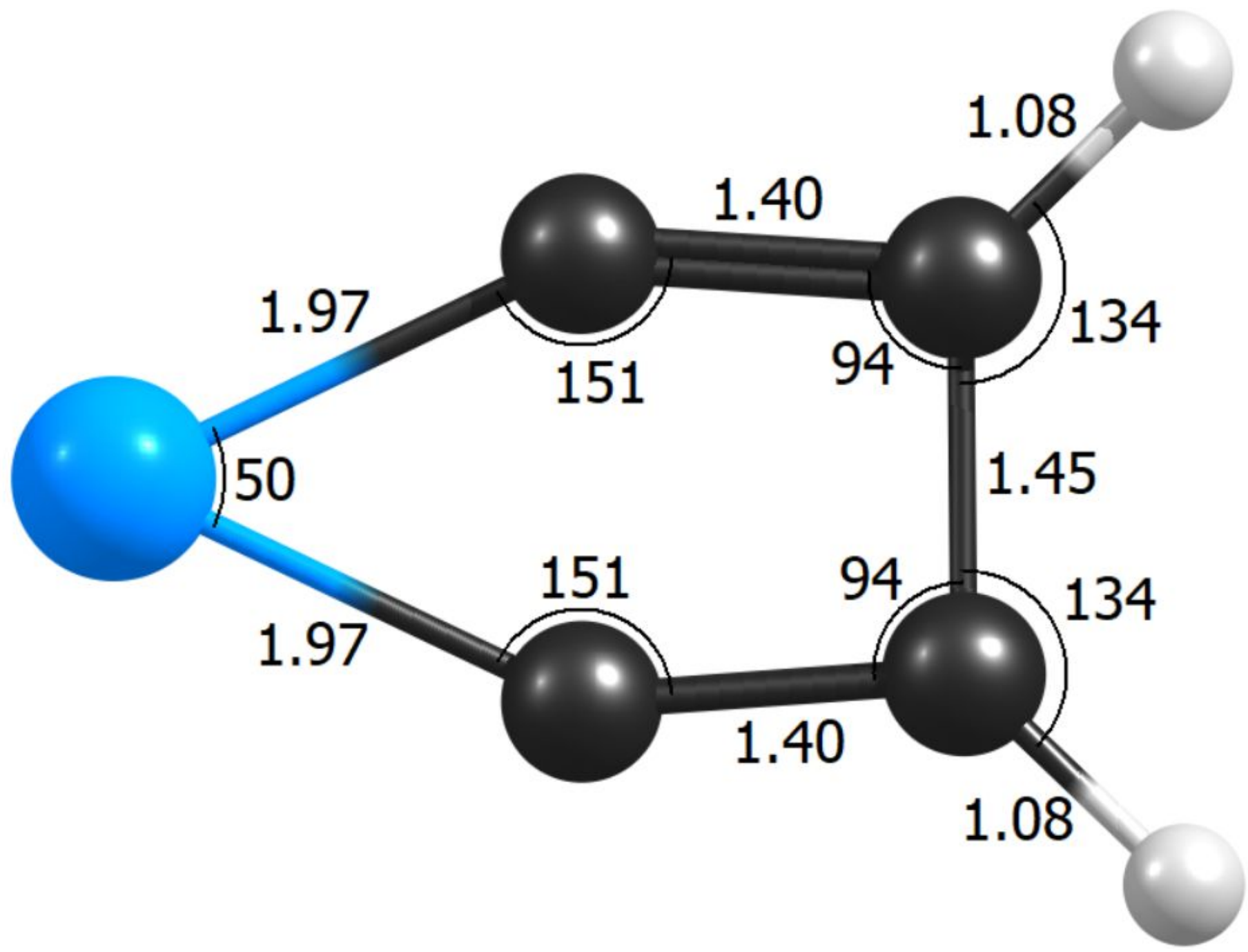

Figure S32. The optimized geometry of isomer 2c-quintet $Ta^+C_4H_2$ followed by its predicted frequencies (cm^{-1}) and IR intensities (km/mol).

Frequency (cm^{-1})	Intensity (km/mol)
225.60	0.0000
253.71	22.1332
442.40	4.9891
454.69	9.9188
619.08	34.9261
660.26	10.2557
759.83	31.3660
771.35	0.0000
1045.10	0.7340
1153.02	17.5466
1256.53	37.1270
1333.82	1.9563
1400.08	44.0611
3204.53	12.6946
3218.70	18.6280

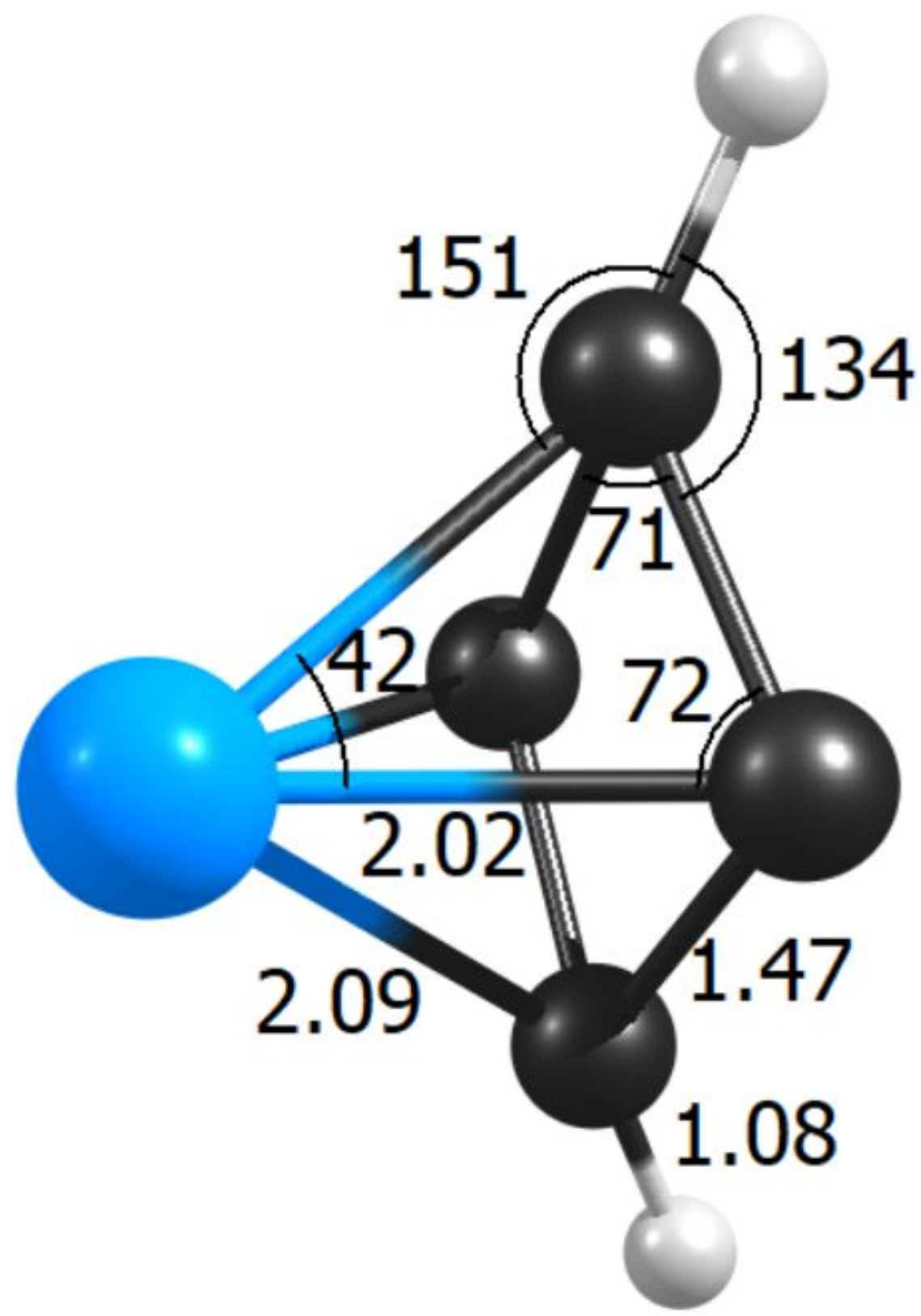

Figure S33. The optimized geometry of the 2d-singlet $Ta^+C_4H_2$ followed by its predicted frequencies (cm^{-1}) and IR intensities (km/mol).

Frequency (cm^{-1})	Intensity (km/mol)
277.37	35.1149
385.29	13.5782
529.09	1.9441
588.58	3.9407
731.98	21.1632
735.23	14.6583
806.90	0.0001
851.15	121.7415
914.99	0.0355
978.61	9.9171
1039.22	0.0000
1141.44	3.1518
1287.91	3.7869
3224.04	41.2404
3226.99	6.3940

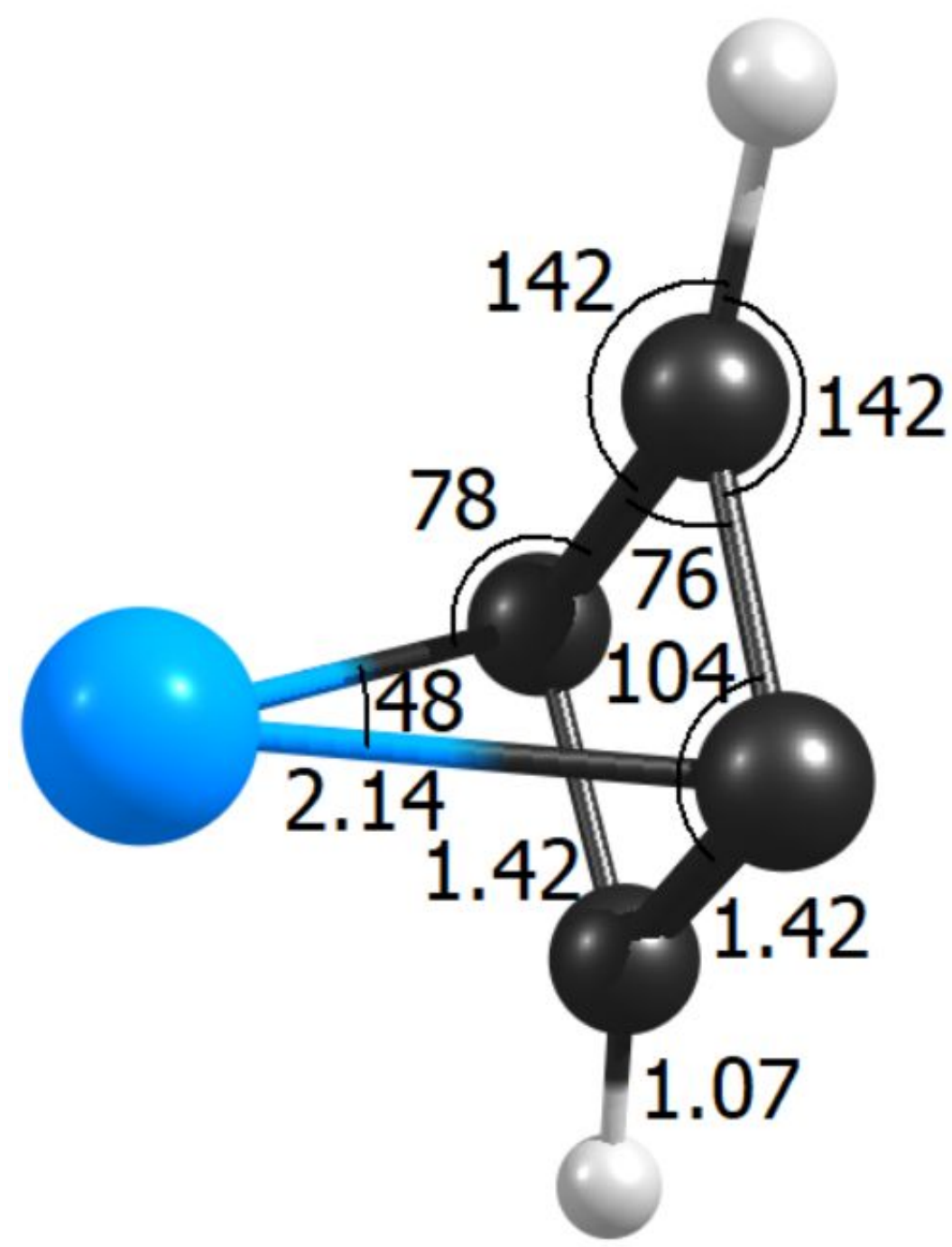

Figure S34. The optimized geometry of the 2d-triplet $Ta^{+}C_4H_2$ followed by its predicted frequencies (cm^{-1}) and IR intensities (km/mol).

Frequency (cm^{-1})	Intensity (km/mol)
163.53	16.2424
346.59	0.4933
463.56	9.7774
480.45	10.0696
573.64	12.0223
708.66	9.2634
804.31	62.7816
877.55	2.3890
885.58	0.0000
1068.96	0.0000
1095.43	0.7174
1283.47	14.8701
1361.44	0.1475
3309.76	170.3106
3315.37	26.9379

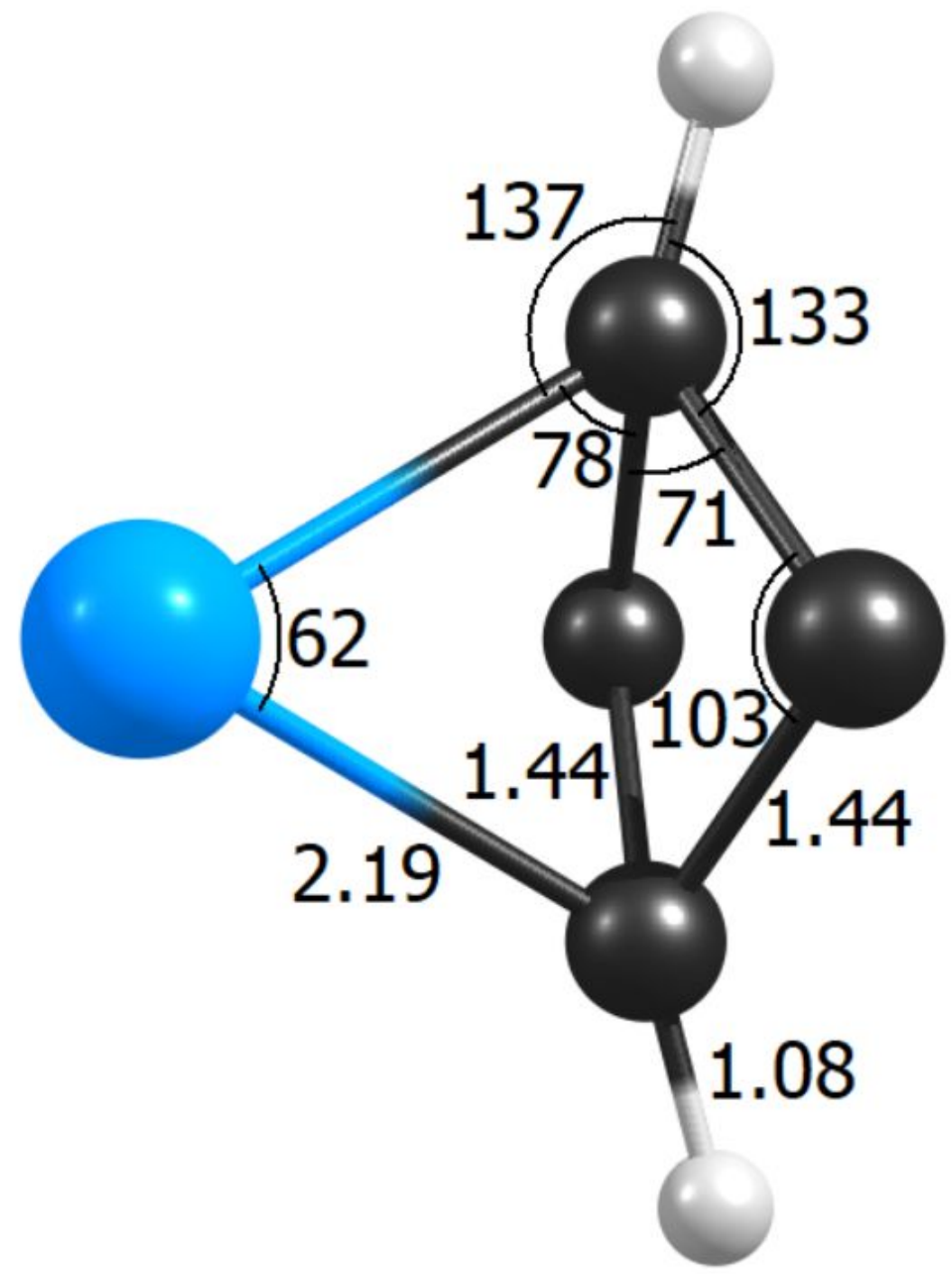

Figure S35. The optimized geometry of the 2d-quintet $Ta^+C_4H_2$ followed by its predicted frequencies (cm^{-1}) and IR intensities (km/mol).

Frequency (cm^{-1})	Intensity (km/mol)
253.61	0.1505
290.12	13.6024
390.06	1.7026
550.38	52.5974
727.44	67.7784
736.46	0.0000
763.02	2.5140
774.94	75.6511
944.34	1.5484
1038.45	0.0000
1081.71	5.3093
1245.17	6.4106
1325.23	1.2493
3249.37	104.9236
3253.19	14.6787

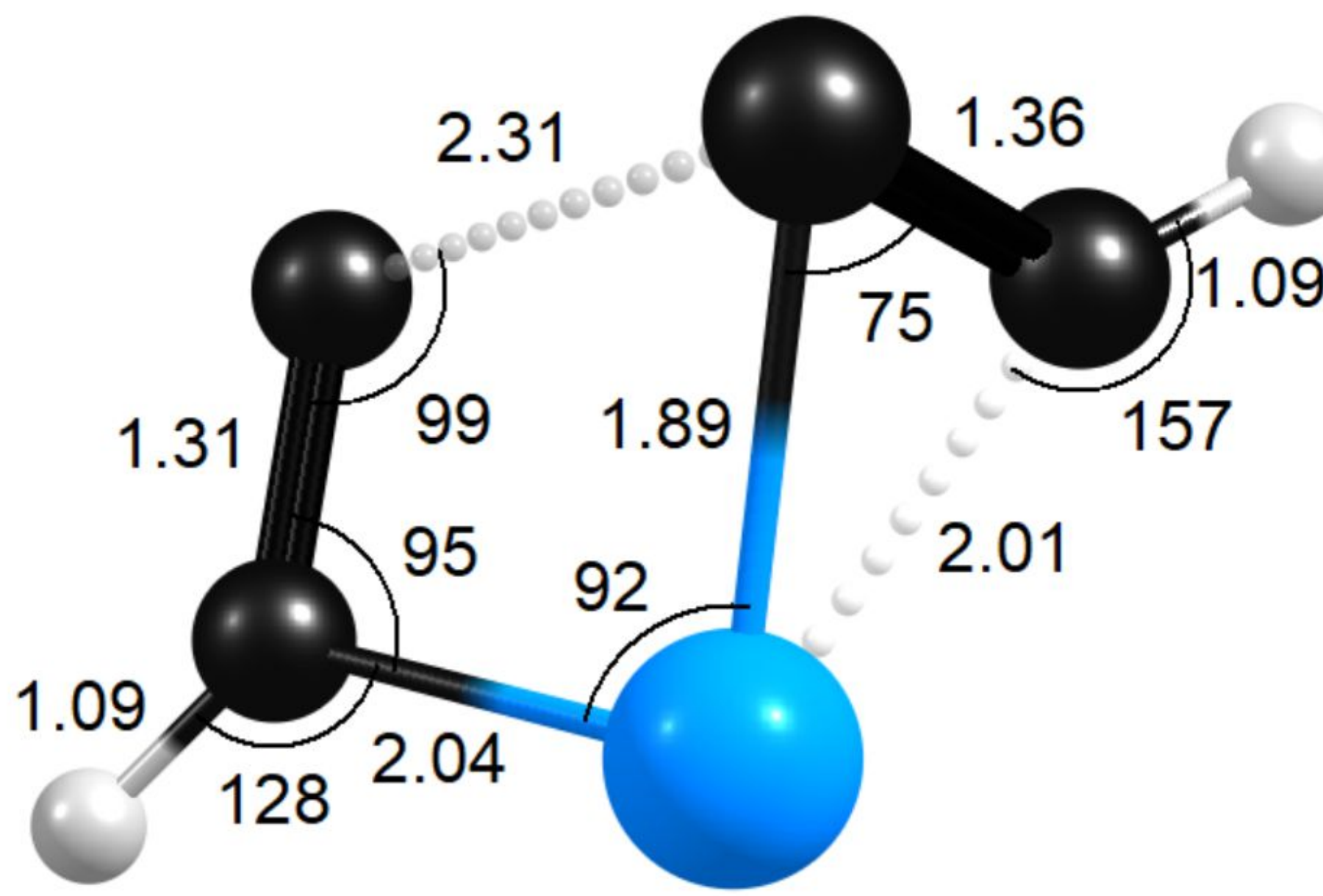

Figure S36. The optimized geometry of TS1-singlet $Ta^{+}C_4H_2$ followed by its predicted frequencies (cm^{-1}) and IR intensities (km/mol).

Frequency (cm^{-1})	Intensity (km/mol)
-214.136064	4.9374
145.167168	5.2529
273.959328	4.9796
369.337632	1.4755
505.464768	21.2583
587.025984	12.5848
611.012352	62.9164
704.06352	12.8359
735.941664	48.0658
785.2152	29.2161
905.894976	75.2597
1313.434272	3.2327
1492.47168	65.2525
3046.243872	144.2582
3049.229088	16.1223

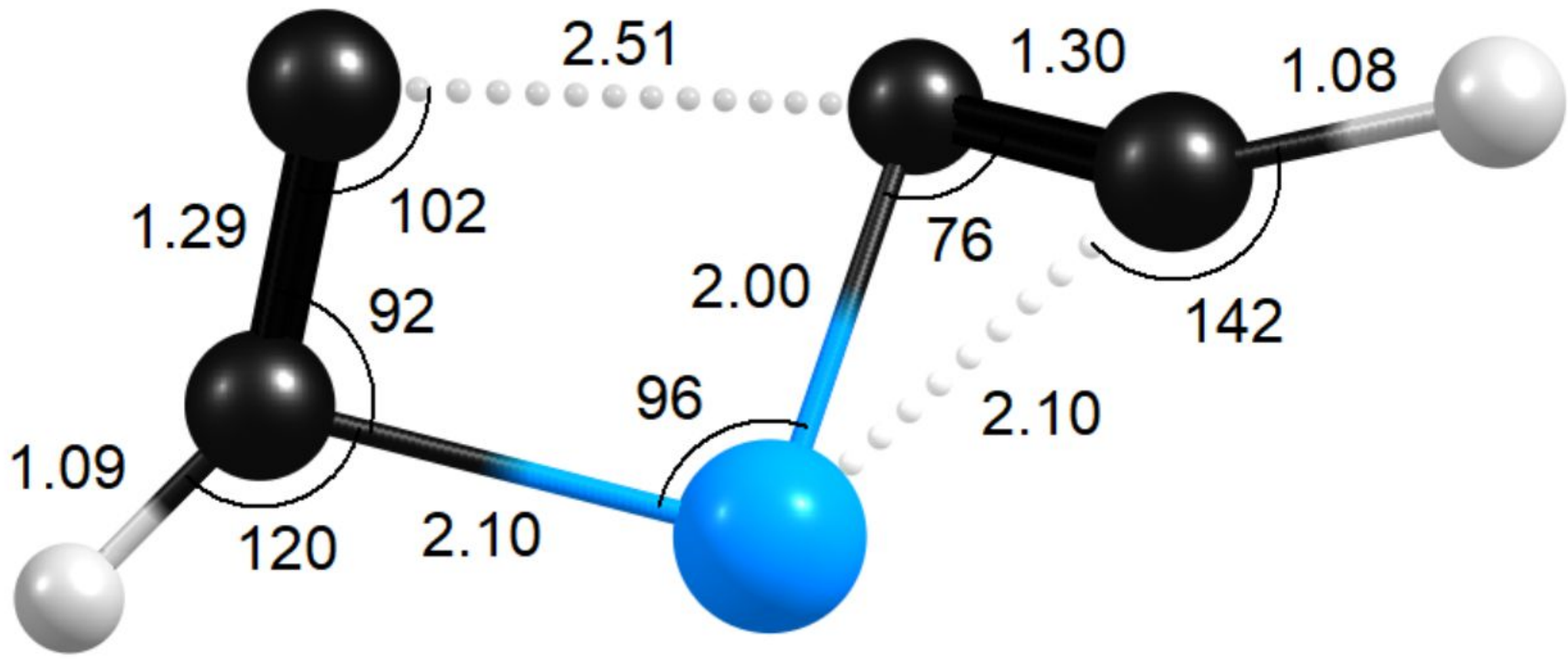

Figure S37. The optimized geometry of TS1-triplet $Ta^{+}C_4H_2$ followed by its predicted frequencies (cm^{-1}) and IR intensities (km/mol).

Frequency (cm^{-1})	Intensity (km/mol)
-264.360288	1.5558
156.316608	8.5438
174.855936	8.5846
266.78592	5.8037
405.450048	15.8312
457.321632	4.5607
549.004032	46.2829
590.230848	9.008
707.014176	25.8593
775.1256	82.6243
781.08	56.2213
1519.712736	11.1075
1604.116128	96.9368
3054.132672	122.0574
3088.500672	85.399

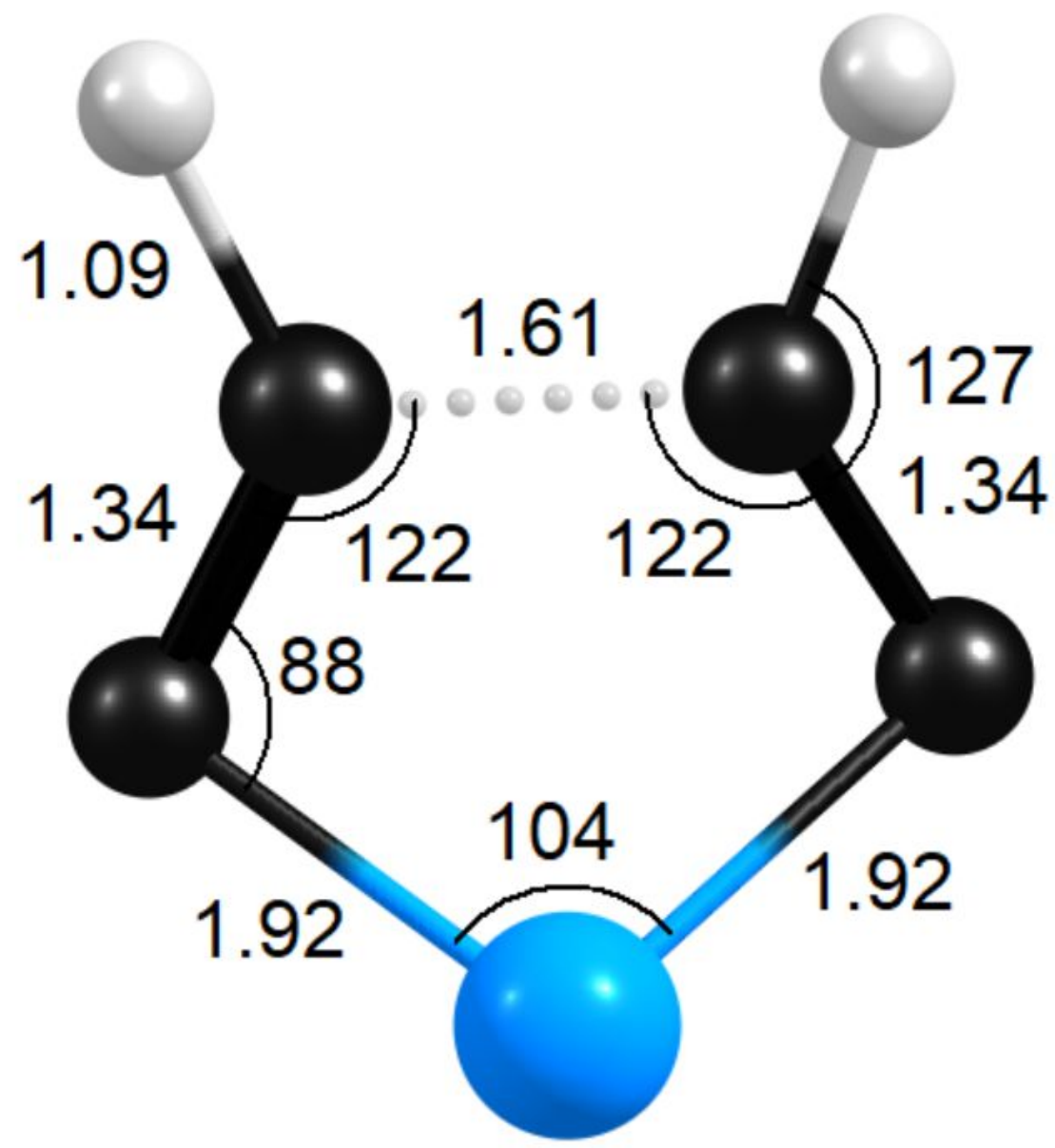

Figure S38. The optimized geometry of TS2-singlet $Ta^{+}C_4H_2$ followed by its predicted frequencies (cm^{-1}) and IR intensities (km/mol).

Frequency (cm^{-1})	Intensity (km/mol)
-476.902848	79.2485
81.988608	0.395
263.808576	15.7856
493.3584	0.238
507.006624	28.7227
660.20304	2.3911
725.388864	43.7459
766.755936	21.5676
866.971584	7.7844
1087.5456	21.3339
1148.738784	31.8544
1350.590784	4.5629
1475.405856	0.0636
2957.137824	3.793
2969.4552	9.0248

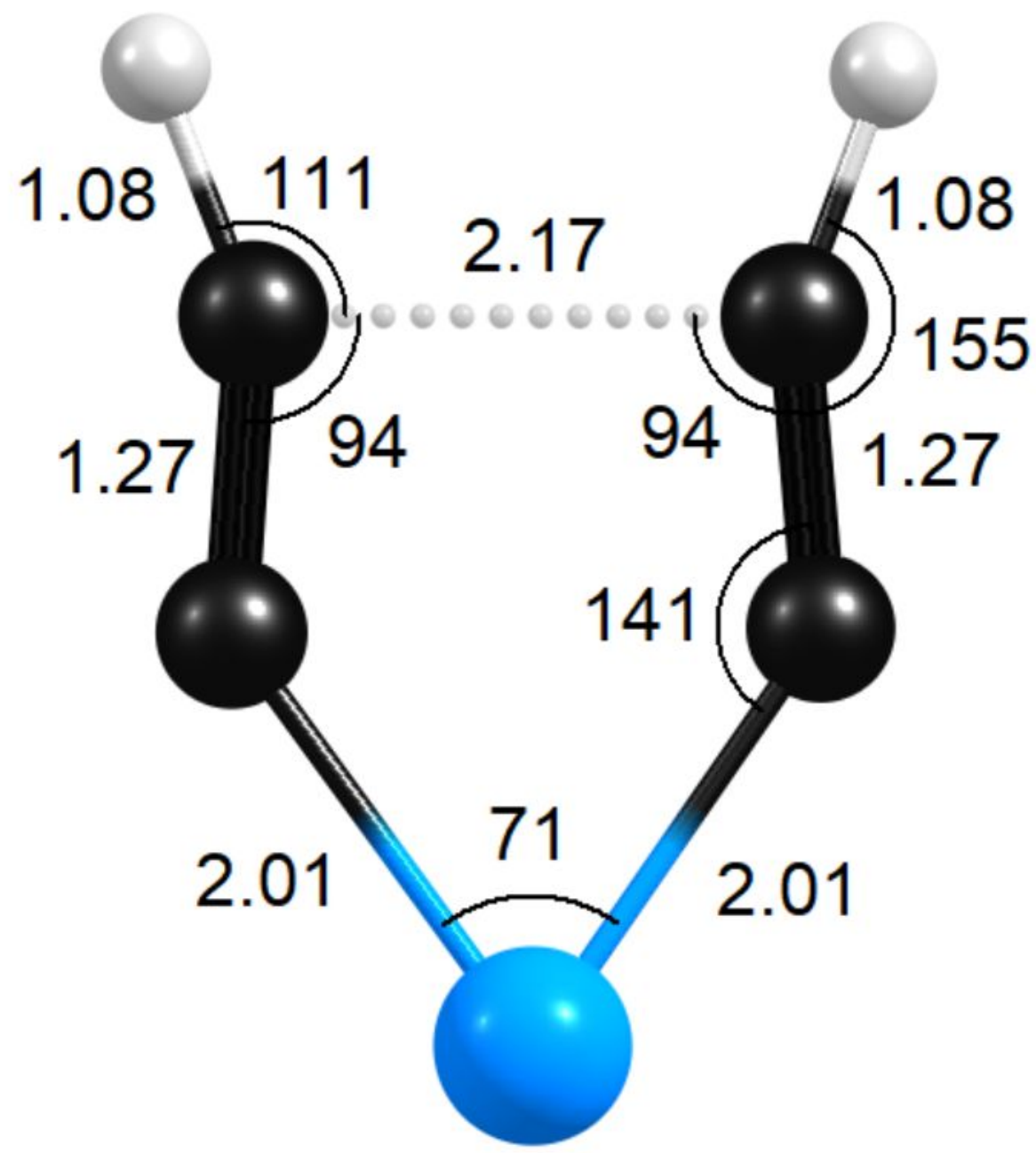

Figure S39. The optimized geometry of TS2-triplet $Ta^{+}C_4H_2$ followed by its predicted frequencies (cm^{-1}) and IR intensities (km/mol).

Frequency (cm^{-1})	Intensity (km/mol)
-497.41968	32.6007
211.205184	49.5945
222.096384	0.0043
307.495488	19.1989
326.246784	11.6761
424.163424	0.4905
482.798688	35.2476
642.562848	71.3558
644.472768	0.0538
696.177024	62.1782
792.122592	15.9768
1566.871968	1.1163
1603.708704	1.4891
3163.774944	40.2119
3172.112832	209.0489

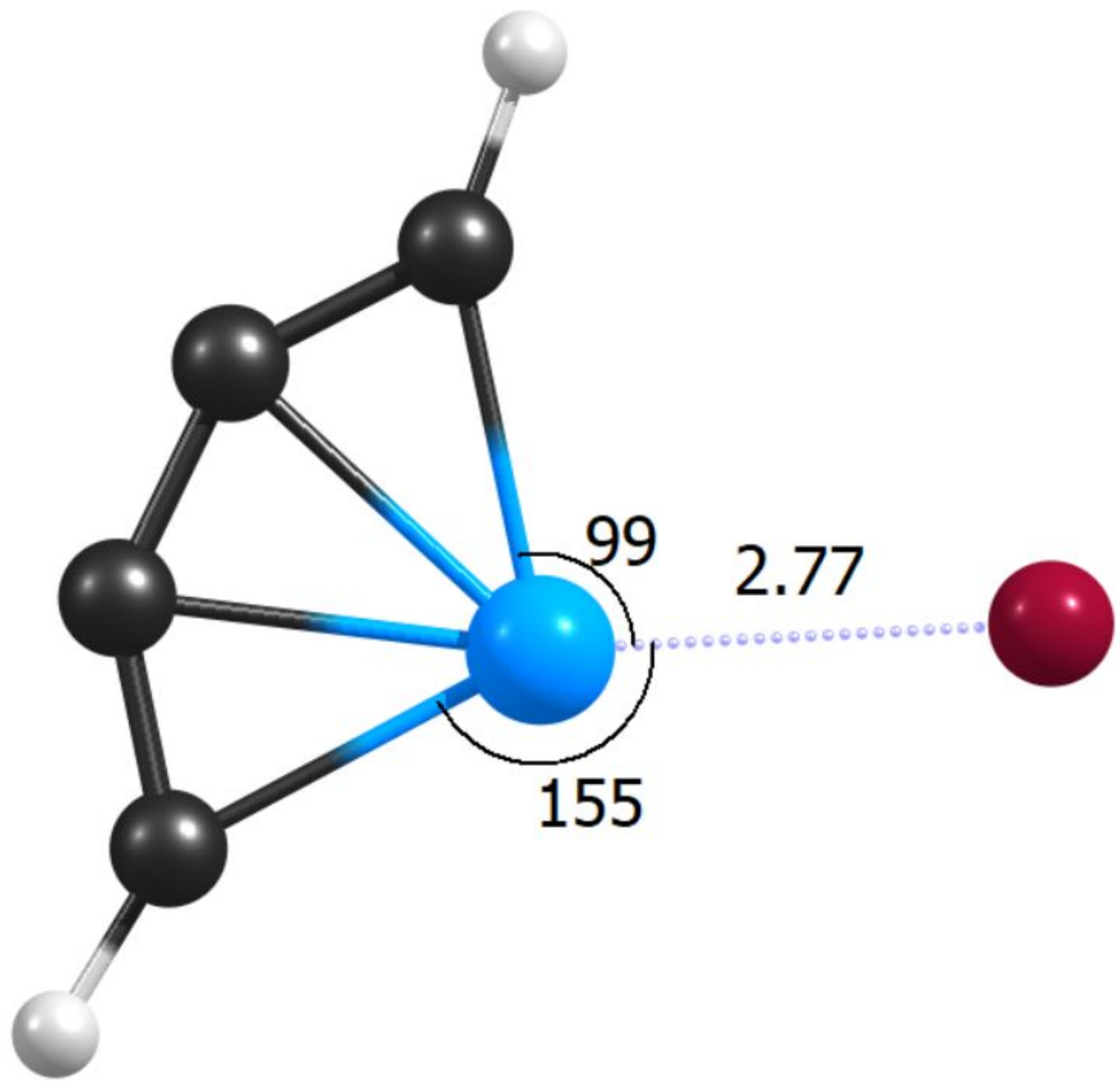

Figure S40. The optimized geometry of isomer 2aI-singlet $Ta^{+}C_4H_2Ar$ followed by its predicted frequencies (cm^{-1}) and IR intensities (km/mol).

Frequency (cm^{-1})	Intensity (km/mol)
45.92	0.3013
68.02	0.8902
123.16	8.4699
188.51	0.0849
419.28	25.1673
438.16	2.2241
536.74	0.0105
597.47	1.6806
632.55	0.2150
713.81	61.5381
866.91	0.4243
920.02	62.0782
926.21	96.3088
1119.81	0.0760
1634.68	8.3187
1927.94	0.1069
3239.92	11.5156
3241.52	120.9174

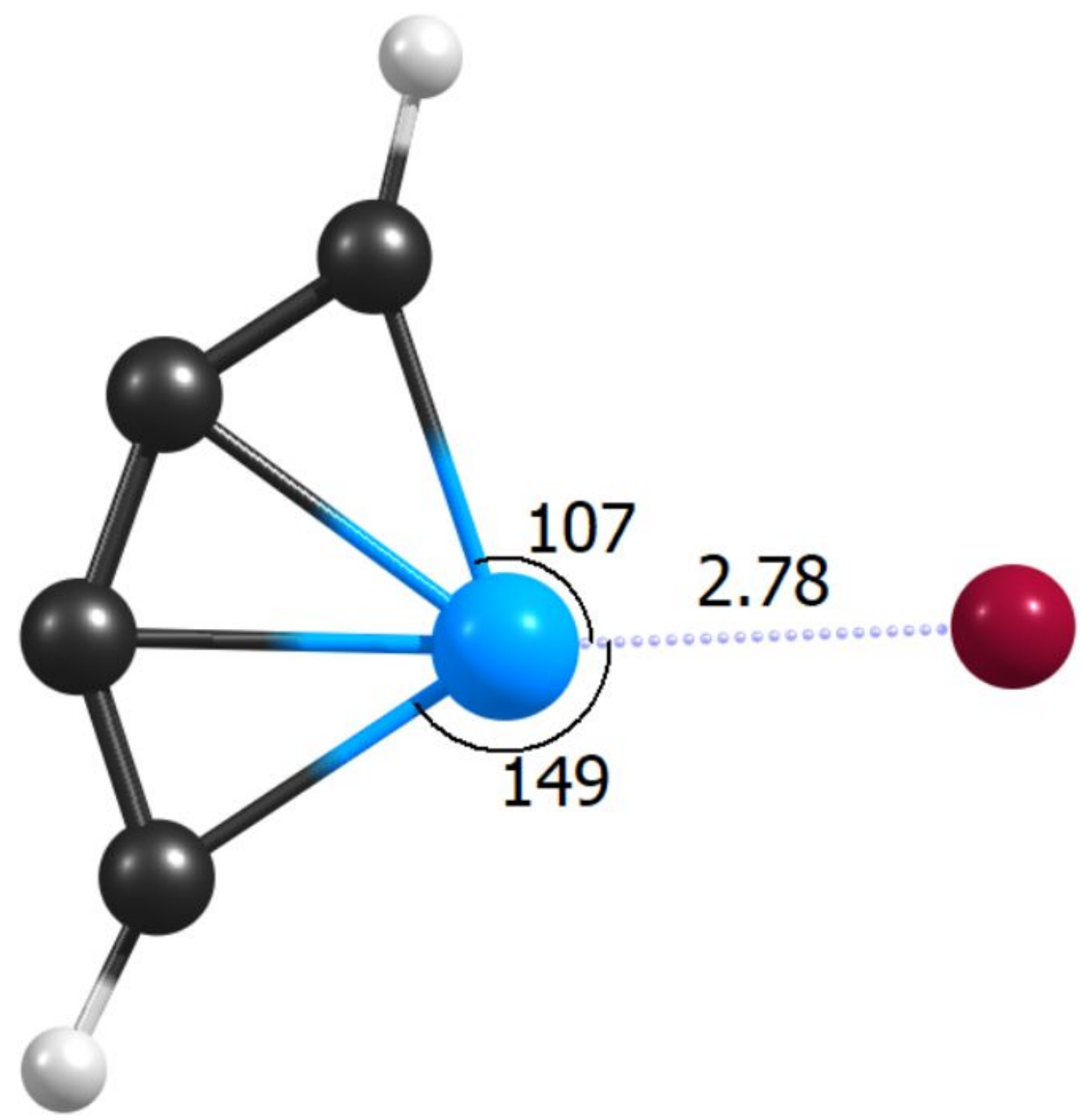

Figure S41. The optimized geometry of isomer 2aI-triplet $Ta^+C_4H_2Ar$ followed by its predicted frequencies (cm^{-1}) and IR intensities (km/mol).

Frequency (cm^{-1})	Intensity (km/mol)	Frequency (cm^{-1})	Intensity (km/mol)
31.32	0.0300	716.86	60.5217
71.10	0.7340	861.48	0.3044
125.87	7.1781	924.64	29.6366
253.99	1.0516	935.03	138.7654
386.91	26.7222	1120.95	0.5158
432.54	7.0596	1623.78	15.8138
536.60	0.0140	1915.74	0.2813
548.93	3.4448	3235.70	11.8281
615.42	0.6604	3237.37	104.2472

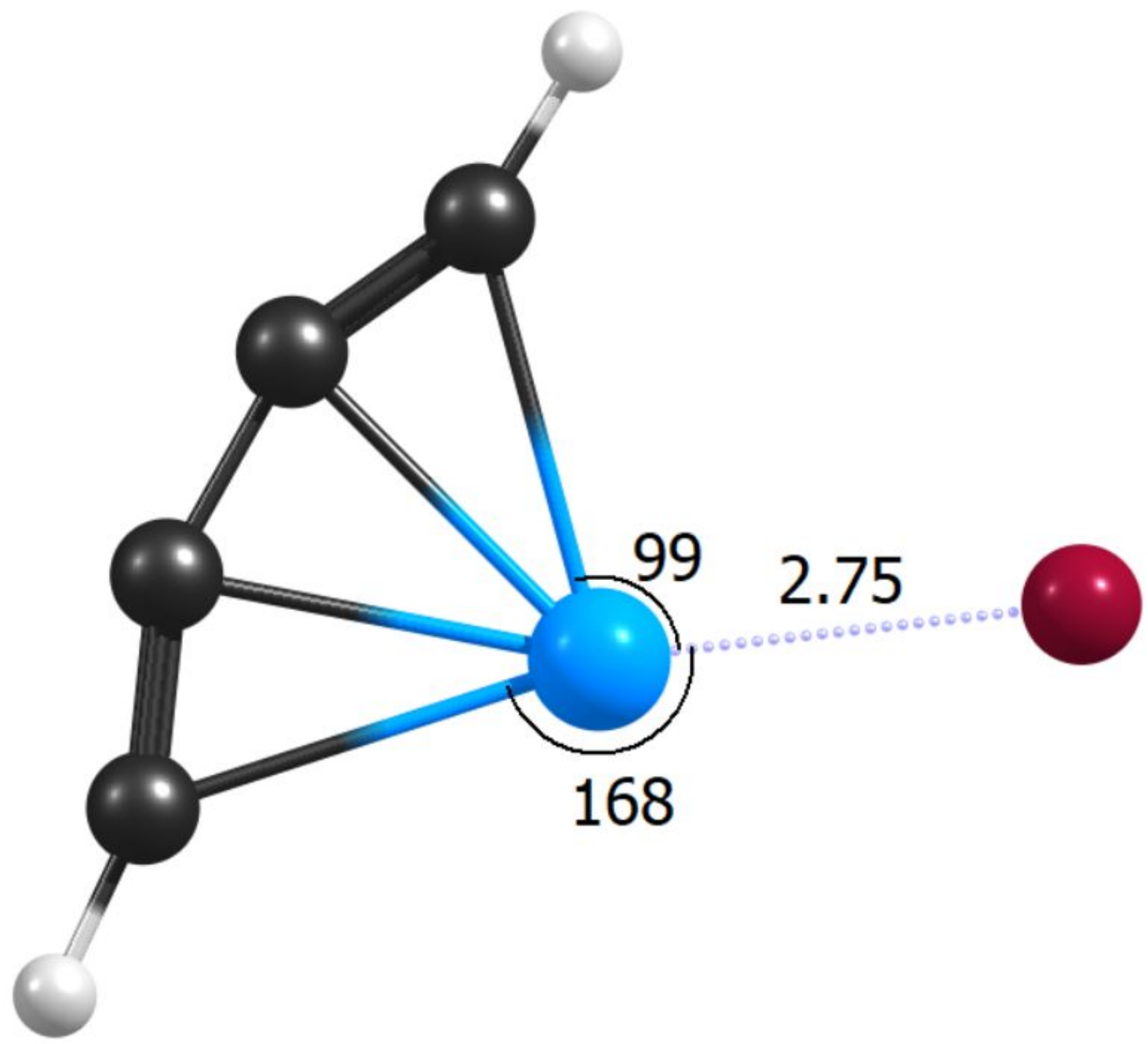

Figure S42. The optimized geometry of isomer 2aI-quintet $Ta^+C_4H_2Ar$ followed by its predicted frequencies (cm^{-1}) and IR intensities (km/mol).

Frequency (cm^{-1})	Intensity (km/mol)	Frequency (cm^{-1})	Intensity (km/mol)
40.17	0.1989	653.34	58.3956
66.69	1.2216	661.99	4.2075
83.11	16.1605	716.61	49.4846
124.96	7.7223	730.57	19.7582
169.02	4.1828	1006.80	0.7970
241.20	0.5230	1933.08	0.1413
372.48	0.1302	2113.93	29.3565
484.59	0.0032	3332.22	134.0153
511.21	3.6834	3346.28	188.5779

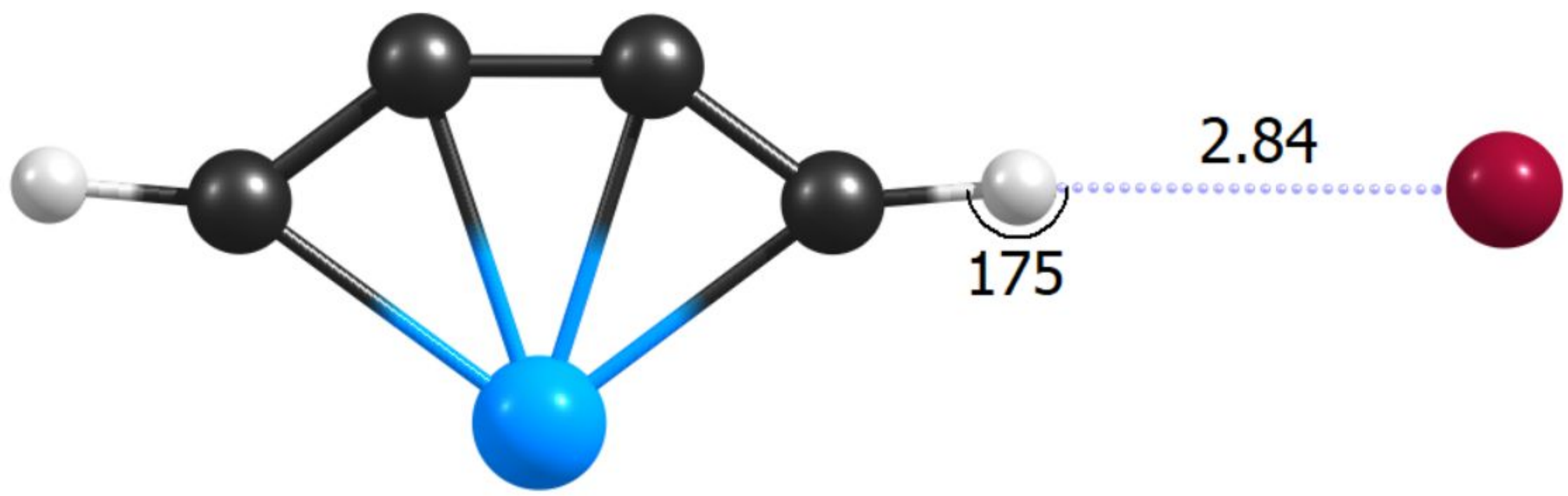

Figure S43. The optimized geometry of isomer 2aII-singlet $Ta^+C_4H_2Ar$ followed by its predicted frequencies (cm^{-1}) and IR intensities (km/mol).

Frequency (cm^{-1})	Intensity (km/mol)	Frequency (cm^{-1})	Intensity (km/mol)
11.21	0.0752	721.65	55.7049
17.82	0.0024	875.47	0.0142
32.91	1.5287	922.28	96.1612
205.85	0.5115	927.34	71.7761
421.91	32.5124	1122.07	0.1199
444.21	0.7047	1633.02	3.2524
535.64	0.0021	1925.49	0.5867
600.27	0.2681	3226.95	154.3967
630.76	0.3909	3237.94	87.5524

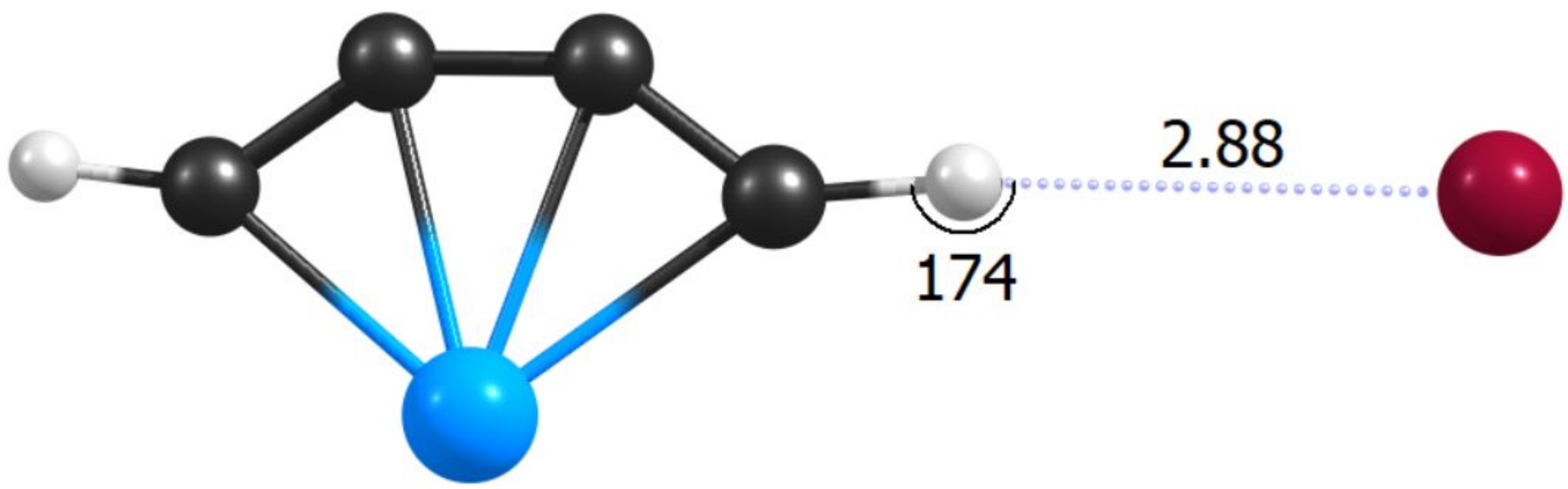

Figure S44. The optimized geometry of isomer 2aII-triplet $Ta^+C_4H_2Ar$ followed by its predicted frequencies (cm^{-1}) and IR intensities (km/mol).

Frequency (cm^{-1})	Intensity (km/mol)	Frequency (cm^{-1})	Intensity (km/mol)
10.03	0.1723	723.84	53.1445
16.36	0.0003	867.54	0.0153
30.96	1.2456	926.58	34.3772
258.43	2.0768	935.27	148.8950
390.07	31.9395	1121.63	0.0543
438.90	2.1588	1622.82	9.3083
535.80	0.0040	1914.06	0.1671
553.54	1.4384	3226.09	126.1060
614.43	0.7482	3234.85	79.0184

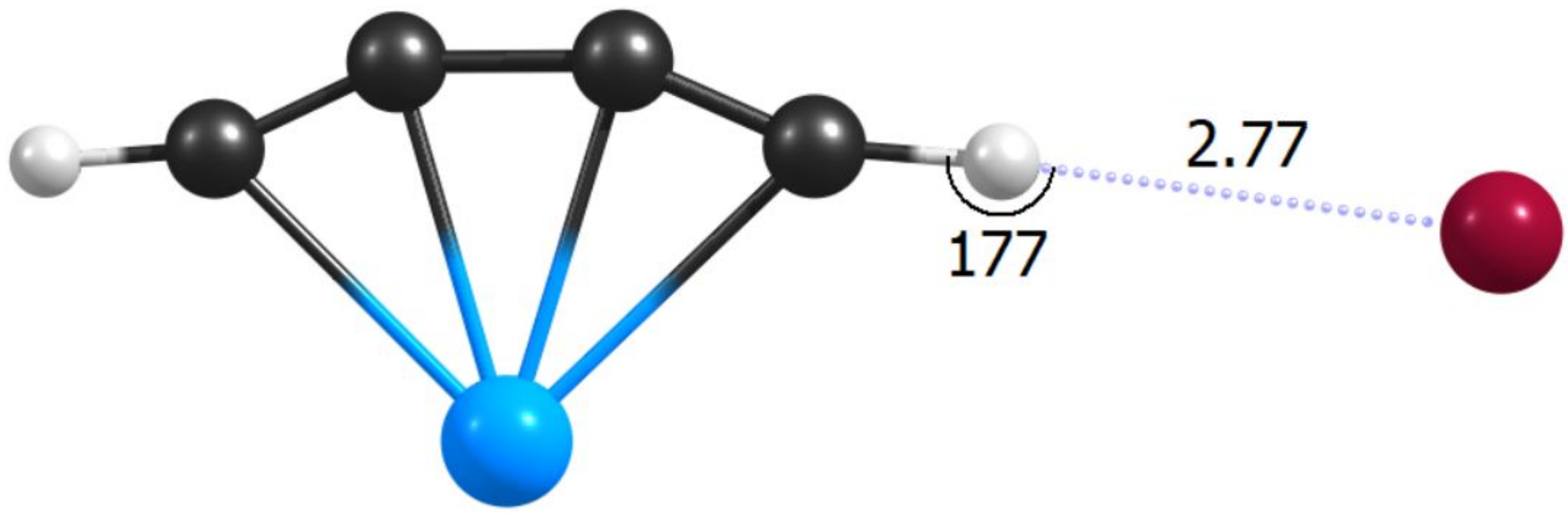

Figure S45. The optimized geometry of isomer 2bI-singlet $Ta^+C_4H_2Ar$ followed by its predicted frequencies (cm^{-1}) and IR intensities (km/mol).

Frequency (cm^{-1})	Intensity (km/mol)	Frequency (cm^{-1})	Intensity (km/mol)
10.04	0.0514	664.06	41.3798
18.27	0.0017	677.14	14.3317
37.52	0.9227	723.50	51.9527
76.80	21.7922	738.25	16.8940
176.77	3.9227	1008.06	0.5101
236.25	1.1532	1937.67	3.1681
371.92	0.4991	2116.11	23.1986
485.30	0.0064	3316.60	304.7885
507.42	3.4415	3333.56	181.5741

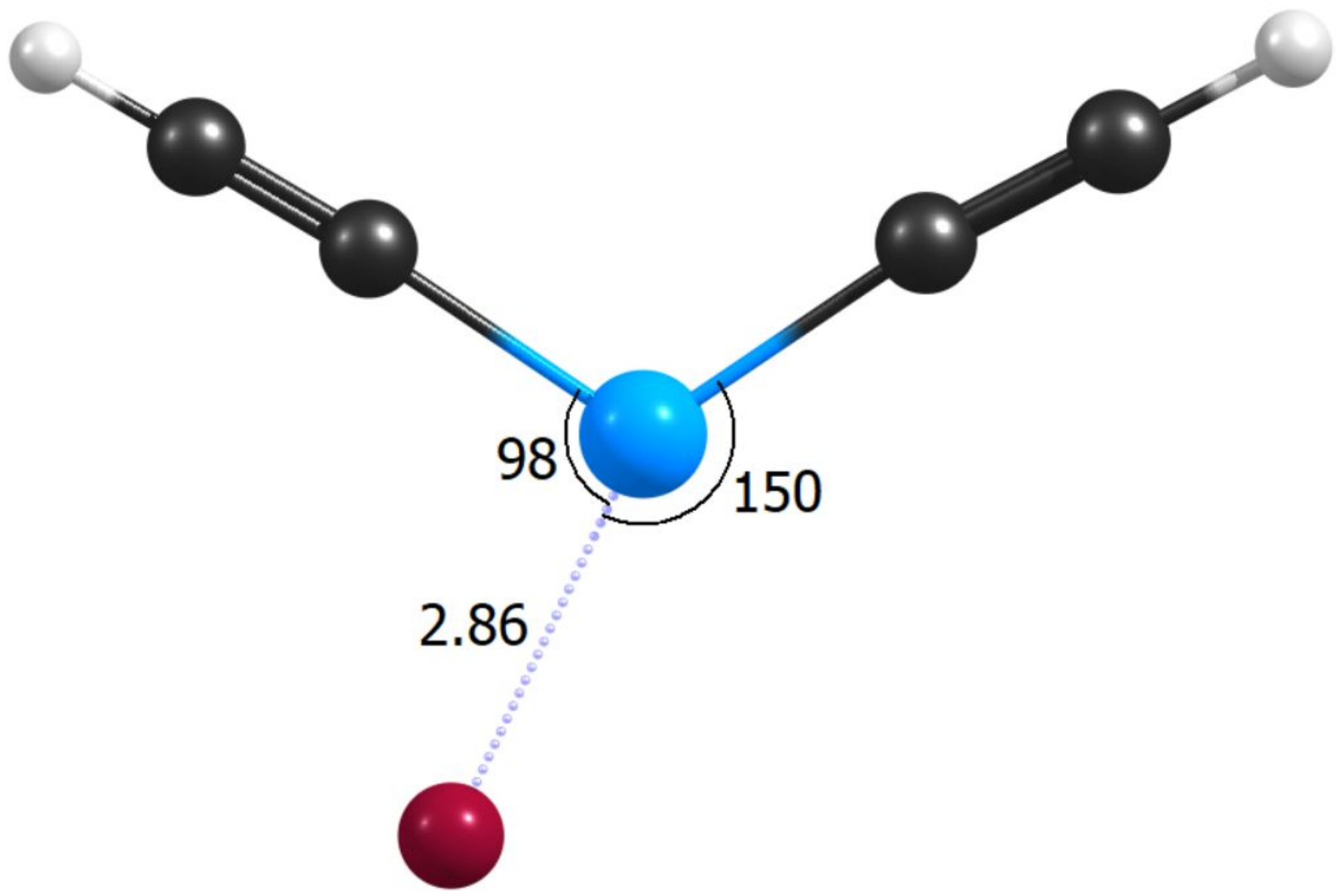

Figure S46. The optimized geometry of isomer 2bI-singlet $Ta^+C_4H_2Ar$ followed by its predicted frequencies (cm^{-1}) and IR intensities (km/mol).

Frequency (cm^{-1})	Intensity (km/mol)	Frequency (cm^{-1})	Intensity (km/mol)
24.04	0.5417	515.75	37.6987
41.85	1.4794	771.60	29.1605
65.47	6.5618	778.94	29.9982
99.46	7.7565	782.58	28.3884
169.02	11.1422	782.91	28.5868
170.87	15.1731	2028.47	511.9293
186.23	35.5838	2042.07	166.2649
254.04	27.2083	3390.11	124.2249
508.92	82.8481	3390.87	61.8543

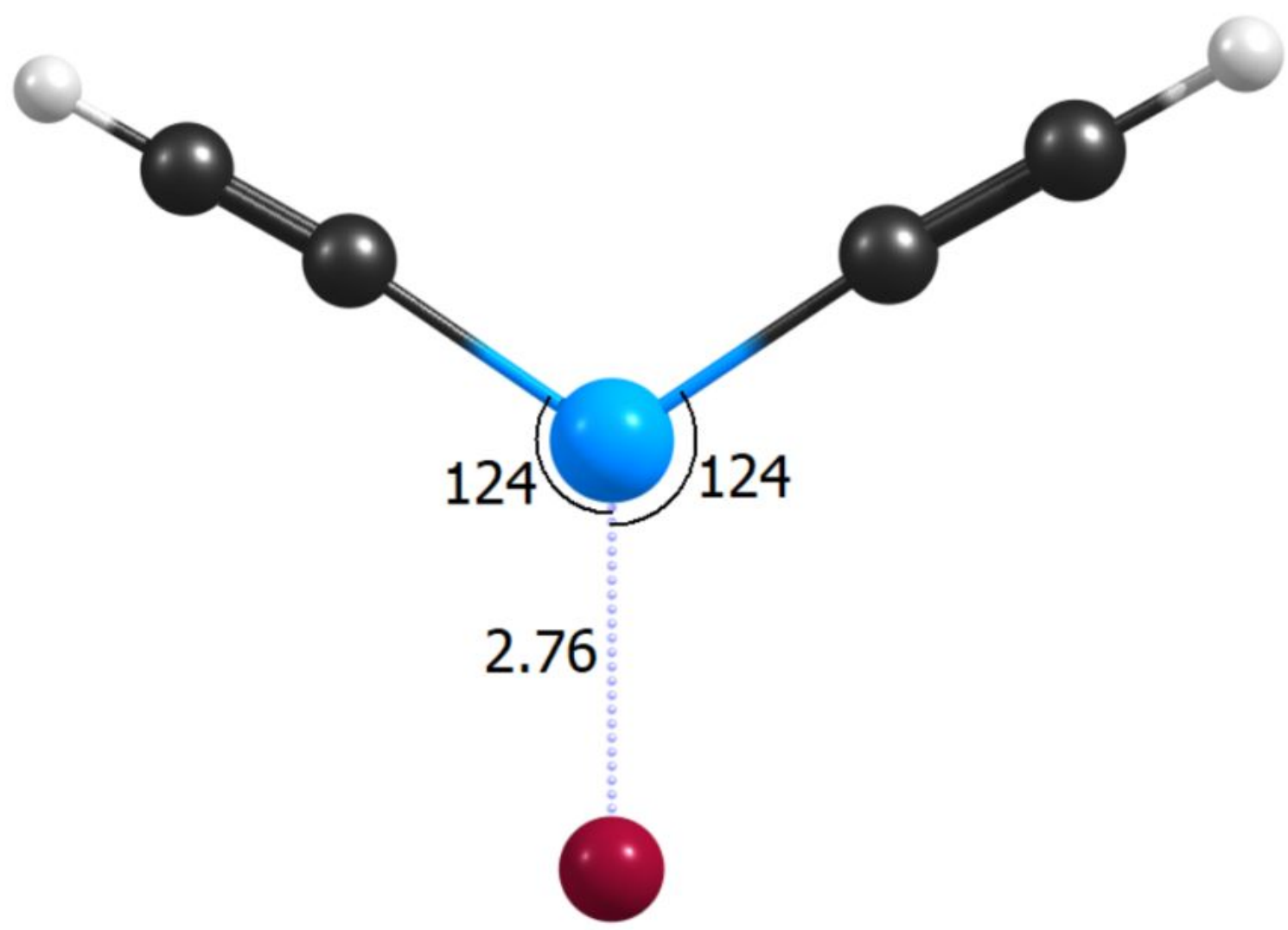

Figure S47. The optimized geometry of isomer 2bII-singlet $Ta^+C_4H_2Ar$ followed by its predicted frequencies (cm^{-1}) and IR intensities (km/mol).

Frequency (cm^{-1})	Intensity (km/mol)	Frequency (cm^{-1})	Intensity (km/mol)
22.61	0.0273	513.73	34.3402
49.19	2.5241	767.21	0.0000
63.13	6.5188	767.55	62.0006
124.00	3.7196	791.61	34.1859
165.79	11.0128	792.42	21.4152
176.09	0.0000	2032.44	456.1462
191.28	48.0837	2046.33	134.7877
246.01	25.1447	3391.33	163.8269
504.99	76.0470	3392.38	37.8472

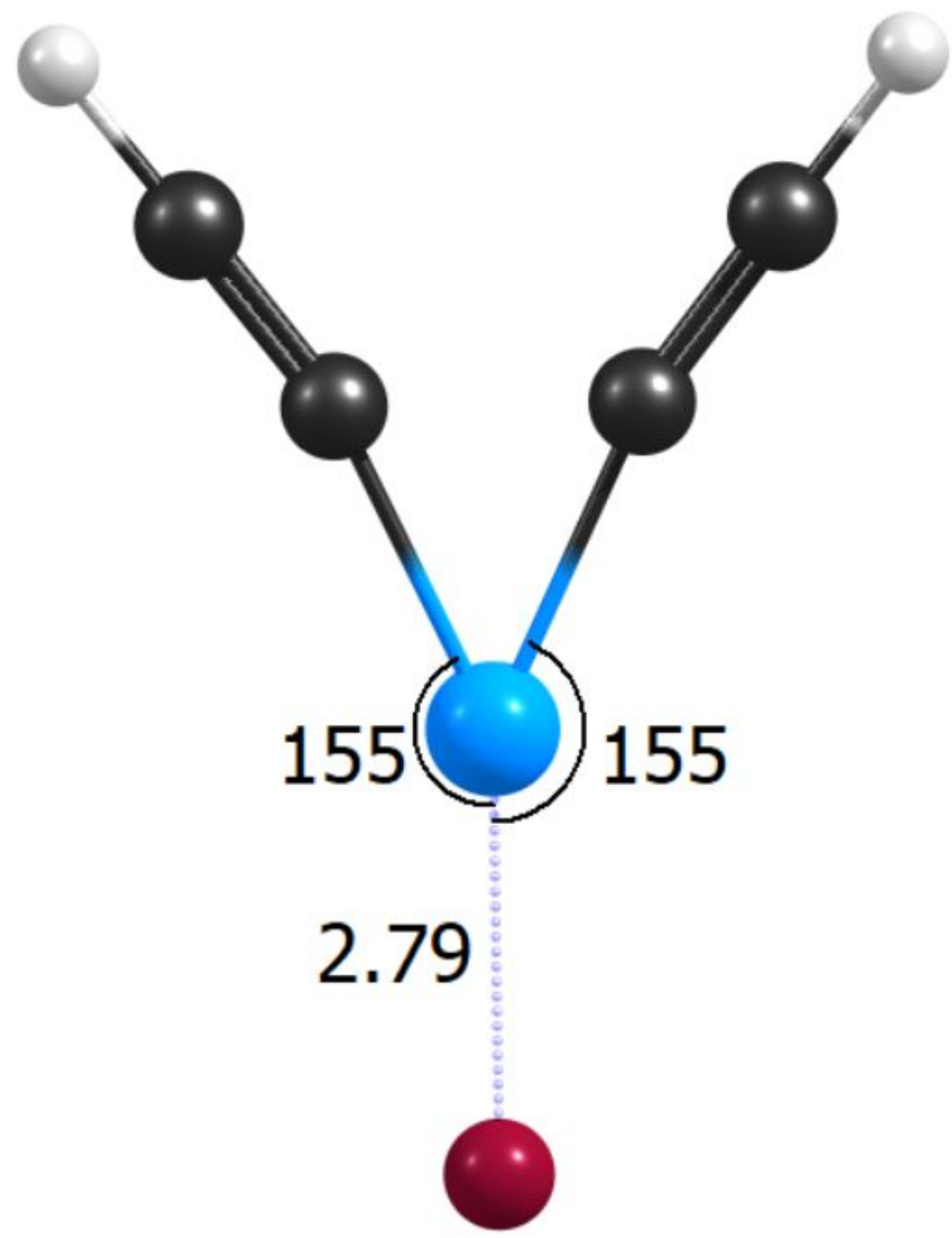

Figure S48. The optimized geometry of isomer 2bI-quintet $Ta^+C_4H_2Ar$ followed by its predicted frequencies (cm^{-1}) and IR intensities (km/mol).

Frequency (cm^{-1})	Intensity (km/mol)	Frequency (cm^{-1})	Intensity (km/mol)
35.03	0.4799	476.90	1.2963
53.17	2.3660	576.70	21.2284
122.65	7.6942	587.70	29.0547
171.93	0.1036	643.62	0.0042
246.01	0.7048	643.97	87.3773
278.52	25.1180	1714.93	0.1043
279.95	30.2267	1796.26	2.4915
281.04	0.0005	3378.85	164.9913
433.80	7.0264	3383.17	229.1647

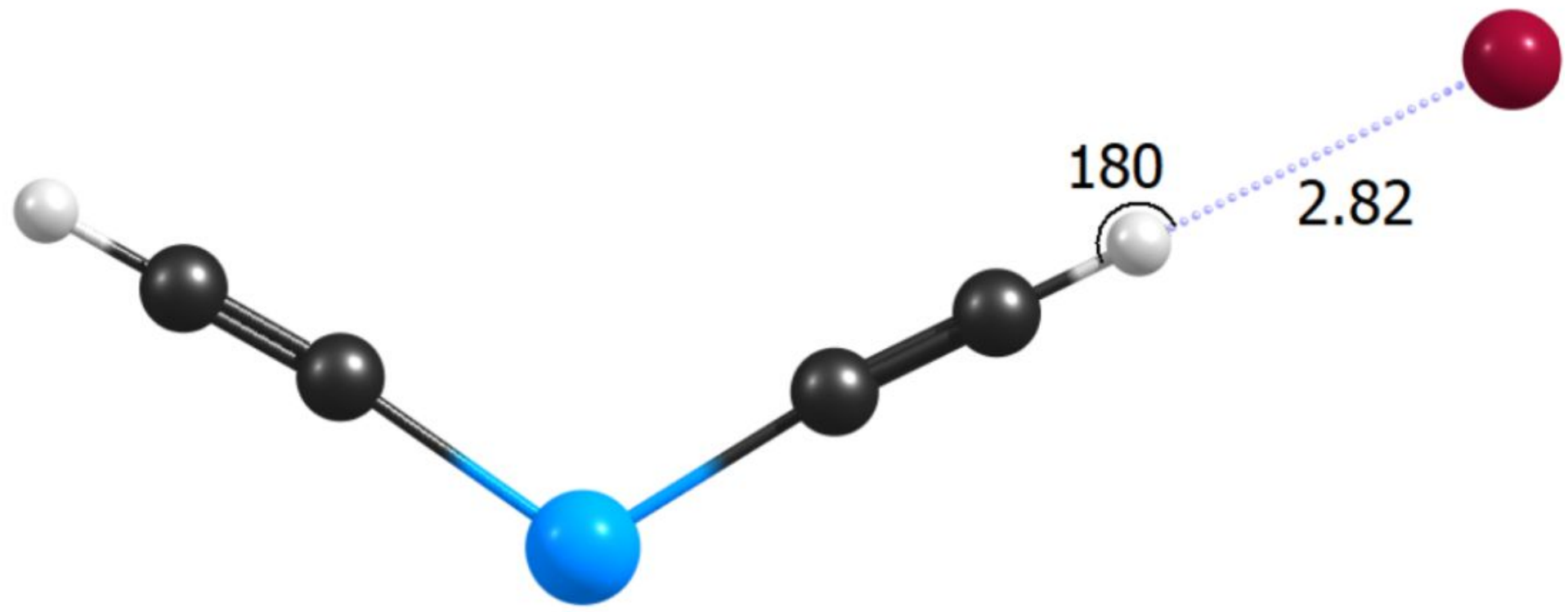

Figure S49. The optimized geometry of isomer 2bII-singlet $Ta^+C_4H_2Ar$ followed by its predicted frequencies (cm^{-1}) and IR intensities (km/mol).

Frequency (cm^{-1})	Intensity (km/mol)	Frequency (cm^{-1})	Intensity (km/mol)
11.85	0.0557	525.56	25.4416
15.92	0.0299	776.55	31.1560
29.98	1.6747	787.59	27.9866
67.34	4.8792	789.58	23.5992
168.07	55.5563	801.64	20.0143
171.93	8.7554	2024.19	677.0069
177.20	0.4619	2038.73	130.7661
260.39	28.9275	3368.66	190.2157
517.41	78.8636	3385.85	94.5170

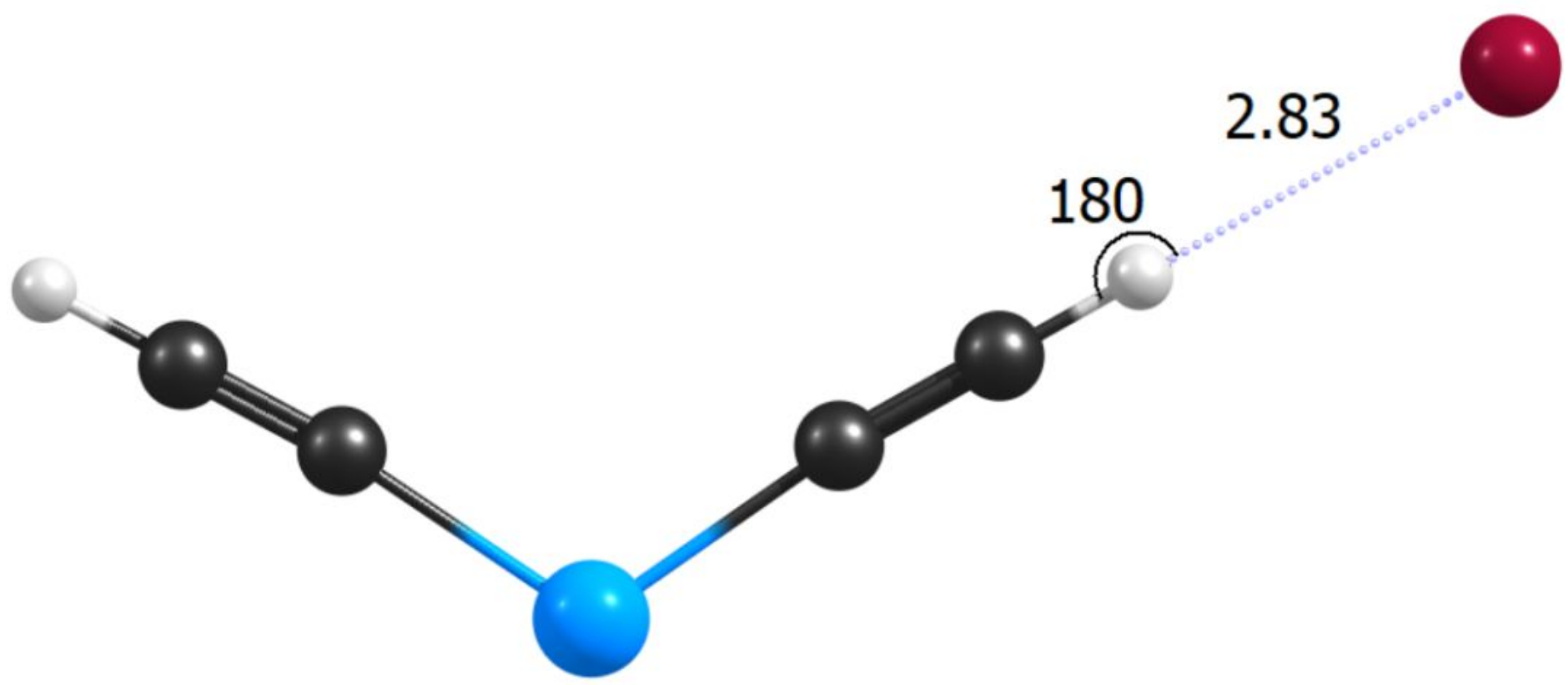

Figure S50. The optimized geometry of isomer 2bII-triplet $Ta^+C_4H_2Ar$ followed by its predicted frequencies (cm^{-1}) and IR intensities (km/mol).

Frequency (cm^{-1})	Intensity (km/mol)	Frequency (cm^{-1})	Intensity (km/mol)
11.61	0.0205	524.20	24.1404
15.81	0.0010	774.39	30.6841
29.50	1.6527	788.66	29.5866
67.02	4.2798	788.68	23.2016
168.90	10.5455	801.19	22.4861
173.87	60.8254	2026.61	604.3934
181.71	0.5492	2041.16	130.8244
249.01	29.4972	3370.59	196.8023
513.43	73.8658	3387.21	97.1661

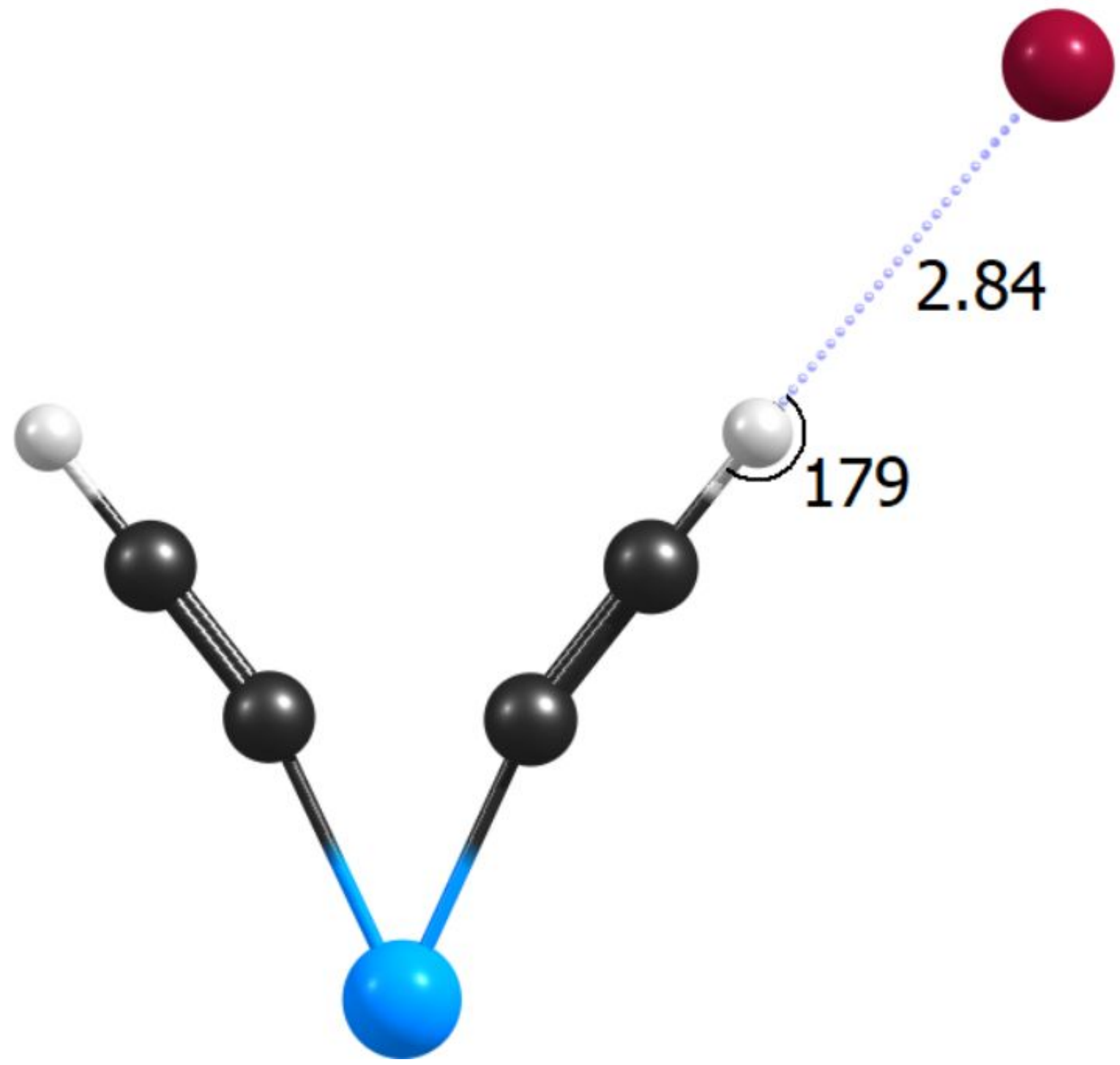

Figure S51. The optimized geometry of isomer 2bII-quintet $Ta^+C_4H_2Ar$ followed by its predicted frequencies (cm^{-1}) and IR intensities (km/mol).

Frequency (cm^{-1})	Intensity (km/mol)	Frequency (cm^{-1})	Intensity (km/mol)
11.85	0.5079	484.84	0.0395
17.28	0.2134	581.61	22.3405
29.07	1.5360	602.80	23.1046
162.14	0.4639	652.46	45.0097
244.99	2.2179	670.23	33.9830
261.50	40.1499	1715.31	1.1144
274.64	23.3197	1796.16	0.2673
285.03	0.0013	3359.96	307.9384
435.06	8.6595	3377.22	203.7140

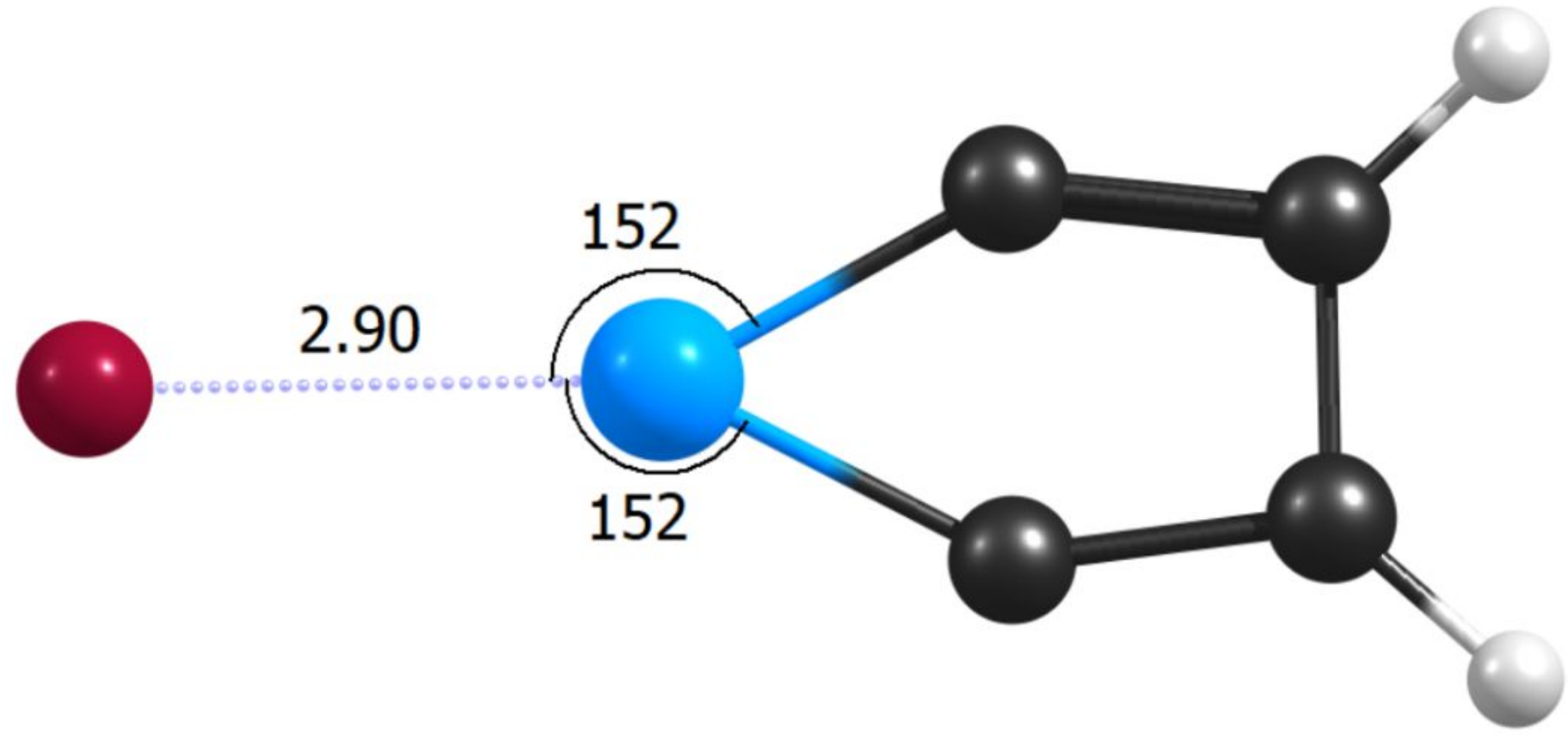

Figure S52. The optimized geometry of isomer 2cI-singlet $Ta^+C_4H_2Ar$ followed by its predicted frequencies (cm^{-1}) and IR intensities (km/mol).

Frequency (cm^{-1})	Intensity (km/mol)	Frequency (cm^{-1})	Intensity (km/mol)
18.88	0.1868	805.00	0.0005
23.19	0.0016	926.32	0.0000
111.93	5.5412	1087.87	5.3982
318.44	51.0583	1165.77	4.7157
473.84	0.1509	1165.80	4.6698
540.34	0.0000	1310.94	13.9912
587.77	35.1395	1475.73	13.7420
729.69	20.7726	3217.87	6.0418
737.71	34.5443	3238.72	10.9650

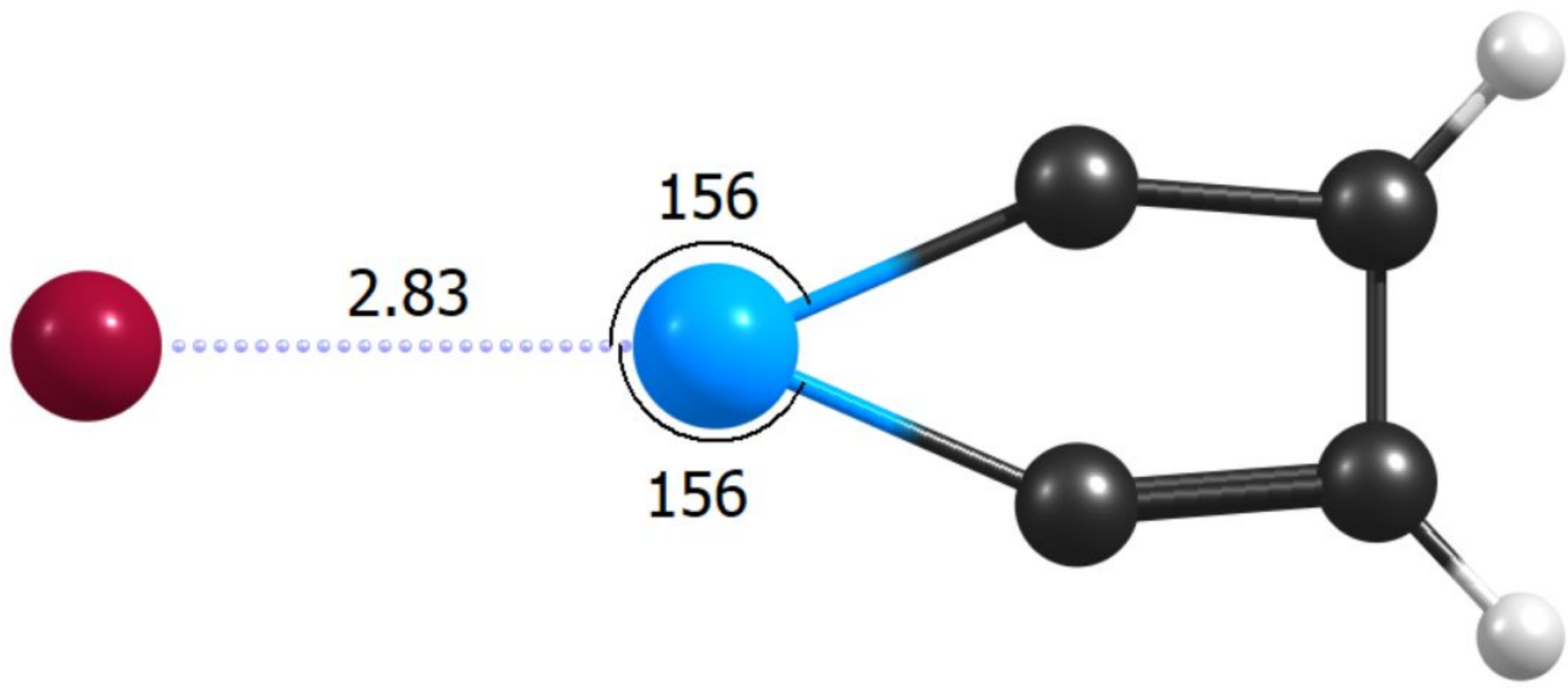

Figure S53. The optimized geometry of isomer 2cI-triplet $Ta^+C_4H_2Ar$ followed by its predicted frequencies (cm^{-1}) and IR intensities (km/mol).

Frequency (cm^{-1})	Intensity (km/mol)	Frequency (cm^{-1})	Intensity (km/mol)
27.79	0.0390	889.58	0.3939
38.69	0.4210	944.46	0.0000
118.58	7.5579	1022.29	3.9830
316.02	22.7094	1109.38	3.4631
380.65	0.5867	1206.08	0.8976
467.79	0.0000	1252.16	29.7308
470.03	22.1112	1567.71	7.7854
707.98	14.4661	3216.67	9.0158
733.51	39.7186	3244.08	9.5824

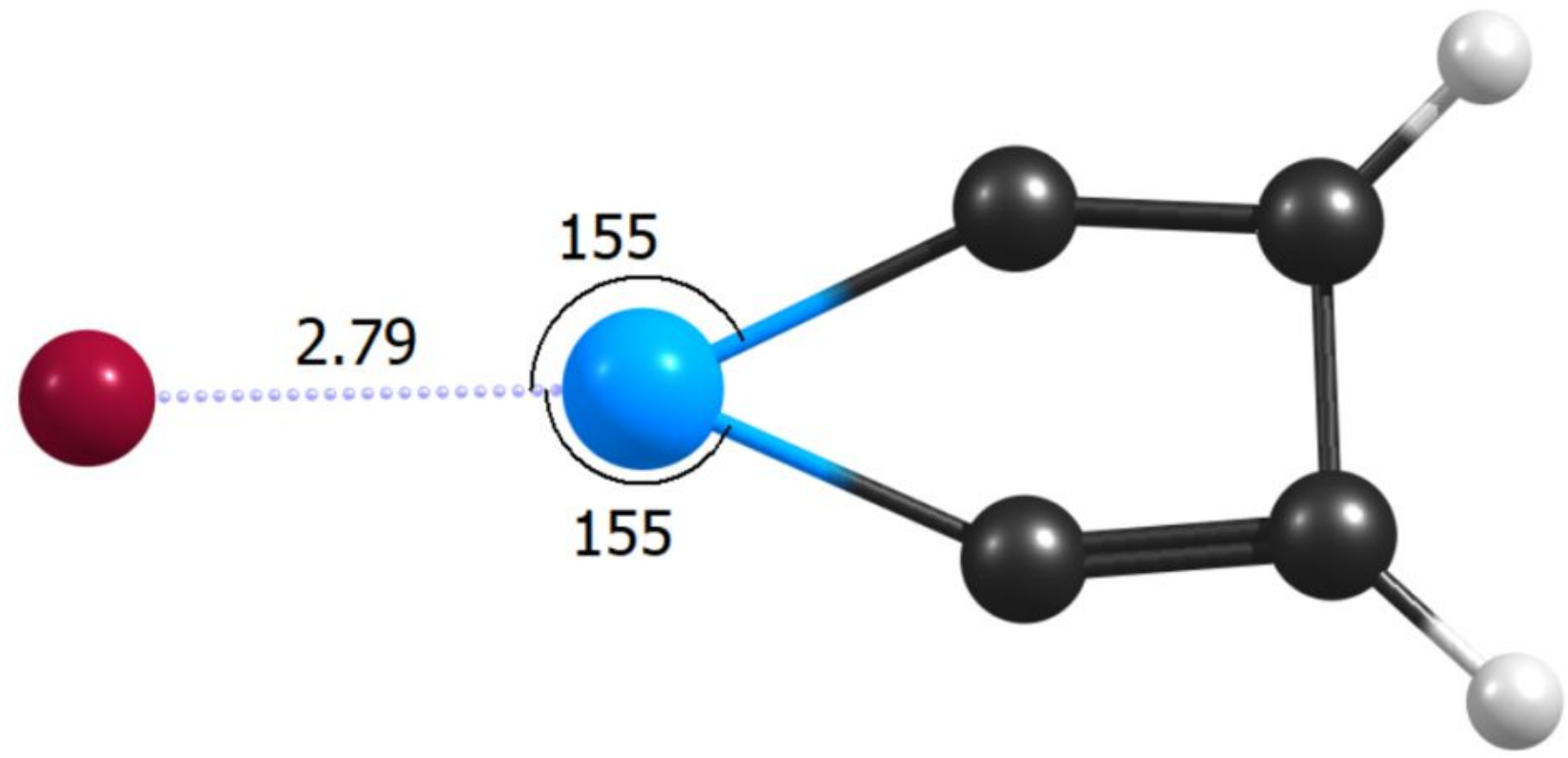

Figure S54. The optimized geometry of isomer 2cI-quintet $Ta^{+}C_4H_2Ar$ followed by its predicted frequencies (cm^{-1}) and IR intensities (km/mol).

Frequency (cm^{-1})	Intensity (km/mol)	Frequency (cm^{-1})	Intensity (km/mol)
26.22	0.1310	764.47	0.0000
38.37	0.7021	765.73	30.1991
124.60	8.3074	1046.66	0.3051
225.33	0.0000	1151.69	14.6529
275.51	16.6785	1256.02	40.3304
444.97	11.4803	1334.69	2.1409
457.59	11.1812	1400.38	37.2831
613.26	34.5808	3203.96	8.7402
680.30	4.8125	3218.24	13.4941

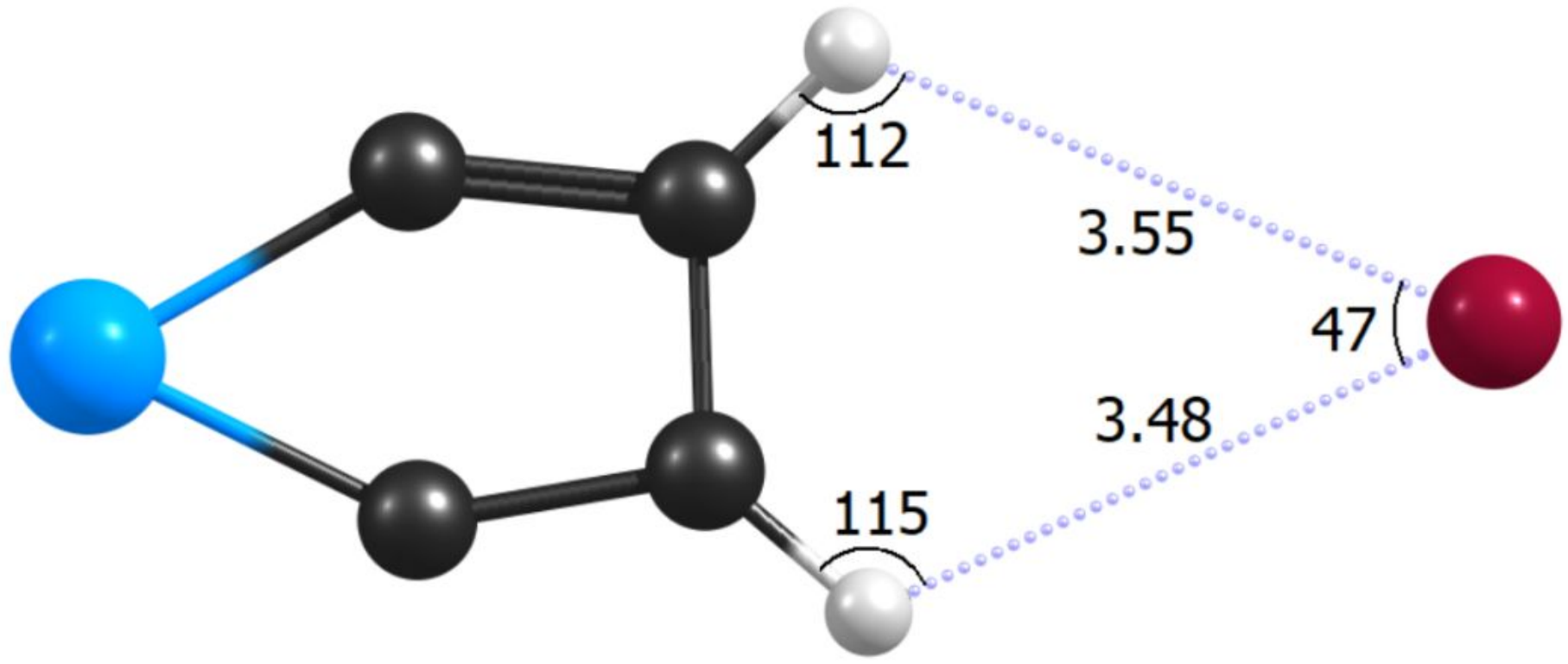

Figure S55. The optimized geometry of isomer 2cII-singlet $Ta^+C_4H_2Ar$ followed by its predicted frequencies (cm^{-1}) and IR intensities (km/mol).

Frequency (cm^{-1})	Intensity (km/mol)	Frequency (cm^{-1})	Intensity (km/mol)
1.41	0.0342	797.04	0.2099
8.66	0.1260	928.89	0.0001
16.88	0.9331	1087.52	9.2259
294.17	62.5725	1164.68	6.3268
471.11	2.4394	1167.09	5.4415
538.11	0.0000	1308.22	16.8927
589.01	37.9354	1475.26	17.4089
733.09	33.3703	3219.83	6.4789
733.31	18.1614	3240.39	13.3587

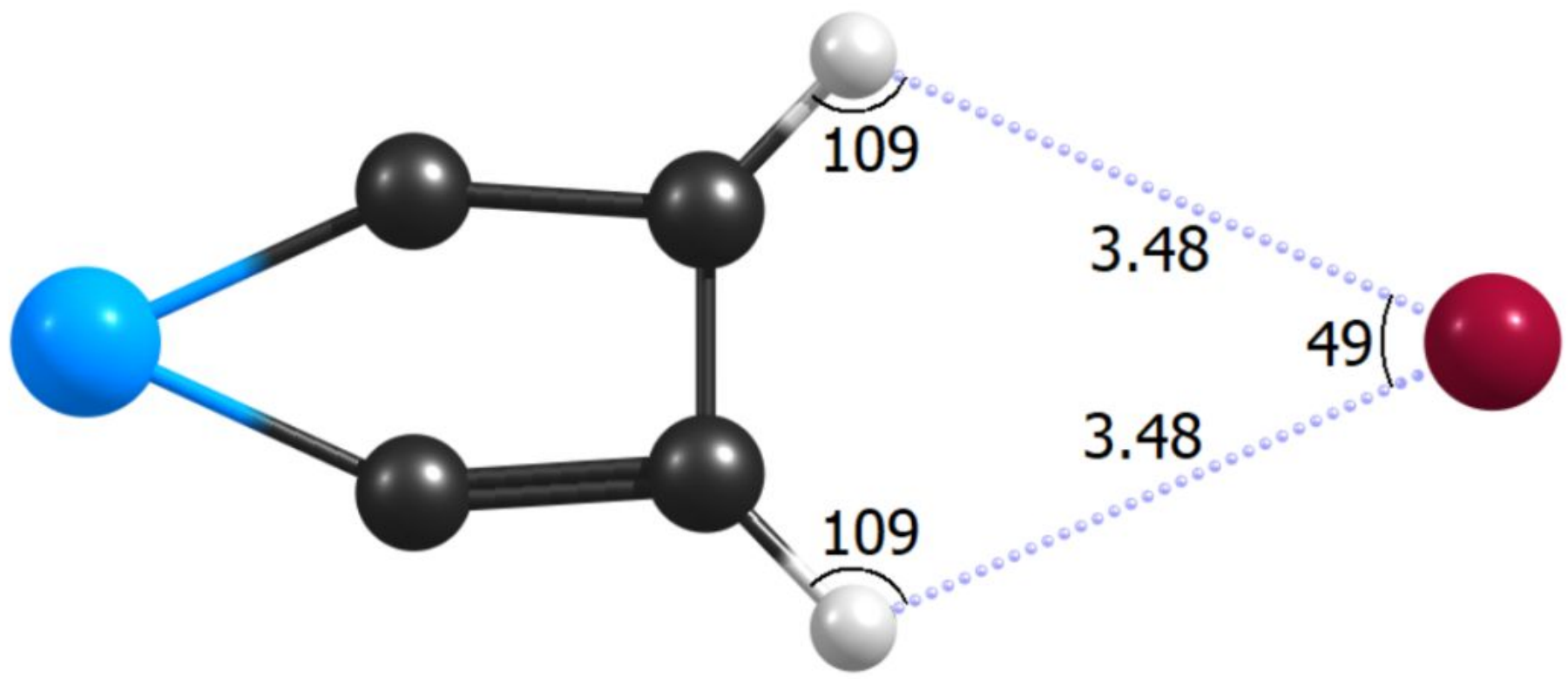

Figure S56. The optimized geometry of isomer 2cII-triplet $Ta^+C_4H_2Ar$ followed by its predicted frequencies (cm^{-1}) and IR intensities (km/mol).

Frequency (cm^{-1})	Intensity (km/mol)	Frequency (cm^{-1})	Intensity (km/mol)
5.94	0.1722	870.52	0.5210
9.81	0.0802	945.93	0.0000
19.26	0.9806	1017.63	5.4468
288.27	27.6433	1105.88	4.9377
385.33	4.1694	1206.00	0.0133
465.79	0.0000	1249.07	34.3202
469.47	24.1495	1563.55	8.7892
693.66	12.3895	3219.63	10.7541
735.26	36.3879	3246.57	13.8055

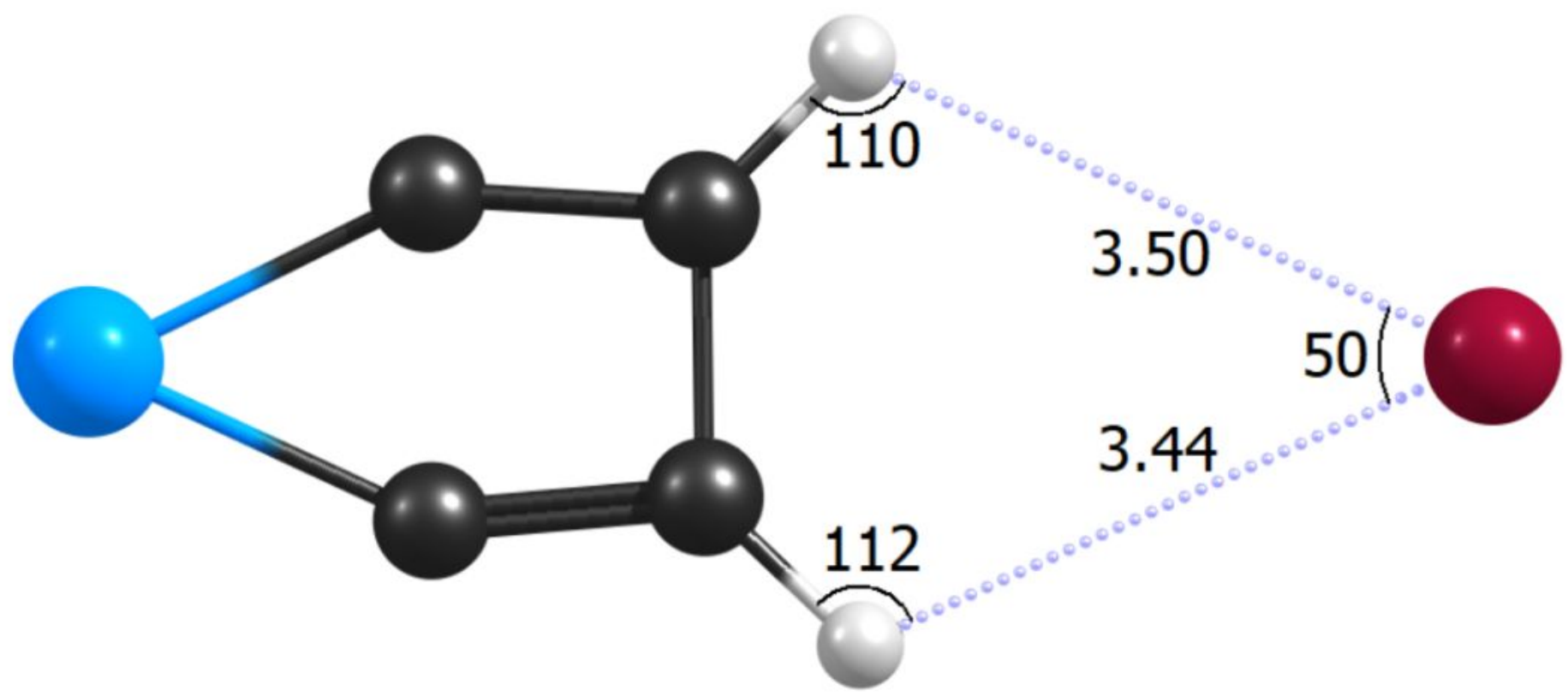

Figure S57. The optimized geometry of isomer 2cII-quintet $Ta^{+}C_4H_2Ar$ followed by its predicted frequencies (cm^{-1}) and IR intensities (km/mol).

Frequency (cm^{-1})	Intensity (km/mol)	Frequency (cm^{-1})	Intensity (km/mol)
6.85	0.3865	759.32	29.2392
6.95	0.2578	772.72	0.0000
20.37	1.0200	1044.38	0.0529
225.86	0.0001	1151.69	19.8635
253.86	21.2622	1255.67	44.9114
442.78	4.4929	1333.21	2.3060
454.52	10.4000	1399.60	45.8401
620.50	32.4887	3206.41	11.6012
660.30	12.3921	3220.61	19.3485

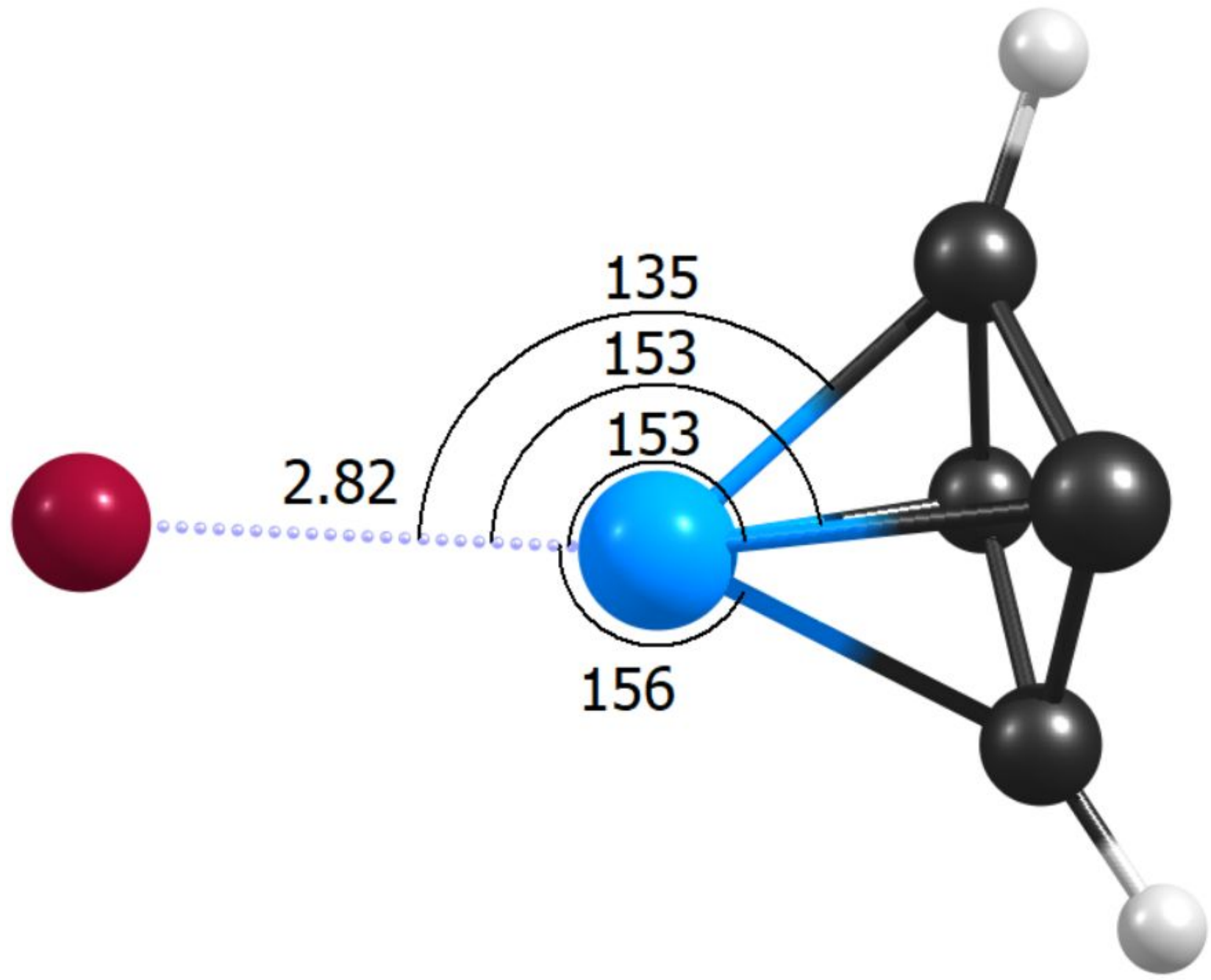

Figure S58. The optimized geometry of isomer 2dI-singlet $Ta^{+}C_4H_2Ar$ followed by its predicted frequencies (cm^{-1}) and IR intensities (km/mol).

Frequency (cm^{-1})	Intensity (km/mol)	Frequency (cm^{-1})	Intensity (km/mol)
11.63	1.3958	815.54	0.1031
44.14	2.4920	851.31	117.4953
123.55	4.8665	918.38	4.3645
274.84	38.4768	981.42	6.4794
372.76	18.0643	1040.88	0.0150
514.79	1.4434	1152.54	11.6123
594.12	2.5050	1301.93	2.8874
731.10	24.0214	3226.31	33.4838
734.36	12.5586	3230.06	9.6656

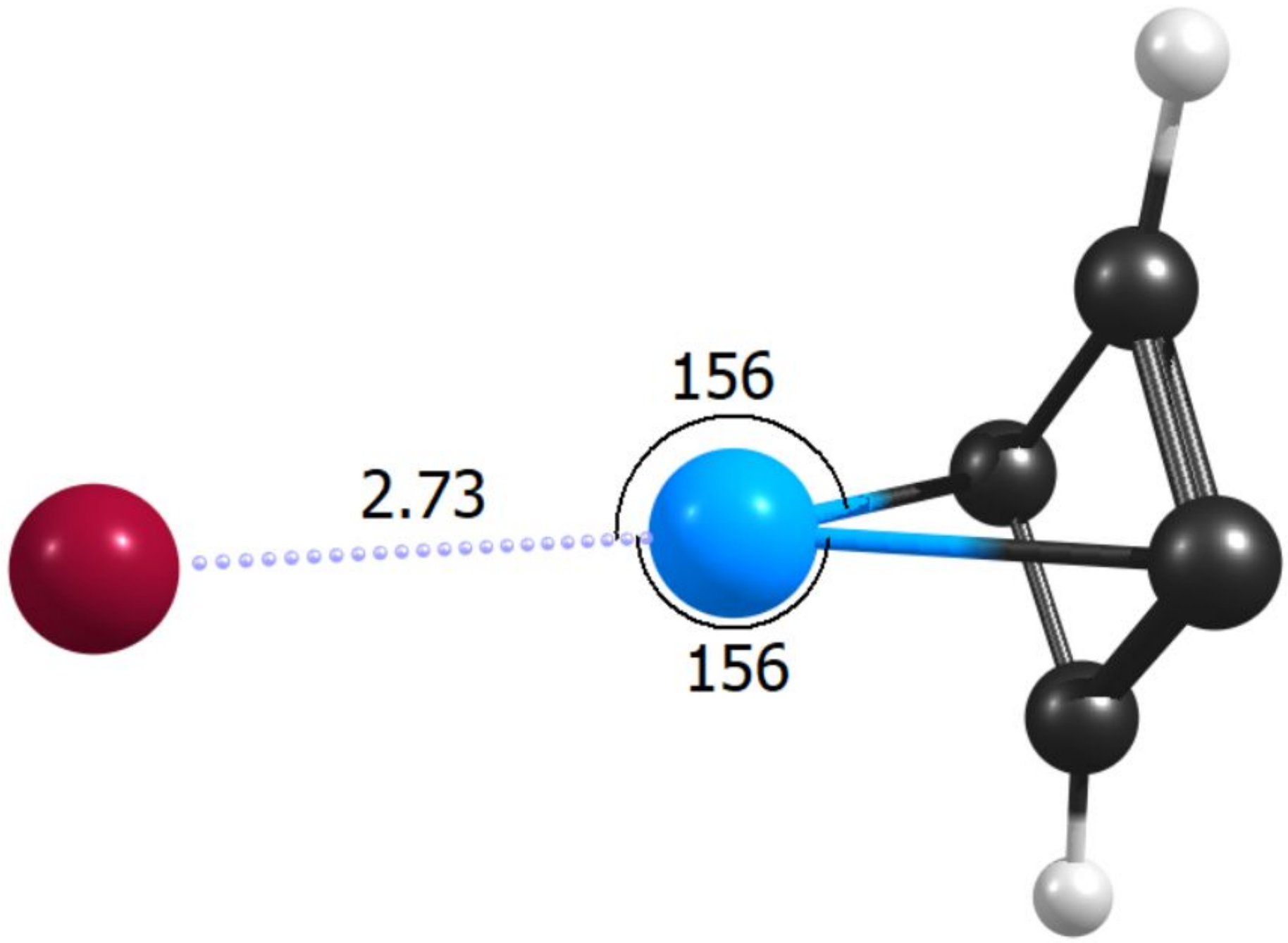

Figure S59. The optimized geometry of isomer 2dI-triplet $Ta^+C_4H_2Ar$ followed by its predicted frequencies (cm^{-1}) and IR intensities (km/mol).

Frequency (cm^{-1})	Intensity (km/mol)	Frequency (cm^{-1})	Intensity (km/mol)
40.35	0.8039	804.00	67.0666
48.50	0.4690	878.82	0.3276
139.66	4.9981	900.19	0.0000
161.83	17.0093	1069.63	0.0000
343.34	0.6968	1096.83	0.2161
475.54	5.4185	1288.39	10.8633
479.35	13.0037	1364.21	0.7960
566.64	8.9900	3314.69	153.0486
711.31	24.9171	3320.51	21.6101

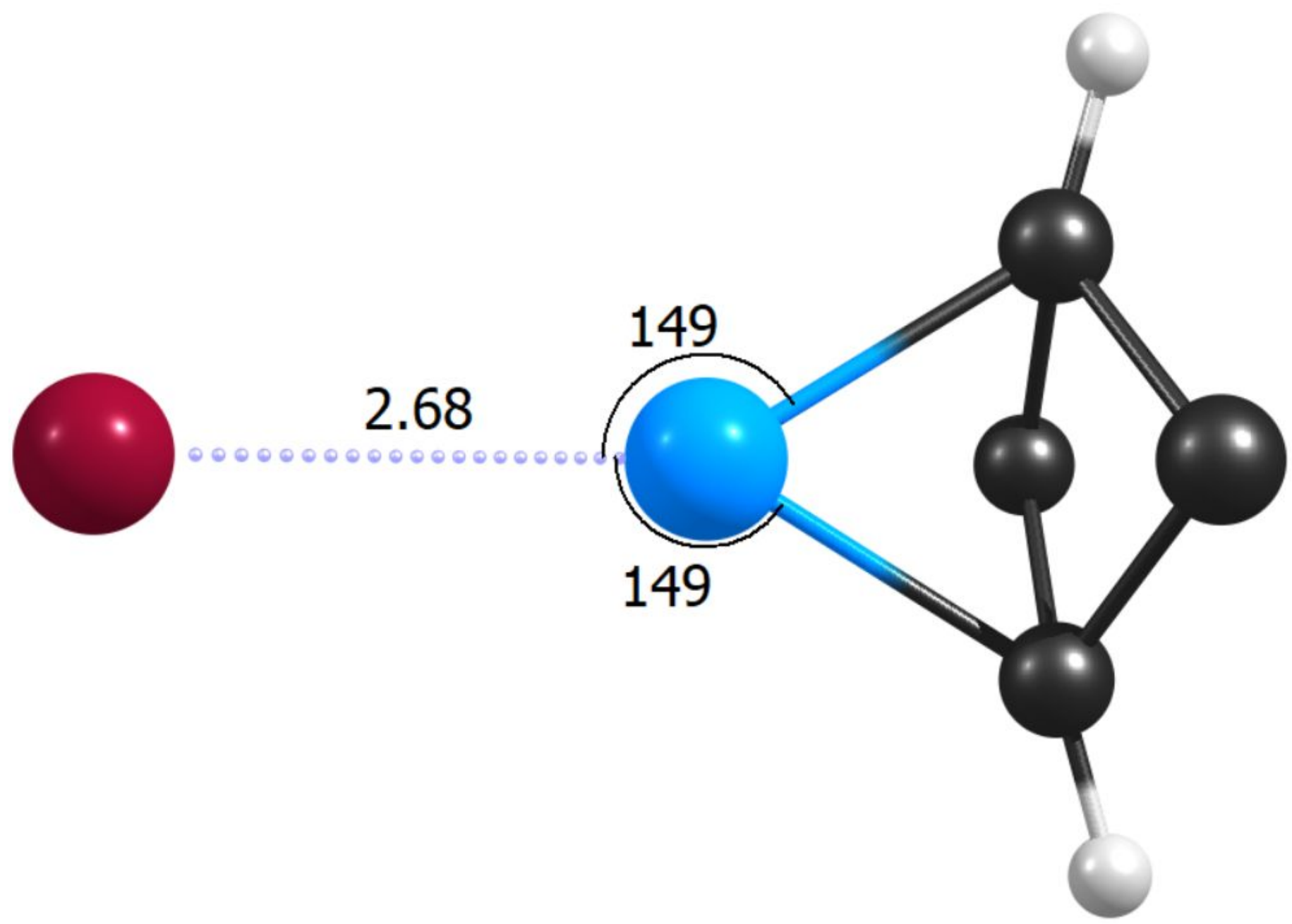

Figure S60. The optimized geometry of isomer 2dI-quintet $Ta^{+}C_4H_2Ar$ followed by its predicted frequencies (cm^{-1}) and IR intensities (km/mol).

Frequency (cm^{-1})	Intensity (km/mol)	Frequency (cm^{-1})	Intensity (km/mol)
39.79	0.0000	771.88	1.0794
46.38	0.0137	772.16	72.9532
150.43	5.3036	946.85	0.6475
266.47	0.4211	1038.83	0.0000
303.50	14.3838	1084.32	4.7755
384.92	0.0019	1246.84	8.0177
567.43	48.5676	1325.92	1.1348
729.44	64.5126	3252.08	87.7013
741.23	0.0000	3255.68	14.3921

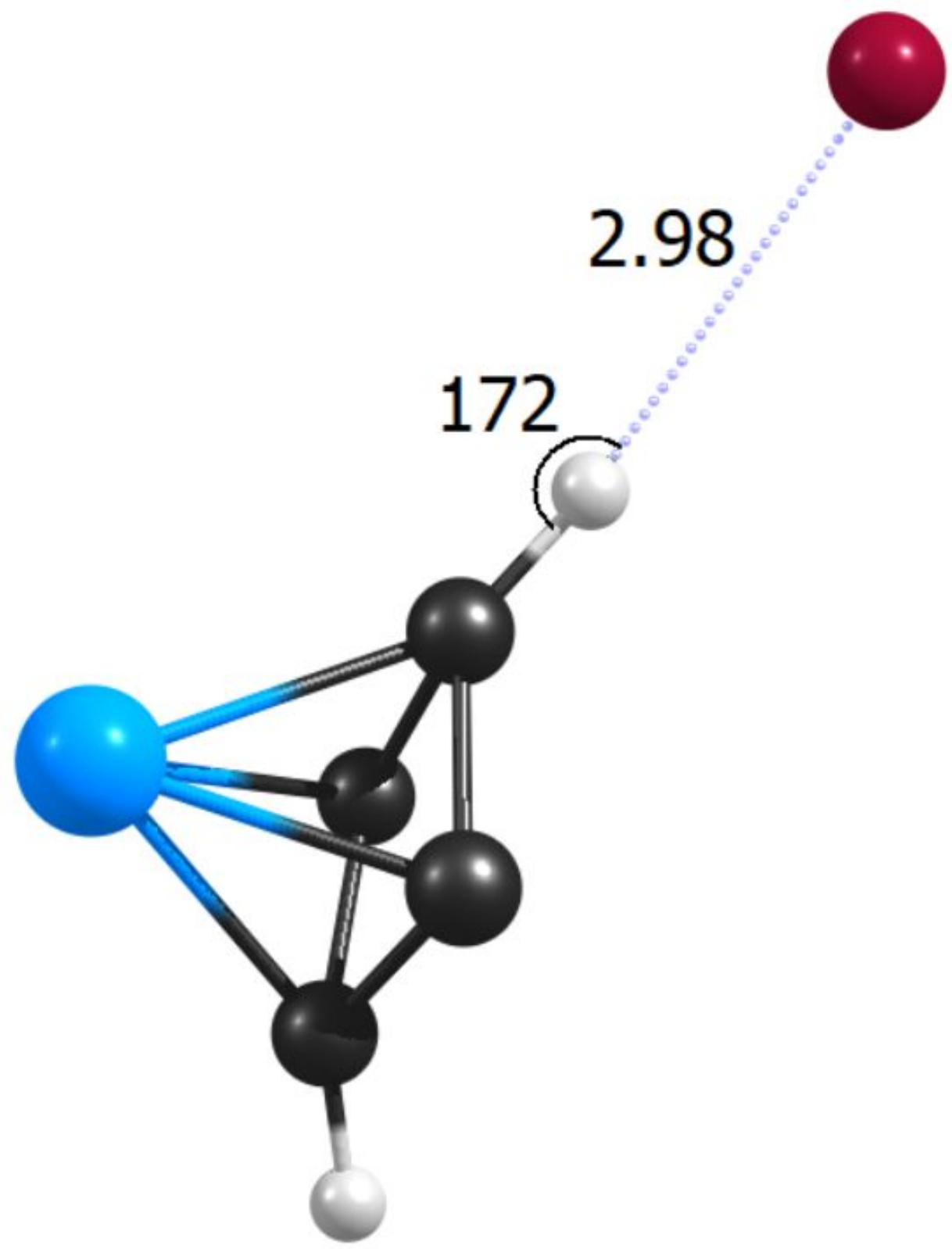

Figure S61. The optimized geometry of isomer 2dII-singlet $Ta^+C_4H_2Ar$ followed by its predicted frequencies (cm^{-1}) and IR intensities (km/mol).

Frequency (cm^{-1})	Intensity (km/mol)	Frequency (cm^{-1})	Intensity (km/mol)
8.23	2.3153	806.31	0.0016
13.25	0.2163	852.83	127.9737
26.55	0.2694	916.15	0.6928
275.82	34.4231	980.75	8.3725
384.95	13.0554	1041.16	0.0272
528.66	1.8455	1141.16	2.7625
589.22	3.1057	1287.51	5.5969
731.75	20.0120	3222.42	66.3077
736.08	13.5299	3226.25	8.4387

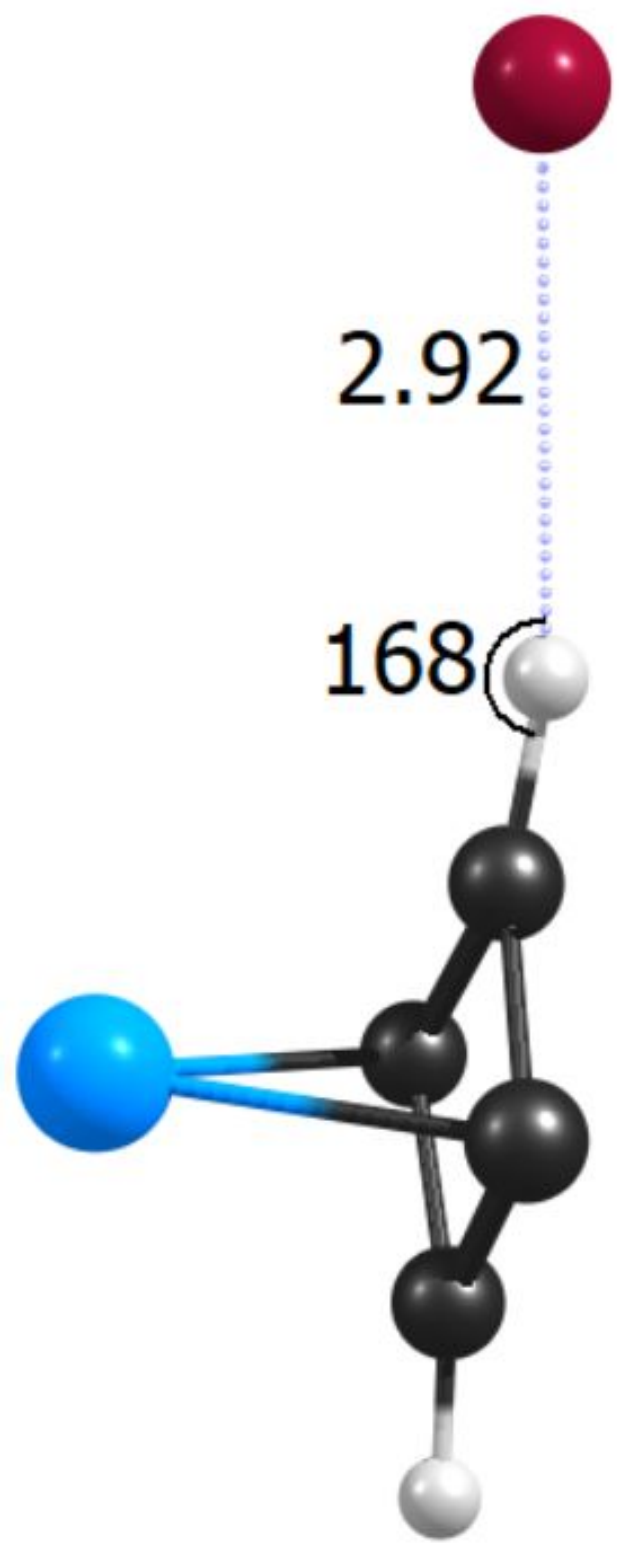

Figure S62. The optimized geometry of isomer 2dII-triplet $Ta^{+}C_4H_2Ar$ followed by its predicted frequencies (cm^{-1}) and IR intensities (km/mol).

Frequency (cm^{-1})	Intensity (km/mol)	Frequency (cm^{-1})	Intensity (km/mol)
5.27	1.3580	804.57	59.6226
14.06	0.0034	877.37	2.4467
32.88	0.2613	884.05	0.0015
169.09	18.1908	1070.05	0.0739
346.98	0.5491	1097.35	0.3577
463.50	9.4937	1283.37	15.2316
480.94	9.2458	1361.40	0.0430
577.11	15.5038	3303.86	199.6692
712.26	7.6397	3313.91	49.4145

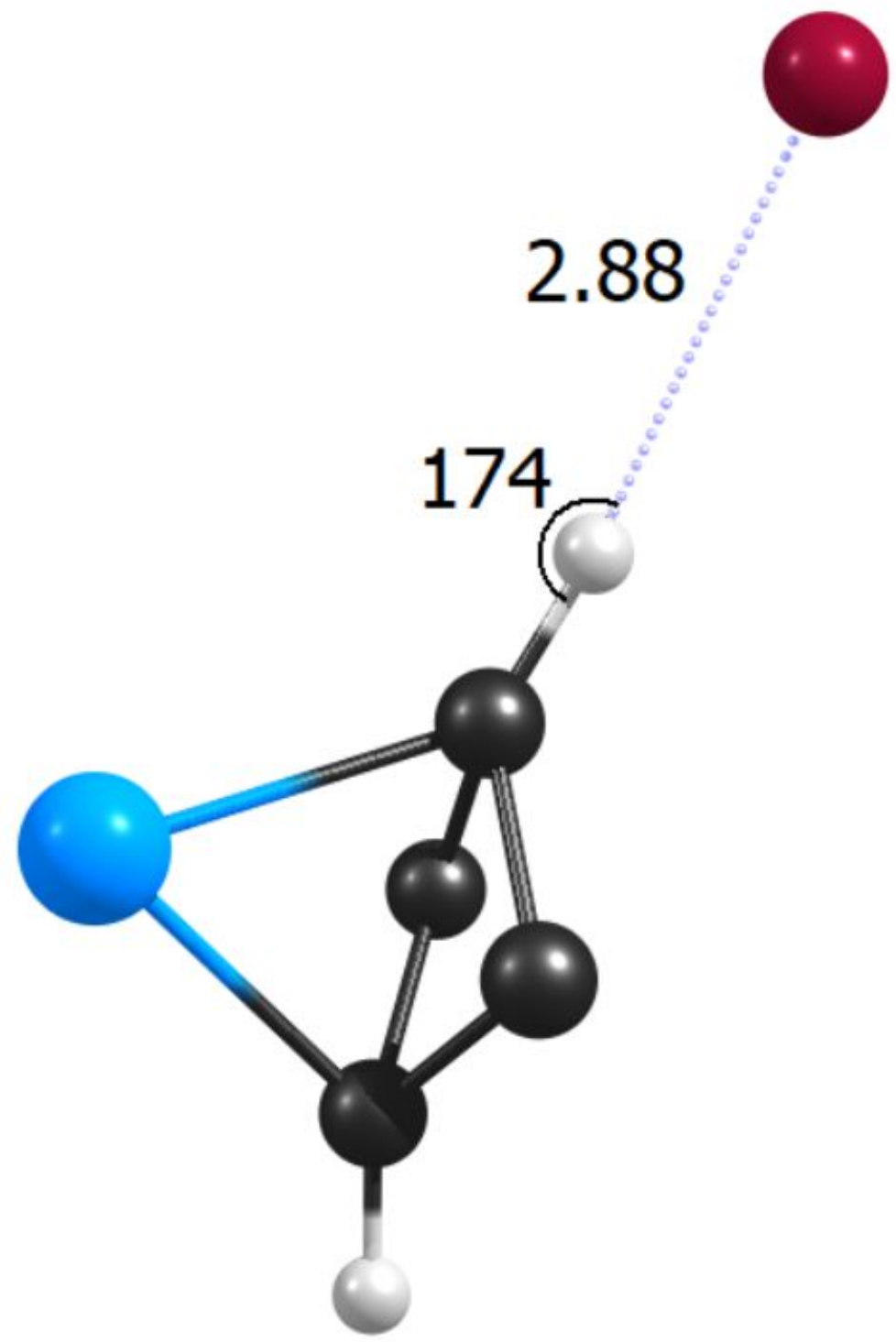

Figure S63. The optimized geometry of isomer 2dII-quintet $Ta^+C_4H_2Ar$ followed by its predicted frequencies (cm^{-1}) and IR intensities (km/mol).

Frequency (cm^{-1})	Intensity (km/mol)	Frequency (cm^{-1})	Intensity (km/mol)
8.57	0.4841	763.82	3.7758
16.62	0.0068	777.55	79.0769
32.23	0.8252	945.46	3.1018
254.21	0.1444	1040.95	0.1747
289.83	15.6760	1084.26	3.9195
390.17	1.5592	1245.29	5.8867
550.38	51.5113	1325.49	2.5016
727.51	64.9211	3243.29	142.5734
735.46	0.0437	3251.95	32.1482

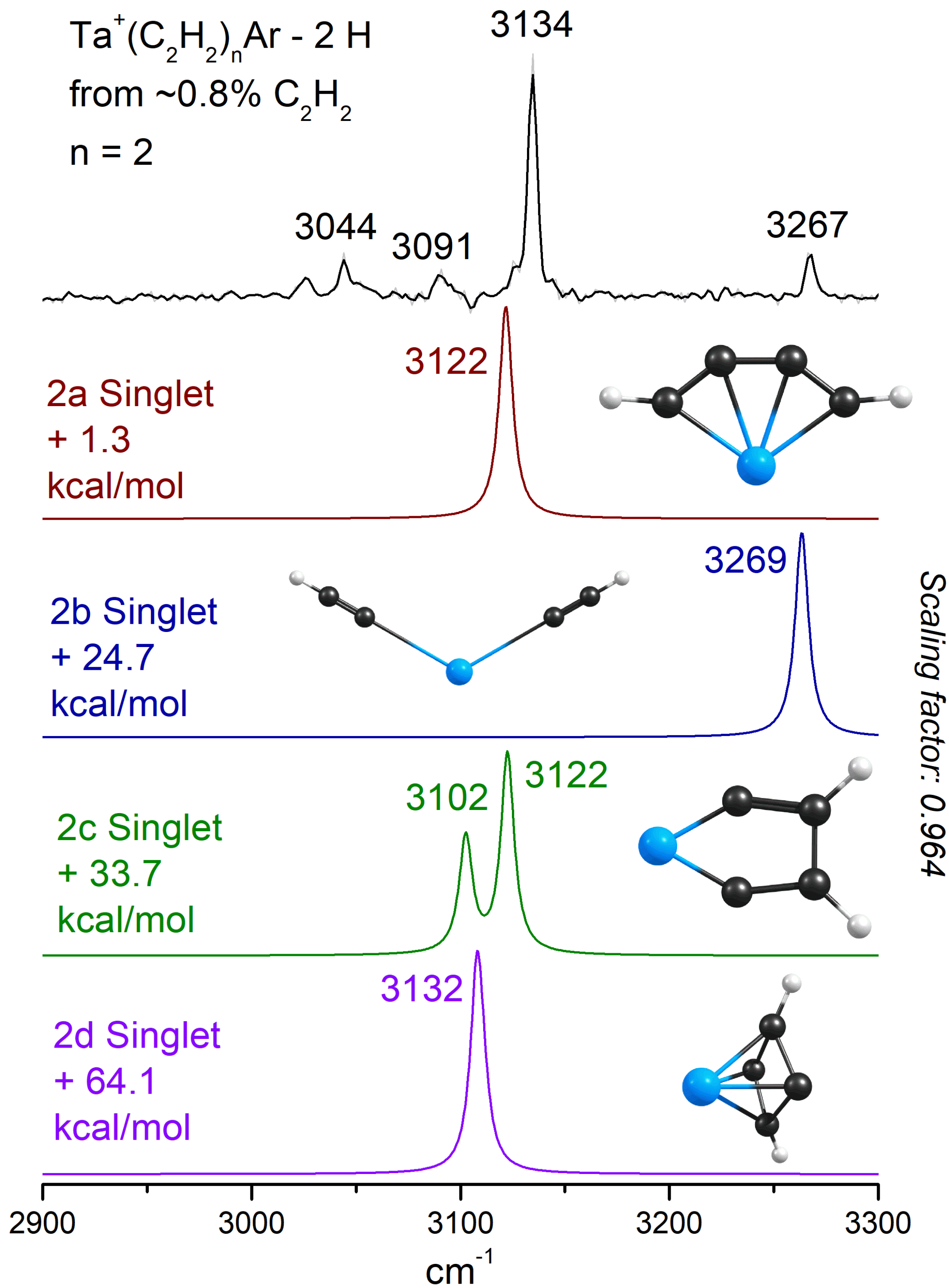

Figure S64. The experimental IR photodissociation spectrum of $Ta^+C_4H_2Ar$ compared with simulated spectra for singlet isomers 2a-2d of $Ta^+C_4H_2$.

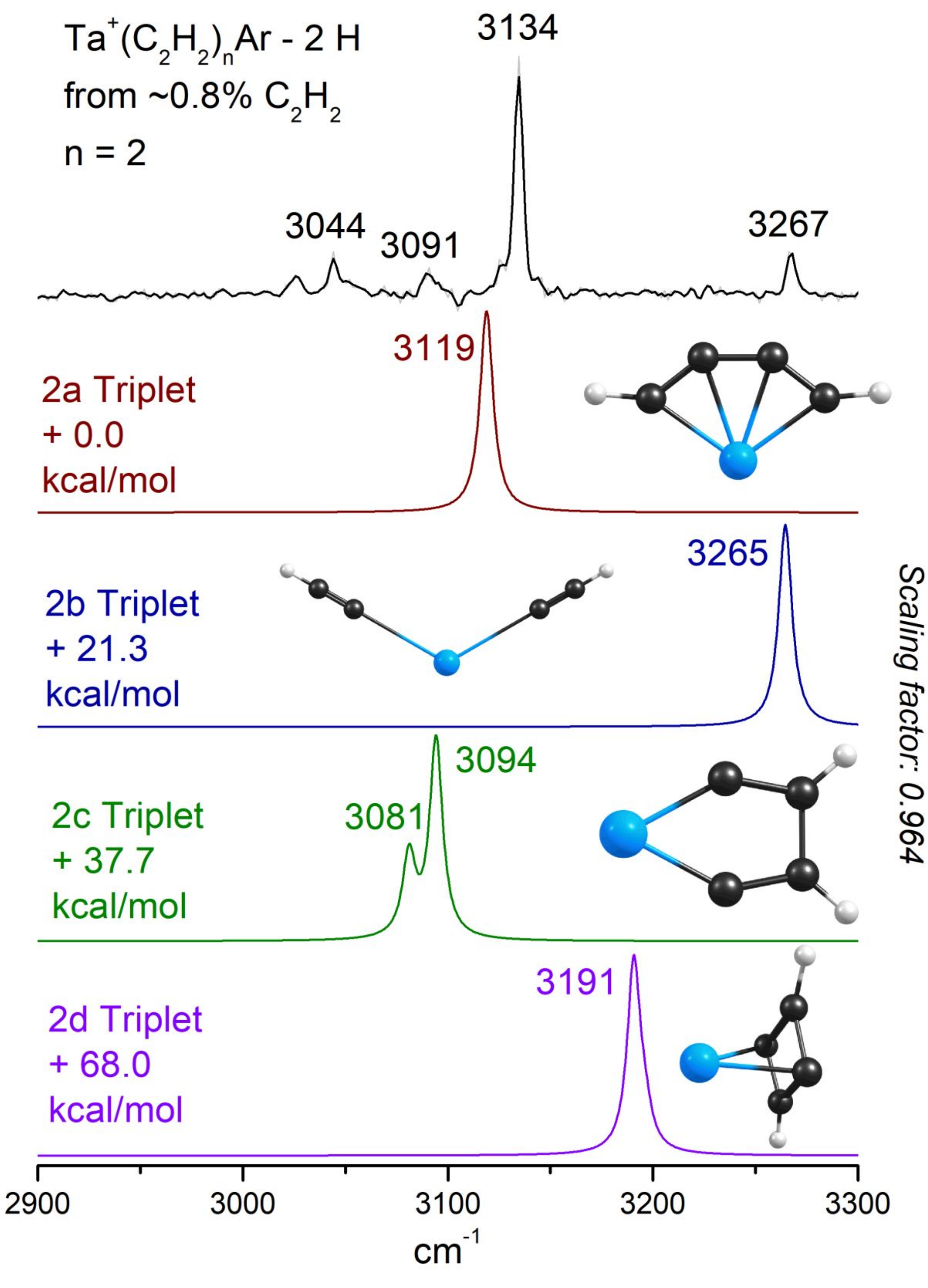

Figure S65. The experimental IR photodissociation spectrum of $Ta^+C_4H_2Ar$ compared with simulated spectra for triplet isomers 2a-2d of $Ta^+C_4H_2$.

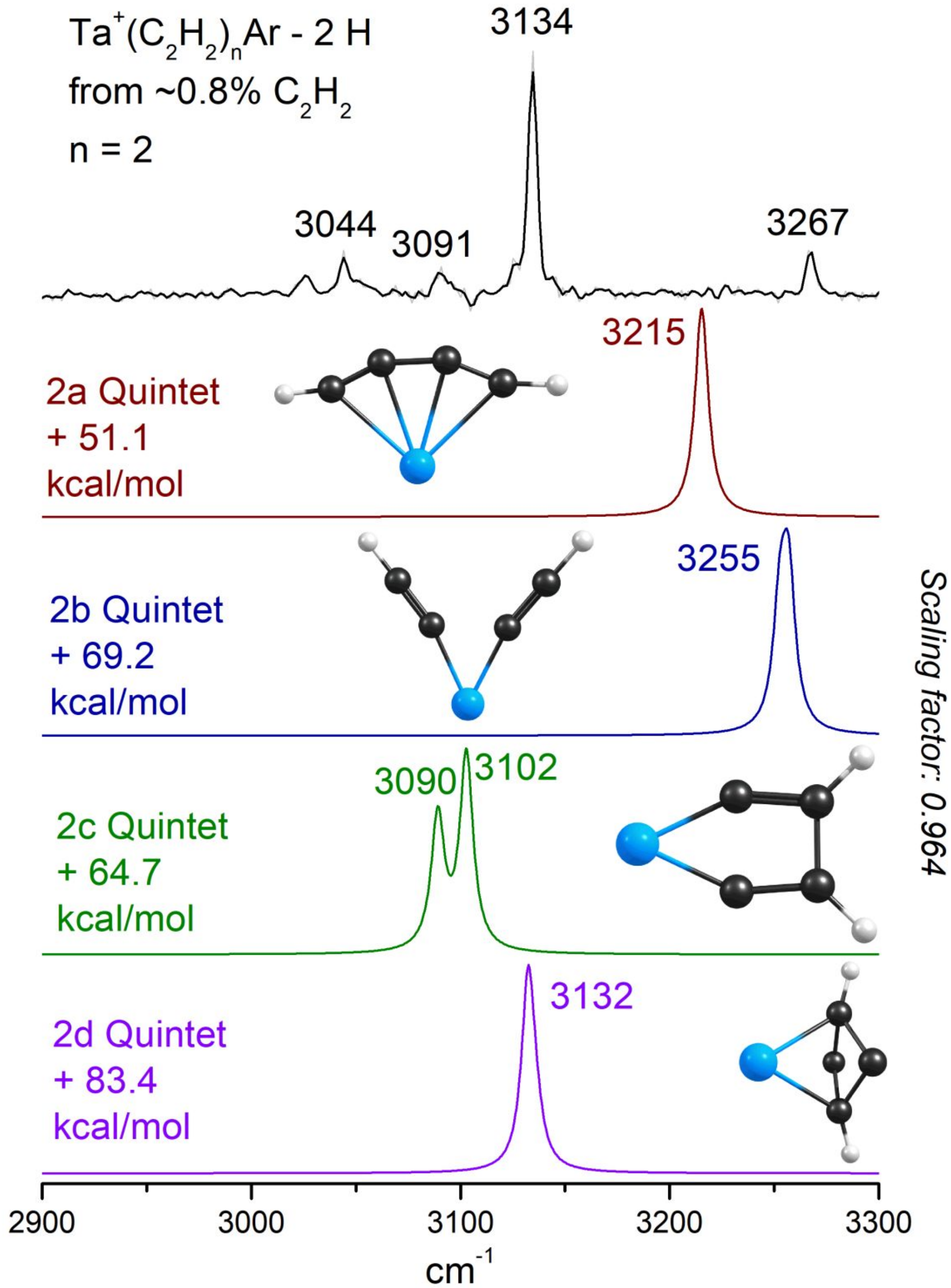

Figure S66. The experimental IR photodissociation spectrum of $Ta^+C_4H_2Ar$ compared with simulated spectra for quintet isomers 2a-2d of $Ta^+C_4H_2$.

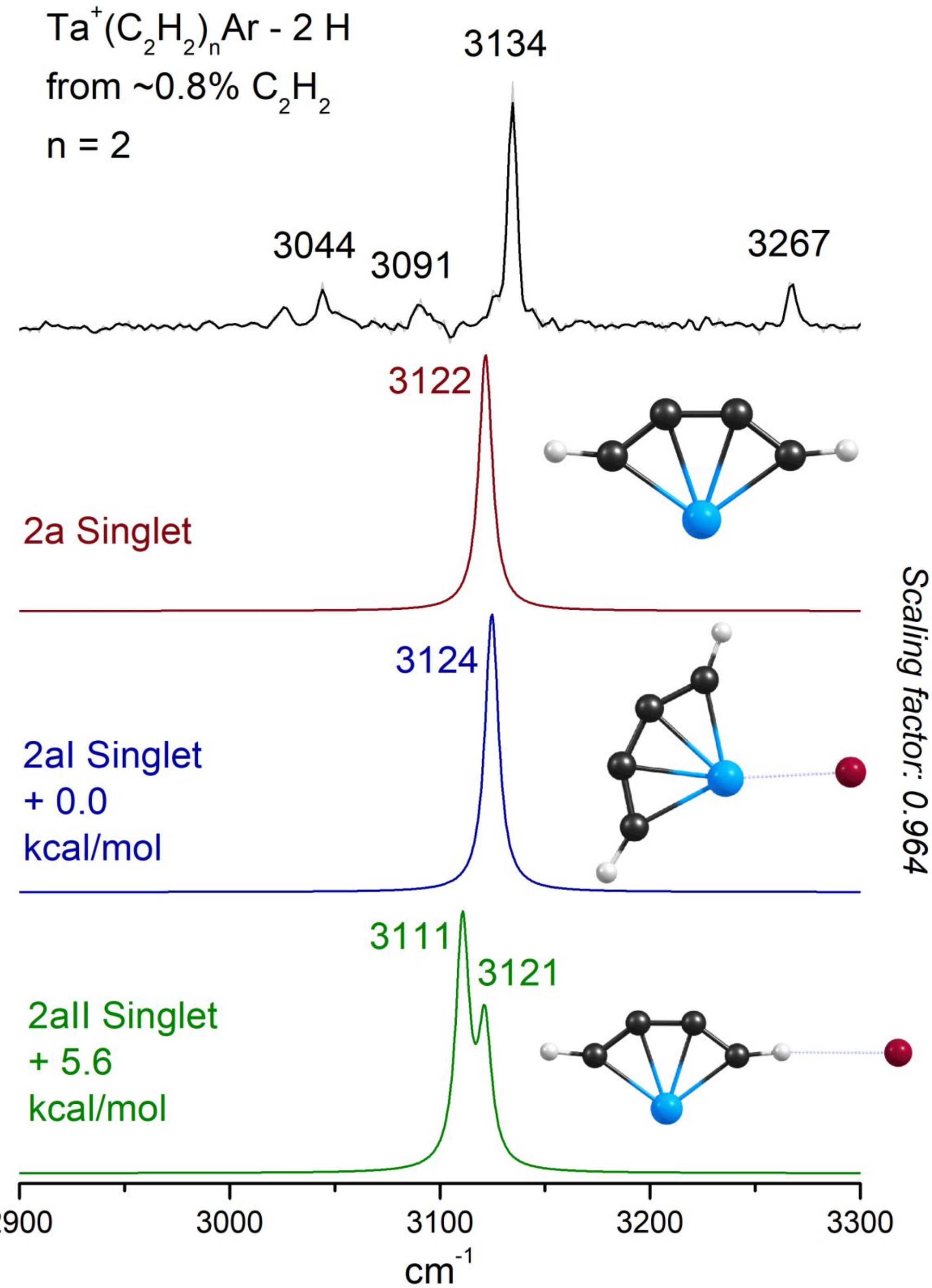

Figure S67. The experimental spectrum measured for $Ta^+C_4H_2Ar$ with simulated spectra for the 2a-singlet and argon-tagged singlet isomers.

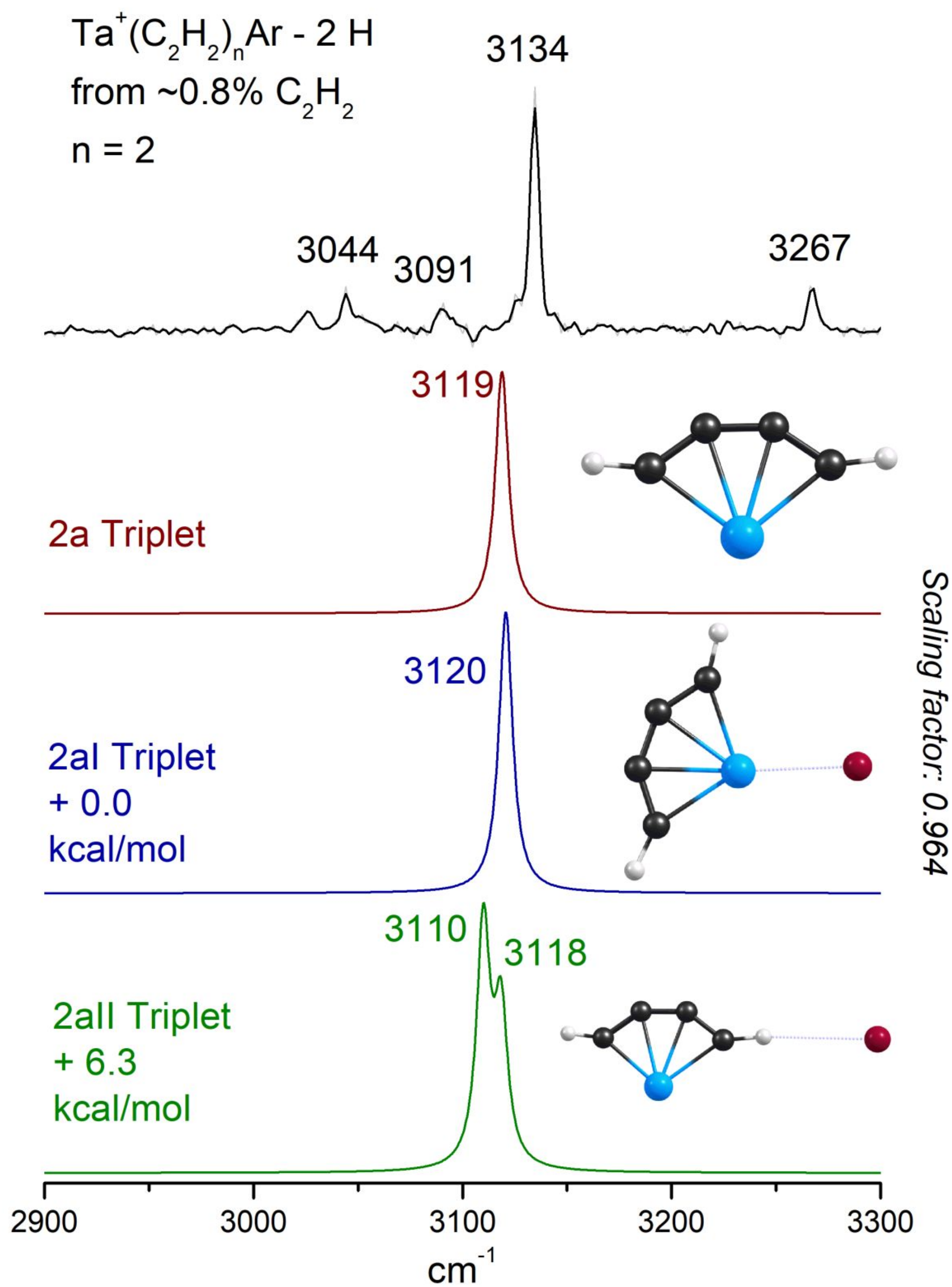

Figure S68. The experimental spectrum measured for $Ta^+C_4H_2Ar$ with simulated spectra for the 2a-triplet and argon-tagged triplet isomers.

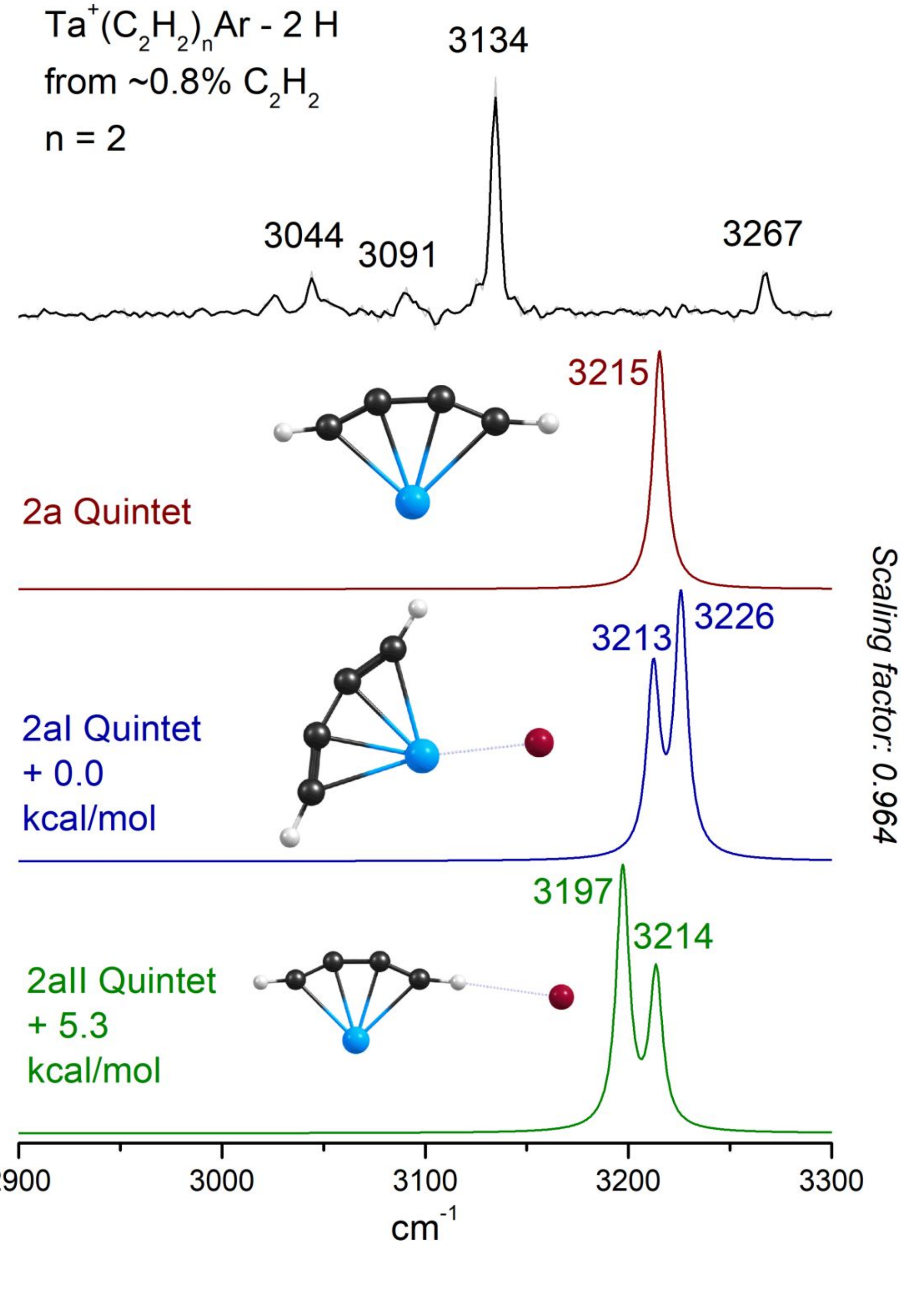

Figure S69. The experimental spectrum measured for $Ta^{+}C_4H_2Ar$ with simulated spectra for the 2a-quintet and argon-tagged quintet isomers.

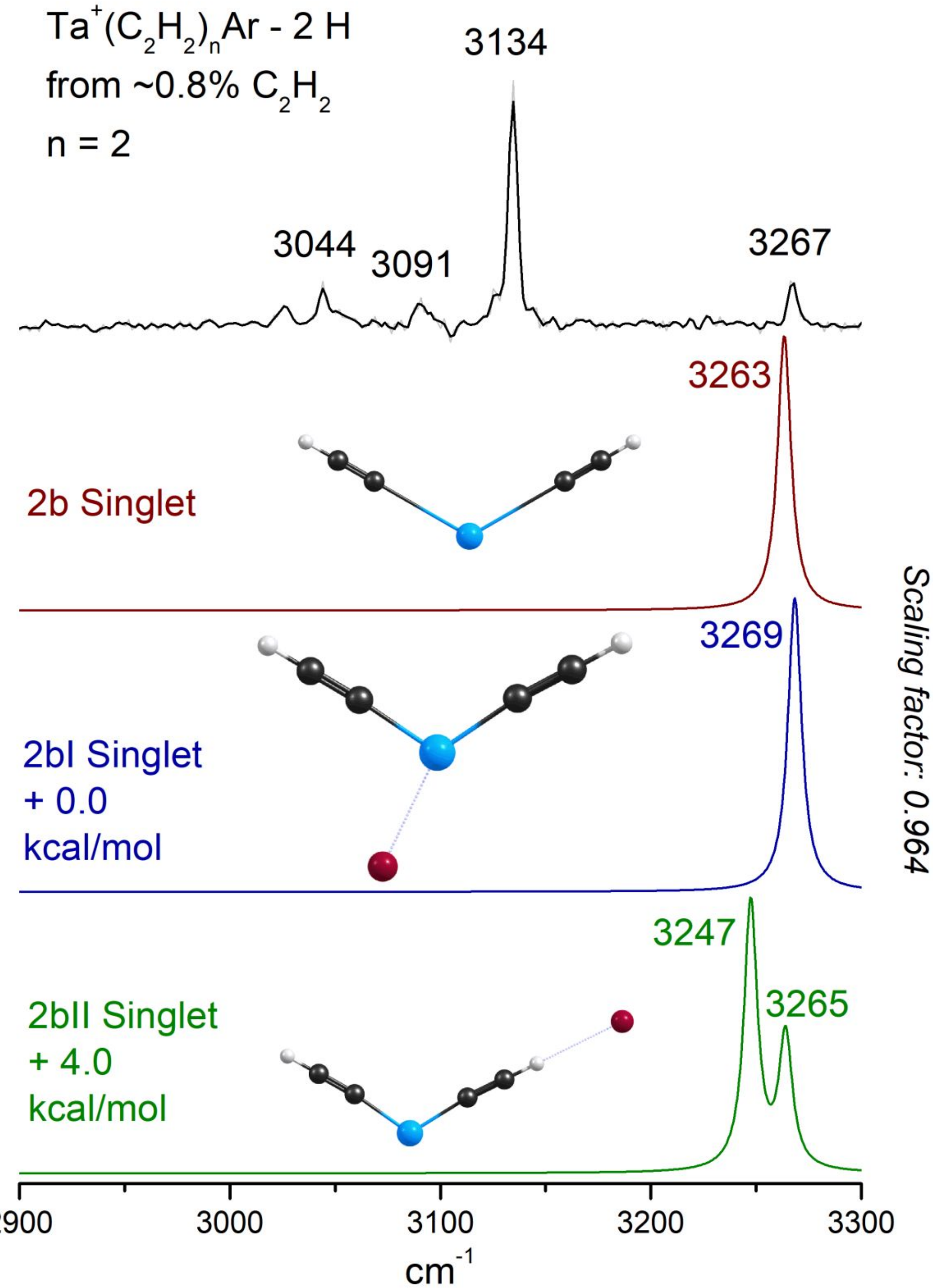

Figure S70. The experimental spectrum measured for $Ta^{+}C_4H_2Ar$ with simulated spectra for the 2b-singlet and argon-tagged singlet isomers.

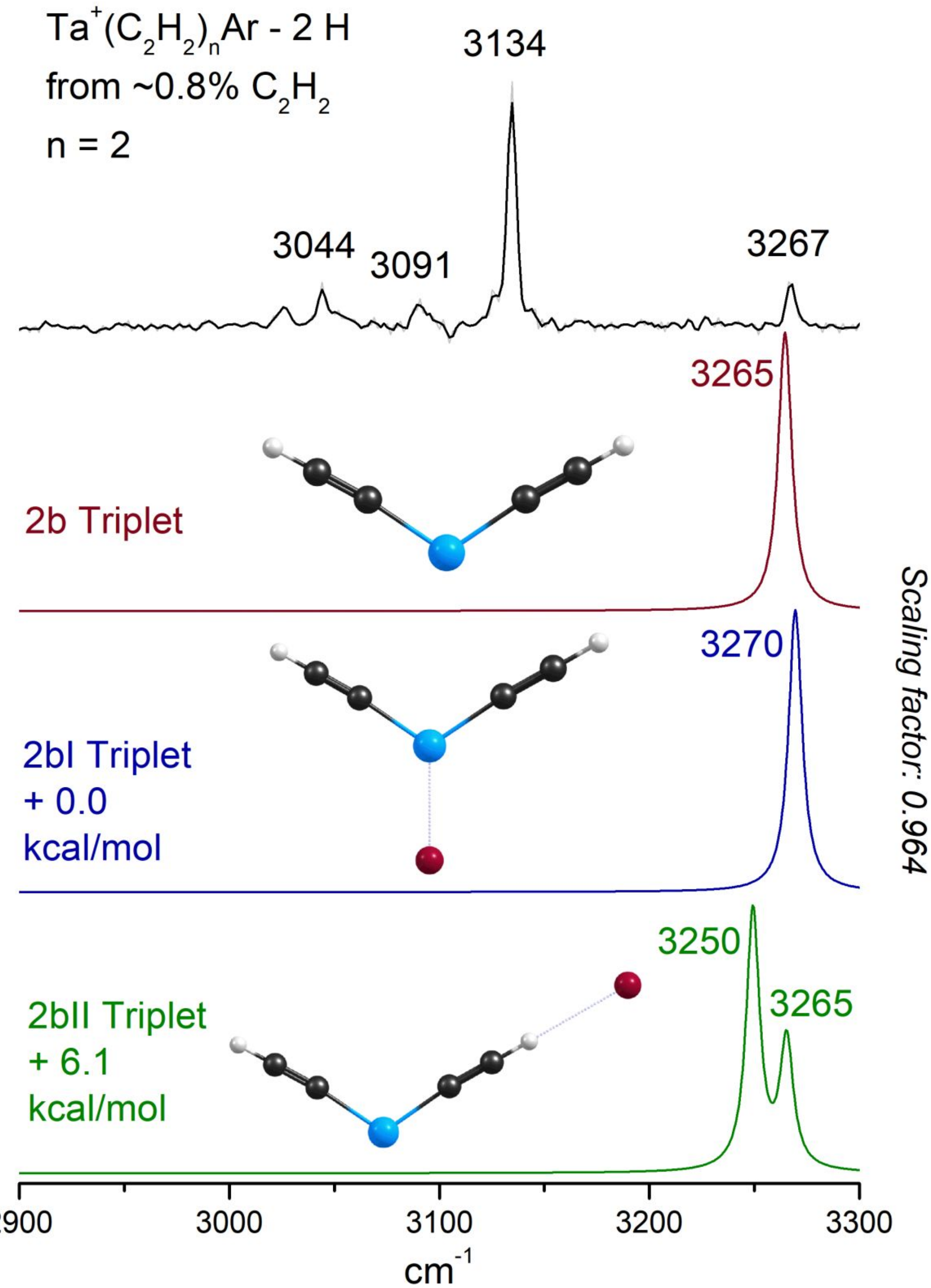

Figure S71. The experimental spectrum measured for $Ta^+C_4H_2Ar$ with simulated spectra for the 2b-triplet and argon-tagged triplet isomers.

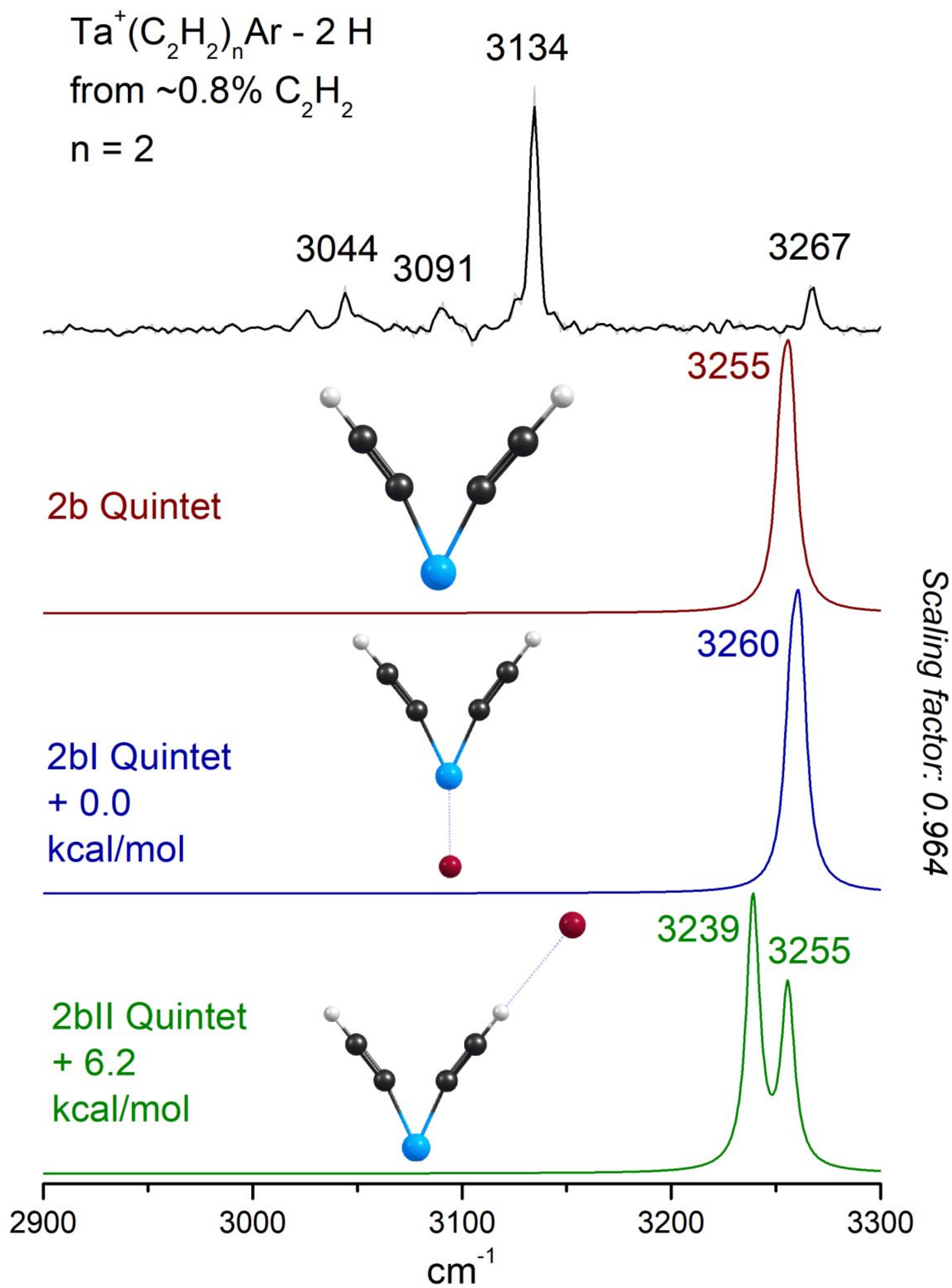

Figure S72. The experimental spectrum measured for $Ta^+C_4H_2Ar$ with simulated spectra for the 2b-quintet and argon-tagged quintet isomers.

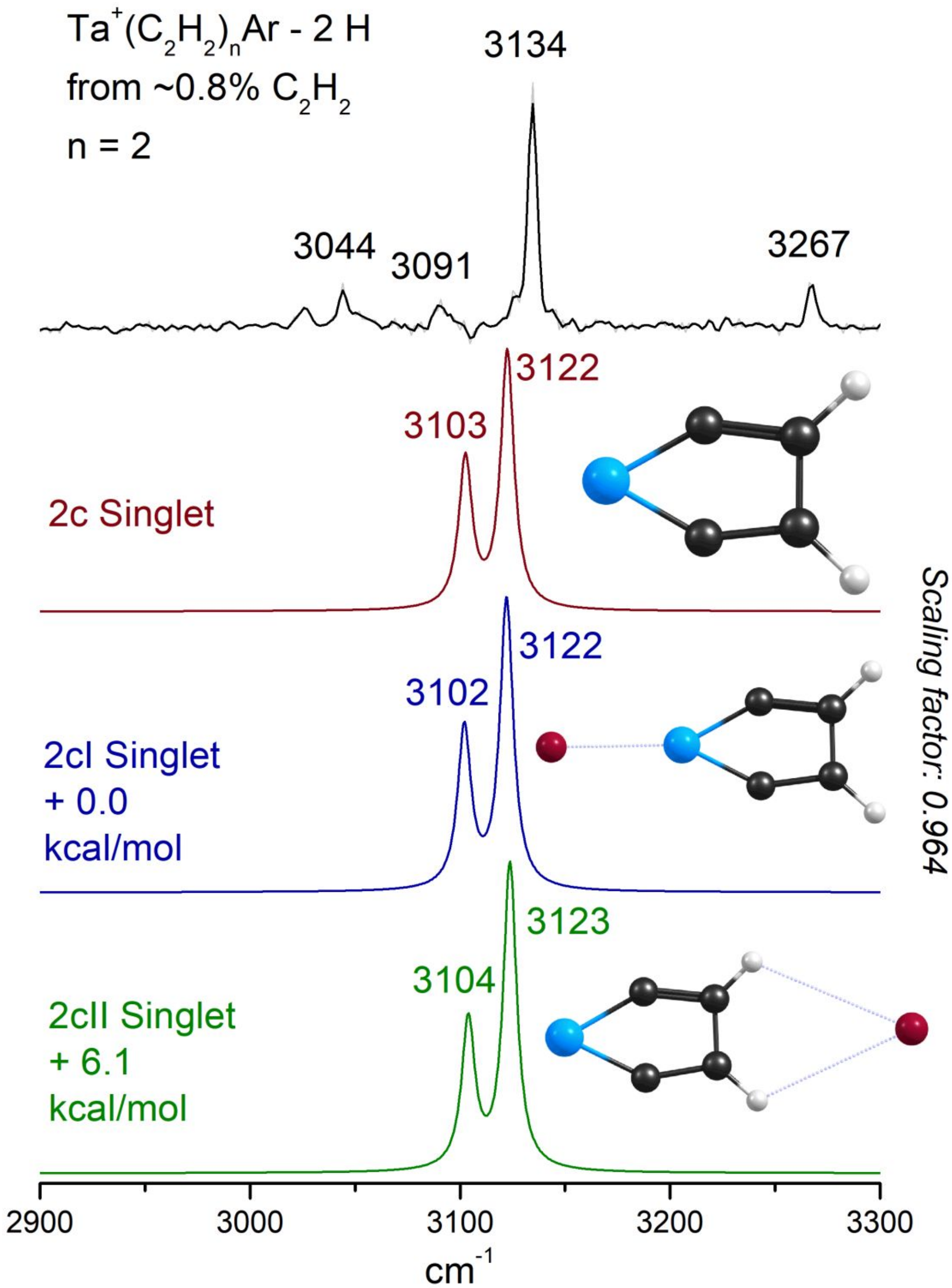

Figure S73. The experimental spectrum measured for $Ta^+C_4H_2Ar$ with simulated spectra for the 2c-singlet and argon-tagged singlet isomers.

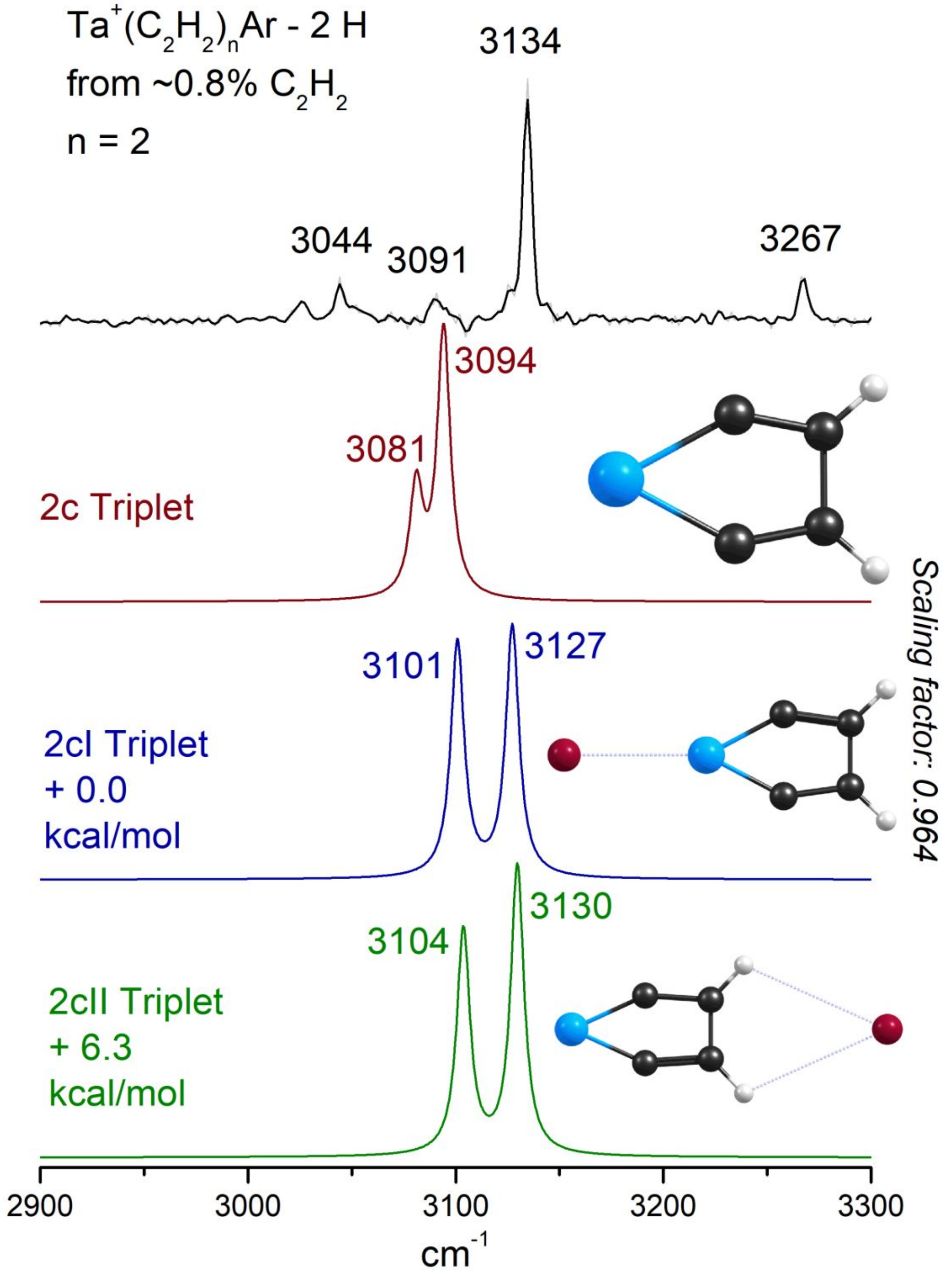

Figure S74. The experimental spectrum measured for $Ta^{+}C_4H_2Ar$ with simulated spectra for the 2c-triplet and argon-tagged triplet isomers.

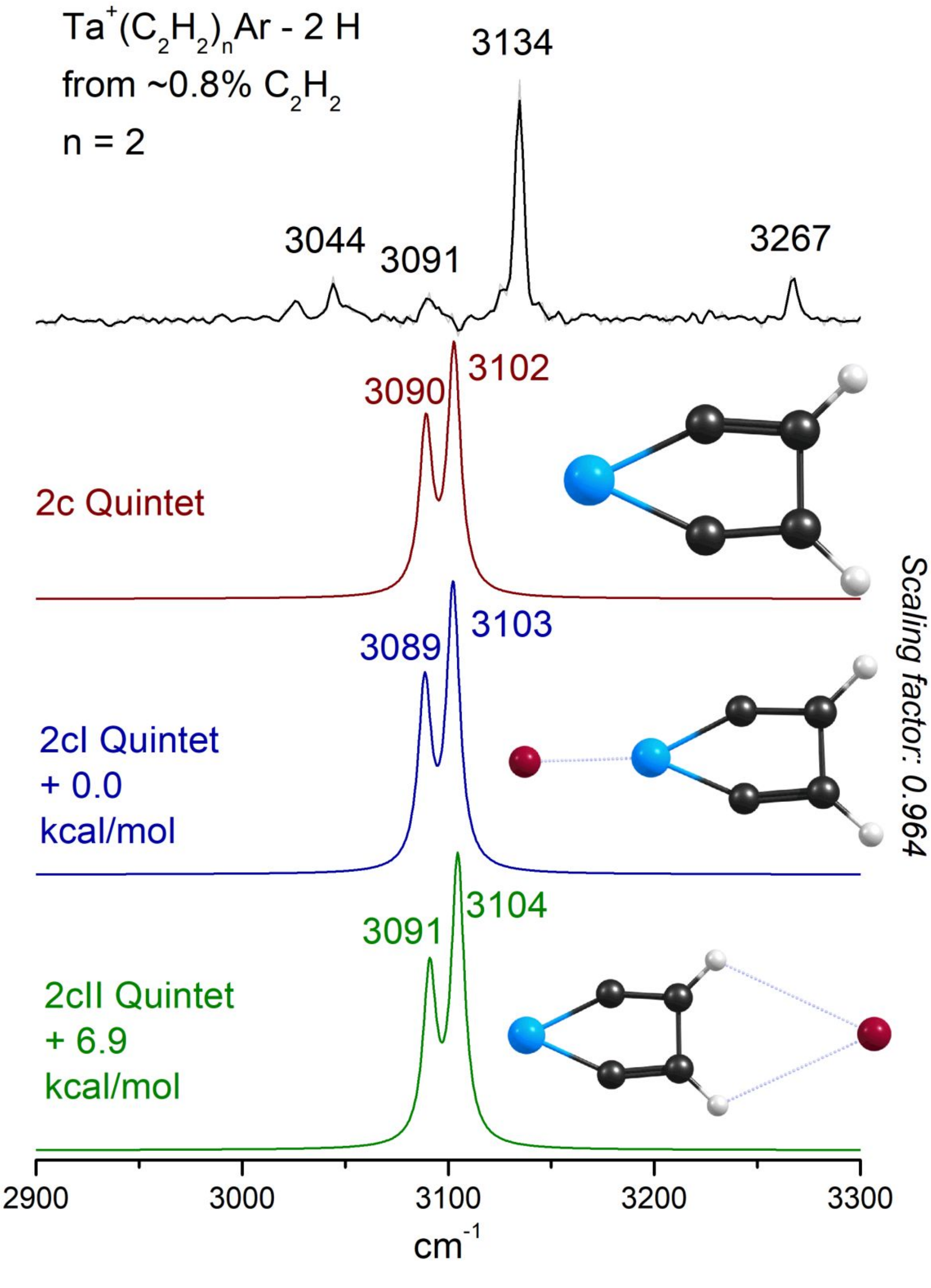

Figure S75. The experimental spectrum measured for $Ta^+C_4H_2Ar$ with simulated spectra for the 2c-quintet and argon-tagged quintet isomers.

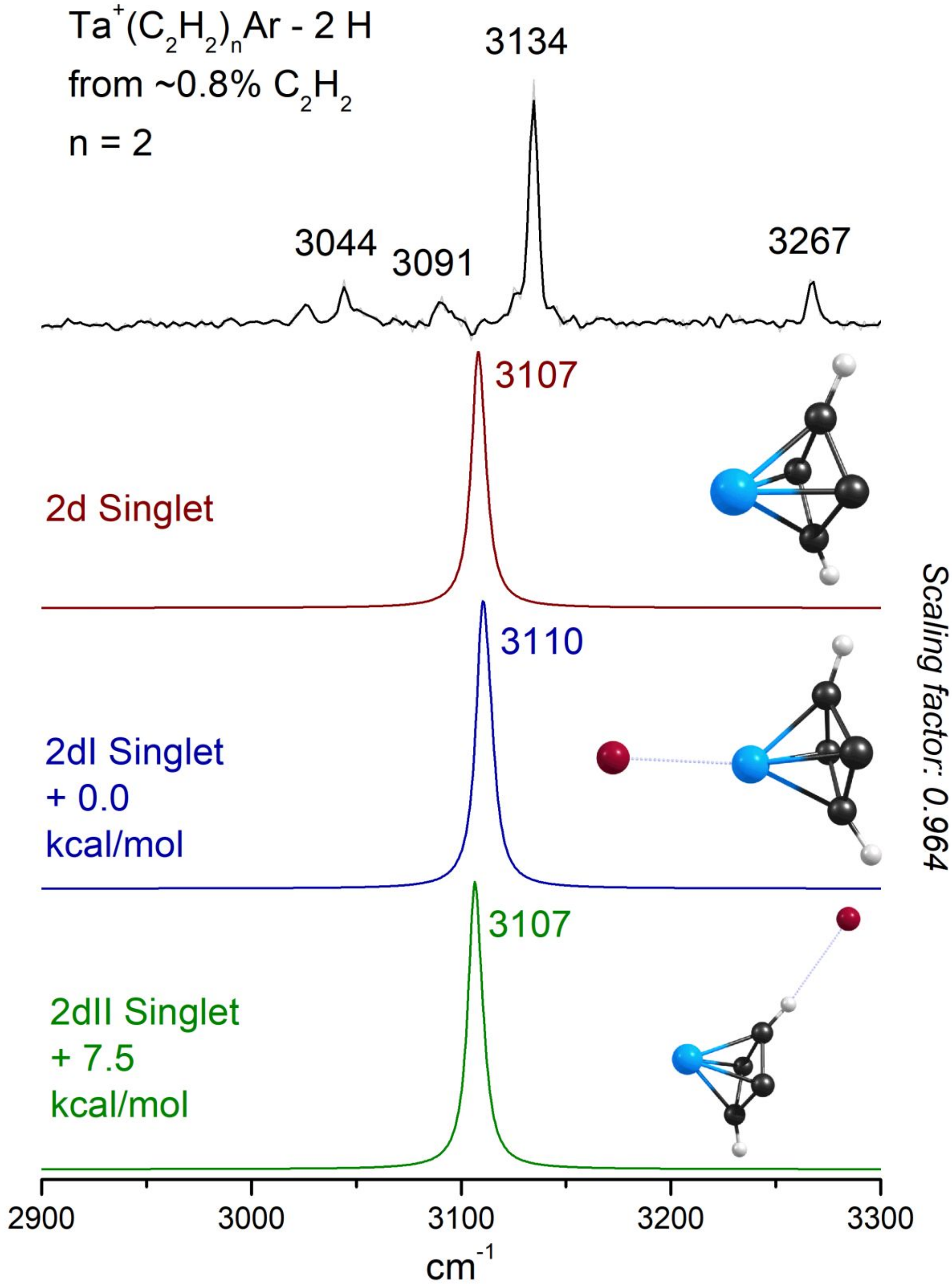

Figure S76. The experimental spectrum measured for $Ta^+C_4H_2Ar$ with simulated spectra for the 2d-singlet and argon-tagged singlet isomers.

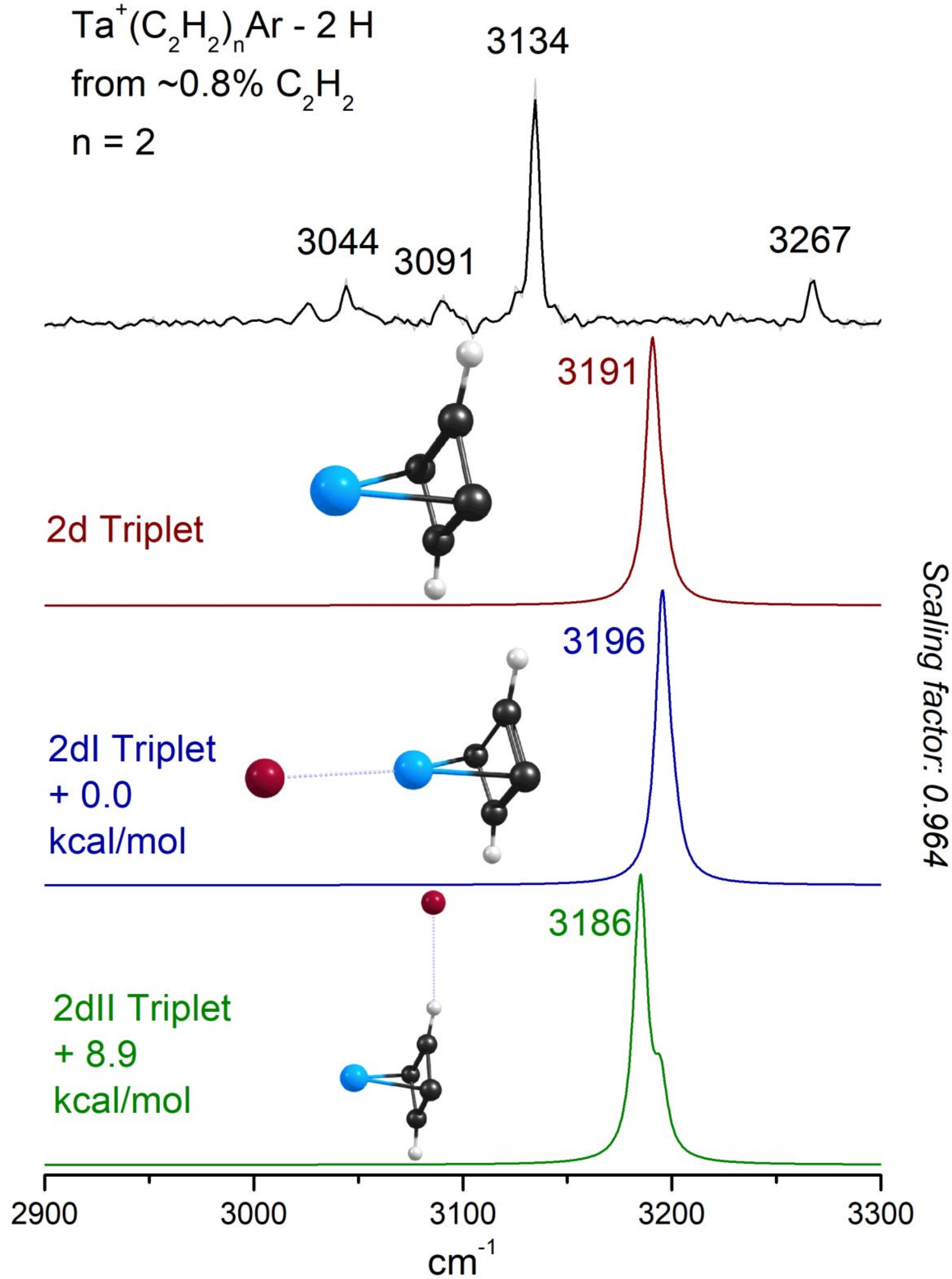

Figure S77. The experimental spectrum measured for $Ta^+C_4H_2Ar$ with simulated spectra for the 2d-triplet and argon-tagged triplet isomers.

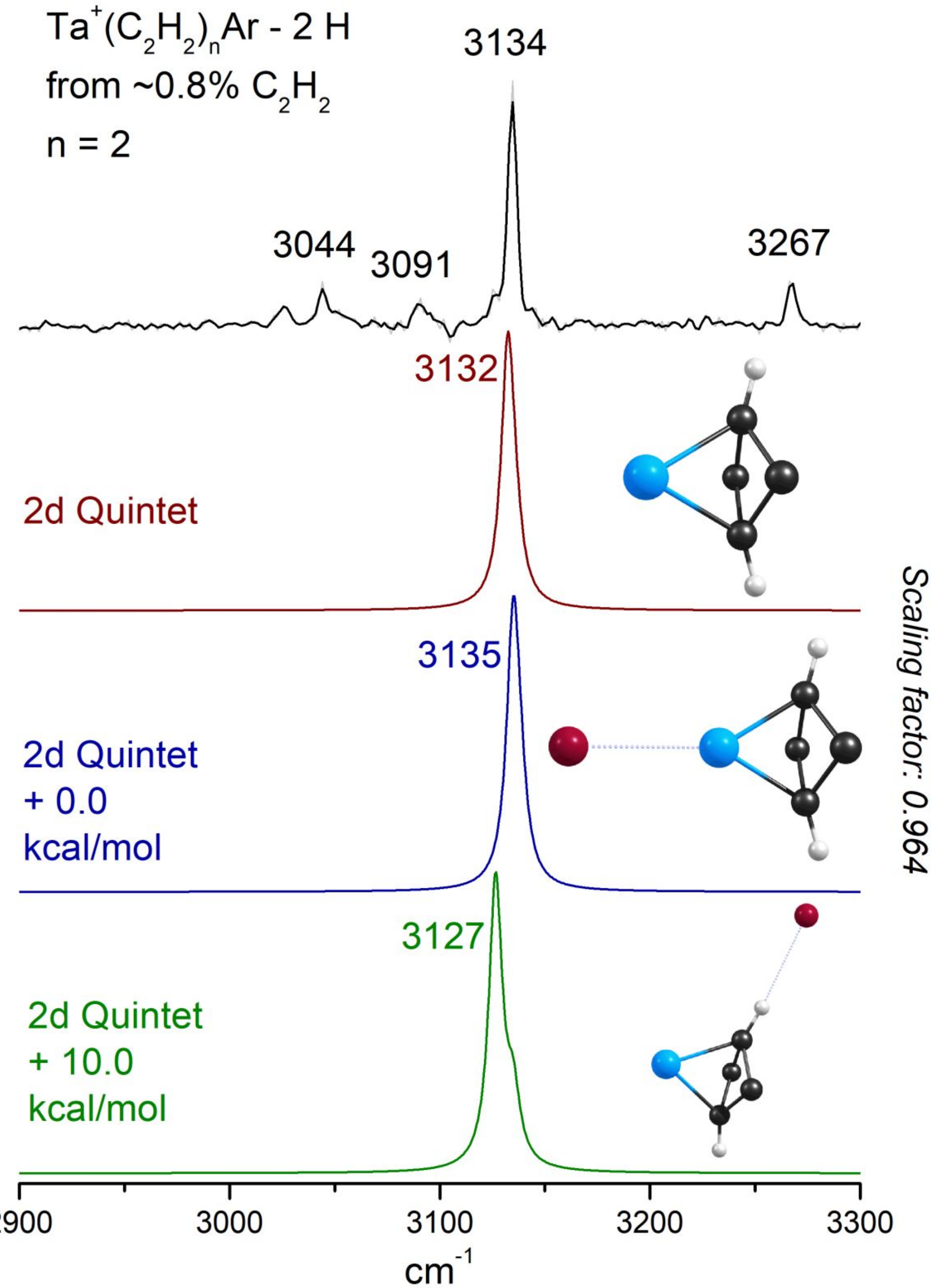

Figure S78. The experimental spectrum measured for $Ta^{+}C_4H_2Ar$ with simulated spectra for the 2d-quintet and argon-tagged quintet isomers.

Table S9. $Ta^+C_6H_4$ electronic energy calculated at the B3LYP/cc-pVTZ(-pp) level with Stuttgardt/Koeln pseudopotential.

Isomer	2s + 1	E (hartree)	Relative E (kcal/mol)
3a	1	-288.151204	+4.6
3a	3	-288.158473	+0.0
3a	5	-288.041229	+73.6
3b	1	-288.150619	+4.9
3b	3	-288.111351	+29.6
3b	5	-288.059610	+62.0
3c	1	-288.144688	+8.7
3c	3	-288.108513	+31.4
3c	5	-288.029647	+80.8
3d	1	-288.115498	+27.0
3d	3	-288.098838	+37.4
3d	5	-288.021734	+85.8
3e	1	-288.116966	+26.0
3e	3	-288.094111	+40.4
3e	5	-288.003632	+97.2
3f	1	-288.115828	+26.8
3f	3	-288.071100	+54.8
3f	5	-287.979449	+112.3
3g	1	-288.053435	+65.9
3g	3	-288.061379	+60.9
3g	5	-287.976541	+114.2
3h	1	-288.136615	+13.7
3h	3	-288.048559	+69.0
3h	5	-288.014527	+90.3
3i	1	-288.064483	+59.0
3i	3	-288.041378	+73.5
3i	5	-288.009181	+93.7
3j	1	-288.037082	+76.2
3j	3	-288.038506	+75.3
3j	5	-287.973405	+116.1
3k	1	-287.982620	+110.3
3k	3	-287.986379	+108.0
3k	5	-287.887380	+170.1

Table S10. Energetics for the formation of $Ta^+C_6H_4$ isomers and transition states from Ta^+ + $3C_2H_2$ at the B3LYP/cc-pVTZ(-pp) level with Stuttgardt/Koeln pseudopotential.

isomer	2s+1	E (hartree)	$[E(Ta^+C_6H_4) + E(H_2)]- [E(Ta^+) + 3C_2H_2]$ (kcal/mol)
$Ta^+ + 3C_2H_2$	1	-289.001008	+15.14
$Ta^+ + 3C_2H_2$	3	-289.025134	0
3a	1	-288.151204	-181.80
3a	3	-288.158473	-186.36
3b	1	-288.150619	-181.43
3b	3	-288.111351	-156.79
3c	1	-288.144688	-177.71
3c	3	-288.108513	-155.01
3d	1	-288.115498	-159.40
3d	3	-288.098838	-148.94
3e	1	-288.116966	-160.32
3e	3	-288.094111	-145.98
3f	1	-288.115828	-159.60
3f	3	-288.0711	-131.54
3g	1	-288.053435	-120.45
3g	3	-288.061379	-125.44
3h	1	-288.136615	-172.65
3h	3	-288.048559	-117.39
3i	1	-288.064483	-127.38
3i	3	-288.041378	-112.89
3j	1	-288.037082	-110.19
3j	3	-288.038506	-111.08
3k	1	-287.98262	-76.01
3k	3	-287.986379	-78.37

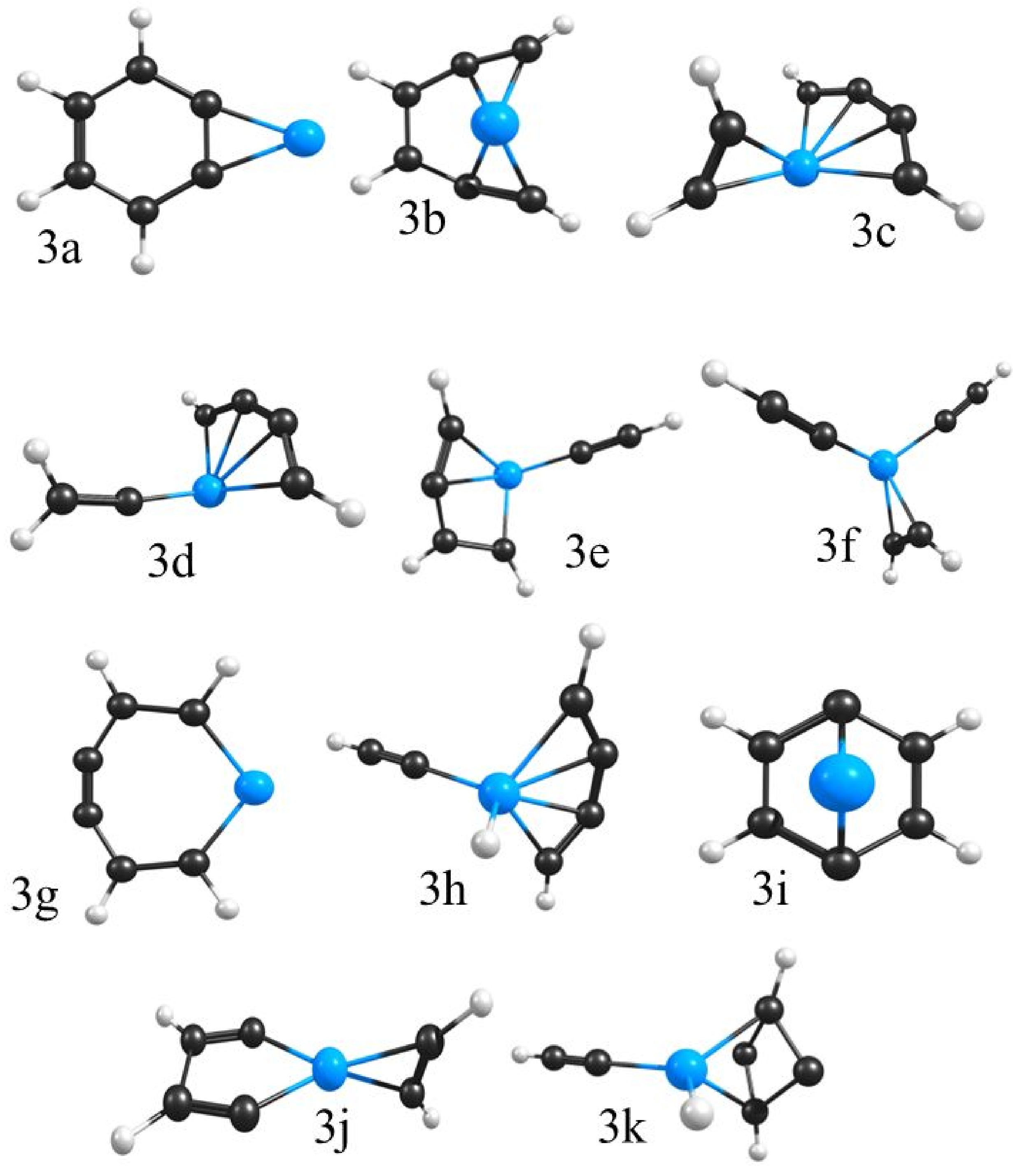

Figure S79. Optimized geometries of $Ta^{+}C_6H_4$.

Table S11. Cartesian coordinates for the optimized geometry of isomer 3a-singlet $Ta^+C_6H_4$ followed by its predicted frequencies (cm^{-1}) and IR intensities (km/mol).

Z	x	y	z
C	-3.0834210	-0.7089620	-0.0000650
C	-3.0834200	0.7089620	-0.0000490
C	-1.9157270	1.4396640	0.0000450
C	-1.9157270	-1.4396640	0.0000420
C	-0.7159630	0.7173800	0.0000960
C	-0.7159630	-0.7173810	0.0001240
H	-4.0352460	-1.2267450	-0.0001540
H	-4.0352460	1.2267460	-0.0001190
H	-1.9276680	2.5225340	0.0000460
H	-1.9276690	-2.5225340	0.0000530
Ta	1.1028380	0.0000000	-0.0000130

Frequency (cm^{-1})	Intensity (km/mol)	Frequency (cm^{-1})	Intensity (km/mol)
145.02	11.0800	1060.40	0.0000
320.76	14.0310	1167.25	2.1261
351.62	9.7333	1198.67	0.5039
406.39	0.0000	1279.58	0.6388
408.46	17.1502	1353.50	0.8245
631.77	12.2329	1402.67	16.6303
641.67	6.6240	1447.13	13.4179
692.83	0.0000	1546.51	4.6400
777.19	43.8288	1579.60	9.4139
866.25	29.4860	3168.32	0.3036
890.81	0.0000	3174.56	0.8721
987.64	10.5489	3183.20	0.5356
1013.33	0.0200	3189.82	0.0420
1018.70	2.1306		

Table S12. Cartesian coordinates for the optimized geometry of isomer 3a-triplet $Ta^+C_6H_4$ followed by its predicted frequencies (cm^{-1}) and IR intensities (km/mol).

Z	x	y	z
C	-3.0833350	-0.7086520	-0.0000160
C	-3.0833350	0.7086520	-0.0000160
C	-1.9154310	1.4370730	0.0000030
C	-1.9154310	-1.4370730	0.0000030
C	-0.7100040	0.7242860	0.0000150
C	-0.7100040	-0.7242860	0.0000150
H	-4.0343420	-1.2277670	0.0000200
H	-4.0343430	1.2277660	0.0000200
H	-1.9275370	2.5200180	0.0000280
H	-1.9275370	-2.5200180	0.0000280
Ta	1.1017670	0.0000000	-0.0000020

Frequency (cm^{-1})	Intensity (km/mol)	Frequency (cm^{-1})	Intensity (km/mol)
147.38	11.4980	1059.35	0.0000
327.40	16.5140	1168.54	1.9707
349.42	10.4941	1199.51	0.0993
403.42	0.0000	1282.44	0.5164
412.82	17.9142	1342.45	0.2993
633.57	14.3698	1409.22	7.5729
637.81	6.4888	1448.26	13.3228
691.74	0.0000	1545.01	8.0783
776.53	44.4869	1583.93	9.5221
845.64	40.4596	3168.29	0.4788
887.85	0.0000	3173.88	0.7389
985.35	10.8135	3182.72	0.6229
1012.63	0.0293	3190.04	0.0689
1020.22	1.7505		

Table S13. Cartesian coordinates for the optimized geometry of isomer 3a-quintet $Ta^+C_6H_4$ followed by its predicted frequencies (cm^{-1}) and IR intensities (km/mol).

Z	x	y	z
C	3.1503350	0.8147560	-0.0000070
C	3.2911220	-0.5883150	-0.0000110
C	2.1963490	-1.4598590	0.0000140
C	1.9171910	1.4356220	0.0000140
C	1.0324080	-0.7391840	0.0000070
C	0.7881760	0.5740440	0.0000030
H	4.0480810	1.4218980	-0.0000220
H	4.2858720	-1.0214800	-0.0000280
H	2.2990600	-2.5354200	0.0000050
H	1.8083390	2.5125270	0.0000130
Ta	-1.1876010	-0.0082180	-0.0000010

Frequency (cm^{-1})	Intensity (km/mol)	Frequency (cm^{-1})	Intensity (km/mol)
70.60	5.8415	1032.01	0.3732
150.25	1.1920	1136.71	2.4858
285.39	7.6807	1176.56	0.2881
388.97	6.9705	1236.28	9.8243
429.39	0.2510	1297.80	19.9339
578.49	10.6555	1406.11	43.9748
582.27	0.0091	1427.49	1.2376
645.41	9.0799	1521.13	43.0026
738.88	70.1531	1573.61	16.3508
852.56	4.6944	3159.13	1.7016
907.59	34.3758	3174.24	6.5894
982.47	0.3161	3190.67	0.7299
1005.09	15.3536	3208.36	8.4024
1008.65	17.4549		

Table S14. Cartesian coordinates for the optimized geometry of isomer 3b-singlet $Ta^+C_6H_4$ followed by its predicted frequencies (cm^{-1}) and IR intensities (km/mol).

Z	x	y	z
C	1.7747890	0.7157920	-0.1628240
C	1.7745180	-0.7164480	-0.1628150
C	0.8625840	-1.3786350	0.6501350
C	0.8631160	1.3783260	0.6501330
C	-0.3742070	-1.5819640	1.0365960
C	-0.3736030	1.5821190	1.0365800
H	2.4536610	1.2523940	-0.8185740
H	2.4531600	-1.2533130	-0.8185870
Ta	-0.4162710	0.0000740	-0.2773260
H	-0.8407020	2.1921140	1.7975410
H	-0.8415260	-2.1917600	1.7975820

Frequency (cm^{-1})	Intensity (km/mol)	Frequency (cm^{-1})	Intensity (km/mol)
223.48	9.5170	952.66	38.2086
285.60	0.2389	965.85	35.0816
294.07	13.1606	1007.35	53.8955
418.97	5.8357	1074.20	17.7841
466.39	2.1889	1143.00	7.9404
547.37	9.3561	1359.73	0.1851
557.85	4.9603	1369.32	2.0924
600.71	5.1548	1741.84	1.9965
631.42	14.0719	1749.74	10.8528
752.11	27.8712	3137.41	3.2996
781.87	3.3963	3147.28	4.3315
813.14	64.2292	3204.19	24.1435
928.67	10.8340	3204.70	28.2883
932.20	22.7138		

Table S15. Cartesian coordinates for the optimized geometry of isomer 3b-triplet $Ta^+C_6H_4$ followed by its predicted frequencies (cm^{-1}) and IR intensities (km/mol).

Z	x	y	z
C	1.7747890	0.7157920	-0.1628240
C	1.7745180	-0.7164480	-0.1628150
C	0.8625840	-1.3786350	0.6501350
C	0.8631160	1.3783260	0.6501330
C	-0.3742070	-1.5819640	1.0365960
C	-0.3736030	1.5821190	1.0365800
H	2.4536610	1.2523940	-0.8185740
H	2.4531600	-1.2533130	-0.8185870
Ta	-0.4162710	0.0000740	-0.2773260
H	-0.8407020	2.1921140	1.7975410
H	-0.8415260	-2.1917600	1.7975820

Frequency (cm^{-1})	Intensity (km/mol)	Frequency (cm^{-1})	Intensity (km/mol)
170.72	0.1201	885.07	86.6001
201.47	7.5197	954.93	13.3691
269.48	5.6259	958.23	5.7940
271.49	19.1223	996.74	1.4186
412.67	32.0462	1130.18	2.1330
523.70	0.3251	1331.96	19.1200
546.45	8.0732	1558.36	3.2524
571.01	3.5447	1706.13	4.3435
609.07	2.4567	1710.76	54.9170
699.27	7.4930	3160.54	55.3816
730.46	31.6530	3161.22	17.5842
768.00	22.4093	3185.08	8.5743
834.40	54.2354	3203.00	8.9611
854.44	63.9313		

Table S16. Cartesian coordinates for the optimized geometry of isomer 3b-quintet $Ta^{+}C_6H_4$ followed by its predicted frequencies (cm^{-1}) and IR intensities (km/mol).

Z	x	y	z
C	-2.2928200	-0.6796090	-0.1915620
C	-2.2928220	0.6795970	-0.1915540
C	-1.0964020	1.3487280	0.1988290
C	-1.0964000	-1.3487350	0.1988250
C	-0.1017380	1.9820950	0.6425590
C	-0.1017240	-1.9820740	0.6425680
H	-3.1462150	-1.2545550	-0.5375810
H	-3.1462200	1.2545360	-0.5375790
Ta	0.6520660	-0.0000010	-0.1251150
H	0.2915380	-2.8156530	1.2053160
H	0.2915190	2.8156980	1.2052730

Frequency (cm^{-1})	Intensity (km/mol)	Frequency (cm^{-1})	Intensity (km/mol)
125.29	2.0260	811.89	42.6214
206.12	2.2397	896.45	1.9413
215.35	2.5914	968.93	10.8569
334.51	17.3030	981.94	9.2515
351.45	0.5989	1171.60	1.1123
369.55	13.5863	1362.90	12.7022
415.33	27.4859	1529.53	4.3442
496.30	3.1213	1870.97	2.5660
544.25	0.0605	1910.27	45.9148
634.48	48.8764	3147.77	8.5354
644.51	39.6089	3163.30	17.6514
706.03	2.6155	3224.86	142.9152
732.53	65.5270	3225.45	49.8392
753.25	23.9860		

Table S17. Cartesian coordinates for the optimized geometry of isomer 3c-singlet $Ta^+C_6H_4$ followed by its predicted frequencies (cm^{-1}) and IR intensities (km/mol).

Z	x	y	z
Ta	-0.1518000	0.0000000	-0.2881460
C	1.0809440	-1.6937500	0.2067820
C	1.8119340	-0.6580600	0.5120930
H	1.1553430	-2.7638400	0.3295210
C	1.8119340	0.6580610	0.5120910
C	1.0809420	1.6937500	0.2067820
H	1.1553410	2.7638400	0.3295210
C	-2.1528230	0.0000000	0.2286880
C	-1.3857710	0.0000000	1.3120900
H	-3.2313990	0.0000000	0.1136920
H	-1.4808570	0.0000000	2.3907460

Frequency (cm^{-1})	Intensity (km/mol)	Frequency (cm^{-1})	Intensity (km/mol)
112.28	1.1275	877.18	0.1756
119.74	0.3226	940.18	36.8411
206.57	3.7192	946.72	127.3194
252.49	4.9656	964.96	0.2701
430.19	6.0098	1090.48	84.6512
451.85	15.2323	1121.78	4.8879
576.23	1.5102	1489.90	7.7595
582.41	10.2257	1612.77	6.5142
599.32	6.3805	1912.10	3.2213
607.52	7.0742	3163.51	18.4489
640.27	25.1102	3198.68	19.6649
727.75	42.8055	3234.24	5.4214
744.74	60.3356	3235.64	97.5409
839.70	10.7510		

Table S18. Cartesian coordinates for the optimized geometry of isomer 3c-triplet $Ta^{+}C_6H_4$ followed by its predicted frequencies (cm^{-1}) and IR intensities (km/mol).

Z	x	y	z
Ta	0.1105630	-0.0000140	-0.0000020
C	-1.2087360	-1.6936650	-0.1126550
C	-1.9963970	-0.6559570	-0.0438680
H	-1.3336670	-2.7632800	-0.1898390
C	-1.9963420	0.6560640	0.0438650
C	-1.2086220	1.6937170	0.1126790
H	-1.3334710	2.7633410	0.1898690
C	2.2960140	-0.2569320	0.5696950
C	2.2959910	0.2569280	-0.5696990
H	2.7523120	-0.6597080	1.4612270
H	2.7523020	0.6597230	-1.4612170

Frequency (cm^{-1})	Intensity (km/mol)	Frequency (cm^{-1})	Intensity (km/mol)
57.88	0.8450	740.76	68.0186
83.47	0.4779	781.88	2.0472
93.88	0.3054	881.24	0.0006
246.09	1.7743	932.60	25.8759
399.19	0.0073	942.11	128.9097
416.22	14.2779	1117.82	3.6434
441.58	9.4694	1609.89	7.8802
457.80	28.0609	1794.99	20.5621
571.50	0.0019	1913.10	0.3058
604.64	0.8978	3203.22	101.0065
635.17	4.9361	3234.76	8.7041
687.31	56.0101	3236.32	95.9489
713.16	0.8631	3272.49	78.2669
722.58	57.2288		

Table S19. Cartesian coordinates for the optimized geometry of isomer 3c-quintet $Ta^+C_6H_4$ followed by its predicted frequencies (cm^{-1}) and IR intensities (km/mol).

Z	x	y	z
Ta	0.2294840	0.0616920	0.0000120
C	-0.7756810	-1.8234490	-0.0000180
C	-1.7974810	-1.0416730	-0.0000230
H	-0.5843570	-2.8871150	-0.0000280
C	-2.3429650	0.1852770	-0.0000180
C	-2.8573140	1.3004930	-0.0000100
H	-3.3347880	2.2555150	-0.0000060
C	2.3856580	-0.3185440	-0.0000140
C	2.3102520	0.9357230	-0.0000430
H	2.9429040	-1.2449080	0.0000010
H	2.6890770	1.9460560	-0.0000740

Frequency (cm^{-1})	Intensity (km/mol)	Frequency (cm^{-1})	Intensity (km/mol)
28.03	0.7699	696.48	38.4659
52.67	0.0252	699.31	0.5093
67.04	1.3050	706.46	64.5919
129.01	4.1319	765.72	12.0284
233.64	5.7321	828.88	52.6748
313.59	1.7950	955.19	9.1467
408.25	6.9337	1758.17	47.8082
417.89	8.1834	1763.92	3.4759
443.40	6.8897	1999.64	30.0877
493.35	3.9365	3194.44	76.1070
541.81	46.1694	3212.48	71.2826
610.59	19.5458	3265.02	113.2964
629.46	47.4935	3394.69	203.1839
693.85	37.4229		

Table S20. Cartesian coordinates for the optimized geometry of isomer 3d-singlet $Ta^+C_6H_4$ followed by its predicted frequencies (cm^{-1}) and IR intensities (km/mol).

Z	x	y	z
Ta	-0.0341320	0.0000020	-0.4026340
C	-0.9909510	-1.6990960	0.5004050
C	-1.5242800	-0.6589020	1.0711790
C	-1.5242760	0.6588910	1.0711790
C	-0.9909530	1.6990920	0.5004160
H	-1.0469030	2.7715330	0.6101600
C	1.7652740	0.0000040	0.2379390
C	2.8978470	-0.0000040	0.9134100
H	2.9282590	-0.0000140	2.0032390
H	-1.0468930	-2.7715390	0.6101380
H	3.8612070	-0.0000060	0.4015560

Frequency (cm^{-1})	Intensity (km/mol)	Frequency (cm^{-1})	Intensity (km/mol)
109.61	1.5832	931.25	34.9898
112.57	4.4601	933.64	131.8037
211.64	13.9769	955.08	10.8937
250.47	2.3837	1017.15	25.2737
319.03	24.2886	1120.10	2.7636
405.50	0.0415	1394.53	7.1921
437.12	5.4392	1623.67	8.9075
468.15	11.8897	1652.94	12.2524
551.68	8.8879	1916.68	3.8832
566.74	0.4879	3067.24	5.3439
596.02	2.8196	3138.64	3.9598
608.87	1.6670	3236.06	7.2469
732.89	68.4620	3237.40	103.3098
873.22	0.0207		

Table S21. Cartesian coordinates for the optimized geometry of isomer 3d-triplet $Ta^+C_6H_4$ followed by its predicted frequencies (cm^{-1}) and IR intensities (km/mol).

Z	x	y	z
Ta	0.0290530	0.0000010	-0.0002310
C	-1.2885520	-1.6888250	0.0002400
C	-2.0898660	-0.6565230	0.0006090
C	-2.0898720	0.6565150	0.0006010
C	-1.2885610	1.6888200	0.0002420
H	-1.4013640	2.7628710	0.0002660
C	2.1195350	0.0000070	0.0001690
C	3.4193160	-0.0000020	0.0005970
H	3.9946120	0.0000050	0.9300050
H	-1.4013500	-2.7628770	0.0002670
H	3.9952280	-0.0000170	-0.9284270

Frequency (cm^{-1})	Intensity (km/mol)	Frequency (cm^{-1})	Intensity (km/mol)
7.72	1.7877	885.45	0.0000
58.79	0.7370	940.18	22.2328
201.18	21.0631	946.02	120.4744
240.82	0.0000	965.58	49.1052
247.34	2.9146	1123.25	6.0024
259.85	5.0435	1332.97	26.3248
424.63	0.4594	1600.91	8.2201
460.33	35.4914	1694.50	2.3249
468.46	16.6509	1911.69	0.8764
574.46	0.0000	3046.55	44.2923
608.15	0.0973	3110.92	18.5135
633.17	4.9595	3231.69	11.9136
740.33	59.6185	3233.03	101.5699
804.63	0.0181		

Table S22. Cartesian coordinates for the optimized geometry of isomer 3d-quintet $Ta^+C_6H_4$ followed by its predicted frequencies (cm^{-1}) and IR intensities (km/mol).

Z	x	y	z
Ta	0.2627240	-0.2336180	-0.2172940
C	-3.8323740	-0.5053430	0.6677840
C	-2.8562040	0.1743130	0.3958470
C	-1.7715340	0.8923610	0.0943050
C	-0.7400960	1.5979520	-0.2136350
H	-0.5213780	2.6522050	-0.3402920
C	2.1696280	0.0367860	0.4457630
C	3.3931800	0.2634630	0.8267510
H	3.9144270	-0.4116780	1.5092510
H	-4.6973180	-1.0821520	0.9090370
H	3.9497960	1.1385460	0.4835990

Frequency (cm^{-1})	Intensity (km/mol)	Frequency (cm^{-1})	Intensity (km/mol)
33.32	1.8568	694.31	39.9061
51.93	1.8873	803.82	42.4290
90.38	1.3574	806.48	12.6895
213.96	1.1321	915.52	3.4090
231.67	21.5580	932.76	42.2799
239.44	5.7731	1332.41	15.7028
278.62	7.7542	1672.45	10.3067
307.98	12.6845	1777.72	15.0082
432.09	6.0491	2047.91	87.8759
466.32	5.5692	3051.33	69.8895
506.73	0.3825	3117.44	18.6146
528.61	9.4907	3164.33	56.0585
629.78	35.4881	3403.62	201.1578
657.65	34.3012		

Table S23. Cartesian coordinates for the optimized geometry of isomer 3e-singlet $Ta^+C_6H_4$ followed by its predicted frequencies (cm^{-1}) and IR intensities (km/mol).

Z	x	y	z
Ta	0.0242160	0.0129890	-0.3786760
C	-0.8664040	-1.4706150	0.6107040
C	-1.9887210	-0.6240250	0.6660340
C	-1.5759510	0.6840510	0.8753580
C	-0.7409330	1.6537180	0.5819900
H	-0.5146850	2.6196300	1.0121560
C	1.8768440	-0.0901300	0.4744130
C	3.0404080	-0.1489620	0.8290300
H	4.0648190	-0.1983590	1.1302040
H	-0.7745190	-2.5167460	0.8762890
H	-3.0148190	-0.8769200	0.3995270

Frequency (cm^{-1})	Intensity (km/mol)	Frequency (cm^{-1})	Intensity (km/mol)
86.92	4.2607	837.56	16.6581
100.05	7.0115	896.01	22.9583
206.08	16.5178	965.54	33.9203
253.99	12.5873	1000.64	52.8781
313.24	2.5290	1104.54	11.3606
372.99	6.3535	1210.07	13.7969
484.77	15.9863	1365.02	5.2659
504.39	47.6056	1734.20	6.8568
557.76	1.6095	2055.36	307.1402
602.53	18.6556	3092.92	0.7083
691.80	30.5507	3190.75	19.8712
749.74	33.6824	3208.98	32.8052
787.70	70.2857	3397.36	77.9572
793.36	26.5829		

Table S24. Cartesian coordinates for the optimized geometry of isomer 3e-triplet $Ta^+C_6H_4$ followed by its predicted frequencies (cm^{-1}) and IR intensities (km/mol).

Z	x	y	z
Ta	0.1043180	0.0121550	-0.2153420
C	-1.1193220	-1.5084930	0.4362370
C	-2.2235300	-0.6946770	0.2797760
C	-1.9543120	0.6860380	0.4052650
C	-1.1708800	1.6851420	0.3595500
H	-1.0445380	2.7061860	0.6877470
C	2.0900400	-0.0361780	0.3124650
C	3.2917260	-0.1015110	0.5092540
H	4.3452110	-0.1571660	0.6820310
H	-1.1734600	-2.5877380	0.5282940
H	-3.2247720	-1.0305430	0.0066420

Frequency (cm^{-1})	Intensity (km/mol)	Frequency (cm^{-1})	Intensity (km/mol)
55.86	2.8134	787.18	26.4491
71.19	3.2311	804.16	67.6747
185.12	17.2594	921.08	3.6191
194.42	21.6975	956.20	27.9150
241.26	5.3624	1085.36	4.2515
289.08	0.7051	1247.52	25.4264
393.96	10.4090	1370.58	8.4336
480.86	6.8092	1826.01	20.8296
493.10	74.0524	2044.37	369.8111
546.41	4.3682	3084.28	16.5536
671.56	70.3010	3177.57	18.9834
699.30	14.2104	3230.61	105.5070
740.84	33.3273	3395.76	74.7077
770.46	33.2384		

Table S25. Cartesian coordinates for the optimized geometry of isomer 3e-quintet $Ta^+C_6H_4$ followed by its predicted frequencies (cm^{-1}) and IR intensities (km/mol).

Z	x	y	z
Ta	0.1368650	-0.0439550	0.2204320
C	-1.9363400	-1.3988710	0.1234970
C	-1.9630630	-0.4934150	-0.8943720
C	-1.8302360	0.8701620	-0.5576190
C	-1.1877180	1.7926770	-0.0088010
H	-1.1387910	2.8161670	0.3211220
C	2.0949390	-0.0816620	-0.4068830
C	3.2990940	-0.0720190	-0.5980850
H	4.3545090	-0.0649810	-0.7660650
H	-2.0650860	-2.4471700	0.3506620
H	-2.0018290	-0.7965600	-1.9436640

Frequency (cm^{-1})	Intensity (km/mol)	Frequency (cm^{-1})	Intensity (km/mol)
53.82	1.6901	773.08	36.6349
60.77	1.8851	778.68	40.0523
156.89	26.1626	796.79	36.1100
183.64	25.4318	840.35	6.0483
194.88	2.2749	1037.72	7.0206
225.77	4.1043	1200.08	11.7659
278.90	5.4972	1403.46	13.3473
343.83	7.1476	1928.17	18.7230
492.03	61.8276	2042.30	324.1905
505.60	2.2196	3059.65	31.5259
521.32	26.0604	3233.27	91.8580
632.09	3.6196	3278.83	127.3300
707.79	57.1114	3397.96	74.6133
767.76	31.6785		

Table S26. Cartesian coordinates for the optimized geometry of isomer 3f-singlet $Ta^{+}C_6H_4$ followed by its predicted frequencies (cm^{-1}) and IR intensities (km/mol).

Z	x	y	z
Ta	-0.0000010	0.0932790	-0.2599430
C	0.0000040	2.1016160	0.0668550
C	0.0000030	1.4467850	1.2239420
H	0.0000040	3.1485100	-0.2155370
H	0.0000080	1.6585020	2.2850960
C	2.7488750	-1.5225080	0.4308270
C	1.7396920	-0.8698300	0.2332870
H	3.6338320	-2.0994000	0.5960180
C	-2.7488730	-1.5225100	0.4308260
C	-1.7396930	-0.8698210	0.2333030
H	-3.6338360	-2.0993960	0.5959950

Frequency (cm^{-1})	Intensity (km/mol)	Frequency (cm^{-1})	Intensity (km/mol)
63.19	2.9861	755.69	59.1362
81.47	5.4640	805.34	11.2057
115.34	2.9299	805.66	43.1201
143.83	6.7867	829.89	7.7660
188.15	44.9034	961.42	1.2513
228.37	0.2154	1076.66	83.9065
257.62	0.2627	1475.94	4.1996
287.90	14.0434	2051.43	576.6188
497.52	22.8213	2063.20	179.6930
507.94	95.6979	3171.96	29.4589
601.29	17.4257	3211.06	31.8928
653.50	26.7795	3394.86	100.8527
737.78	97.5464	3395.11	44.2036
755.39	11.4093		

Table S27. Cartesian coordinates for the optimized geometry of isomer 3f-triplet $Ta^{+}C_6H_4$ followed by its predicted frequencies (cm^{-1}) and IR intensities (km/mol).

Z	x	y	z
Ta	0.0000010	0.0495300	0.0000370
C	-0.1275410	2.2259530	-0.6091810
C	0.1275690	2.2260450	0.6090040
H	-0.3439320	2.6317850	-1.5855410
H	0.3439690	2.6319890	1.5853150
C	2.8158760	-1.6140300	-0.0282420
C	1.7685370	-0.9910400	-0.0459060
H	3.7318920	-2.1654880	-0.0131470
C	-2.8158970	-1.6139960	0.0281020
C	-1.7685530	-0.9910170	0.0458480
H	-3.7319180	-2.1654450	0.0129390

Frequency (cm^{-1})	Intensity (km/mol)	Frequency (cm^{-1})	Intensity (km/mol)
25.48	0.3469	728.56	78.7751
57.59	0.0013	775.88	2.8054
63.62	5.6183	778.55	47.1790
85.81	7.7929	778.69	18.0353
149.51	52.6070	798.14	0.6511
172.01	0.4072	798.28	55.5822
190.87	2.1023	1814.79	20.3646
253.37	14.9462	2049.53	665.3435
391.80	8.5076	2061.03	206.8284
410.49	0.0307	3209.21	134.9204
493.89	35.8252	3284.09	101.2978
500.63	108.4263	3394.27	99.4995
691.01	61.1984	3394.43	34.6050
704.83	0.3910		

Table S28. Cartesian coordinates for the optimized geometry of isomer 3f-quintet $Ta^{+}C_6H_4$ followed by its predicted frequencies (cm^{-1}) and IR intensities (km/mol).

Z	x	y	z
Ta	-0.3318120	-0.1869640	-0.2957010
C	-2.2119840	-0.0284070	0.9480870
C	-1.7283540	1.1184660	0.9066180
H	-2.9135910	-0.7935140	1.2424600
H	-1.6169800	2.1711670	1.1034520
C	2.2662130	1.8445330	-0.0199490
C	1.2686760	1.1014140	-0.0917700
H	3.0965660	2.5167610	0.0169070
C	2.5978660	-1.2660280	0.8405370
C	1.4977920	-0.8676450	0.4165340
H	3.5150420	-1.6600520	1.2230130

Frequency (cm^{-1})	Intensity (km/mol)	Frequency (cm^{-1})	Intensity (km/mol)
59.13	0.7110	680.40	19.1981
80.49	3.3548	686.47	9.4982
105.69	1.0926	688.54	46.6729
157.95	0.1787	690.17	84.2348
221.77	0.7160	717.78	66.2156
258.97	1.1938	755.46	24.5396
267.14	28.0200	1784.10	70.7718
298.27	19.4391	1807.01	64.1768
376.42	4.2437	1835.23	2.2587
393.09	7.0573	3220.16	117.7052
432.45	1.0470	3297.75	105.7909
441.02	0.2891	3379.63	76.4330
587.63	30.0574	3382.13	251.2528
612.52	31.3743		

Table S29. Cartesian coordinates for the optimized geometry of isomer 3g-singlet $Ta^+C_6H_4$ followed by its predicted frequencies (cm^{-1}) and IR intensities (km/mol).

Z	x	y	z
C	2.4474160	0.6136110	0.1610170
C	2.4474260	-0.6136090	0.1610120
C	1.7501090	-1.7810380	-0.0489030
C	1.7501020	1.7810390	-0.0489030
C	0.3739750	-1.6007890	-0.2354130
C	0.3739650	1.6008040	-0.2354060
H	2.2198050	-2.7564050	-0.1257710
H	2.2198020	2.7564030	-0.1257860
Ta	-0.8066370	-0.0000020	0.0394780
H	-0.2065220	2.4743160	-0.5753870
H	-0.2065360	-2.4742900	-0.5753830

Frequency (cm^{-1})	Intensity (km/mol)	Frequency (cm^{-1})	Intensity (km/mol)
110.84	3.7714	944.66	0.1527
170.92	1.2043	1043.36	0.9255
253.29	1.5601	1082.73	8.5310
300.84	0.0734	1247.33	5.3978
313.96	16.8108	1252.23	47.2409
468.98	0.9857	1293.95	11.9967
505.78	1.0905	1374.28	0.0026
506.12	1.8862	1443.83	1.4183
583.65	34.3574	2129.07	2.0993
603.74	16.6780	2979.50	2.8744
661.16	11.2981	2980.31	10.0362
694.88	65.7716	3153.75	5.1968
897.56	3.8114	3154.69	4.8349
938.98	3.7858		

Table S30. Cartesian coordinates for the optimized geometry of isomer 3g-triplet $Ta^+C_6H_4$ followed by its predicted frequencies (cm^{-1}) and IR intensities (km/mol).

Z	x	y	z
C	-2.4383800	0.6132040	-0.1845780
C	-2.4383650	-0.6132120	-0.1846200
C	-1.7495460	-1.7825640	0.0544530
C	-1.7495490	1.7825600	0.0544390
C	-0.3841280	-1.6010450	0.2746210
C	-0.3841320	1.6010480	0.2746300
H	-2.2256120	-2.7548540	0.1312540
H	-2.2256100	2.7548560	0.1312100
Ta	0.8074420	0.0000010	-0.0449120
H	0.1862640	2.4690350	0.6412100
H	0.1862830	-2.4690230	0.6412070

Frequency (cm^{-1})	Intensity (km/mol)	Frequency (cm^{-1})	Intensity (km/mol)
93.51	3.7327	952.05	0.2959
172.01	1.8551	1050.53	0.5661
263.55	1.6057	1086.57	9.8983
303.28	0.0002	1256.55	8.5754
325.42	17.4411	1259.88	49.2204
474.79	0.2803	1311.01	9.2848
493.77	1.4296	1377.71	0.4681
509.98	4.0699	1445.31	0.5120
589.46	39.4879	2138.23	1.0719
609.81	19.2307	2990.12	3.8573
671.64	9.9018	2990.50	11.3454
706.91	77.8618	3153.69	4.3461
897.66	4.4262	3154.55	4.9595
946.81	5.5105		

Table S31. Cartesian coordinates for the optimized geometry of isomer 3g-quintet $Ta^{+}C_6H_4$ followed by its predicted frequencies (cm^{-1}) and IR intensities (km/mol).

Z	x	y	z
C	2.8319330	0.6924270	0.0000010
C	2.8319360	-0.6924270	0.0000000
C	1.8347540	-1.6457550	0.0000000
C	1.8347500	1.6457560	0.0000010
C	0.4704170	-1.3924960	0.0000000
C	0.4704130	1.3925050	0.0000000
H	2.1756560	-2.6828620	0.0000000
H	2.1756570	2.6828610	0.0000010
Ta	-0.8982430	-0.0000010	0.0000000
H	-0.2123850	2.2781720	0.0000010
H	-0.2123840	-2.2781580	-0.0000010

Frequency (cm^{-1})	Intensity (km/mol)	Frequency (cm^{-1})	Intensity (km/mol)
125.0025	0	1044.4651	0.2448
156.2178	0.1409	1129.8083	3.4045
163.1068	0.059	1232.031	9.2559
275.9853	2.1735	1307.1901	0.5071
378.6823	3.8218	1312.7508	22.1262
450.6732	0	1393.2764	0.8216
472.7743	2.332	1454.5247	42.9367
505.4721	4.1768	1480.3036	57.4237
588.4795	1.6191	1512.0765	4.3724
599.2856	0	1532.1227	1.4511
634.9265	130.3499	3047.4278	1.3893
805.7731	1.0265	3048.3918	7.8514
812.2524	7.5157	3137.6222	0.2623
837.6079	2.9016	3139.5318	0.0077
917.0542	0	3157.4354	0.0008
1003.6101	3.1499	3172.3331	0.0028
1011.6922	0		

Table S32. Cartesian coordinates for the optimized geometry of isomer 3h-singlet $Ta^+C_6H_4$ followed by its predicted frequencies (cm^{-1}) and IR intensities (km/mol).

Z	x	y	z
Ta	0.0222470	-0.0000050	-0.2927260
C	-1.0772620	-1.7010500	0.3922330
C	-1.7352790	-0.6602060	0.8107170
C	-1.7352660	0.6602510	0.8107060
C	-1.0772280	1.7010750	0.3922030
H	-1.1521990	2.7762550	0.4565140
C	1.9461000	-0.0000220	0.4199420
C	3.1175880	0.0000110	0.7523810
H	4.1488530	0.0000380	1.0355830
H	-1.1522550	-2.7762280	0.4565630
H	-0.1003780	-0.0000200	-2.0487880

Frequency (cm^{-1})	Intensity (km/mol)	Frequency (cm^{-1})	Intensity (km/mol)
78.50	5.1006	785.48	29.7901
85.60	2.5983	786.86	28.7062
204.65	9.7791	878.68	0.0748
213.28	22.7949	922.62	124.4853
293.30	6.6138	926.04	37.5053
429.95	28.6856	1119.12	1.4759
439.29	2.6577	1625.64	4.4964
493.28	53.0325	1917.86	6.8063
558.60	0.6070	1928.73	113.9443
586.43	0.1957	2063.18	394.1219
606.04	6.3094	3238.38	9.5670
640.31	1.4378	3239.56	138.1388
646.04	25.0359	3393.43	69.4210
744.02	71.2171		

Table S33. Cartesian coordinates for the optimized geometry of isomer 3h-triplet $Ta^+C_6H_4$ followed by its predicted frequencies (cm^{-1}) and IR intensities (km/mol).

Z	x	y	z
Ta	0.0830190	0.0656210	-0.2557320
C	-1.0759160	-1.9022240	0.0835550
C	-1.7312300	-0.9610420	0.6091780
C	-1.9477610	0.3578540	0.7546000
C	-1.6676170	1.5477730	0.5024910
H	-1.8593620	2.6022170	0.4615730
C	2.0072730	-0.0028720	0.4315260
C	3.1934830	-0.0291310	0.7114760
H	4.2346110	-0.0500140	0.9535110
H	-0.9678640	-2.9598280	-0.0908810
H	-0.1371330	1.5551480	-1.2127110

Frequency (cm^{-1})	Intensity (km/mol)	Frequency (cm^{-1})	Intensity (km/mol)
62.34	1.6547	720.37	6.0139
72.22	3.6704	761.99	30.8368
166.10	20.2835	785.71	46.9802
193.42	22.4267	791.53	31.8327
196.17	15.0938	805.81	15.1677
213.26	16.1386	1051.10	6.6507
270.38	17.8806	1762.07	21.0308
404.15	10.7940	1824.60	1.3160
482.83	7.5438	2009.94	38.2752
494.46	47.3010	2046.68	328.5061
518.70	6.4631	3268.48	129.6232
555.72	14.4980	3332.10	147.1466
571.39	32.8612	3395.25	79.5716
688.35	66.5675		

Table S34. Cartesian coordinates for the optimized geometry of isomer 3h-quintet $Ta^{+}C_6H_4$ followed by its predicted frequencies (cm^{-1}) and IR intensities (km/mol).

Z	x	y	z
Ta	-0.0840720	0.1751760	-0.1873180
C	1.8827090	1.3990860	0.4413400
C	2.1388810	0.1840400	0.5241600
C	1.8040140	-1.1160230	0.3544830
C	1.1328190	-2.0997750	0.0425480
H	0.8059910	-3.1011440	-0.1506380
C	-2.1198890	-0.1195470	0.1748640
C	-3.2836560	-0.1820230	0.6416140
H	-4.2004020	-0.1920100	1.1916660
H	2.1357040	2.4398190	0.5288040
H	-1.9333230	-0.3290760	-0.9697090

Frequency (cm^{-1})	Intensity (km/mol)	Frequency (cm^{-1})	Intensity (km/mol)
35.80	0.3787	686.06	24.9018
61.96	2.9821	705.59	26.8289
100.09	16.2946	740.89	38.3763
192.38	4.3695	763.04	37.0476
198.47	4.9129	1007.94	5.0187
250.04	0.3582	1104.73	22.2196
282.20	28.4018	1680.01	4.4848
360.07	53.1705	1909.89	5.9659
411.37	0.3406	2092.32	48.9263
420.68	7.7920	2255.71	19.1602
453.31	18.3681	3304.17	158.8041
500.17	2.5791	3356.49	176.3493
539.87	11.9867	3376.18	218.6705
648.89	35.8046		

Table S35. Cartesian coordinates for the optimized geometry of isomer 3i-singlet $Ta^{+}C_6H_4$ followed by its predicted frequencies (cm^{-1}) and IR intensities (km/mol).

Z	x	y	z
C	0.7724710	-0.2555460	1.3532660
C	1.5501810	0.7912230	0.6970040
C	0.7724710	-1.5596250	0.6969940
H	1.9678600	1.6182500	1.2620790
H	0.6145620	-2.4725590	1.2621070
C	1.5501810	0.7912230	-0.6970040
C	0.7724710	-1.5596250	-0.6969940
H	1.9678600	1.6182500	-1.2620790
H	0.6145620	-2.4725590	-1.2621070
C	0.7724710	-0.2555460	-1.3532660
Ta	-0.5795380	0.1917260	0.0000000

Frequency (cm^{-1})	Intensity (km/mol)	Frequency (cm^{-1})	Intensity (km/mol)
350.56	5.4456	1007.64	0.7960
375.92	0.0000	1046.50	41.4576
384.17	0.0798	1135.57	1.1297
399.17	4.3646	1172.16	50.5950
534.57	2.1258	1180.28	0.0000
617.66	0.0000	1309.17	0.0000
677.43	41.7432	1323.13	2.2480
715.04	54.4147	1339.92	26.8495
808.62	111.1589	1391.69	5.5154
896.05	0.0006	3149.23	0.0035
922.67	10.6326	3149.50	6.0602
928.87	19.5688	3161.19	3.4707
944.09	1.6529	3163.86	1.1111
958.03	0.0000		

Table S36. Cartesian coordinates for the optimized geometry of isomer 3i-triplet $Ta^{+}C_6H_4$ followed by its predicted frequencies (cm^{-1}) and IR intensities (km/mol).

Z	x	y	z
C	-0.9112480	0.2118650	1.3913330
C	-1.4628480	-0.9102500	0.7151640
C	-0.9112480	1.4622290	0.7151650
H	-1.6330980	-1.8433450	1.2442790
H	-0.6523930	2.3747220	1.2442800
C	-1.4628480	-0.9102500	-0.7151640
C	-0.9112480	1.4622290	-0.7151650
H	-1.6330980	-1.8433450	-1.2442790
H	-0.6523930	2.3747220	-1.2442800
C	-0.9112480	0.2118650	-1.3913330
Ta	0.6026730	-0.1401220	0.0000000

Frequency (cm^{-1})	Intensity (km/mol)	Frequency (cm^{-1})	Intensity (km/mol)
327.44	2.1078	1001.68	10.2475
328.41	4.8523	1044.27	0.1532
367.78	3.8425	1132.52	1.7264
373.27	0.0000	1226.17	0.7823
497.10	11.5882	1256.37	0.0000
603.32	0.0000	1300.52	15.4353
668.98	15.2332	1337.34	3.1062
671.46	23.4699	1366.27	25.9247
847.34	5.2361	1410.61	0.0000
857.48	95.1867	3138.74	10.0848
866.89	33.4128	3138.75	1.9033
974.91	0.1461	3151.90	14.0890
975.81	1.6842	3154.52	0.3229
979.48	0.0000		

Table S37. Cartesian coordinates for the optimized geometry of isomer 3i-quintet $Ta^+C_6H_4$ followed by its predicted frequencies (cm^{-1}) and IR intensities (km/mol).

Z	x	y	z
C	1.1976450	0.0056330	1.2969010
C	1.1976450	1.2835350	0.7071730
C	1.2096650	-1.2722120	0.7071730
H	1.2314190	2.1905330	1.3015430
H	1.2519680	-2.1788530	1.3015410
C	1.1976450	1.2835350	-0.7071730
C	1.2096650	-1.2722120	-0.7071730
H	1.2314190	2.1905330	-1.3015430
H	1.2519680	-2.1788530	-1.3015410
C	1.1976450	0.0056330	-1.2969010
Ta	-0.6606330	-0.0031070	0.0000000

Frequency (cm^{-1})	Intensity (km/mol)	Frequency (cm^{-1})	Intensity (km/mol)
213.02	149.1018	992.38	7.3847
255.10	48.7183	1073.55	0.3111
262.36	8.2591	1114.65	2.9687
301.62	0.0000	1287.73	0.0000
398.03	5.9971	1335.70	1.3195
493.74	13.2871	1363.32	15.9258
519.89	22.3459	1383.71	78.6338
565.40	0.0000	1384.61	26.9530
708.92	0.0000	1475.77	0.0000
788.45	262.8968	3142.97	0.0000
817.36	11.4665	3148.16	5.1527
925.68	3.5357	3155.89	4.5404
953.40	25.9751	3156.49	1.6392
979.99	5.0336		

Table S38. Cartesian coordinates for the optimized geometry of isomer 3j-singlet $Ta^{+}C_6H_4$ followed by its predicted frequencies (cm^{-1}) and IR intensities (km/mol).

Z	x	y	z
C	1.4330940	-0.9500690	0.0000010
C	2.7762710	-0.7359180	0.0000050
H	3.6116300	-1.4268100	0.0000180
C	1.4331730	0.9500340	0.0000020
C	2.7763290	0.7357990	0.0000070
H	3.6117490	1.4266110	0.0000160
Ta	-0.3238870	0.0000310	-0.0000050
C	-2.3319650	-0.0000740	-0.6543170
C	-2.3319350	-0.0000770	0.6543530
H	-3.0547280	-0.0001240	-1.4610780
H	-3.0546650	-0.0001250	1.4611430

Frequency (cm^{-1})	Intensity (km/mol)	Frequency (cm^{-1})	Intensity (km/mol)
48.38	0.8515	871.09	0.0000
50.40	4.2645	942.31	0.0000
233.30	0.0000	965.81	1.0821
279.79	5.9120	965.81	96.3216
388.23	25.2946	1187.66	14.9295
418.45	0.0000	1228.74	48.0437
438.01	0.1517	1396.62	5.5854
551.85	2.9965	1509.16	0.4667
659.13	4.7147	1527.33	74.8576
676.47	7.3795	3174.38	2.9402
698.66	28.9364	3175.49	25.8233
734.40	86.5478	3186.36	15.3044
775.59	44.4089	3206.42	87.1829
830.75	8.2036		

Table S39. Cartesian coordinates for the optimized geometry of isomer 3j-triplet $Ta^{+}C_6H_4$ followed by its predicted frequencies (cm^{-1}) and IR intensities (km/mol).

Z	x	y	z
C	-1.2865170	0.0478310	0.8521220
C	-2.6153020	-0.4176100	0.7089580
H	-3.3674860	-0.6420800	1.4532540
C	-1.2865260	0.0478770	-0.8521220
C	-2.6153160	-0.4175590	-0.7089540
H	-3.3675240	-0.6419450	-1.4532510
Ta	0.4668090	0.3607140	0.0000050
C	1.3523830	-1.3797640	0.6566470
C	1.3523430	-1.3797580	-0.6566980
H	1.6258450	-2.0271260	1.4807880
H	1.6257480	-2.0270970	-1.4808760

Frequency (cm^{-1})	Intensity (km/mol)	Frequency (cm^{-1})	Intensity (km/mol)
100.11	0.0675	867.85	0.2009
119.55	4.6894	941.06	0.9492
165.11	1.7931	1038.47	74.0760
300.30	17.2836	1060.47	0.1109
377.74	0.8810	1149.83	1.9162
385.65	5.0900	1313.25	53.5477
472.41	15.7331	1336.15	17.1054
566.64	0.7435	1343.21	0.4919
615.63	13.3173	1542.94	14.9743
658.48	10.2848	3170.25	11.1956
694.17	18.3581	3194.76	2.8295
744.06	104.1610	3201.69	20.1589
754.10	42.9910	3211.13	8.9044
848.14	3.6805		

Table S40. Cartesian coordinates for the optimized geometry of isomer 3j-quintet $Ta^+C_6H_4$ followed by its predicted frequencies (cm^{-1}) and IR intensities (km/mol).

Z	x	y	z
C	-1.2243580	-0.0631240	-1.2597030
C	-2.4927040	-0.3775440	-0.6967550
H	-3.3609850	-0.6739750	-1.2803520
C	-1.2243560	-0.0630720	1.2597080
C	-2.4927010	-0.3775080	0.6967720
H	-3.3609820	-0.6739040	1.2803860
Ta	0.3521120	0.3075590	-0.0000070
C	1.2769070	-1.5002030	0.0000280
C	2.2467260	-0.6024530	0.0000160
H	1.1486710	-2.5757420	0.0000380
H	3.3320650	-0.6247920	0.0000180

Frequency (cm^{-1})	Intensity (km/mol)	Frequency (cm^{-1})	Intensity (km/mol)
94.20	4.4670	935.27	0.0260
114.16	0.1450	956.33	0.4755
151.49	2.6276	1039.05	103.2914
264.73	6.2790	1070.59	2.3345
317.86	1.0163	1084.04	0.9862
403.19	1.9592	1170.31	2.9040
492.09	0.6038	1325.94	43.7648
506.59	1.5378	1355.39	62.1166
616.90	12.7171	1491.06	7.3587
642.76	3.0973	3127.64	3.7746
650.05	2.5425	3145.76	13.2966
738.35	43.8144	3152.80	16.1751
749.80	27.3226	3192.85	19.9119
830.82	41.5464		

Table S41. Cartesian coordinates for the optimized geometry of isomer 3k-singlet $Ta^{+}C_6H_4$ followed by its predicted frequencies (cm^{-1}) and IR intensities (km/mol).

Z	x	y	z
C	-1.6086990	0.5065050	-1.1141670
C	-1.4112600	1.5000660	-0.0982520
C	-1.4715920	0.7031710	1.0992170
C	-2.2467530	0.0261180	0.0841300
H	-1.9010240	0.5450820	-2.1513870
H	-1.6522330	0.9348640	2.1370290
Ta	0.1280460	-0.3600060	0.0043030
C	3.0992010	0.9909230	-0.0506320
C	1.9723890	0.5251610	-0.0070010
H	4.0899770	1.3938290	-0.0872850
H	0.1161950	-2.1049790	0.3077570

Frequency (cm^{-1})	Intensity (km/mol)	Frequency (cm^{-1})	Intensity (km/mol)
22.76	8.9135	800.54	11.6421
50.40	2.3859	803.53	28.6929
126.96	41.5728	805.70	42.2799
140.60	16.6257	961.56	1.8364
232.90	12.1660	1041.32	0.0054
272.94	18.6375	1100.71	5.4864
346.89	2.8677	1252.46	0.3449
372.55	2.8300	1320.95	1.1973
489.65	56.9791	1891.25	111.7479
591.53	16.9527	2049.71	468.3667
610.87	32.8001	3244.34	97.4685
729.11	63.7638	3249.45	30.3618
784.54	28.6813	3386.24	72.0217
797.15	19.2078		

Table S42. Cartesian coordinates for the optimized geometry of isomer 3k-triplet $Ta^{+}C_6H_4$ followed by its predicted frequencies (cm^{-1}) and IR intensities (km/mol).

Z	x	y	z
C	-1.3283710	0.8920720	-1.0464500
C	-1.3683620	1.4755550	0.2761260
C	-1.7032270	0.3425980	1.0915720
C	-2.2195420	0.0986670	-0.2281920
H	-1.4068460	1.3066040	-2.0390630
H	-2.1122460	0.2343390	2.0832310
Ta	0.1206900	-0.3680100	-0.0121070
C	3.0901320	0.9695750	0.1345880
C	1.9564430	0.5316140	0.0273160
H	4.0868090	1.3482810	0.2250190
H	0.0595050	-1.8849950	-0.9151360

Frequency (cm^{-1})	Intensity (km/mol)	Frequency (cm^{-1})	Intensity (km/mol)
54.12	0.7274	792.77	3.0409
70.11	4.6493	795.28	35.8237
139.19	23.1916	801.70	63.6184
177.49	32.1931	954.80	0.3904
200.43	16.3184	1035.81	0.0335
302.51	17.1314	1094.76	5.3124
341.65	3.9027	1239.09	2.3389
391.67	2.9379	1324.73	1.8655
493.43	58.7217	1902.04	118.5494
590.49	29.6975	2047.57	435.8779
609.38	26.6254	3245.96	100.2774
715.79	66.2033	3250.85	31.8942
780.54	30.9568	3387.47	75.1424
784.28	3.7120		

Table S43. Cartesian coordinates for the optimized geometry of isomer 3k-quintet $Ta^{+}C_6H_4$ followed by its predicted frequencies (cm^{-1}) and IR intensities (km/mol).

Z	x	y	z
C	-1.5464850	0.7604280	-1.0175650
C	-1.3876010	1.4391090	0.2753700
C	-1.8224720	0.3086490	1.0586440
C	-2.2508550	-0.2536250	-0.2164840
H	-1.6225340	1.0995810	-2.0382330
H	-2.0781450	0.1485450	2.0931400
Ta	0.1321390	-0.2800960	-0.0122070
C	3.2557540	0.7286440	0.0804450
C	2.0921480	0.3651420	0.0347720
H	4.2783380	1.0399970	0.1175740
H	-0.2667270	-1.9311900	-0.5724970

Frequency (cm^{-1})	Intensity (km/mol)	Frequency (cm^{-1})	Intensity (km/mol)
20.62	2.1300	789.73	29.1920
46.30	3.7110	836.00	19.4938
142.56	37.6968	859.86	5.2245
154.44	17.3280	947.65	3.6483
227.30	2.9516	1077.43	0.2081
311.88	13.7959	1109.41	7.6225
337.21	13.6718	1212.25	17.3959
357.04	9.5436	1231.77	24.8433
464.93	59.3050	1766.43	55.1653
517.14	37.5355	2047.23	430.0289
687.63	23.8320	3238.77	65.2790
697.59	15.8446	3247.95	43.6533
739.61	1.7819	3390.88	69.7526
781.07	30.6042		

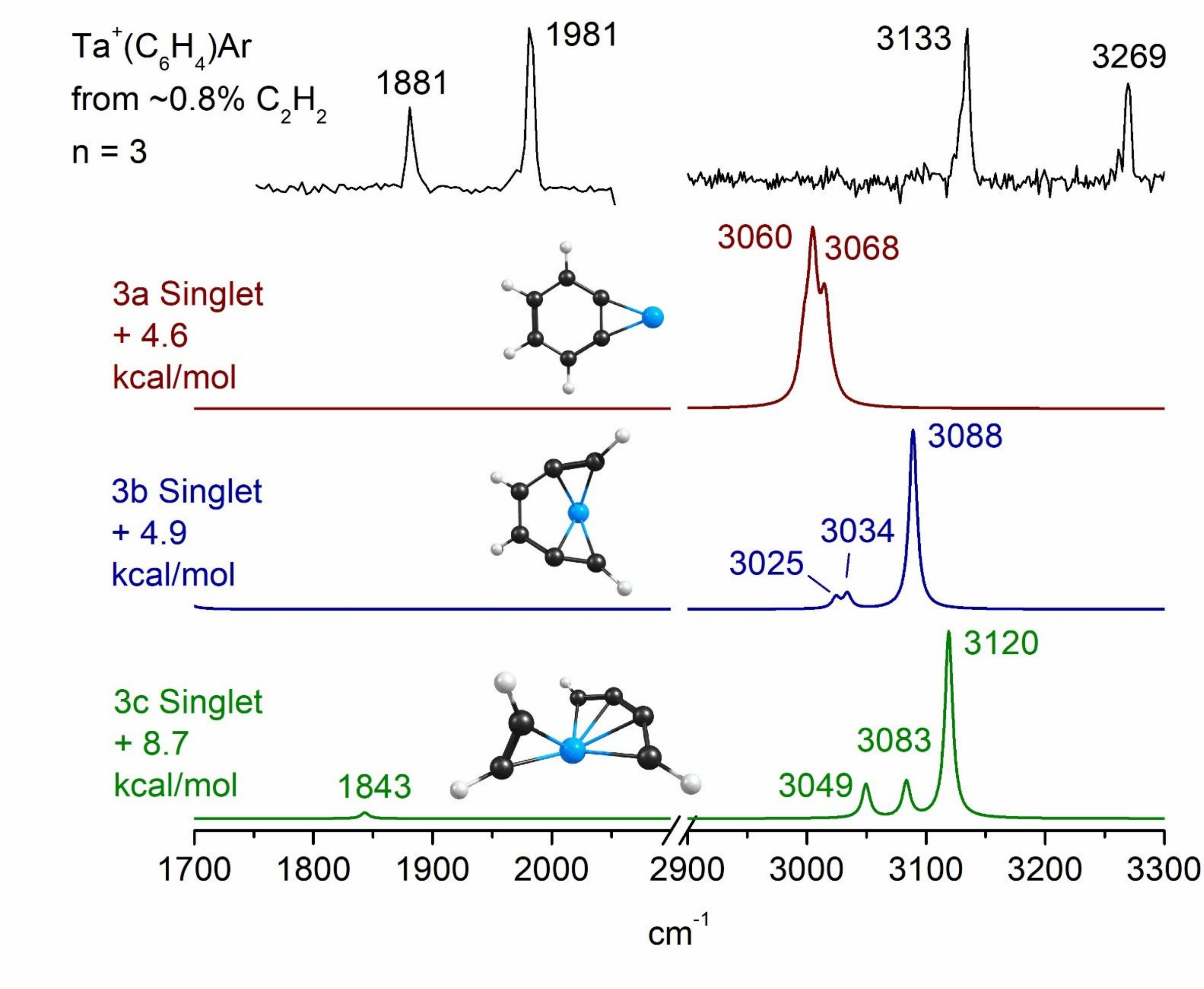

Figure S80. The experimental IR photodissociation spectrum of $Ta^+C_6H_4Ar$ compared with simulated spectra for singlet isomers 3a-3c of $Ta^+C_6H_4$.

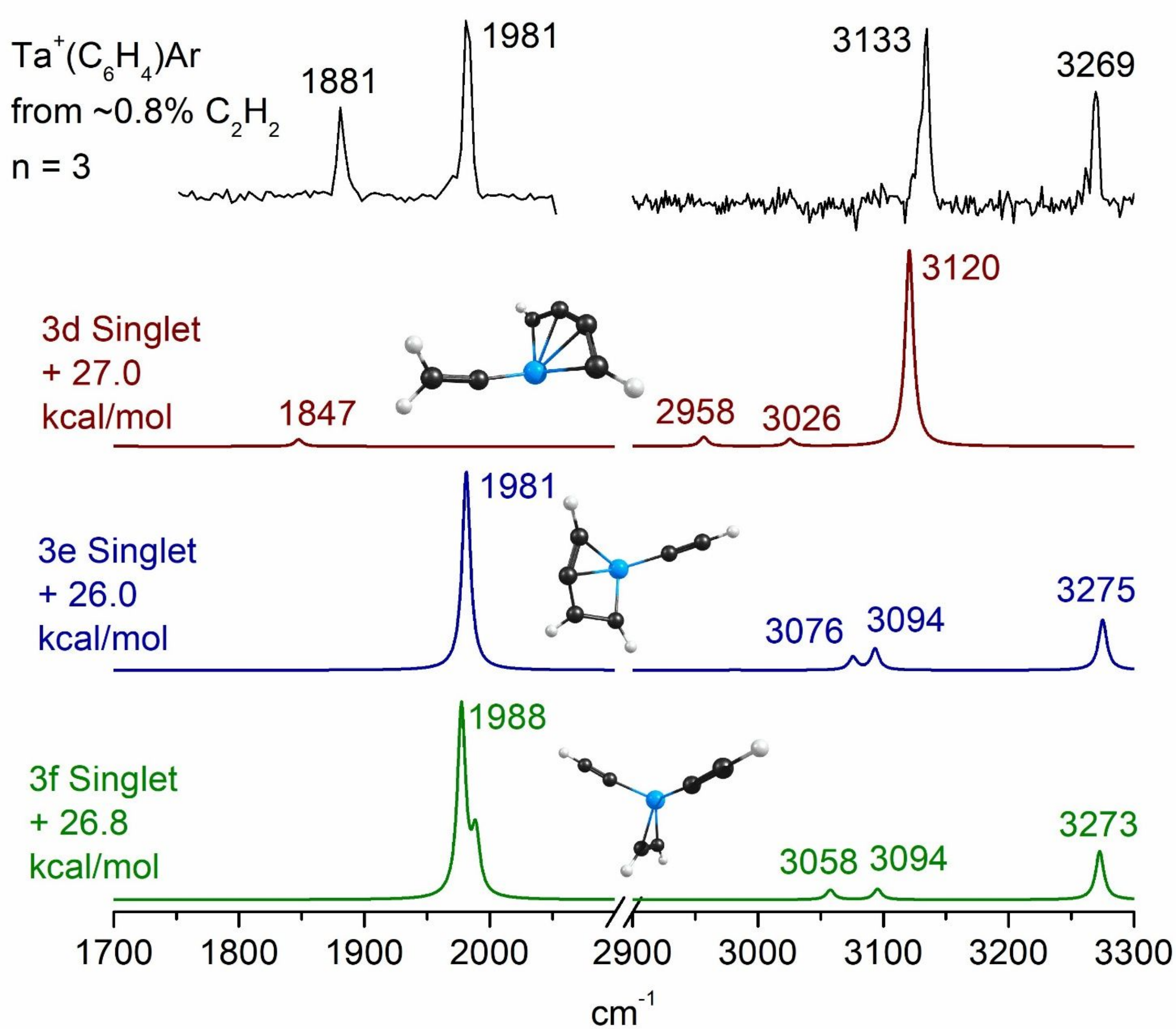

Figure S81. The experimental IR photodissociation spectrum of $Ta^{+}C_6H_4Ar$ compared with simulated spectra for singlet isomers 3d-3f of $Ta^{+}C_6H_4$.

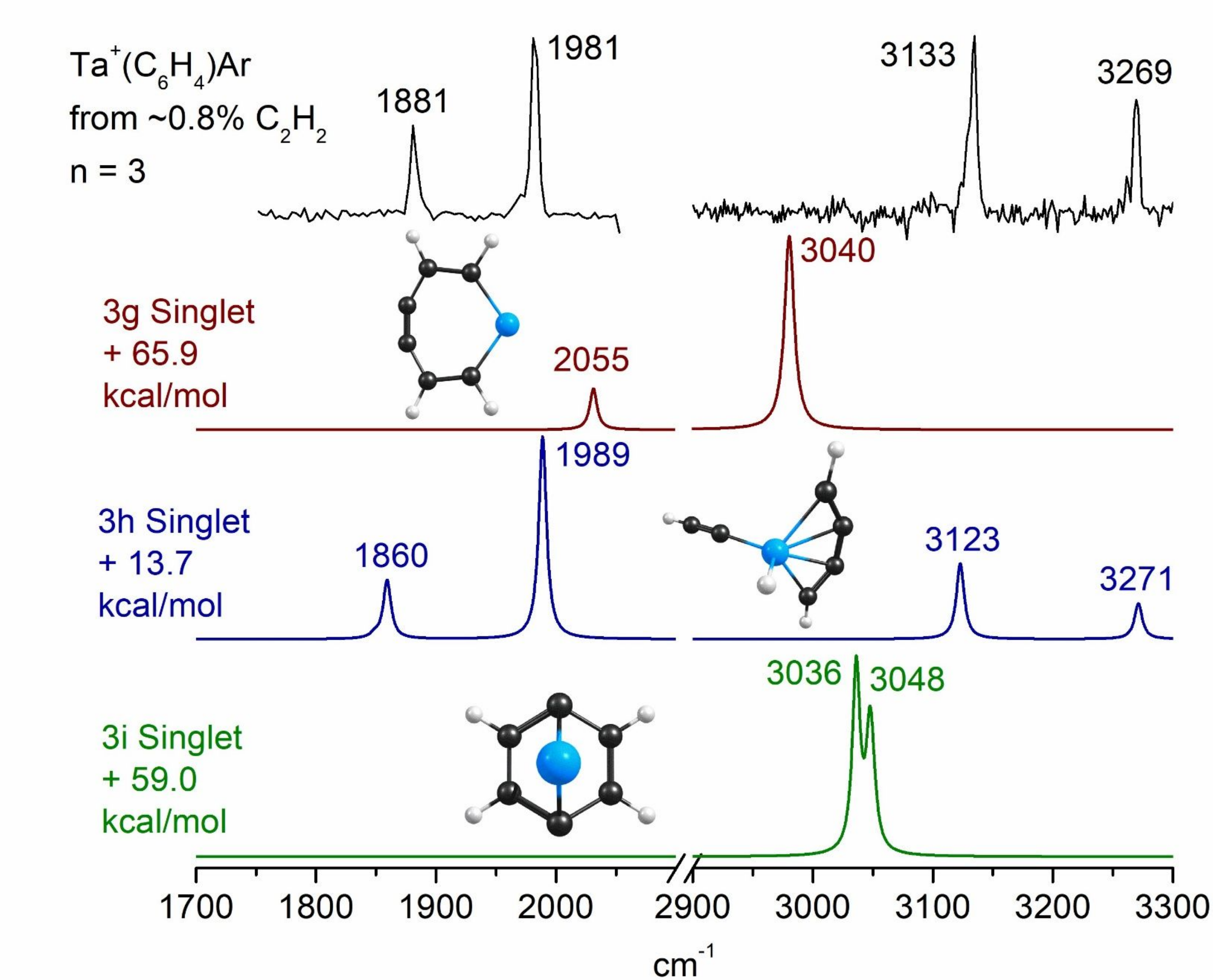

Figure S82. The experimental IR photodissociation spectrum of $Ta^+C_6H_4Ar$ compared with simulated spectra for singlet isomers 3g-3i of $Ta^+C_6H_4$.

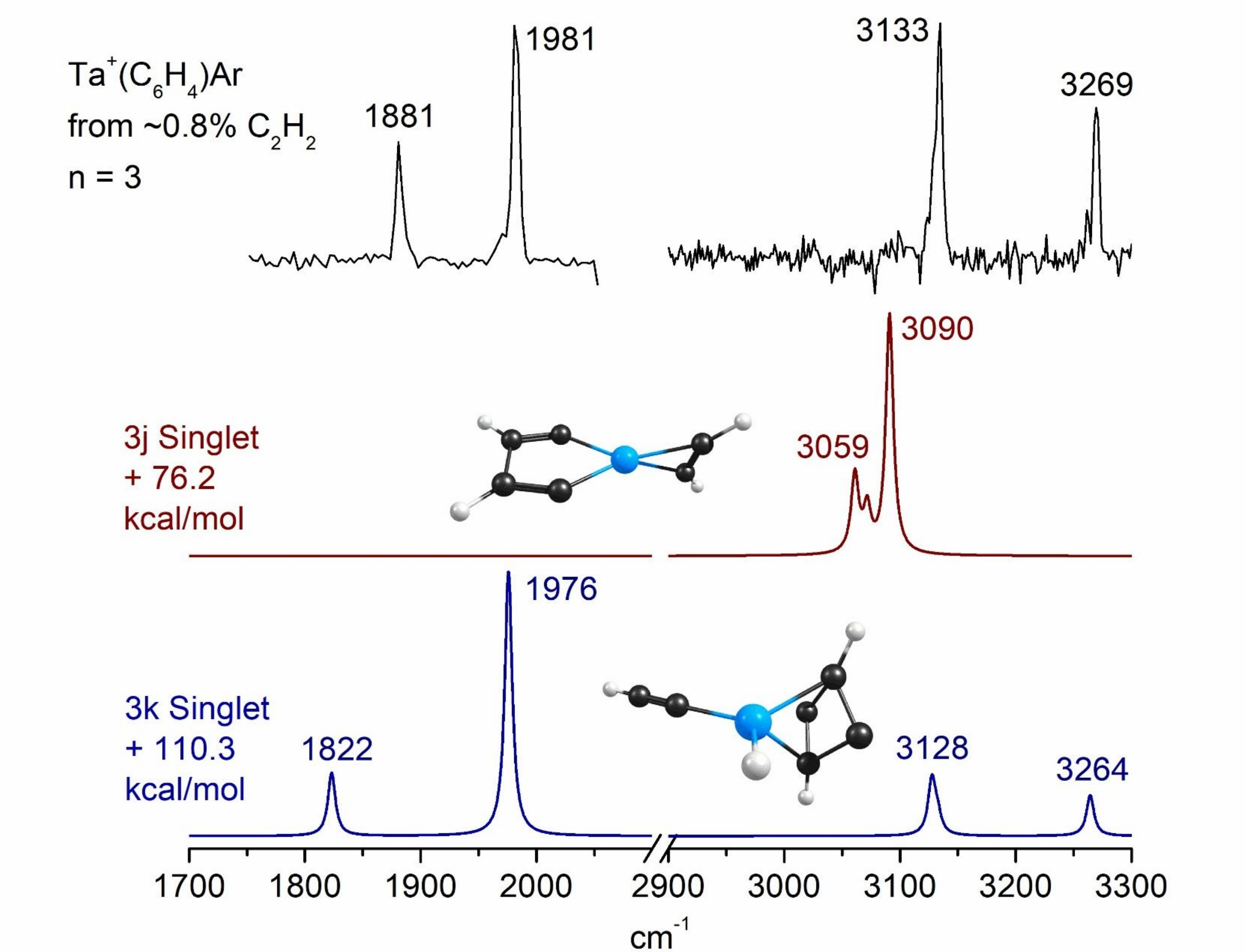

Figure S83. The experimental IR photodissociation spectrum of $Ta^+C_6H_4Ar$ compared with simulated spectra for singlet isomers 3j-3k of $Ta^+C_6H_4$.

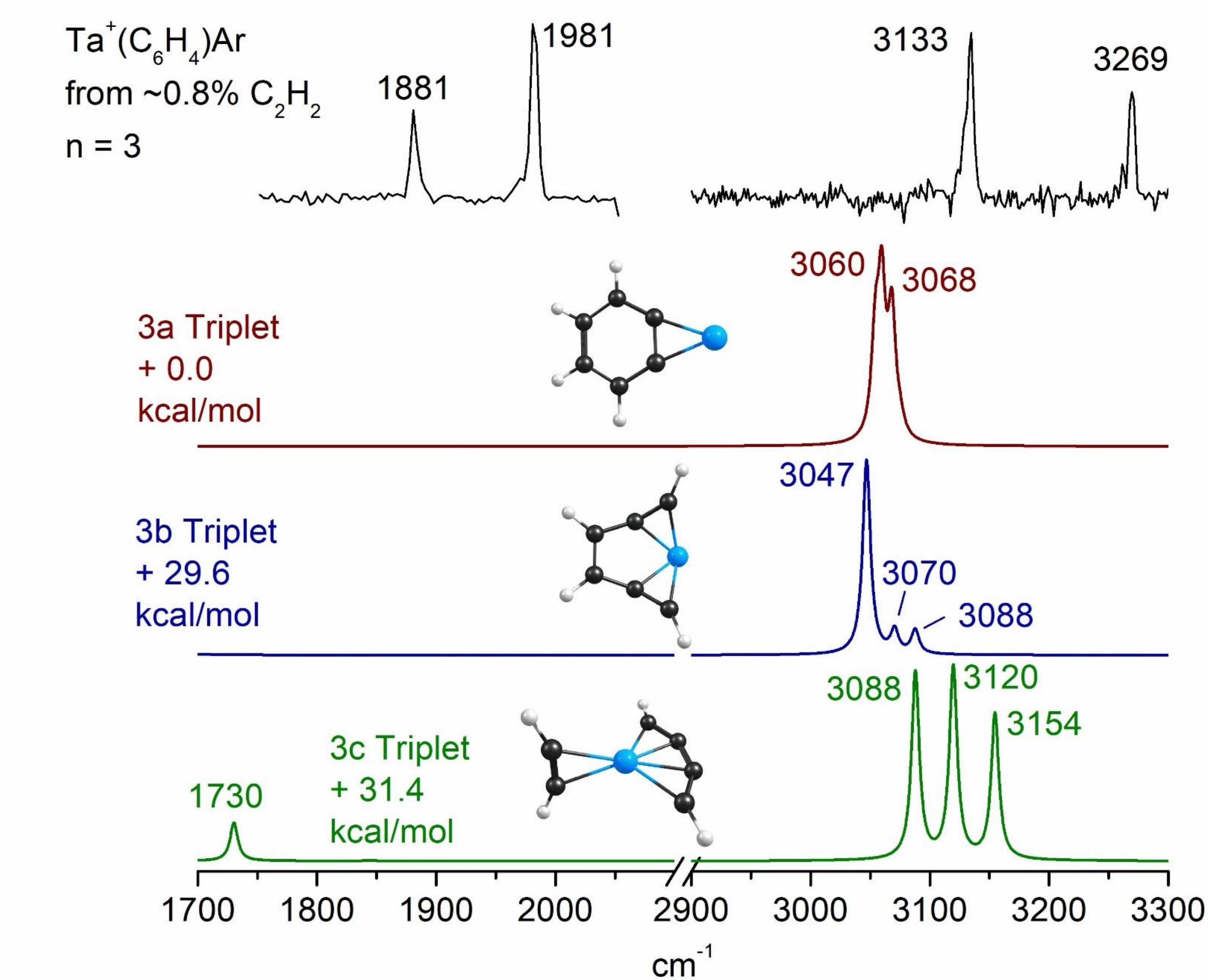

Figure S84. The experimental IR photodissociation spectrum of $Ta^+C_6H_4Ar$ compared with simulated spectra for triplet isomers 3a-3c of $Ta^+C_6H_4$.

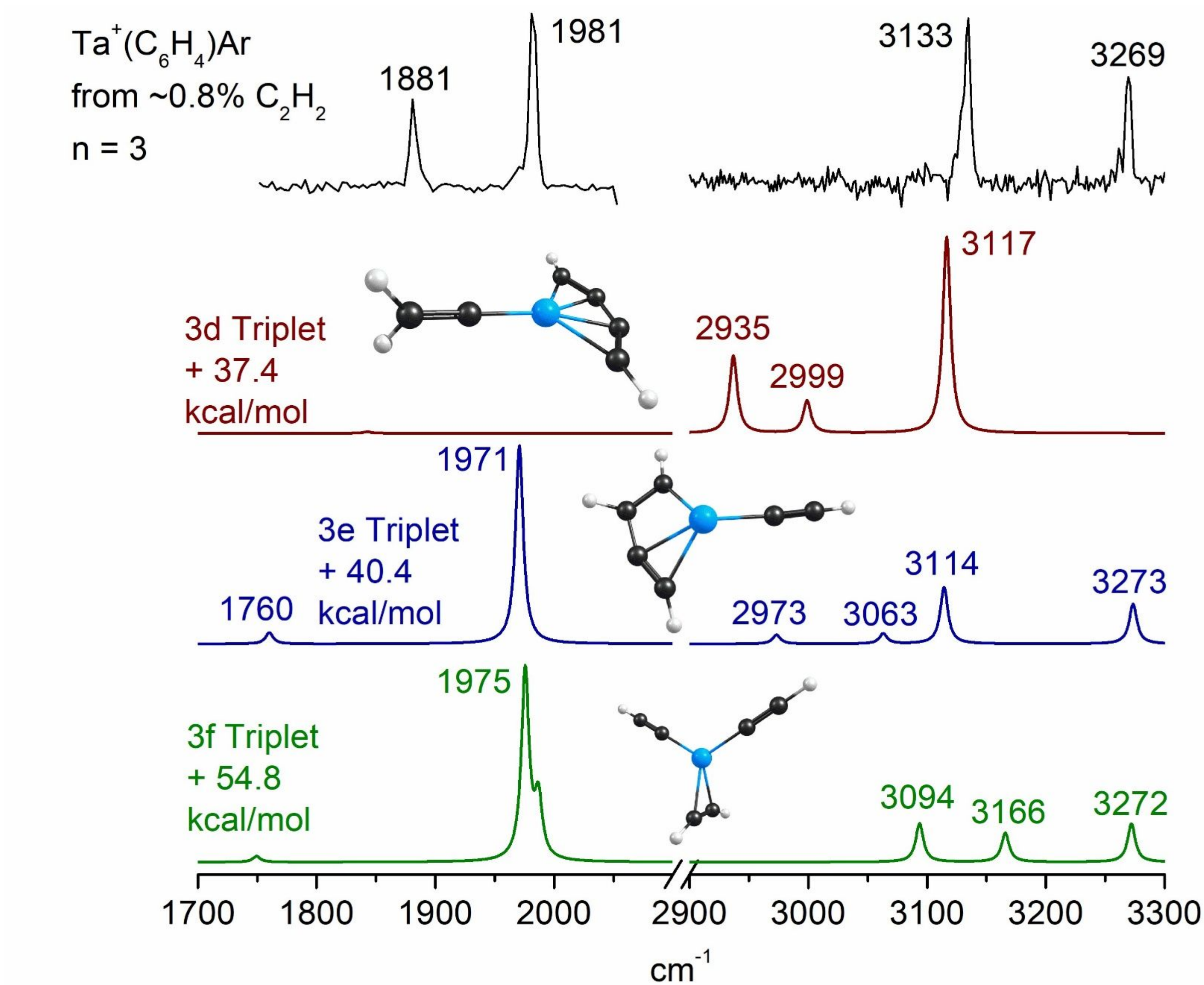

Figure S85. The experimental IR photodissociation spectrum of $Ta^+C_6H_4Ar$ compared with simulated spectra for triplet isomers 3d-3f of $Ta^+C_6H_4$.

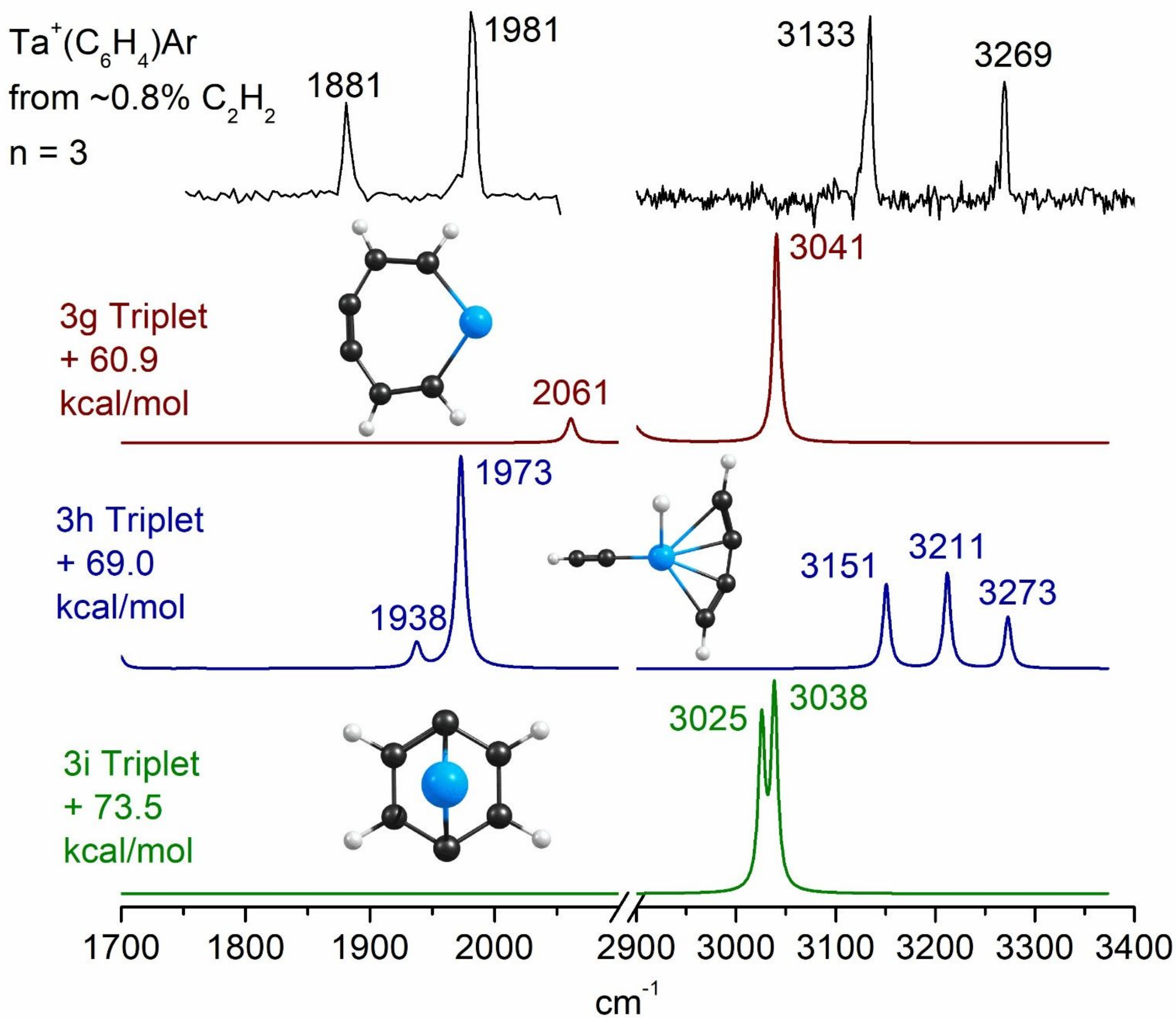

Figure S86. The experimental IR photodissociation spectrum of $Ta^{+}C_6H_4Ar$ compared with simulated spectra for triplet isomers 3g-3i of $Ta^{+}C_6H_4$.

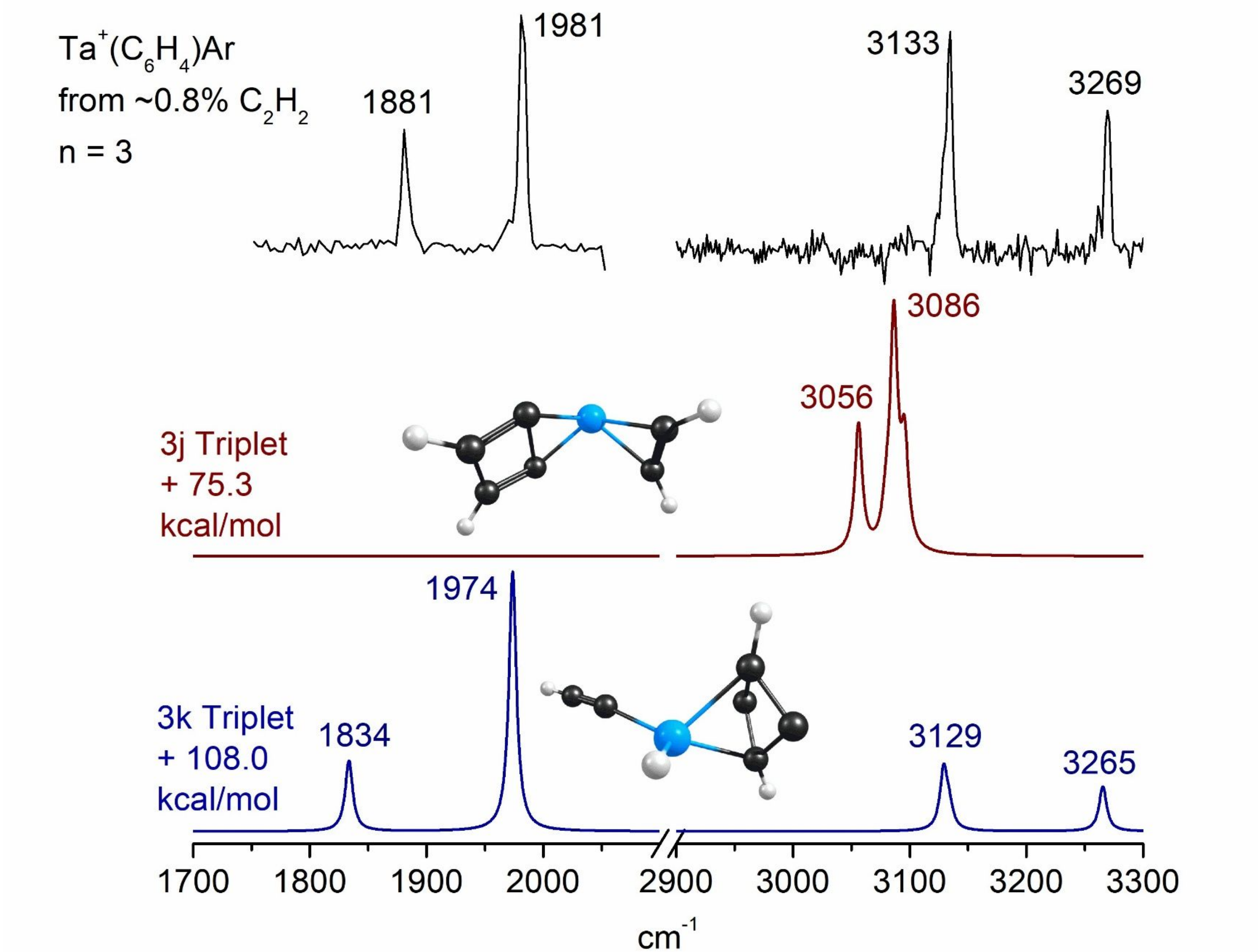

Figure S87. The experimental IR photodissociation spectrum of $Ta^+C_6H_4Ar$ compared with simulated spectra for triplet isomers 3j-3k of $Ta^+C_6H_4$.

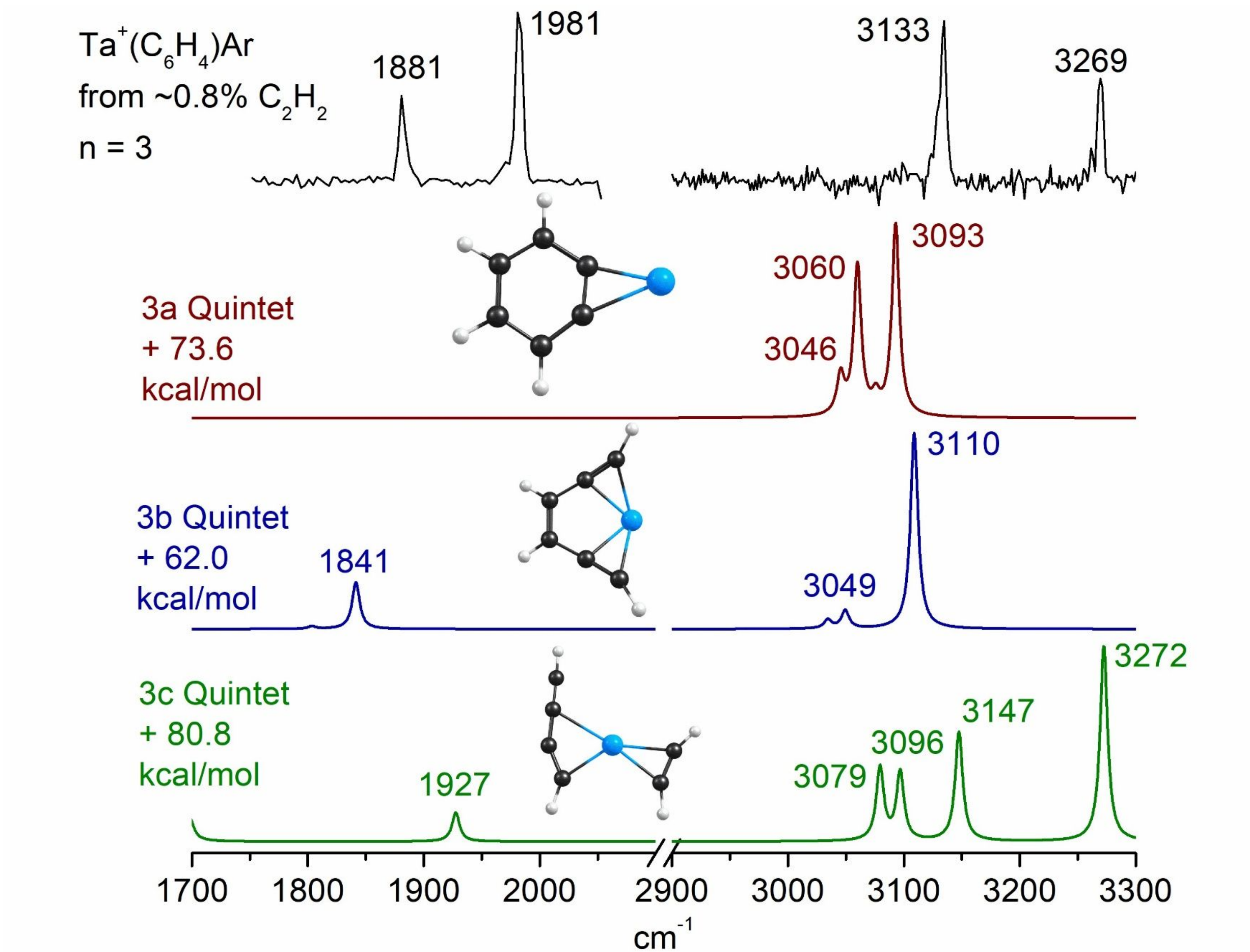

Figure S88. The experimental IR photodissociation spectrum of $Ta^+C_6H_4Ar$ compared with simulated spectra for quintet isomers 3a-3c of $Ta^+C_6H_4$.

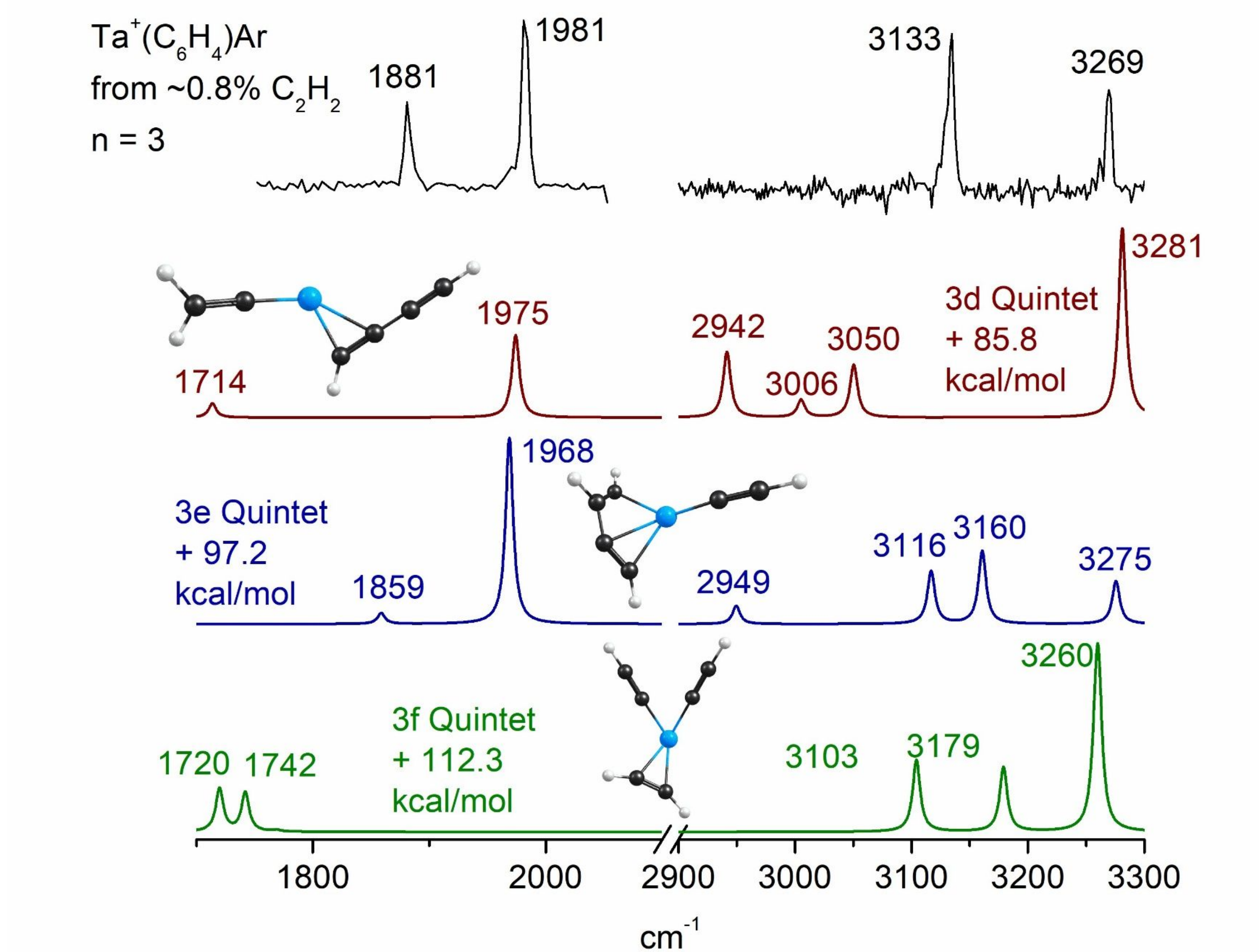

Figure S89. The experimental IR photodissociation spectrum of $Ta^+C_6H_4Ar$ compared with simulated spectra for quintet isomers 3d-3f of $Ta^+C_6H_4$.

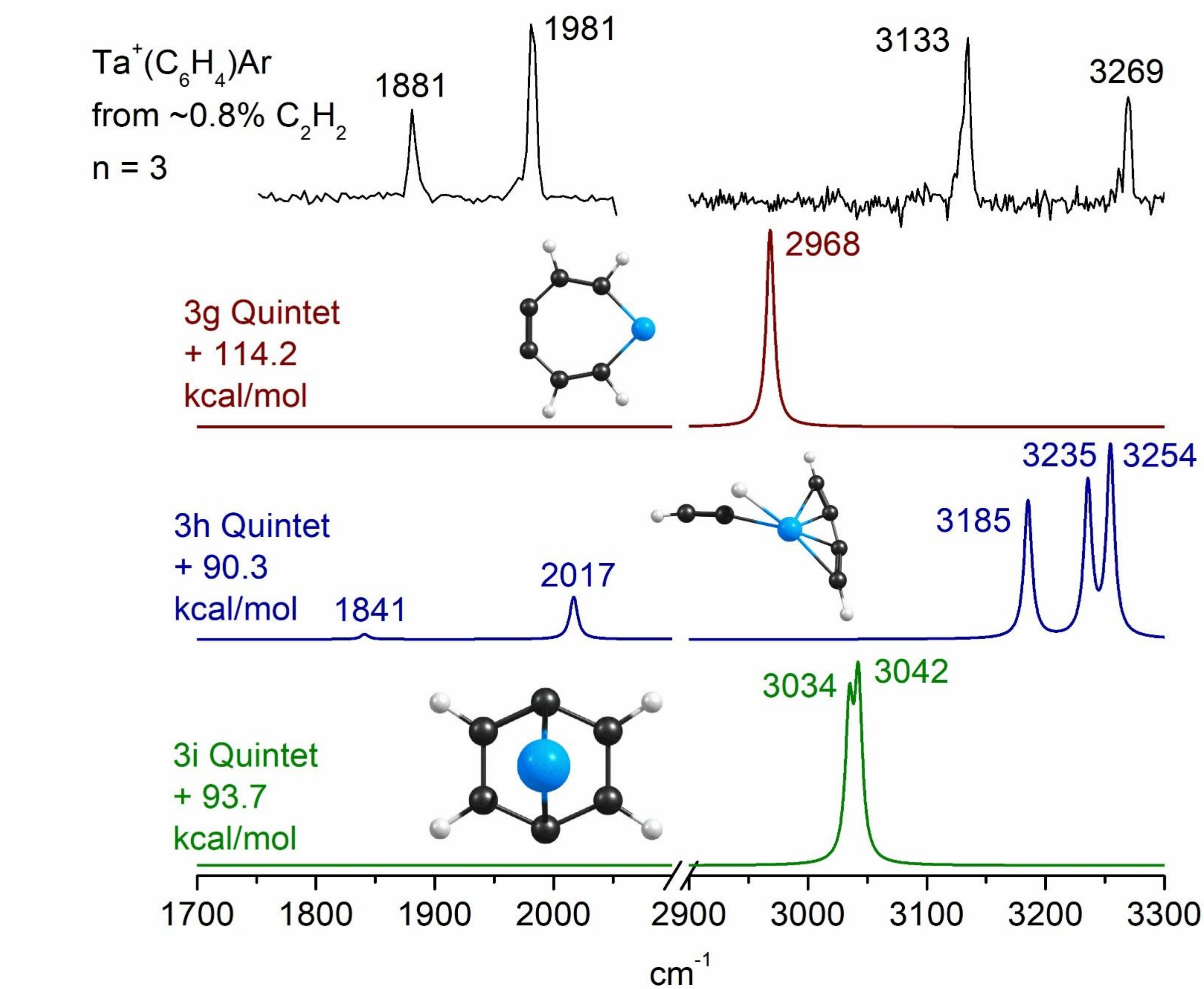

Figure S90. The experimental IR photodissociation spectrum of $Ta^+C_6H_4Ar$ compared with simulated spectra for quintet isomers 3g-3i of $Ta^+C_6H_4$.

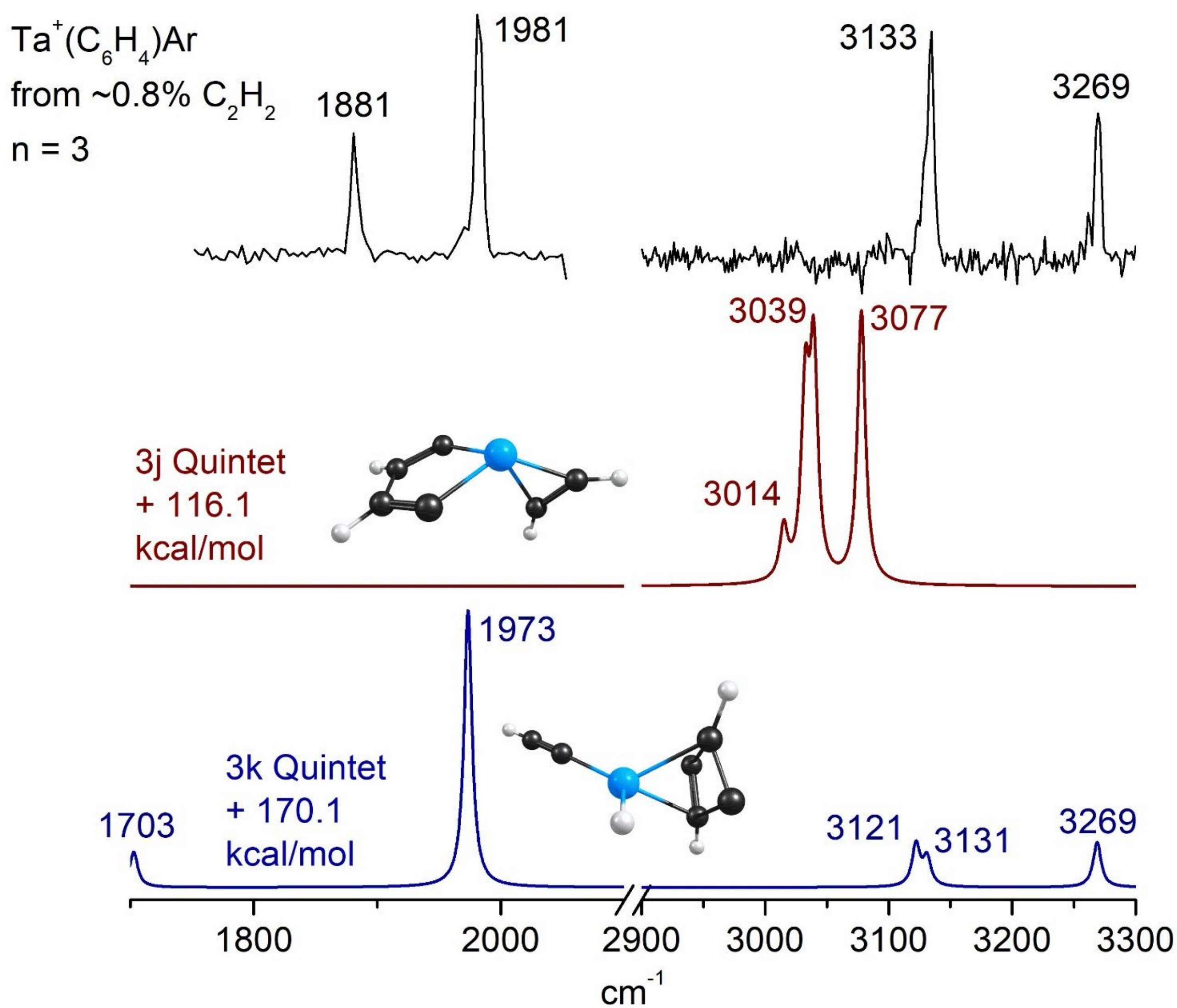

Figure S91. The experimental IR photodissociation spectrum of $Ta^+C_6H_4Ar$ compared with simulated spectra for quintet isomers 3j-3k of $Ta^+C_6H_4$.

Table S44. $Ta^+C_8H_6$ electronic energy calculated at the B3LYP/cc-pVTZ(-pp) level with Stuttgardt/Koeln pseudopotential.

Isomer	2s + 1	E (hartree)	Relative E (kcal/mol)
4a	1	-365.472897	+0.0
4a	3	-365.469133	+2.4
4a	5	DNC*	-
4b	1	-365.472407	+0.3
4b	3	-365.385403	+54.9
4b	5	-365.366842	+66.6
4c	1	DNC*	-
4c	3	DNC*	-
4c	5	DNC*	-

*Did not converge

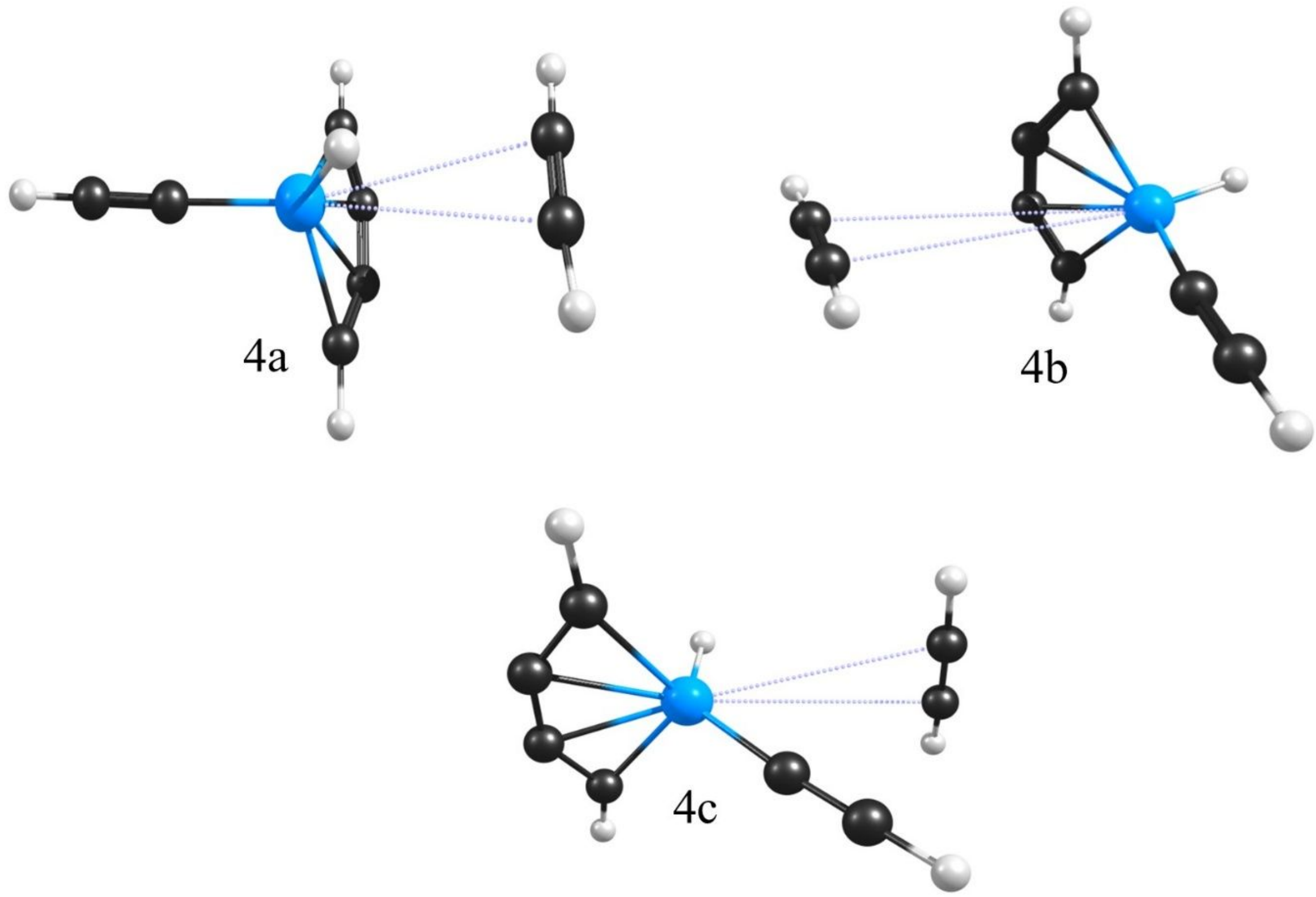

Figure S92. Optimized* geometries of $Ta^+C_8H_6$.
*Structure 4c could not be optimized as the calculations did not converge; the input geometry is given.

Table S45. Cartesian coordinates for the optimized geometry of isomer 4a-singlet Ta+C8H6 followed by its predicted frequencies (cm-1) and IR intensities (km/mol).

Z	x	y	z
Ta	0.2649440	0.1479740	0.1332080
C	-0.3163130	0.4232370	-1.9104210
C	-0.7211210	-0.7715750	-1.5943230
C	-0.7373740	-1.6532420	-0.6158020
C	-0.3573870	-1.8480970	0.6124130
H	-0.3730010	-2.6505010	1.3341910
C	2.3101320	0.1999620	0.2061320
C	3.5162640	0.3136760	0.3259700
H	4.5744430	0.4190100	0.4362190
H	-0.3081320	1.0558110	-2.7849970
H	-0.3777410	1.3826570	1.2095320
C	-3.1415460	1.1207520	0.2117470
C	-3.2722410	0.1596100	0.9165390
H	-3.0936790	1.9980190	-0.3898300
H	-3.4452630	-0.6730260	1.5571660

Frequency (cm-1)	Intensity (km/mol)	Frequency (cm-1)	Intensity (km/mol)
12.94	5.2656	741.02	88.5415
18.26	0.1356	772.51	70.8678
46.86	1.9339	782.16	29.0772
60.88	2.3638	783.34	28.9847
97.62	1.0302	802.75	127.1230
110.21	3.3140	877.01	0.0670
129.81	3.2350	926.22	95.9781
213.70	9.3800	927.60	54.3649
225.59	19.5835	1122.85	1.6511
292.98	5.0069	1626.93	4.2853
384.61	27.9617	1916.86	4.2876
439.86	0.9226	1939.32	131.2273
498.05	67.8879	2056.75	59.1698
536.77	0.0501	2064.64	370.5749
581.56	0.0344	3238.83	7.4446
594.65	10.1233	3240.18	111.2782
637.43	1.8636	3383.37	114.9152
657.77	0.2520	3395.97	70.3593
671.52	61.9243	3486.48	2.4103
684.56	2.1963		

Table S46. Cartesian coordinates for the optimized geometry of isomer 4a-triplet $Ta^+C_8H_6$ followed by its predicted frequencies (cm^{-1}) and IR intensities (km/mol).

Z	x	y	z
Ta	-0.1429930	0.0529370	-0.0077190
C	0.5711470	1.7883670	1.2388200
C	1.5492860	1.5229210	0.4746570
C	2.0913950	0.6688960	-0.4178250
C	2.3344110	-0.3038380	-1.1323830
H	2.7366860	-1.0767010	-1.7519070
C	0.0295570	-1.9491480	0.4447430
C	0.1510150	-3.1407590	0.6834970
H	0.2453770	-4.1815910	0.9016780
H	0.2276410	2.4874600	1.9835190
H	-1.9047210	0.4320390	0.9650720
C	-2.4369550	0.5873130	-0.0328010
C	-1.8147390	0.3565330	-1.1836680
H	-3.4596850	0.8832350	0.1847040
H	-2.2575070	0.4094210	-2.1698050

Frequency (cm^{-1})	Intensity (km/mol)	Frequency (cm^{-1})	Intensity (km/mol)
60.01	0.8340	700.39	40.2350
77.96	1.0949	772.66	29.9751
89.22	4.7735	804.24	55.1248
104.03	5.4063	869.30	15.1769
118.31	6.1181	930.65	50.9688
139.45	0.5302	984.48	8.4934
238.10	16.0198	1001.62	12.3751
243.32	10.1021	1184.35	27.2027
266.69	10.3756	1458.23	14.3543
330.56	8.2114	1683.70	15.8484
351.66	3.2161	1820.14	18.9652
431.17	1.3899	1998.46	221.9419
463.08	7.5520	2033.00	34.2722
475.54	30.9333	2569.76	13.4664
505.36	5.3132	3145.77	13.9721
526.44	19.2615	3198.67	25.4890
617.17	8.7456	3255.42	95.5393
621.68	52.4895	3378.35	169.5129
668.37	20.1663	3403.85	76.0352
697.24	20.9122		

Table S47. Cartesian coordinates for the optimized geometry of isomer 4b-singlet $Ta^{+}C_8H_6$ followed by its predicted frequencies (cm^{-1}) and IR intensities (km/mol).

Z	x	y	z
Ta	0.2649440	0.1479740	0.1332080
C	-0.3163130	0.4232370	-1.9104210
C	-0.7211210	-0.7715750	-1.5943230
C	-0.7373740	-1.6532420	-0.6158020
C	-0.3573870	-1.8480970	0.6124130
H	-0.3730010	-2.6505010	1.3341910
C	2.3101320	0.1999620	0.2061320
C	3.5162640	0.3136760	0.3259700
H	4.5744430	0.4190100	0.4362190
H	-0.3081320	1.0558110	-2.7849970
H	-0.3777410	1.3826570	1.2095320
C	-3.1415460	1.1207520	0.2117470
C	-3.2722410	0.1596100	0.9165390
H	-3.0936790	1.9980190	-0.3898300
H	-3.4452630	-0.6730260	1.5571660

Frequency (cm^{-1})	Intensity (km/mol)	Frequency (cm^{-1})	Intensity (km/mol)
7.89	0.4755	742.88	72.1275
15.38	0.9432	771.80	79.0206
22.64	0.1121	783.92	161.6543
50.99	0.4602	784.18	33.5280
78.48	3.7270	784.65	2.5036
83.06	3.9054	875.17	0.1510
84.91	2.3581	923.21	123.8011
203.43	10.8328	926.21	36.7377
212.75	22.0660	1119.96	1.6816
290.55	3.4861	1625.52	4.3409
433.25	26.7695	1918.51	8.9354
441.78	2.6539	1927.88	116.2058
491.76	50.4575	2063.25	288.5703
559.11	0.6718	2065.29	83.9015
582.82	0.3364	3240.16	7.8891
607.33	9.0096	3241.37	130.8166
641.11	1.2333	3390.16	101.4636
645.82	24.4804	3395.24	66.7916
653.50	0.0346	3492.80	0.9282
668.48	0.4769		

Table S48. Cartesian coordinates for the optimized geometry of isomer 4b-triplet $Ta^{+}C_8H_6$ followed by its predicted frequencies (cm^{-1}) and IR intensities (km/mol).

Z	x	y	z
Ta	0.7498600	-0.2256440	0.1952680
C	-0.9796170	0.8446780	1.2472380
C	-1.4972380	0.1028400	0.3659140
C	-1.3308250	-0.9174090	-0.4993600
C	-0.6595420	-1.8070060	-1.0487230
H	-0.4663260	-2.7059900	-1.5995030
C	2.1706520	1.0412520	-0.5615340
C	3.1098150	1.7371290	-0.9067880
H	3.9358410	2.3445620	-1.2085630
H	-1.1404420	1.6052890	1.9929810
H	1.4011120	-1.8765440	0.2773830
C	-4.3369350	1.1864160	-0.7892070
C	-4.7006380	0.3954810	0.0347690
H	-4.0581340	1.8997430	-1.5286960
H	-5.0658440	-0.2953550	0.7579590

Frequency (cm^{-1})	Intensity (km/mol)	Frequency (cm^{-1})	Intensity (km/mol)
13.57	0.3319	682.25	60.6717
34.78	0.3599	708.07	19.0880
40.06	0.2433	761.71	28.8110
54.75	7.7127	769.09	20.9849
63.98	4.6538	774.16	93.8804
73.81	5.7355	781.60	113.9183
86.61	0.6123	787.40	21.8462
123.79	33.2663	788.12	116.3099
190.30	16.7180	1041.28	3.7697
197.30	18.9672	1796.83	29.6385
221.49	15.1260	1829.31	1.4194
279.85	15.6199	2021.45	36.8229
357.31	49.2422	2047.71	357.2079
465.14	2.7378	2056.58	48.0527
492.32	48.8958	3266.59	102.2077
517.08	5.5025	3341.34	155.4744
542.01	30.1599	3386.62	115.2951
580.90	43.5317	3397.73	81.1117
657.39	0.0137	3489.16	0.0370
673.29	0.4163		

Table S49. Cartesian coordinates for the optimized geometry of isomer 4b-quintet $Ta^+C_8H_6$ followed by its predicted frequencies (cm^{-1}) and IR intensities (km/mol).

Z	x	y	z
Ta	0.7753160	-0.2545530	-0.1948660
C	-0.2537050	-1.9630820	0.9262570
C	-1.1829650	-1.2707980	0.3655520
C	-1.3879490	-0.2141650	-0.4188190
C	-2.3869290	0.6519440	-0.9152930
H	-2.2256560	1.0892560	-1.8940380
C	1.7075930	1.4645120	0.4480210
C	2.3378420	2.4643210	0.7455710
H	2.8950250	3.3381190	1.0054630
H	-0.0311790	-2.8345290	1.5109940
H	1.7162360	-1.6486210	0.3003790
C	-3.5172020	1.0475820	-0.1860880
C	-3.8611550	0.6322700	1.0553210
H	-4.1774390	1.7618510	-0.6771080
H	-3.5082480	0.0008150	1.8564190

Frequency (cm^{-1})	Intensity (km/mol)	Frequency (cm^{-1})	Intensity (km/mol)
24.85	0.6707	757.62	65.7927
56.49	0.1064	784.82	30.4782
70.06	2.6272	810.87	25.5836
110.84	2.8770	862.17	60.2257
143.37	2.8440	913.16	8.8931
179.26	81.6121	958.66	7.1485
199.67	18.5196	1124.77	13.3916
228.00	3.0219	1256.34	0.3403
297.18	67.3968	1367.78	5.9925
335.11	8.9242	1414.20	14.9906
372.75	91.2117	1476.47	122.8743
415.64	16.7951	1742.80	261.4307
483.76	50.2702	1882.38	27.8545
498.57	29.1731	2046.13	331.8997
561.02	8.9631	3103.98	2.3811
634.62	25.0449	3163.39	5.9121
671.29	1.8500	3238.33	7.6758
691.75	20.4120	3315.65	95.2683
700.81	70.1367	3400.97	74.8740
757.03	41.0540		

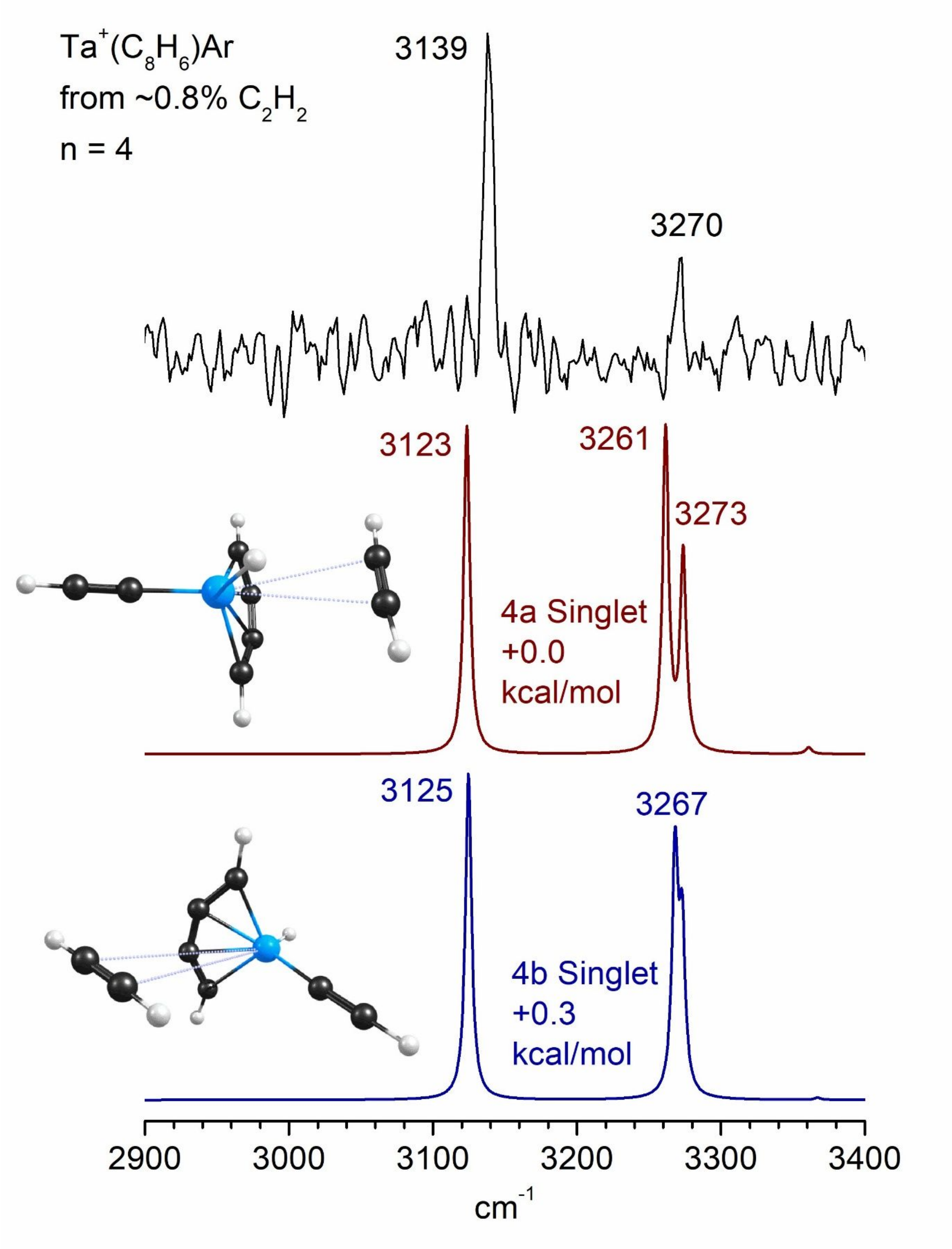

Figure S93. The experimental IR photodissociation spectrum of $Ta^+C_8H_4Ar$ compared with simulated spectra for singlet isomers 4a-4b of $Ta^+C_8H_4$.

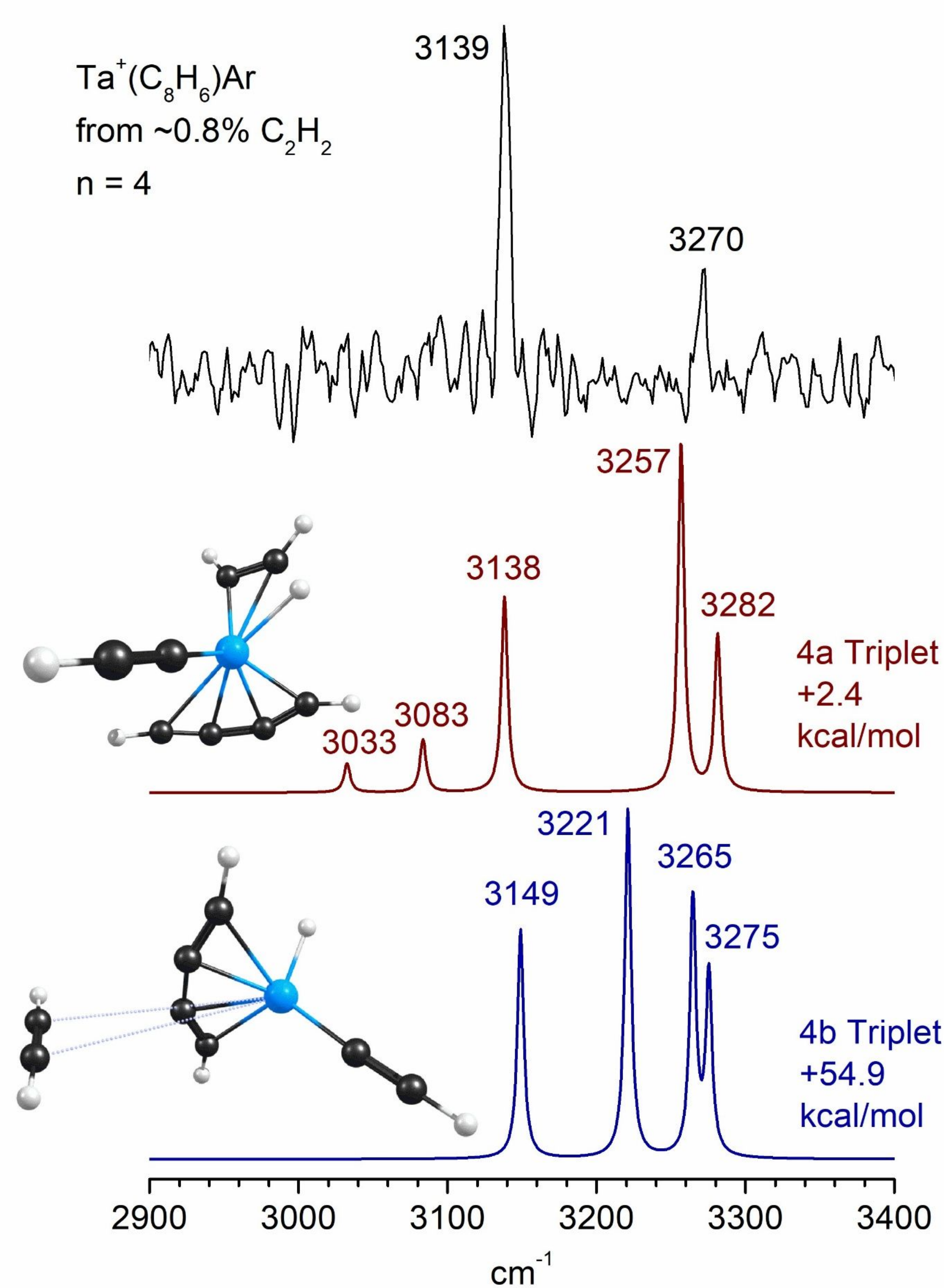

Figure S94. The experimental IR photodissociation spectrum of $Ta^+C_8H_4Ar$ compared with simulated spectra for triplet isomers 4a-4b of $Ta^+C_8H_4$.

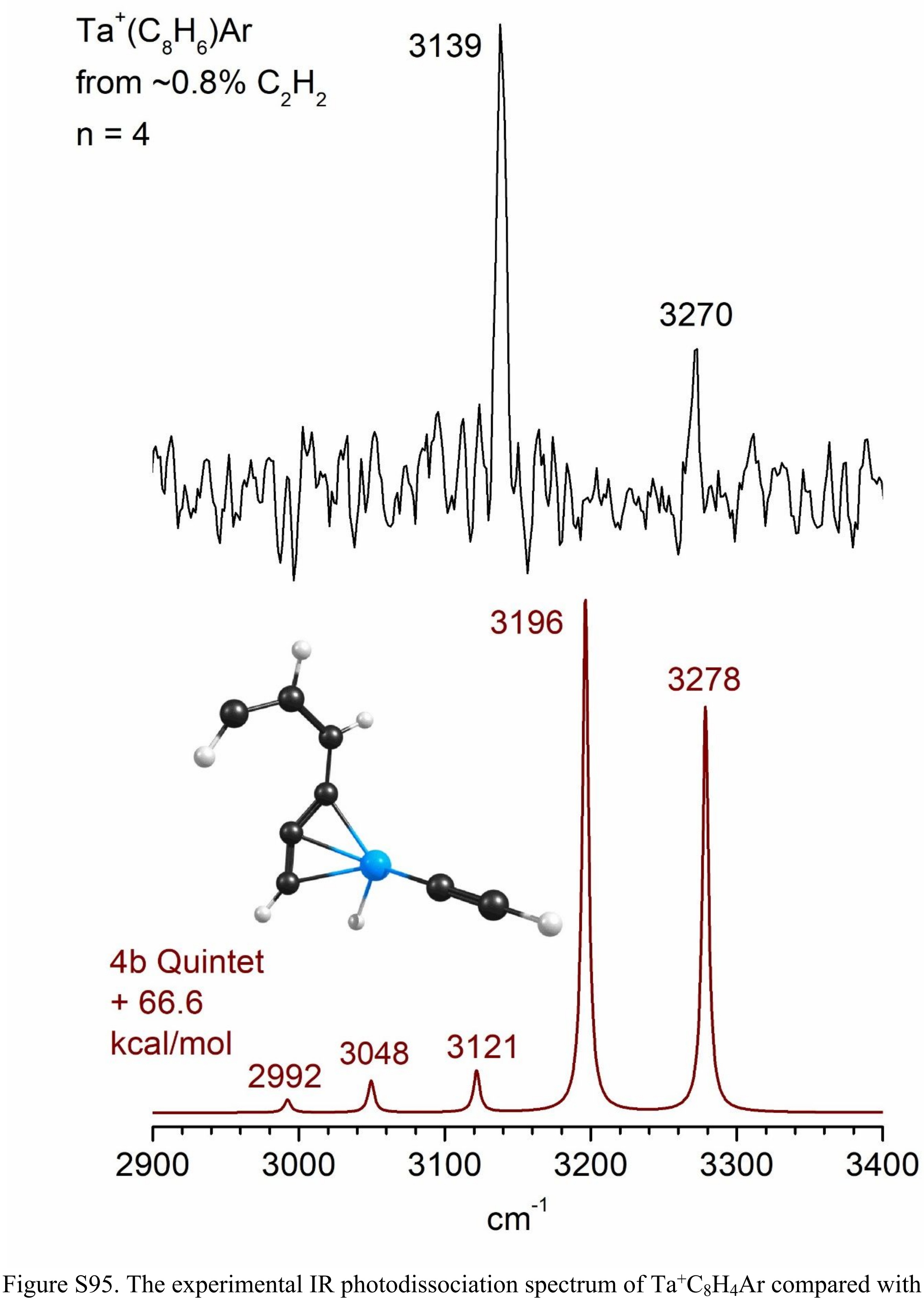

Figure S95. The experimental IR photodissociation spectrum of $Ta^+C_8H_4Ar$ compared with simulated spectra for quintet isomer 4b of $Ta^+C_8H_4$.
